# Nomenclature for factors of the HLA system, 2010

**DOI:** 10.1111/j.1399-0039.2010.01466.x

**Published:** 2010-04

**Authors:** S G E Marsh, E D Albert, W F Bodmer, R E Bontrop, B Dupont, H A Erlich, M Fernández-Viña, D E Geraghty, R Holdsworth, C K Hurley, M Lau, K W Lee, B Mach, M Maiers, W R Mayr, C R Müller, P Parham, E W Petersdorf, T Sasazuki, J L Strominger, A Svejgaard, P I Terasaki, J M Tiercy, J Trowsdale

**Affiliations:** SGE Marsh, Anthony Nolan Research InstituteLondon, UK (Chairman); ED AlbertMunich, Germany; WF Bodmer, Weatherall Institute of Molecular Medicine, Oxford UniversityOxford, UK; RE Bontrop, Biomedical Primate Research CentreRijswijk, The Netherlands; B Dupont, Sloan-Kettering Institute for Cancer ResearchNew York, USA; HA Erlich, Roche Molecular SystemsAlameda, USA; M Fernández-Viña, MD AndersonHouston, USA; DE Geraghty, Fred Hutchinson Cancer CenterSeattle, USA; R Holdsworth, Victorian Transplantation and Immunogenetics ServiceMelbourne, Australia; CK Hurley, Georgetown UniversityWashington, USA; M Lau, UCLA School of MedicineLos Angeles, USA; KW Lee, Hallym UniversityAnyang City, South Korea; B Mach, University of GenevaGeneva, Switzerland; M Maiers, National Marrow Donor ProgramMinneapolis, USA; WR Mayr, Medical University of ViennaVienna, Austria; CR Müller, Zentrales Knochenmarkspender-RegisterUlm, Germany; P Parham, Stanford University School of MedicineStanford, USA; EW Petersdorf, Fred Hutchinson Cancer CenterSeattle, USA; T Sasazuki, International Medical Centre of JapanTokyo, Japan; JL Strominger, Harvard UniversityCambridge, USA; A Svejgaard, State University HospitalCopenhagen, Denmark; PI TerasakiLos Angeles, USA; JM Tiercy, Hôpital Cantonal UniversitaireGeneva, Switzerland; J Trowsdale, Cambridge UniversityCambridge, UK

The WHO Nomenclature Committee for Factors of the HLA System met following the 14^th^ International HLA and Immunogenetics Workshop in Melbourne, Australia in December 2005 and Buzios, Brazil during the 15^th^ International HLA and Immunogenetics Workshop in September 2008. This report documents the additions and revisions to the nomenclature of HLA specificities following the principles established in previous reports (1–18).

## 1. Naming of HLA genes and alleles

A number of HLA gene fragments have been reported and named. These are HLA-T previously known as HLA-16 (19), HLA-U previously known as HLA-21 (19), HLA-V previously known as HLA-75 (19), HLA-W previously known as HLA-80 (19), HLA-P previously known as HLA-90 (19) and HLA-Y previously known as HLA-BEL/COQ/DEL (20, 21). A full list of all recognised HLA genes is given in Table 1.

**Table 1:** Names for genes in the HLA region

Name^a^	Previous equivalents	Molecular characteristics
*HLA-A*	—	Class I α-chain
*HLA-B*	—	Class I α-chain
*HLA-C*	—	Class I α-chain
*HLA-E*	E, ‘6.2’	Associated with class I 6.2-kB Hind III fragment
*HLA-F*	F, ‘5.4’	Associated with class I 5.4-kB Hind III fragment
*HLA-G*	G, ‘6.0’	Associated with class I 6.0-kB Hind III fragment
*HLA-H*	H, AR, ‘12.4’, HLA-54	Class I pseudogene associated with 5.4-kB Hind III fragment
*HLA-J*	cda12, HLA-59	Class I pseudogene associated with 5.9-kB Hind III fragment
*HLA-K*	HLA-70	Class I pseudogene associated with 7.0-kB Hind III fragment
*HLA-L*	HLA-92	Class I pseudogene associated with 9.2-kB Hind III fragment
*HLA-N*	HLA-30	Class I gene fragment associated with a 1.7kb Hind III fragment
***HLA-P***	HLA-90	Class I gene fragment associated with 9.0-kB Hind III fragment
*HLA-S*	HLA-17	Class I gene fragment associated with a 3.0kb Hind III fragment
***HLA-T***	HLA-16	Class I gene fragment associated with 16.0-kB Hind III fragment
***HLA-U***	HLA-21	Class I gene fragment associated with 2.1-kB Hind III fragment
***HLA-V***	HLA-75	Class I gene fragment associated with 7.5-kB Hind III fragment
***HLA-W***	HLA-80	Class I gene fragment associated with 8.0-kB Hind III fragment
*HLA-X*	HLA-X	Class I gene fragment
***HLA-Y***	HLA-BEL/COQ/DEL	Class I gene fragment
*HLA-Z*	HLA-Z1	Class I gene fragment located within the HLA Class II region
*HLA-DRA*	DRα	DR α chain
*HLA-DRB1*	DRβI, DR1B	DR β1 chain determining specificities DR1, DR2, DR3, DR4, DR5 etc
*HLA-DRB2*	DRβII	Pseudogene with DR β-like sequences
*HLA-DRB3*	DRβIII, DR3B	DR β3 chain determining DR52 and Dw24, Dw25, Dw26 specificities
*HLA-DRB4*	DRβIV, DR4B	DR β4 chain determining DR53
*HLA-DRB5*	DRβIII	DR β5 chain determining DR51
*HLA-DRB6*	DRBX, DRBσ	DRB pseudogene found on DR1, DR2 and DR10 haplotypes
*HLA-DRB7*	DRBψ1	DRB pseudogene found on DR4, DR7 and DR9 haplotypes
*HLA-DRB8*	DRBψ2	DRB pseudogene found on DR4, DR7 and DR9 haplotypes
*HLA-DRB9*	M4.2 βexon	DRB pseudogene, isolated fragment
*HLA-DQA1*	DQα1, DQ1A	DQ α chain
*HLA-DQB1*	DQβ1, DQ1B	DQ β chain
*HLA-DQA2*	DXα, DQ2A	DQ α-chain-related sequence, not known to be expressed
*HLA-DQB2*	DXβ, DQ2B	DQ β-chain-related sequence, not known to be expressed
*HLA-DQB3*	DVβ, DQB3	DQ β-chain-related sequence, not known to be expressed
*HLA-DOA*	DNA, DZα, DOα	DO α chain
*HLA-DOB*	DOβ	DO β chain
*HLA-DMA*	RING6	DM α chain
*HLA-DMB*	RING7	DM β chain
*HLA-DPA1*	DPα1, DP1A	DP α chain
*HLA-DPB1*	DPβ1, DP1B	DP β chain
*HLA-DPA2*	DPα2, DP2A	DP α-chain-related pseudogene
*HLA-DPA3*	DPA3	DP α-chain-related pseudogene
*HLA-DPB2*	DPβ2, DP2B	DP β-chain-related pseudogene
*TAP1*	ABCB2, RING4, Y3, PSF1	ABC (ATP Binding Cassette) transporter
*TAP2*	ABCB3, RING11, Y1, PSF2	ABC (ATP Binding Cassette) transporter
*PSMB9*	LMP2, RING12	Proteasome-related sequence
*PSMB8*	LMP7, RING10	Proteasome-related sequence
*MICA*	MICA, PERB11.1	Class I chain-related gene
*MICB*	MICB, PERB11.2	Class I chain-related gene
*MICC*	MICC, PERB11.3	Class I chain-related pseudogene
*MICD*	MICD, PERB11.4	Class I chain-related pseudogene
*MICE*	MICE, PERB11.5	Class I chain-related pseudogene

a Gene names given in bold type have been assigned since the 2004 Nomenclature report.

a. Conditions for acceptance of new allele sequences

As emphasised in previous reports, there are required conditions for acceptance of new sequences for official names.

1. Where a sequence is obtained from cDNA, or where PCR products are subcloned prior to sequencing, several clones should have been sequenced.
2. Sequencing should always be performed in both directions.
3. If direct sequencing of PCR amplified material is performed, products from at least two separate PCR reactions must have been sequenced.
4. In individuals who are heterozygous for a locus, and where one of the alleles is novel, the novel allele must be sequenced in isolation from the second allele. Thus an allele sequence that is derived using a sequence-based typing (SBT) methodology, where both alleles of a heterozygous individual are sequenced together, is insufficient evidence for assignment of an official designation.
5. Sequence derived solely from the primers used to amplify an allele must not be included in the submitted sequence.
6. Where possible, a novel sequence should be confirmed by typing of genomic DNA using a method such as PCR-SSOP or PCR-SSP. Where a new sequence contains either a novel mutation or a previously unseen combination of nucleotides (sequence motif), this must be confirmed by a DNA typing technique. This may require the use of newly designed probes or primers to cover the new mutation; these reagents should also be described.
7. An accession number in a databank should have been obtained. Sequences may be submitted to the databases online at the following addresses:
  EMBL: www.ebi.ac.uk/Submissions/index.html
  GenBank: www.ncbi.nlm.nih.gov/Genbank/submit.html
  DDBJ: www.ddbj.nig.ac.jp/sub-e.html
8. Full-length sequences are preferable though not essential; the minimum requirements are complete exons 2 and 3 for an HLA class I sequence and complete exon 2 for an HLA class II sequence.
9. Where a novel sequence differs only within an intron or other non-coding part of the gene, a full-length sequence must be obtained, which covers all coding and non-coding regions. In the absence of a full-length genomic sequence from the most closely related allele that is identical in its exon sequence, it may be required that this also be sequenced and submitted before a name can be assigned to the novel sequence.
10. Where possible, a paper in which the new sequence is described should be submitted for publication. Copies of draft publications can be submitted to the database by email or FAX.
11. Sequences derived solely from tumour material will not be considered for nomenclature.
12. The complete HLA type for the *HLA-A*, -*B* and *-DRB1* genes should be submitted for the material in which a novel allele has been defined. In addition the sample should have been characterised for the second allele at the locus of interest in a heterozygous individual.
13. DNA or other material, preferably cell lines, should, wherever possible, be made available in a publicly accessible repository or alternatively, at least in the originating laboratory. The WHO Nomenclature Committee will maintain documentation on this material.
14. Submission of a sequence to the WHO Nomenclature Committee should be performed using the online submission tool available at www.ebi.ac.uk/imgt/hla/subs/submit.html. Researchers are expected to complete a questionnaire relating to the sequence and provide a comparison of their new sequence with known related alleles. If the sequence cannot be submitted using the online web tools, researchers should contact hla@alleles.org directly for details of alternative submission methods.

Although at present it is only a recommendation that full-length sequences of the coding region of novel alleles be submitted it was widely felt that in the future this should become a requirement for submission. Such requirement would remove many of the currently encountered ambiguities in the assignment of names to alleles for which partial sequences have been submitted and should not be burdensome as sequencing techniques have improved substantially since the submission conditions were first devised. In cases where novel mutations or polymorphisms are detected in non-coding regions of the gene, it will be a requirement that full-length sequences be submitted of both the novel allele and its most closely related allele.

It should be noted with some caution that cells from which only partial sequences have been obtained may later be shown to have different or novel alleles when further sequencing is performed. This is of particular importance in cases where partial sequences of what appears to be the same allele have been obtained from several different cells. In such cases, all cells studied have been listed in this report.

Current practice is that official designations will be promptly assigned to newly described alleles in periods between Nomenclature Committee meetings, provided that the submitted data and its accompanying description meet the criteria outlined above. A list of the newly reported alleles is published each month in nomenclature updates in the journals Tissue Antigens, Human Immunology and the International Journal of Immunogenetics. The listing of references to new sequences does not imply priority of publication. The use of numbers or names for alleles, genes or specificities which pre-empt assignment of official designations by the Nomenclature Committee is strongly discouraged.

The list of those genes in the HLA region considered by the WHO Nomenclature Committee is given in Table 1.

b. New Allele Sequences

A total of 2558 HLA alleles have been named since the last report (18). The newly named alleles are shown in bold typeface in Tables 2 to 11. For HLA class I, 616 *HLA-A*, 913 *HLA-B*, 446 *HLA-C*, four *HLA-E*, 19 *HLA-F*, 31 *HLA-G*, 12 *HLA-H*, nine *HLA-J*, six *HLA-K*, five *HLA-L*, four *HLA-P* and three *HLA-V* alleles were named, making a total of 3249 class I alleles with official names. For HLA class II, 368 *HLA-DRB1*, 12 *HLA-DRB3*, one *HLA-DRB4*, one *HLA-DRB5*, seven *HLA-DQA1*, 45 *HLA-DQB1*, six *HLA-DPA1*, 22 *HLA-DPB1*, one *HLA-DMB* and four *HLA-DOA* alleles were named, making a total of 1198 class II alleles with official names. Eleven *MICA* alleles were named bringing their total to 68 and 12 *MICB* alleles bringing their total to 30 alleles, see Table 12. The total number of alleles at each locus assigned with official names as of 31^st^ December 2009 is given in Table 13. A full list of all allele names that have been deleted is given in Table 14.

**Table 3:** Designations of HLA-B alleles

HLA allele^a^	Pre 2010 designation	HLA specificity	Previous equivalents	Individual or cell line from which the sequence was derived	Accession number	References or submitting author(s)
B*07:02:01	B*070201	B7	B7.2, B*07L	JY, PP, RD105U, RD105, L5, L7, GN00105, 383008, PGF, PAT541	M16102, M32317, P01889, U29057, L47338, U49904, U49905, AJ292075, AJ309047, AL671277, DQ249181	(29)^b^, (141)^b^
B*07:02:02	B*070202	B7	B*0702V, B*07AD	HGW12327, DZA10	Y13567, AJ002675	
B*07:02:03	B*070203	B7	B*07N	RN1373B	AF002273, AF017314	
B*07:02:04	B*070204	B7	—	GN00433	AY296125, AY296126	
B*07:02:05	B*070205	B7	—	D22669	AM493903	O Avinens
B*07:02:06	B*070206	B7	—	LUMC-B1	AM746337	(64)
B*07:02:07	B*070207	B7	—	MHHAKB-490085, HN-69654-1, HN-21410-5, HN-51978-7, HN-34589-1, HN-69548-0, HN-65317-7, HN-48912-4	AM778679, FJ594498, FJ594577, FJ594579, FJ594497, FJ594599, FJ853762, FJ868485	R Blasczyk, Histogenetics^b^
B*07:02:08	B*070208	B7	—	HN-24405-0, HN-76695-6, HN-999114, HN-46788-1, HN-12328-3	FJ549405, FJ853793, FJ392162, GQ449646, GQ859541	Histogenetics
B*07:02:09	B*070209	B7	—	H09020	FJ869339	(142)
B*07:02:10	B*070210	B7	—	HN-62104-5, HN-53909-8, HN-87721-5, HN-28858-1, HN-97825-6	FJ234987, FJ234999, FJ346233, FJ346326, GQ994063	Histogenetics
B*07:02:11	B*070211	B7	—	HN-03271-4	FJ235022	Histogenetics
B*07:02:12	B*070212	B7	—	HN-B-174638	FJ235055	Histogenetics
B*07:02:13	B*070213	B7	—	HN-64115-8	FJ594716	Histogenetics
B*07:02:14	B*070214	B7	—	91550018, HN-59808-2	GQ375767, FJ765977	D Fuerst, Histogenetics^b^
B*07:02:15	B*070215	B7	—	HN-68819-4	FJ346337	Histogenetics
B*07:02:16	B*070216	B7	—	HN-34150-5	FJ392175	Histogenetics
B*07:02:17	B*070217	B7	—	HN-9444-3	FJ853800	Histogenetics
B*07:02:18	B*070218	B7	—	HN-6858794	FJ866148	Histogenetics
B*07:02:19	B*070219	B7	—	JMDP01K046	AB512680	K Tadokoro
B*07:03	B*0703	B703	BPOT	POT71, BPot, 1156492+	X64454, U21053, AM117564	J Rowlands^b^
B*07:04	B*0704	B7	B7E	10243, CB1008/02-PR-01408	U04245, AJ564010, AJ564011	
B*07:05:01	B*070501	B7	B*07ZEL	GEE018, ZEL, CF, SZ-77	L33922, U18661, U21052, GQ161942	HY Zou^b^
B*07:05:02	B*070502	B7	B*07MVE0605	Z-0015026	AM050156	(143)
B*07:05:03	B*070503	B7	—	56	DQ162803	(144)
B*07:06	B*0706	B7	B7-L79	L7901	X91749	
B*07:07	B*0707	B7	—	DAPO, C211	Z70315, AJ556171	
B*07:08	B*0708	B7^c^	—	A.McG	X99735	
B*07:09	B*0709	B7	B*07ML, B*07DKDC	TER#939, DKDC, 011147550	AJ003063, AF106043, AF106044, AF106045, AF132018, AF132019, AF132020	
B*07:10	B*0710	B7^c^	B*07AE	A.E., 0200201604Haplo	AJ223602, AM236752	S Ulrich^b^
B*07:11	B*0711	B7	B-0702v	001524990	AF056481, AF056482	
B*07:12	B*0712	B7^c^	—	GN00216, GN00232	AF061865, AF061866, AF072443, AF072444	
B*07:13	B*0713	—	—	346-808, NT01090	AF065646, AF065647, GQ251352	CK Hurley^b^
B*07:14	B*0714	B7^c^	B*0707Var	012774733, NM4B169, GN00330	AF127806, AF127807, AF132491, AF165854, AF165855, AF205532, AF205533	
B*07:15	B*0715	B7	B*07021Var	NM4B274, 4344PL	AF148809, AF148810, AJ243371, AJ243372	
B*07:16	B*0716	B7	B*0703Variant	CT-VC, NT01102	AJ237594, AJ237595, GQ251363	CK Hurley^b^
B*07:17	B*0717	B7	—	R99171035G, TBC59473	AF173936, AB256953	M Satake^b^
B*07:18:01	B*071801	—	—	CL183	AF189017	
B*07:18:02	B*071802	—	—	DNA78151, HN-3157224	AM778128, FJ594549	J Enczmann, Histogenetics^b^
B*07:19	B*0719	—	B*0704V	GN00323, GN00335	AF198648, AF198649, AF226689, AF226690	
B*07:20	B*0720	—	B*0702V, B*07MSB	CU26, SMB7N, MHH-000773	AJ251770, AJ251771, AF244146, AF244147, AJ278043, AJ278044	
B*07:21	B*0721	—	B*07021new	NM5b91	AF255714, AF255715	
B*07:22:01	B*072201	—	B*07021variant	10009909	AJ400823	
B*07:22:02	B*072202	—	—	BY00278, HN-6966060	EU522472, FJ600623	(36), Histogenetics^b^
B*07:23	B*0723	—	B*07021V	GN00368	AF279113, AF279114	
B*07:24	B*0724	B7	B*07021var	BEL-LEI, BY00293	AJ401222, EU555324	CK Hurley^b^
B*07:25	B*0725	—	B*CBU138	CBU138	AF313415, AF313416	
B*07:26	B*0726	B7	B*07BJ	BSF, 14622	AF317496, AF317497, AJ311257	
B*07:27	B*0727	—	B*KHOLM	KHOLM	AF343000, AF343001	
B*07:28	B*0728	—	B*ALTHO	ALTHO	AF402322, AF402323	
B*07:29	B*0729	—	—	BY0029	AF443285, AF443286	
B*07:30	B*0730	B7	—	D25857	AB073300, AB073668	
B*07:31	B*0731	—	B7x42	VTIS87843	AY124570, AY124571	
B*07:32	B*0732	—	—	78844, 78843, 917/03	AJ550636, AJ550637, AJ550638, AY675510	
B*07:33	B*0733	—	—	10143386, 34303	AJ549199, AJ937776	T Gervais^b^
B*07:34	B*0734	—	—	121036	AJ558103	
B*07:35	B*0735	—	—	A3609	AY330327, AY330328	
B*07:36	B*0736	—	—	03Tp2186, UAS12342, 4040	AJ581083, AJ581084, AM231297	M Bengtsson^b^
B*07:37	B*0737	—	—	21149/02, NT00713	AY390377, AY390378, EF375693	CK Hurley^b^
B*07:38	B*0738	—	—	BY00054, BY00439	AY562130, AY562131, FJ688153	CK Hurley^b^
B*07:39	B*0739	—	—	MHH0402736	AJ870971	(145)
B*07:40	B*0740	B7	—	NJ6548	AY758393, AY758394, AY758395	(146)
B*07:41	B*0741	—	—	CTM8683455	AY971144	JL Vicario
B*07:42	B*0742	—	—	NT00571, 4229311	DQ007037, DQ007038, AM931289	(147), L Hammond^b^
B*07:43	B*0743	—	—	2005050305, BY00435	DQ088145, DQ088146, FJ688157	(148), CK Hurley^b^
B*07:44	B*0744	—	—	CTM9748540	DQ196429	JL Vicario
B*07:45	B*0745	—	—	NT00623	DQ270208, DQ270208	(149)
B*07:46	B*0746	B7	—	UA-225-TP3611	AM182459	M Bengtsson
B*07:47	B*0747	—	—	151235, HN-90158-1, HN-55850-5, HN-53392-9, HN-52240-1	DQ343758, FJ594589, FJ868470, FJ868472, FJ868489	K Hirv, Histogenetics^b^
B*07:48	B*0748	—	—	BY00085, BY00131, NT00700, MHHAKB-175281, HN-174-4	DQ455017, DQ924381, EF195111, AM493309, FJ594592	(77), CK Hurley^b^, R Blasczyk^b^, Histogenetics^b^
B*07:49N	B*0749N	Null	—	WLODslaw	AM236599	(150)
B*07:50	B*0750	—	—	FFM1840AN	AM236590	AM Little
B*07:51	B*0751	—	—	VTIS146548, BY00421	EF088202, FJ669609	BD Tait, CK Hurley^b^
B*07:52	B*0752	—	—	NT00698	EF195109	(85)
B*07:53	B*0753	—	—	NT00714, NT00743, NT00977	EF375692, EU185514, EU716061	(85), CK Hurley^b^
B*07:54	B*0754	—	—	NT00718, HN-01873-0	EF422078, FJ868468	(85), Histogenetics^b^
B*07:55	B*0755	—	—	BJ031	EF472969	Z Zhang
B*07:56	B*0756	—	—	94858	EF517947	MS Leffell
B*07:57	B*0757	B7	—	DJEGen, HN-09475-9	AM689936, FJ594600	A Dormoy, Histogenetics^b^
B*07:58	B*0758	B7	—	99415CB	AB306895	H Inoko
B*07:59	B*0759	—	—	LUMC-B2	AM746338	(64)
B*07:60	B*0760	—	—	BJ034480	EU131141	X Shan
B*07:61	B*0761	—	—	LUMC-B34	AM904554	(64)
B*07:62	B*0762	—	—	MHHAKB-519696, HN-43686-6, HN39376-1, HN-84760-8, HN-01994-1, HN-31099-4	AM906167, FJ594562, FJ594593, FJ594553, FJ853759, FJ853760	R Blasczyk, Histogenetics^b^
B*07:63	B*0763	—	—	263776, HN-13211-7, HN-21642-3, HN-05697-8, HN-16465-0, HN-6008615	EU410615, FJ594502, FJ594503, FJ594588, FJ600617, FJ594617	J Mytilineos, Histogenetics^b^
B*07:64	B*0764	—	—	BY00282, BY00403	EU522468, FJ619492	(36), CK Hurley^b^
B*07:65	B*0765	—	—	LUMC-B47	AM946388	(64)
B*07:66	B*0766	—	—	JMDP01K013	AB435170	K Tadokoro
B*07:67N	B*0767N	Null	—	MHHI-602067	AM920503	P Horn
B*07:68	B*0768	—	—	DKM918035, HN-64013-5, HN-00794-6, HN-61579-6	EU931583, FJ346242, FJ594697, FJ765970	A Vigh, Histogenetics^b^
B*07:69	B*0769	—	—	IME725-08T, IME726-08T, IME728-08T	FJ603314	(151)
B*07:70	B*0770	B7	—	ALZ6703	EU184011	D Thomas
B*07:71	B*0771	—	—	BY00395, HN-7668309	FJ619499, FJ765770	CK Hurley, Histogenetics^b^
B*07:72	B*0772	—	—	BY00399, HN-7715052	FJ619477, FJ765552	CK Hurley, Histogenetics^b^
B*07:73	B*0773	—	—	HN-13623-5, HN-13757-1, HN-52511-6, HN-80443-9	FJ235032, FJ235033, FJ346302, FJ853796	Histogenetics
B*07:74	B*0774	—	—	HN-13304-0, HN-69732-0, HN-21605-0, HN1687-2, HN-83039-6	FJ346261, FJ235059, FJ765542, FJ853795, FJ765954	Histogenetics
B*07:75	B*0775	—	—	09d00312	FM995501	JDH Anholts
B*07:76	B*0776	—	—	NT01023, HN-692448	FJ797379, FJ468325	CK Hurley, Histogenetics^b^
B*07:77	B*0777	—	—	60638317, HN-57433-7	FJ821318, GQ401200	(152), Histogenetics^b^
B*07:78	B*0778	—	—	HN-4811	FJ234986	Histogenetics
B*07:79	B*0779	—	—	HN-53200-1	FJ235002	Histogenetics
B*07:80	B*0780	—	—	HN-4604976, HN-24661-7	FJ235012, GQ914797	Histogenetics
B*07:81	B*0781	—	—	HN-4287194	FJ235017	Histogenetics
B*07:82	B*0782	—	—	HN-6387-3	FJ235037	Histogenetics
B*07:83	B*0783	—	—	HN-70201-1	FJ235050	Histogenetics
B*07:84	B*0784	—	—	HN-46165-8	FJ346304	Histogenetics
B*07:85	B*0785	—	—	HN-19997-8	FJ765555	Histogenetics
B*07:86	B*0786	—	—	HN-89470-0	FJ765859	Histogenetics
B*07:87	B*0787	—	—	HN-05165-4, HN-51115-3, HN-48869-4, HN-92184-1, HN-78438-5, HN-97350-0, HN-30108-0, HN-84250-6, HN-30107-2, HN-20795-6	FJ346252, FJ346253, FJ346316, FJ494827, FJ875564, FJ969942, GQ245737, GQ254338, GQ345063, GQ994071	Histogenetics
B*07:88	B*0788	—	—	HN-50073-6, HN-89144-0, HN-894858, HN-73515-5	FJ346259, FJ346276, FJ346289, FJ875573	Histogenetics
B*07:89	B*0789	—	—	HN-18662-7	FJ346282	Histogenetics
B*07:90	B*0790	—	—	HN-39120-2	FJ346305	Histogenetics
B*07:91	B*0791	—	—	Xian07	GQ231485	M Liu
B*07:92	B*0792	B7	—	HN-36978-7, HSR146198	FJ346310, FN568353	Histogenetics, H Tran^b^
B*07:93	B*0793	—	—	HN-1126213	FJ392164	Histogenetics
B*07:94	B*0794	—	—	HN-5591438	FJ765771	Histogenetics
B*07:95	B*0795	—	—	HN-08589-3, HN-86817-4	FJ853790, FJ392182	Histogenetics
B*07:96	B*0796	—	—	HN-27061-3	FJ853803	Histogenetics
B*07:97	B*0797	—	—	HN-8027505	FJ866142	Histogenetics
B*07:98	B*0798	—	—	HN-45887-8, HN-44247-2, HN32171-8, HN-64356-6	FJ866157, FJ640579, FJ765927, FJ875662	Histogenetics
B*07:99	B*0799	—	—	HN-54406-6	FJ866165	Histogenetics
B*08:01:01	B*080101	B8	—	LCL721, MF, CGM1, HECO, 12506397, COX, PAT135, PAT144	M59841, M24036, M28204, L76093, AJ295294, AL669854, DQ249172, DQ249174	(29)^b^, (141)^b^
B*08:01:02	B*080102	B8	—	MHHZ-00015060, HN-17110-2, HN-51327-9, HN-38925-2, HN68691-9, HN-22943-9, HN-8204060	AJ971432, FJ594609, FJ594614, FJ594730, FJ868475, FJ868482, FJ594612	R Blasczyk, Histogenetics^b^
B*08:01:03	B*080103	B8	—	CambsB8	AM158301	R Goodman
B*08:01:04	B*080104	B8	—	COLO829, MHHZ-00026812, HN-87341-8, HN-21895-9, HN-145906, HN-14718-3	AY623605, AM493310, FJ594558, FJ594581, FJ594596, FJ853768	S Adams, (153), Histogenetics^b^
B*08:01:05	B*080105	B8	—	106998, HN-25941-9	AM850143, FJ853758	J Enczmann, Histogenetics^b^
B*08:01:06	B*080106	B8	—	70685	FM179947	M Danzer
B*08:01:07	B*080107	B8	—	HN-1269-9, HN-85124-4	FJ235027, GQ254351	Histogenetics
B*08:01:08	B*080108	B8	—	HN-00172-4, HN-69625-2, HN-82902-4, HN-82787-0, HN-12663-3, HN-60847-6, P-664367	FJ538279, FJ853792, FJ875561, GQ240393, GQ449633, GQ994057, FN430731	Histogenetics, R Blasczyk^b^
B*08:01:09	B*080109	B8	—	HN-5635052	FJ765788	Histogenetics
B*08:01:10	B*080110	B8	—	HN-0957535, HN-0957550	FJ765780, FJ765781	Histogenetics
B*08:02	B*0802	B8	B8JON, B8V	20015, 19315	U04244	
B*08:03	B*0803	B8	B*08NR	NR	U28759	
B*08:04	B*0804	—	B*08New-UW	BLB, JS, PF	U67330, U67331, U74386	
B*08:05	B*0805	—	rn083B	rn083B	U88254, AF017315	
B*08:06	B*0806	B8	B-08v	009048430	AF056483, AF056484	
B*08:07	B*0807	B8	B*NV	BM1 101910	AF105226	
B*08:08N	B*0808N	Null	B8Null	STRIJOHN, RS	Y18552	
B*08:09	B*0809	B8	B*08HO, B*MW	H.O., MW-ARCBS, HM-ARCBS, GN00244, GN00287, ANO	AJ131852, AJ131853, AF117768, AF117769, AF127247, AF127248, AF102559, AF102560, AF176073, AF176074, AJ276994, AJ607425	
B*08:10	B*0810	B8	B*0801Var	R.E	AJ133101, AJ133102	
B*08:11	B*0811	—	—	NMDP -ID#035343375	AF213681, AF213682	
B*08:12:01	B*081201	—	B*0801V	GN00344, G3543, GN00371	AF226150, AF226151, AJ276427, AF279674, AF279675	
B*08:12:02	B*081202	—	—	HN-51339-9	FJ235008	Histogenetics
B*08:12:03	B*081203	—	—	HN-83595-3, HN-98041-3	FJ346331, GQ240392	Histogenetics
B*08:13	B*0813	—	—	2000-21-622-7, NT01097	AF310144, AF310145, GQ251359	CK Hurley^b^
B*08:14	B*0814	—	—	GN00386	AY016211, AY016212	
B*08:15	B*0815	B8	—	VTIS37741	AY057398, AY057399	
B*08:16	B*0816	B8	—	026575043, FH1127	AF468046, AF468047, AY832855, AY832856	
B*08:17	B*0817	—	—	C.E.-120339	AJ508056, AJ508057, AJ508058	
B*08:18	B*0818	B8	—	VTIS85342	AY210415, AY210416	
B*08:19N	B*0819N	Null	B*08SM	11829109	AJ532609	
B*08:20	B*0820	—	—	10150742	AJ549202	
B*08:21	B*0821	—	—	GN00434	AY296127, AY296128	
B*08:22	B*0822	—	—	162273	AJ634744	
B*08:23	B*0823	—	—	MHH-0305726, HN-34496-4, NT01099	AJ878870, FJ594611, GQ251383	R Blasczyk, Histogenetics^b^, CK Hurley^b^
B*08:24	B*0824	—	B*8MVE0904	MHHZ-00006379, HN-74321-5	AJ880423, FJ594604	R Blasczyk, Histogenetics^b^
B*08:25	B*0825	—	—	MHHZ-00013188	AM039492	R Blasczyk
B*08:26	B*0826	—	—	NT00570	DQ120784, DQ120785	(149)
B*08:27	B*0827	—	—	N2917	DQ320644, DQ320645	LA Baxter-Lowe
B*08:28	B*0828	—	—	90082725, HN-90068-2, HN-42792-4, HN-34419-4, HN-37806-1	AM072963, FJ853767, FJ868467, FJ868469, FJ868476	(154), Histogenetics^b^
B*08:29	B*0829	B8	—	CTM-4096286	DQ417689	(155)
B*08:30N	B*0830N	Null	—	264040	DQ641496	K Hirv
B*08:31	B*0831	—	—	BY00141	EF173482	(149)
B*08:32	B*0832	—	—	1817140	EF156418	D Smillie
B*08:33	B*0833	—	—	ADBA10260AN	AM492196	AM Little
B*08:34	B*0834	B8	—	99239	AM778129	J Enczmann
B*08:35	B*0835	—	—	NT00750, 461129019, HN-50855-7	EU256488, AM747237, FJ594563	(85), V Dubois^b^, Histogenetics^b^
B*08:36	B*0836	—	—	BY00283	EU522467	(36)
B*08:37	B*0837	—	—	70878, HN-58346-4, HN-77049-8, HN-8350091, HN-54062-1	FM179948, FJ235042, FJ346287, FJ866170, FJ952587	M Danzer, Histogenetics^b^
B*08:38	B*0838	B8	—	49595	FM177891	(156)
B*08:39	B*0839	—	—	HN-05818-0, HN-4582414, HN-93071-6	FJ346271, FJ765570, FJ952586	Histogenetics
B*08:40	B*0840	—	—	BY00430, HN-11509-7	FJ688162, FJ765812	CK Hurley, Histogenetics^b^
B*08:41	B*0841	—	—	HN-49468-2, BY00481, HN-98338-7, HN-97818-9	FJ234989, GQ410100, GQ199710, GQ254343	Histogenetics, CK Hurley^b^
B*08:42	B*0842	—	—	HN-14418-1	FJ235041	Histogenetics
B*08:43	B*0843	—	—	HN-75612-1	FJ765863	Histogenetics
B*08:44	B*0844	—	—	HN-57023-6	FJ346334	Histogenetics
B*08:45	B*0845	—	—	HN-37008-9	FJ853781	Histogenetics
B*08:46	B*0846	—	—	HN-84979-5	FJ853794	Histogenetics
B*08:47	B*0847	—	—	HN-45333-4	FJ853802	Histogenetics
B*08:48	B*0848	—	—	HN-77831-6	FJ640581	Histogenetics
B*08:49	B*0849	—	—	HN-6751512	FJ765784	Histogenetics
B*08:50	B*0850	—	—	HN-6520188	FJ765768	Histogenetics
B*08:51	B*0851	—	—	HN-21578-0	FJ765813	Histogenetics
B*08:52	B*0852	—	—	HN-75383-4	FJ765842	Histogenetics
B*08:53	B*0853	—	—	HN-31452-7	FJ765854	Histogenetics
B*08:54	B*0854	—	—	HN-4859149	FJ765868	Histogenetics
B*13:01:01	B*130101	B13	B13.1	HE, SDI, YTY, TAC, SZ-74	M24075, D50290, FJ973631	(157)^b^
B*13:01:02	B*130102	B13	—	09006203	FJ868754	A Vigh
B*13:01:03	B*130103	B13	—	K34517	GU136390	M Lin
B*13:02:01	B*130201	B13	B13.2, B13N	LBF, TO, HJB, PKM, TAC, L7901, 1510200306	M19757, M24041, D50291, AJ295278, AF196182	
B*13:02:02	B*130202	B13	—	NT00574, NT00629, NT00773	DQ007041, DQ007042, DQ334731, DQ334732, EU375812	(147), CK Hurley^b^
B*13:02:03	B*130203	B13	—	MIPO321144AN	AM000052	AM Little
B*13:02:04	B*130204	B13	—	NT00706, MST987659354	EF375690, EU086373	(85), R Kelsch^b^
B*13:02:05	B*130205	B13	—	p3568	FJ596198	(142)
B*13:02:06	B*130206	B13	—	HN-70777-7	FJ346234	Histogenetics
B*13:03	B*1303	—	B New	CTM4956865, CTM2956866	U14943	
B*13:04	B*1304	—	B*15X21	TER847, 27B, 76002	U75533, U88248, AF017316, Y12378, Y12379	
B*13:06	B*1306	—	B*1301V	GN00336, BY00457	AF226691, AF226692, FJ346234	CK Hurley^b^
B*13:07N	B*1307N	Null	B1301V1	JCB13747	AB032598	
B*13:08Q	B*1308Q	—	—	PACO, LUMC-B45	AJ295279, AM934702	JDH Anholts^b^
B*13:09	B*1309	—	—	2000-112-197	AY034808, AY034809	
B*13:10	B*1310	—	—	2001-7709, BY00404	AF461046, AF461047, FJ619484	CK Hurley^b^
B*13:11	B*1311	—	—	119555, BY00405	AJ496551, AJ496552, AJ496553, FJ619491	CK Hurley^b^
B*13:12	B*1312	—	—	GN00416, Scu3556m	AY428806, AY428807, EU516353	FdP Sanchez Gordo^b^
B*13:13	B*1313	B13	—	CHD1144	AY505490	
B*13:14	B*1314	—	—	118591	AM086443	(158)
B*13:15	B*1315	—	—	FRBA36844AN	AM114035	AM Little
B*13:16	B*1316	—	—	BY00087	DQ455015	(57)
B*13:17	B*1317	—	—	BY00124	DQ832588	CK Hurley
B*13:18	B*1318	—	—	BJ030	EF472968	Z Zhang
B*13:19	B*1319	—	—	BJ034	EF555208	Z Zhang
B*13:20	B*1320	—	—	BJ037013	EF195123	X Shan
B*13:21	B*1321	—	—	SCCAB2	EF539269	A Smith
B*13:22:01	B*132201	—	—	BY00309, HN-s5746869	EU643607, FJ765805	(36), Histogenetics^b^
B*13:22:02	B*132202	—	—	SZ-18	FJ561483	(159)
B*13:23	B*1323	—	—	BJ067050	EU884291	X Shan
B*13:25	B*1325	—	—	SZ-31	GQ141863	(157)
B*13:26	B*1326	—	—	54600326	FJ898283	C Zhu
B*13:27	B*1327	—	—	HN-73120-1	FJ346300	Histogenetics
B*13:28	B*1328	—	—	Xian13	GQ231484	S Ye
B*13:29	B*1329	—	—	HN-6362029	FJ392169	Histogenetics
B*13:30	B*1330	—	—	HN-04449-4	FJ502325	Histogenetics
B*13:31	B*1331	—	—	HN-5794422	FJ560457	Histogenetics
B*13:32	B*1332	—	—	HN-7628141, BY00533	FJ765550, GU066745	Histogenetics, CK Hurley
B*13:33	B*1333	—	—	HN-3340275	FJ765775	Histogenetics
B*13:34	B*1334	—	—	HN-0300931018822	FJ858892	Histogenetics
B*13:35	B*1335	—	—	HN-94049-4	FJ875563	Histogenetics
B*13:36	B*1336	—	—	JMDP36K066	AB536746	K Tadokoro
B*14:01	B*1401	B64(14)	—	MRWC, 32367, W6106, WT51, SZ-91	M24040, X94574, FJ986193	HY Zou^b^
B*14:02:01	B*140201	B65(14)	—	BB, CGM1, CM1402, 10038822, PAT135, PAT218	M59840, M24032, U90558, AJ301657, DQ249173, DQ249176	(141)^b^
B*14:02:02	B*140202	B65(14)	—	86743, HN-2931859	AY957460, FJ594622	MS Leffell, Histogenetics^b^
B*14:02:03	B*140203	B65(14)	—	HN-84155-1	FJ346284	Histogenetics
B*14:02:04	B*140204	B65(14)	—	HN-3547	FJ765538	Histogenetics
B*14:02:05	B*140205	B65(14)	—	BY00531	GU066743	CK Hurley
B*14:03	B*1403	B14^c^	B*1402v	DT16, DT3, E210, 26093	U91330, U91331, AF015271, AF015272, AF279664, AJ496177, AJ496178	
B*14:04	B*1404	—	B*14N	RN1429B	AF002275, AF017317	
B*14:05	B*1405	—	—	S18, 012867131	AF031142, AF031143, AF110259, AF110260, AF110261	
B*14:06:01	B*140601	B14	Sofh3713, wk B14	Fli, 30569	AJ131193, AJ131194, AJ544887, AJ544888	
B*14:06:02	B*140602	B14	B*1402 Variant	GN00248, NT01076	AF102567, AF102568, GQ251369	CK Hurley^b^
B*14:07N	B*1407N	Null	—	CTM5683370, HN-08766-8	AY973957, FJ868463	JL Vicario, Histogenetics^b^
B*14:08	B*1408	—	—	BY00286, HN-41827-8, HN-76118-0	EU555330, FJ594561, FJ853763	(36), Histogenetics^b^
B*14:09	B*1409	—	—	BY00313, HN-7102160, HN-6998912	EU643063, FJ235046, FJ765779	(36), Histogenetics^b^
B*14:10	B*1410	—	—	HN-95293-7, 1937-08, HN-00593-1, HN-03676-1	FJ346272, FM995216, FJ594702, FJ502322	Histogenetics, F Emmerich
B*14:11	B*1411	—	—	HN-02890-1	FJ346273	Histogenetics
B*14:12	B*1412	—	—	BY00489, HN-57692-5	GQ410108, FJ976863	CK Hurley, Histogenetics^b^
B*14:13	B*1413	—	—	HN-53717-3	FJ235072	Histogenetics
B*14:14	B*1414	—	—	HN-48742-8	FJ640582	Histogenetics
B*14:15	B*1415	—	—	HN-15517-8	FJ346314	Histogenetics
B*14:16	B*1416	—	—	HN-52577-6	FJ346338	Histogenetics
B*14:17	B*1417	—	—	HN-0727001	FJ765955	Histogenetics
B*14:18	B*1418	—	—	HN-71743-6, HN-03501-0	FJ875560, GQ245747	Histogenetics
B*15:01:01:01	B*15010101	B62(15)	—	MF, HA, BCK, OLGA (OLL)^c^, KT17, PP, FUR, YAG, BA3, BA4, BA5, 141020031, PAT218, CUI23, MCF	M28203, M83193, U03859, D50292, L48400, AJ295140, L79939, L48400, DQ249177, EF203076, CR759828	(141)^b^, (160)^b^, S Beck^b^
B*15:01:01:02N	B*15010102N	Null	BM1947	BEL-13-JA, 29139	Y17110, AM048844	J Rowlands^b^
B*15:01:02	B*150102	B62(15)	B*1501Var1	PUSPAT, BWH56458, NMDP#015329287, NMDP#015329535, NMDP#015329246, NMDP#015329097, NMDP#015329436	Y17063, Y17168, AF053999, AF054000, AF106626, AF106627	
B*15:01:03	B*150103	B62(15)	B*15New	AG-SP	AF109724, AF109725	
B*15:01:04	B*150104	B62(15)	B*15SRE	ET79538	AJ297940, AJ297941	
B*15:01:06	B*150106	B62(15)	—	LUMC-B9, V50340	AM748045	(64)
B*15:01:07	B*150107	B62(15)	—	90484	DQ869010	MS Leffell
B*15:01:08	B*150108	B62(15)	—	MHHZ-00033538	AM931064	R Blasczyk
B*15:01:09	B*150109	B62(15)	—	HN40602-7, HN-26429-0	FJ235049, FJ346246	Histogenetics
B*15:01:10	B*150110	B62(15)	—	HN-97851-0, HN-49704-1, HN-7127082	FJ765546, FJ866156, FJ765557	Histogenetics
B*15:01:11	B*150111	B62(15)	—	NT01069	GQ251347	CK Hurley
B*15:01:12	B*150112	B62(15)	—	HN-6866011	FJ600625	Histogenetics
B*15:01:13	B*150113	B62(15)	—	HN-62698-9	FJ866160	Histogenetics
B*15:01:14	B*150114	B62(15)	—	HN-8715483, HN-8715715	FJ875678, FJ875679	Histogenetics
B*15:01:15	B*150115	B62(15)	—	HN-9592550, HN-86512-7	FJ875680, FJ875552, FJ875680	Histogenetics
B*15:01:16	B*150116	B62(15)	—	HN-708D	FJ796992	Histogenetics
B*15:02:01	B*150201	B75(15)	B15N, B*1502	APA, LW, CAY, DCH4060, DCH4061, DCH3086, 12WDCH018, 12WDCH017, 12WDCH002, 12WDCH003, 12WDCH016	M75138, M83192, D50293, AF014769, AF014770, AF014771, AF014772, AF014773, AF014774, AF014775, AF014776, AF014777, AF014778, AF014779, AF014780, AF014781, AF014782, AF014783, AF014784	
B*15:02:02	B*150202	B75(15)	—	HN-71751-2	FJ489875	Histogenetics
B*15:02:03	B*150203	B75(15)	—	HN-3219446	FJ392165	Histogenetics
B*15:02:04	B*150204	B75(15)	—	HN-675880	FJ468323	Histogenetics
B*15:03:01	B*150301	B72(70)	—	CC, 26931, 31708, NT006891, SZ-50	X61709, EF156370, FJ986195	CK Hurley^b^, HY Zou^b^
B*15:03:02	B*150302	B72(70)	—	HN-4301284, HN-2455892	FJ235016, FJ765794	Histogenetics
B*15:03:03	B*150303	B72(70)	—	HN-16009-3, HN-25061-3, HN-13580-4, HN-89393-3, HN-30081-3, HN-89387-5	FJ235021, FJ235028, FJ549413, GQ149275, GQ199709, GQ245731	Histogenetics
B*15:04	B*1504	B62(15)	Bw62-G	GRC138, KG, GRC187, GRC-150	M84382, AJ292970	
B*15:05:01	B*150501	B62(15)	Bw62.1	VB, T01-1808	M83191, AJ620474, AJ620475	
B*15:05:02	B*150502	B62(15)	—	HN-86737-1, HN-98202-4	FJ875570, GQ245748	Histogenetics
B*15:06	B*1506	B62(15)	Bw62.4	WI, BY00560	M83194, GU138066	CK Hurley^b^
B*15:07	B*1507	B62(15)	Bw62.5	SB, 23664, SZ-97	M83195, AJ496316, AJ496317, FJ986196	HY Zou^b^
B*15:08	B*1508	B75(15)	B62variant, B15.3	KHAGNI, LATIF, DAN723, CB761/02-PR-01365	L11666, AJ563450, AJ563451	
B*15:09	B*1509	B70	B70.1	34863, 32040	L11571, AJ877173	T Gervais^b^
B*15:10:01	B*151001	B71(70)	B70.2	25514, 19014, GU373, GU2092, GU2037, GU5175	L11570, U11262, U11264, U11269	
B*15:10:02	B*151002	B71(70)	—	HN-6971276, HN-45679-7	FJ765804, GQ240387	Histogenetics
B*15:11:01	B*151101	B75(15)	B15variant, B75v	LEE743, AZ195, AZ319, 13703390	L11604, D50294, AM085497	J Rowlands^b^
B*15:03:03	B*150303	B72(70)	—	HN-16009-3, HN-25061-3, HN-13580-4, HN-89393-3, HN-30081-3, HN-89387-5	FJ235021, FJ235028, FJ549413, GQ149275, GQ199709, GQ245731	Histogenetics
B*15:04	B*1504	B62(15)	Bw62-G	GRC138, KG, GRC187, GRC-150	M84382, AJ292970	
B*15:05:01	B*150501	B62(15)	Bw62.1	VB, T01-1808	M83191, AJ620474, AJ620475	
B*15:05:02	B*150502	B62(15)	—	HN-86737-1, HN-98202-4	FJ875570, GQ245748	Histogenetics
B*15:06	B*1506	B62(15)	Bw62.4	WI, BY00560	M83194, GU138066	CK Hurley^b^
B*15:07	B*1507	B62(15)	Bw62.5	SB, 23664, SZ-97	M83195, AJ496316, AJ496317, FJ986196	HY Zou^b^
B*15:08	B*1508	B75(15)	B62variant, B15.3	KHAGNI, LATIF, DAN723, CB761/02-PR-01365	L11666, AJ563450, AJ563451	
B*15:09	B*1509	B70	B70.1	34863, 32040	L11571, AJ877173	T Gervais^b^
B*15:10:01	B*151001	B71(70)	B70.2	25514, 19014, GU373, GU2092, GU2037, GU5175	L11570, U11262, U11264, U11269	
B*15:10:02	B*151002	B71(70)	—	HN-6971276, HN-45679-7	FJ765804, GQ240387	Histogenetics
B*15:11:01	B*151101	B75(15)	B15variant, B75v	LEE743, AZ195, AZ319, 13703390	L11604, D50294, AM085497	J Rowlands^b^
B*15:11:02	B*151102	B75(15)	B1511V1	JCBT2513	AB036051	
B*15:11:03	B*151103	B75(15)	—	BY00132	DQ924379	CK Hurley
B*15:11:04	B*151104	B75(15)	—	09-1237	GQ867275	YM Park
B*15:12	B*1512	B76(15)	B76	THAI742, HA108	L11603, AJ833646	
B*15:13	B*1513	B77(15)	B77	RSA-ND, CAM020, PETCH, 12WDCH009, 12WDCH010, 12WDCH011, 12WDCH028, SZ-51	L15005, D50295, U90424, U90425, U90422, U90423, U90420, U90421, U90418, U90419, FJ986197	HY Zou^b^
B*15:14	B*1514	B76(15)	B76	SS713, NT00785	L19937, EU484044	CK Hurley^b^
B*15:15	B*1515	B62(15)	B62s	MLH727, LDM, 1510200301	L22027, L49343, L49343	
B*15:16	B*1516	B63(15)	B63.1, 8W66	DOP-ND, 21909, 31133, NT00690	L09735, EF156367	CK Hurley^b^
B*15:17:01:01	B*15170101	B63(15)	B63	JAP-NF, PARMG, CU40A	U01848, U35431, AJ300181, EF203077	(160)^b^
B*15:17:01:02	B*15170102	B63(15)	B*1517 var	Terasaki EXT#95	AJ308397	
B*15:17:02	B*151702	B63(15)	B*1517ERN1204	MHHZ-00014019, HN-95179-5, HN-21735-0, HN-08981-7	AJ880424, FJ594731, FJ868478, FJ868483	R Blasczyk, Histogenetics^b^
B*15:18:01	B*151801	B71(70)	B*7901, B “X”-HS, B71	HS, GU2739, GU2760, MSU, ML108, ML108U, SZ-72	U11266, U11268, D50296, U57966, FJ986198	HY Zou^b^
B*15:18:02	B*151802	B71(70)	—	HN-59809-3	FJ346255	Histogenetics
B*15:18:03	B*151803	B71(70)	—	BY00478	GQ410097	CK Hurley
B*15:18:04	B*151804	B71(70)	—	HN-00570	FJ765772	Histogenetics
B*15:19	B*1519	B76(15)	B76	GEE018	U03027	
B*15:20	B*1520	B62(15)	—	OLGA (OLL), KRC110	U06862	
B*15:21	B*1521	B75(15)	B15Ab	BJ, HWY, 14247373, 12WDCH022	L32862, D44500, U32678, U91332, U91333	
B*15:23	B*1523	—	B’NM5’	TK765, 23509	L37881, AJ504398, AJ504399	
B*15:24	B*1524	B62(15)	B*15ZEL, 1501-B4a, B*1501-Bw4	ZEL, SF94-140, HSV48	U16309, L42146, AJ556174	
B*15:25:01	B*152501	B62(15)	B*15AOH, B*1525	WON, M, HM, BY0007, 12WDCH012, 12WDCH023, 12WDCH025, DCH3258, DCH1109, SZ-102	U18660, U50710, U52177, U52178, U91336, U91337, U91334, U91335, AF014785, AF014786, AF014787, AF014788, AF014789, AF014790, FJ986200	HY Zou^b^
B*15:25:02	B*152502	B62(15)	—	HN-5592857, HN-6100148, HN-6100064	FJ234993, FJ392168, FJ875664	Histogenetics
B*15:26N	B*1526N	Null	B-null	K.I.	D49824	
B*15:27:01	B*152701	B62(15)	—	PELE, HA048, SZ-79	L42144, L40182, AJ784257, AJ784258, FJ986203	HY Zou^b^
B*15:27:02	B*152702	B62(15)	—	RA1242	EF622511	M Yu
B*15:27:03	B*152703	B62(15)	—	SZ-13	EU617016	(161)
B*15:28	B*1528	B62(15)^c^	B15v1	YTR	D44499	
B*15:29	B*1529	B15	B15v3	DKA, 463/02-IKEM	D44501, AJ550458, AJ550459	
B*15:30	B*1530	B62(15)^c^	B*1501V1	EFTO, GN00104, GN00108, 1-5004, 1-5026	L42296, U49900, U49901, U52171, U52172, FJ609980	C Alaez^b^
B*15:31	B*1531	B75(15)	B*1502V	ALDE, GN00110, m11209	L42145, U52173, U52174, AY640121	
B*15:32	B*1532	B62(15)	—	DCH036, 12WDCH038, 12WDCH027, JMDP36K024, SZ-85	X95410, U83580, U83581, AB436622, FJ986201	K Tadokoro^b^, HY Zou^b^
B*15:33	B*1533	B15	—	GN00103	U49898, U49899	
B*15:34	B*1534	B62(15)^c^	—	GN00105, CB1078/02	U49902, U49903, AJ549112, AJ549113	
B*15:35	B*1535	B62(15)^c^	—	GN00106, UCLA447, JMDP01K067	U52167, U52168, AJ968559, AB536871	T Gervais^b^, K Tadokoro^b^
B*15:36	B*1536	—	B*15MD	MD674	U58315, U58316	
B*15:37	B*1537	B70^c^	—	11112331, CTM1984782	U55022, U55023, AF016641	
B*15:38:01	B*153801	—	—	#10, TBC38656	U95084, U95085, AB285119	M Satake^b^
B*15:38:02	B*153802	—	—	HN-43380-8	FJ346336	Histogenetics
B*15:39	B*1539	B62(15)^c^	ZA016, B*15MZH	ZA016, GN00177, T228, NM3906, 1510200313, JMDP01K068	AF016302, AF009681, AF017080, AF017081, AF033501, AF033502, AF060504, AF060505, AF033501, AF033502, AB536872	K Tadokoro^b^
B*15:40	B*1540	—	—	GN00181, GN00206, NT00762	AF028597, AF028598, AF054003, AF054004, EU330461	CK Hurley^b^
B*15:42	B*1542	—	B*15/55Var	PB(16962), 43885	Y15841, AJ749950, AJ966738	J Rowlands^b^
B*15:43	B*1543	—	B*1501Var2	GN00211	AF054011, AF054012	
B*15:44	B*1544	—	B*1521Var	GN00212	AF061857, AF061858	
B*15:45	B*1545	B62(15)	B*15JL	J.L, GN00219	AJ007605, AJ007606, AF071765, AF071766	
B*15:46	B*1546	B72(70)	B*15UL, B1501V2	S.Z., 97-02707, JCBB13806, TBC46587	AJ007603, AJ007604, AF110250, AF110251, AF110252, AB036049, AB185095	
B*15:47	B*1547	—	—	346-516, 30129	AF07265, AF072266, AJ536757, AJ536758	
B*15:48	B*1548	B62(15)	—	009326174/HR1858	AF072377, AF072378	
B*15:49	B*1549	—	B*1503V	NMDP#016220287	AF105029, AF105030	
B*15:50	B*1550	—	B*1501Variant	121-08035	AF108424, AF108425	
B*15:51	B*1551	B70	B*NO = B*27New	NO-ARCBS	AF117766, AF117767	
B*15:52	B*1552	B15	B*15 Variant	01223584, UCLA01203301, GN00288, 99-2200, GN00328, GN00343	AF127810, AF127811, AF132488, AF172869, AF172870, AF176075, AF176076, AF189248, AF189249, AF189250, AF202451, AF202452, AF226152, AF226153	
B*15:53	B*1553	—	B*15 Variant	012436002, NT01095	AF129296, AF129297, AF132487, GQ251357	CK Hurley^b^
B*15:54	B*1554	—	B*1503v	GN00257, E3541	AF135536, AF135537, AJ245869	
B*15:55	B*1555	B15	B*1531new	T2059	AJ249316, AJ249317, AJ249318, AJ249319, AJ249320, AJ249321, AJ249322	
B*15:56	B*1556	—	B*1501V2	GN00315, BY00302	AF181846, AF181847, EU555315	CK Hurley^b^
B*15:57	B*1557	—	B*15New	NDS-758	AF188885, AF188886, AF188887	
B*15:58	B*1558	B62(15)	B*15KSW, ?B62(15)	99-2202, KSW, SZ-101	AF190278, AF190279, AF190280, AF184607, AF184608, FJ986202	HY Zou^b^
B*15:60	B*1560	—	B1501V4	JCBT1283	AB036050	
B*15:61	B*1561	—	B*1503V	1999-158-3366, BY00561	AF251356, AF251357, GU138065	CK Hurley^b^
B*15:62	B*1562	—	—	GN00363, NT00726	AF266527, AF266528, EF536013	CK Hurley^b^
B*15:63	B*1563	—	B*1545V	Toba44, GN00364, JMDP36K025	AF275626, AF275627, AF281150, AF281151, AB436623	K Tadokoro^b^
B*15:64	B*1564	—	B*1518V	GN00367	AF279111, AF279112	
B*15:65	B*1565	—	B*CB3654	CB3654	AF335310, AF335311	
B*15:66	B*1566	—	—	UCB-163-1999	AJ308399	
B*15:67	B*1567	—	—	MCH104, ML1777	AF335547	
B*15:68	B*1568	B35	B*15/48	13365831, H050-2	AY033429, AY033430, AY033431, EF101896	C Wang^b^
B*15:69	B*1569	—	B*15var	BHCP, 03/102308_AH	AJ298282, AJ298289, AJ632337, AJ632338	
B*15:70	B*1570	B62(15)	—	285D	AY057402, AY057403	
B*15:71	B*1571	B62(15)	—	FH66, FH67	AY065827, AY065828, AY065829	
B*15:72	B*1572	—	—	FH60	AY065830, AY065831, AY065832	
B*15:73	B*1573	B62(15)	—	11470, 28580, 4268	AJ459483, AJ489936, AJ489937, EU715289	FdP Sanchez Gordo^b^
B*15:74	B*1574	—	—	0455-5706-3, BY00410	AJ507655, AJ507656, FJ619486	CK Hurley^b^
B*15:75	B*1575	—	—	GN00425, GN00427	AY178185, AY178186, AY178189, AY178190	
B*15:76	B*1576	—	—	111542	AJ535668	
B*15:77	B*1577	—	—	10143636	AJ549201	
B*15:78:01	B*157801	B15	JMSH	GWML, 14513382	AJ550947, AJ550948, AM085498	J Rowlands^b^
B*15:78:02	B*157802	B15	—	HN-42973-7	FJ235026	Histogenetics
B*15:79N	B*1579N	Null	—	SCCA-01	AY303573, AY303574	
B*15:80	B*1580	B70	B1518var	GR1202	AJ564721	
B*15:81	B*1581	—	—	TBC-T4719	AB087510, AB087511, AB087512	
B*15:82	B*1582	B62(15)	B*15MAN	MAN	AJ579858, AJ579859, AJ579860	
B*15:83	B*1583	—	—	EVKA	AJ586140	
B*15:84	B*1584	B62(15)	—	TBC43137	AB174891, AB174892, AB174893	
B*15:85	B*1585	—	—	HL-03-2236	AY596955, AY596956	
B*15:86	B*1586	—	—	Yang-Z, Yang-Zp, Yang-h, VTIS123353, B2727	AY604038, AY766187, AY787588, AY787589, AY787590	
B*15:87	B*1587	—	—	HL-03-3416	AY596957, AY596958	
B*15:88	B*1588	—	—	212537	AJ633569	
B*15:89	B*1589	—	—	159633, 12964	AJ697648, AJ876903	R Collins^b^
B*15:90	B*1590	—	—	m11900	AY640122	
B*15:91	B*1591	—	—	m11430	AY640123	
B*15:92	B*1592	—	B*1501V8	TBC333193	AB183462	
B*15:93	B*1593	B71(70)	B*1518V1	TBC45841	AB185094	
B*15:94N	B*1594N	Null	B*1501N1	TBC45516	AB187125	
B*15:95	B*1595	—	—	85498	AY833432	
B*15:96	B*1596	B62(15)	—	17910	AJ812565	
B*15:97	B*1597	—	—	MHHZ-00013000	AJ871413	R Blasczyk
B*15:98	B*1598	—	—	NT00523	AY877249, AY877250	(147)
B*15:99	B*1599	—	—	NT00518	AY877255, AY877256	(147)
B*15:101	B*9501	—	B*15KEM1204	MHHZ-00013118	AJ880425	R Blasczyk
B*15:102	B*9502	B62(15)	—	TBCT2876	AB183460	M Satake
B*15:103	B*9503	B70	—	15183	AJ504800, AJ504801, AJ504802	EMvdB Loonen
B*95:104	B*9504	—	—	TBC55192	AB218625	M Satake
B*15:105	B*9505	—	—	NT00611	DQ167211, DQ167212	(149)
B*15:106	B*9506	—	—	DNA-0206, 79475	AM075816, AM904558	M Bengtsson, JDH Anholts^b^
B*15:107	B*9507	B62(15)	—	13890190	AM087018	(162)
B*15:108	B*9508	B71(70)	—	CTMAL-5469642	DQ241733	JL Vicario
B*15:109	B*9509	—	—	BY00079	DQ244125, DQ244126	(163)
B*15:110	B*9510	—	—	NT00631	DQ334735, DQ334736	(149)
B*15:111N	B*9511N	Null	—	TBC58168	AB247568	M Satake
B*15:112	B*9512	B15	—	Chendu6060	DW364132	(164)
B*15:113	B*9513	—	—	VTIS139151, BY00468, HN-8470600, HN5219230, HN-6931163, HN-5218992	DQ400510, DQ400511, FJ976684, FJ765811, FJ594722, FJ875655, FJ875656	BD Tait, CK Hurley^b^, Histogenetics^b^
B*15:114	B*9514	B70	—	HXX1277	AM229665, AM229666	(165)
B*15:115	B*9515	—	—	BY00084	DQ455016	(77)
B*15:116	B*9516	—	—	6090213	AM259370	A Wölpl
B*15:117	B*9517	—	—	BY000123	DQ832587	CK Hurley
B*15:118	B*9518	—	—	TJBC-9741	DQ864729	(166)
B*15:119	B*9519	—	—	NT00675	DQ832585	(149)
B*15:120	B*9520	—	B*1501V7	TBC183836	AB183461	M Satake
B*15:121	B*9521	—	—	K33818	DQ912834	(167)
B*15:122	B*9522	—	—	261464	DQ885886	K Hirv
B*15:123	B*9523	—	—	2564213	EF183477	(168)
B*15:124	B*9524	—	—	CB4630	EF187276	(169)
B*15:125	B*9525	—	B*15MVE0906	MHHN-189445, HN-146241, HN-487222, NT01098	AM407884, FJ594723, FJ853766, GQ251360	R Blasczyk, Histogenetics^b^, CK Hurley^b^
B*15:126	B*9526	—	—	33154	EF207231	(170)
B*15:127	B*9527	—	—	93977	EF215531	MS Leffell
B*15:128	B*9528	—	—	MATHMar	AM491588	A Dormoy
B*15:129	B*9529	—	—	T14321	EF473219	(171)
B*15:131	B*9531	—	—	147351	AM712922	(120)
B*15:132	B*9532	—	—	DCI-001	EF620033	S Fossey
B*15:133	B*9533	—	—	BJ044	EU022523	L Wang
B*15:134	B*9534	—	—	P2476	EU046491	(172)
B*15:135	B*9535	—	—	MHHAKB-438106, HN-24099-5, HN-28758-2, HN-1086698, HN-54905-7	AM778232, FJ594688, FJ594691, FJ600618, FJ853761	R Blasczyk, Histogenetics^b^
B*15:136	B*9536	—	—	B13403	EU081879	(173)
B*15:137	B*9537	—	—	BJ047	EU099586	Z Zhang
B*15:138	B*9538	—	—	BJ048	EU091341	S Cui
B*15:139	B*9539	—	—	BJ046	EU079372	L Wang
B*15:140	B*9540	—	—	D28140	AM905050	O Avinens
B*15:141	B*9541	—	—	FR, BY00277, BY00301, HN-17577-5, HN-371750, HN-4424500, HN-2603084, HN-1474995	EU378915, EU522473, EU555317, FJ765831, FJ594725, FJ594728, FJ594687, FJ875659	FB Johnson, CK Hurley^b^, Histogenetics^b^
B*15:142	B*9542	—	—	NT00781	EU484049	CK Hurley
B*15:143	B*9543	—	—	BY00285	EU522465	(36)
B*15:144	B*9544	—	—	CTJ-18300	EU547493	L Yan
B*15:145	B*9545	—	—	BY00288	EU555328	(36)
B*15:146	B*9546	B62(15)	—	CS00003, HCP-95	EU564806, AM711637	K Cao, Odl Calle-Martin
B*15:147	B*9547	—	—	BY00314, HN-55784-3, HN-1208383, HN-198192	EU643602, FJ853777, FJ765798, FJ875669	(36), Histogenetics^b^
B*15:148	B*9548	—	—	JMDP36K004	AB434759	K Tadokoro
B*15:149N	B*9549N	Null	—	SZ-15	EU693920	(174)
B*15:150	B*9550	—	—	317529-B15new, Jan-20, HN-9419667	AM980457, FJ360529, FJ765556	T Lebedeva, (175)^b^, Histogenetics^b^
B*15:151	B*9551	—	—	RMAT	AM747238	(176)
B*15:152	B*9552	—	—	SDBC-HLA-3	FJ374888	W He
B*15:153	B*9553	—	—	14343/08, HN-71150-9, HN-70918-2, HN-04085-5, HN-76459-8, HN-17386-8, HN-50145-3, HN-61040-4, HN-96967-5, HN-51811-9, HN-50489-5, HN-77532-2, HN-01906-4, HN-91313-3, HN96223-1, HN-1153829, HN-21535-8, HN-63152-6, HN-57199-8, HN-57761-5, HN-33766-3, HN-75152-5, HN-73160-1, HN-88641-3, HN-94111-9, HN-86738-9, HN-35520-8	FM957458, FJ502329, FJ502331, FJ235006, FJ235036, FJ235067, FJ346298, FJ346317, FJ853791, FJ866154, FJ866158, FJ866162, FJ866164, FJ866171, FJ866174, FJ765568, FJ765769, FJ765846, FJ765946, FJ765947, FJ765974, FJ875565, FJ898493, GQ149278, GQ245745, GQ254341, GQ859547	S Wienzek, Histogenetics^b^
B*15:154	B*9554	—	—	BY00398, HN-22104-7, HN-25903-0, HN-03065-2, HN-47125-8, HN-0042411	FJ619496, FJ346294, FJ866153, FJ866169, FJ866181, FJ765803	CK Hurley, Histogenetics^b^
B*15:155	B*9555	—	—	BY00400, HN-7712737	FJ619495, FJ765551	CK Hurley, Histogenetics^b^
B*15:156	B*9556	—	—	BY00416	FJ619479	CK Hurley
B*15:157	B*9557	—	—	HN-85486-9, BY00432, HN-23183-7, HN-14375-2, HN-98600-9, HN-30172-8	FJ346268, FJ688160, FJ765814, FJ866163, FJ866145, FJ549408	Histogenetics, CK Hurley
B*15:158	B*9558	—	—	NT01024, HN-697595	FJ797378, FJ468326	CK Hurley, Histogenetics^b^
B*15:159	B*9559	—	—	NT01012, HN-749933, HN-2141102	FJ797390, FJ489872, FJ976855	CK Hurley, Histogenetics^b^
B*15:160	B*9560	—	—	NT01028, HN-54413-3, HN-777132, HN-16411-8	FJ797374, FJ235040, FJ549409, FJ765984	CK Hurley, Histogenetics^b^
B*15:161	B*9561	—	—	ATAB3398AN	FM211648	AM Little
B*15:162	B*9562	—	—	HN-3334, HN-1088	FJ235004, FJ765539	Histogenetics
B*15:163	B*9563	—	—	JMDP36K035	AB477104	K Tadokoro
B*15:164	B*9564	—	—	NT01070	GQ251348	CK Hurley
B*15:165	B*9565	—	—	HN-64402-5	FJ235069	Histogenetics
B*15:166	B*9566	—	—	HN-56415-4	FJ765857	Histogenetics
B*15:167	B*9567	—	—	HN-19327-4, HN-42954-6	FJ346236, FJ866183	Histogenetics
B*15:168	B*9568	—	—	NT01067	GQ251345	CK Hurley
B*15:169	B*9569	—	—	JMDP36K036	AB477105	K Tadokoro
B*15:170	B*9570	—	—	BY00490, HN-6944719	GQ410109, FJ765789	CK Hurley, Histogenetics^b^
B*15:171	B*9571	—	—	HN-36446-4	FJ346306	Histogenetics
B*15:172	B*9572	—	—	HN-44141-2, HN-04380-8	FJ346309, FJ898491	Histogenetics
B*15:173	B*9573	—	—	HN-90206-8	FJ346332	Histogenetics
B*15:174	B*9574	—	—	HN-35843-4	FJ554618	Histogenetics
B*15:175	B*9575	—	—	HN-51953-8	FJ765540	Histogenetics
B*15:176	B*9576	—	—	HN-41015-8, HN-23024-7	FJ853788, FJ866179	Histogenetics
B*15:177	B*9577	—	—	HN-31725-7	FJ853804	Histogenetics
B*15:178	B*9578	—	—	5790900505	GQ845008	L Yan
B*15:179	B*9579	—	—	JS0901	GQ468520	C Wang
B*15:180	B*9580	—	—	COH09-03370, COH09-03372	GQ912703	K Gendzekhadze
B*15:181N	B*9581N	Null	—	HN-72542-2, HN-62535-9, HN-95028-4	FJ875640, FJ875647, GQ245715	Histogenetics
B*15:182N	B*9582N	Null	—	HN-95367-1	FJ875653	Histogenetics
B*15:183	B*9583	—	—	HN-6117736	FJ765817	Histogenetics
B*15:184	B*9584	—	—	HN-44736612	FJ765960	Histogenetics
B*15:185	B*9585	—	—	BY00534	GU066746	CK Hurley
B*15:186	B*9586	—	—	JMDP36K033	AB477102	K Tadokoro
B*18:01:01	B*180101	B18	—	SGAR, F24, MM1801, VEN, BY00058, MHH977560, QBL	M24039, U90559, AJ310507, DQ007938, AJ505010, AJ505011, AJ495878, AJ495932, AJ495986, AL845443	CK Hurley^b^, (177)^b^, (105)^b^
B*18:01:02	B*180102	B18	—	6ABC124	AY045737, AY045738	
B*18:01:03	B*180103	B18	—	90233	DQ886702	MS Leffell
B*18:01:04	B*180104	B18	—	HN-69205-5	FJ346335	Histogenetics
B*18:01:05	B*180105	B18	—	HN47585-7	FJ866159	Histogenetics
B*18:01:06	B*180106	B18	—	HN-3233058	FJ494824	Histogenetics
B*18:01:07	B*180107	B18	—	HN-45151-5	FJ765926	Histogenetics
B*18:01:08	B*180108	B18	—	HN-7694	FJ875562	Histogenetics^b^
B*18:02	B*1802	B18	B18PE	PETCH, BY00059, SZ-63	D25275, DQ007939, GQ118994	CK Hurely^b^, HY Zou^b^
B*18:03	B*1803	B18	B1803	BM66, GSW002, T36121	X94480, Y07824, AJ309979	
B*18:04	B*1804	B18^c^	B*18IM	IMM348, 11542527	U38792, U38793, AM110757	J Rowlands^b^
B*18:05	B*1805	B18	B*18GSW	GSW001, DZA1, GN00341	Y07710, AJ002676, AY608898, AY608899	
B*18:06	B*1806	B18	—	CTM-9985836, BY00559	AF033351, GU138067	CK Hurley^b^
B*18:07:01	B*180701	—	B*MF	GN00210, MF-ARCBS	AF054009, AF054010, AF117774, AF117775	
B*18:07:02	B*180702	—	—	HN-28357-5, HN-35293-4, HN-39607-9	FJ765844, FJ866173, GQ994065	Histogenetics
B*18:08	B*1808	—	B*1801New	NM4b448, NT01113	AF148636, AF148637, GQ373160	CK Hurley^b^
B*18:09	B*1809	B18	B18OP	6259OP, GN00345	AJ243374, AJ243376, AF274500, AF274501	
B*18:10	B*1810	—	B*1801V	GN00324	AF198650, AF198651	
B*18:11	B*1811	—	—	GN00362, NT00776	AF266525, AF266526, EU375809	CK Hurley^b^
B*18:12	B*1812	—	B*1801V	GN00366, NT01086	AF275716, AF275717, GQ251379	CK Hurley^b^
B*18:13	B*1813	—	—	2000-56-617, BY00408	AF310138, AF310139, FJ619488	CK Hurley^b^
B*18:14	B*1814	—	—	2000-224-257, 00-809	AY042672, AY042673, AF403249	
B*18:15	B*1815	—	—	2000-084-2159	AY042686, AY042687	
B*18:17N	B*1817N	Null	—	WVAN, AVAN	AF416771	
B*18:18	B*1818	—	—	28626	AJ489938, AJ489939	
B*18:19	B*1819	—	—	232305, 83084	AY331381, AJ581457, AJ581458, AJ581459	
B*18:20	B*1820	—	—	166757, NT00522	AJ634463, AY935262, AY903433, AY903434	CK Hurley^b^
B*18:21	B*1821	—	—	Z-00018417	AM050157	R Blasczyk
B*18:22	B*1822	—	—	BY00078, BY00455, BY00456	DQ244137, DQ244138, DQ244139, FJ842967, FJ842968	(88), CK Hurley^b^
B*18:23N	B*1823N	Null	—	DZA05-5	AM403098	S Ulrich
B*18:24	B*1824	—	—	R1088	EF050739	M Yu
B*18:25	B*1825	—	—	91962, NT00782, BY00321, HN-6764895	EF141101, EU484048, EU643595, FJ875654	MS Leffell, CK Hurley^b^, Histogenetics^b^
B*18:26	B*1826	—	—	K110228, BY00442	AM492197, FJ688150	AM Little, CK Hurley^b^
B*18:27	B*1827	—	—	LUMC-B14	AM746478	(64)
B*18:28	B*1828	—	—	MHHAKB-438906, MHHAKB-477183, MHHAKB-468157, HN-31631-5, HN95633-5, HN-08910-2, HN-71468-5, HN-54020-4	AM906169, AM931047, AM931065, FJ594548, FJ594551, FJ594552, FJ594594, FJ868465	P Horn, Histogenetics^b^
B*18:29	B*1829	—	—	BY00281	EU522469	(36)
B*18:30	B*1830	—	—	BY00343	EU924809	CK Hurley
B*18:31	B*1831	—	—	BY00345	EU924807	CK Hurley
B*18:32	B*1832	—	—	179199, HN-7716157, HN-7714160	FM211161, FJ866144, FJ392170	(178), Histogenetics^b^
B*18:33	B*1833	—	—	BY00396, HN-08399-6, HN-5903965, HN-35697-8, BY00511, BY00530, HN-02964-7	FJ619498, FJ853772, FJ875667, FJ875568, GQ410123, GU066742, GQ859551	CK Hurley, Histogenetics^b^
B*18:34	B*1834	—	—	HN-64083-9, HN-64781-8, HN-93799-5, HN-42936-1	FJ392181, FJ346277, FJ346279, FJ866178, FJ765953	Histogenetics
B*18:35	B*1835	—	—	60734C32, HN-N96546, HN-N260491	GQ250939, GQ240386, GQ900552	MdG Bicalho, Histogenetics^b^
B*18:36	B*1836	—	—	HN-1500441	FJ235019	Histogenetics
B*18:37	B*1837	—	—	HN-28873559	FJ235020	Histogenetics
B*18:38	B*1838	—	—	HN-12592-0	FJ346247	Histogenetics
B*18:39	B*1839	—	—	HN-60114-5, HN-96918-7	FJ346251, FJ853784	Histogenetics
B*18:40	B*1840	—	—	HN-51843-2, HN-8917026	FJ346295, FJ853806	Histogenetics
B*18:41	B*1841	—	—	HN-15248-8	FJ494818	Histogenetics
B*18:42	B*1842	—	—	HN-61476-7	FJ494830	Histogenetics
B*18:43	B*1843	—	—	HN-08065-3, HN-81457-1	FJ549412, FJ952584	Histogenetics
B*18:44	B*1844	—	—	HN-24460-8, HN-89082-8	FJ765815, FJ875559	Histogenetics
B*18:45	B*1845	—	—	HN-38283-8	FJ765816	Histogenetics
B*18:46	B*1846	—	—	HN-1713168	FJ765796	Histogenetics
B*18:47	B*1847	—	—	HN-4276, HN-33661-9	FJ765819, GQ468246	Histogenetics
B*27:01	B*2701	B27	27f	LH, PIL-139	L76935	
B*27:02	B*2702	B27	27e, 27K, B27.2	BRUG, NV, KSH	X03664, X03667, L38504, U18659	
B*27:03	B*2703	B27	27d, 27J	CH (CHI)	M54883	
B*27:04:01	B*270401	B27	27b, 27C, B27.3	WEWAK 1, DH, DEW-ND, SZ-76	U27608, GQ118997	HY Zou^b^
B*27:04:02	B*270402	B27	—	2005042857	DQ088143, DQ088144	(179)
B*27:04:03	B*270403	B27	—	NT01073	GQ251382	CK Hurley
B*27:05:02	B*270502	B27	27a, 27W, B27.1	CD, HC, MRWC, KCA, MVL, LG2, BRUG, BTB, CU40B	X03945, M12967, L20086, M14013, M12678, AJ420238, EF203078	(160)
B*27:05:03	B*270503	B27	B27MW	HHE, SF0002	X83727, X83737, AJ548757, AJ548758	
B*27:05:04	B*270504	B27	FMVB27	20836	AJ250630, AJ250631, AJ250632	
B*27:05:05	B*270505	B27	—	8998871, 6998872	AF480612	
B*27:05:06	B*270506	B27	B27v	610916	AJ505129, AJ505130	
B*27:05:07	B*270507	B27	—	GN00360, NT00696, LUMC-B49	AF266521, AF266522, AY700219, EF176682, AM946390	CK Hurley^b^, JDH Anholts^b^
B*27:05:08	B*270508	B27	—	MHH0308622	AJ871404	R Blasczyk
B*27:05:09	B*270509	B27	—	2005-1295, HN-11822-8	AJ969671, FJ976781	(180), Histogenetics^b^
B*27:05:10	B*270510	B27	—	93520, HN-2922057	EF215529, FJ600616	MS Leffell, Histogenetics^b^
B*27:05:11	B*270511	B27	—	HN-91474-5	FJ235071	Histogenetics
B*27:05:12	B*270512	B27	—	HN-56115-3	FJ392173	Histogenetics
B*27:05:13	B*270513	B27	—	HN-77796-1	FJ765818	Histogenetics
B*27:06	B*2706	B27	27D, B27.4	LIE, PAR, TER Cell#995, 10130285	X73578, U35734, AJ292971, AJ550736	
B*27:07	B*2707	B27	B27-HS	HS, 23444, SZ-94	M62852, AM233567, GQ118995	T Gervais^b^, HY Zou^b^
B*27:08	B*2708	B2708	B7Qui	19418, BCK, 33402	L19923, AJ849644	
B*27:09	B*2709	B27	B27-ci	Ci, NT00775	Z33453, EU375810	CK Hurley^b^
B*27:10	B*2710	B27	B2705v	KRICO, NMDP0392-7903-9	L76095, AJ308990, AJ310147	
B*27:11	B*2711	B27	B27KH	K.H.	D83043	
B*27:12	B*2712	B27	WIS1/C846	RW, MT3, RK, CTM4896, 38256	U90244, U90245, Y14582, AF022783, AJ577721	
B*27:13	B*2713	B27	B27052W496D	W496D	AF026218	
B*27:14	B*2714	B27^c^	—	65-90810, 01168999	AF072763, AF072764, AF110256, AF110257, AF110258	
B*27:15	B*2715	—	B “X”-Bw6	KC, BY00437	Y16637, Y16638, FJ688148	CK Hurley^b^
B*27:16	B*2716	—	B*27052 Variant	GN00246	AF102563, AF102564	
B*27:17	B*2717	B27	B27TO	4388TO	AJ243373, AJ243375	
B*27:18	B*2718	—	—	99-2198	AF189012, AF189013, AF189014	
B*27:19	B*2719	B27	—	BFLR	AF190146, AF190147	
B*27:20	B*2720	B27	B*27CHN	KMP01-1379, BY00555	AF170578, AF170579, GU138071	CK Hurley^b^
B*27:21	B*2721	—	B*2706V	GN00334	AF218578, AF218579	
B*27:23	B*2723	—	B*27IG	30733VTIS, 35520	AF305196, AF305197, AJ298262	
B*27:24	B*2724	—	—	2000-161-3004, SZ-33	AY042670, AY042671, GQ304759	HY Zou^b^
B*27:25	B*2725	—	—	2000-119-979, SZ-93	AF408160, AF408161, GQ118996	HY Zou^b^
B*27:26	B*2726	—	—	m11702	AY640120	
B*27:27	B*2727	—	—	W17962, NT00526	AY745872, AY745873, AY745874, AY877257, AY877258	CK Hurley^b^
B*27:28	B*2728	—	—	NT00514	AY877253, AY877254	(147)
B*27:29	B*2729	B27	—	UA-2004-TP-3826	AJ937708	M Bengtsson
B*27:30	B*2730	B27	—	2005/92, HN-10069-7, HN-23540-5	AJ969956, FJ594606, FJ868487	(181), Histogenetics^b^
B*27:31	B*2731	—	—	NT00589	DQ105581, DQ105582	(149)
B*27:32	B*2732	—	—	HUCL325070AN	AM087470	AM Little
B*27:33	B*2733	—	—	NG1807444	DQ367066, DQ367067	D Smillie
B*27:34	B*2734	—	—	158567	AM230596	(182)
B*27:35	B*2735	—	—	UAS13072	AM233491	M Bengtsson
B*27:36	B*2736	—	—	BY00091, SAMPLE-7319, SZ-6	DQ455018, DQ455019, DQ840458, DQ915176	(57), L Yan^b^, (183)^b^
B*27:37	B*2737	—	—	NT00686	EF173481	(85)
B*27:38	B*2738	B27	—	SAUVSer	AM689935	A Dormoy
B*27:39	B*2739	—	—	NT00755	EU275159	(85)
B*27:40	B*2740	—	—	269088	EU341811	(184)
B*27:41	B*2741	—	—	BY00298	EU555320	(36)
B*27:42	B*2742	—	—	BY00292, HN-4123369	EU555325, FJ976780	(36), Histogenetics^b^
B*27:43	B*2743	—	—	BY00318	EU643598	(36)
B*27:44	B*2744	—	—	BJ60	FJ463035	W Li
B*27:45	B*2745	—	B*27new	Henan-13	FJ411059	B Zhang
B*27:46	B*2746	—	—	BY00401, HN-5092736	FJ619494, FJ235045	CK Hurley, Histogenetics^b^
B*27:47	B*2747	—	—	HN-11895-9, I2008-5929, HN-45714-9	FJ346260, GQ848647, FJ796989	Histogenetics, M Sprague^b^
B*27:48	B*2748	—	—	HN-16480-5, HN8015-7	FJ346264, FJ866176	Histogenetics
B*27:49	B*2749	—	—	HN-36591-7, HN-09752-5	FJ392178, FJ640577	Histogenetics
B*27:50	B*2750	—	—	NT01029, HN-779823	FJ797373, FJ489871	CK Hurley, Histogenetics^b^
B*27:51	B*2751	—	—	09-360, HN-62875-5	FJ985693, GQ900555	M Chambers, Histogenetics^b^
B*27:52	B*2752	—	—	CHN73493	FJ966367	H Alves
B*27:53	B*2753	—	—	HN-36132-9, HN-52286-2, HN-52328-2	FJ235009, FJ235010, FJ235011	Histogenetics
B*27:54	B*2754	—	—	HN-1888119	FJ235018	Histogenetics
B*27:55	B*2755	—	—	HN-83547-4, HN-38259-6	FJ235063, FJ468320	Histogenetics
B*27:56	B*2756	—	—	HN-65627-6	FJ235070	Histogenetics
B*27:57	B*2757	—	—	HN-79600-2	FJ346249	Histogenetics
B*27:58	B*2758	—	—	HN-33014-4	FJ346313	Histogenetics
B*27:59N	B*2759N	Null	—	BY00521, 254459	GQ867215, FN422393	CK Hurley, T Lebedeva
B*27:60	B*2760	—	—	HN-5161570, HN-4010737, HN-90373-2	FJ765791, FJ765792, GQ149277	Histogenetics
B*27:61	B*2761	—	—	HN-0826820	FJ765808	Histogenetics
B*27:62	B*2762	—	—	HN-54802-0	FJ875554	Histogenetics
B*35:01:01	B*350101	B35	—	HS, KT17, GU2739, CMM, KT12, 1510200302	M28109-12, U11265, L63544, AJ420239, L63544	
B*35:01:02	B*350102	B35	—	GN00356	AF260977, AF260978	
B*35:01:03	B*350103	B35	—	TBC52130	AB200230	M Satake
B*35:01:04	B*350104	B35	—	NT00573	DQ007039, DQ007040	(147)
B*35:01:05	B*350105	B35	—	MHH0303078	AM050159	R Blasczyk
B*35:01:06	B*350106	B35	—	BY00106	DQ514598	(149)
B*35:01:07	B*350107	B35	—	CS00004, HN-5812299	EU564807, FJ594590	K Cao, Histogenetics^b^
B*35:01:08	B*350108	B35	—	HN-42895-5	FJ235052	Histogenetics
B*35:01:09	B*350109	B35	—	HN-27182-5	FJ235034	Histogenetics
B*35:01:10	B*350110	B35	—	BY00486	GQ410105	CK Hurley
B*35:01:11	B*350111	B35	—	HN-08590-3, HN-78334-9, HN-03705-2, HN-21479-7, HN-39298-0	FJ346319, FJ853780, FJ765554, GQ345060, GQ401196	Histogenetics
B*35:01:12	B*350112	B35	—	HN-06227-4	FJ346324	Histogenetics
B*35:01:13	B*350113	B35	—	HN-47625-2	FJ853799	Histogenetics
B*35:01:14	B*350114	B35	—	HN-5709345	FJ494826	Histogenetics
B*35:01:15	B*350115	B35	—	HN-1642615, HN-97678-7	FJ765797, FJ952582	Histogenetics
B*35:01:16	B*350116	B35	—	HN-2644283	FJ360522	Histogenetics
B*35:01:17	B*350117	B35	—	HN-692059	FJ468324	Histogenetics
B*35:01:18	B*350118	B35	—	HN-7566220	FJ765870	Histogenetics
B*35:01:19	B*350119	B35	—	HN-18209-4, HN-83813-6	FJ765985, GQ900547	Histogenetics
B*35:02:01	B*350201	B35	—	DL, 388, SZ-96	M63454, U90563, GQ119000	HY Zou^b^
B*35:02:02	B*350202	B35	B*35NO1204	MHHZ-00013111, HN-59372-1, HN-69984-7, HN-09297-7, HN-09514-5, HN-16306-7, HN-64433-0	AJ884579, FJ588695, FJ594727, FJ868484, FJ868473, FJ868477, FJ868486	R Blasczyk, Histogenetics^b^
B*35:02:03	B*350203	B35	—	HSP-B350203	FM205711	O Calle-Martin
B*35:02:04	B*350204	B35	—	09218414	GU191548	D Fuerst
B*35:03:01	B*350301	B35	—	C1R, HMY2, 12405, 13159, 093, SZ-83	M81798, D50299, U90564, GQ118998	HY Zou^b^
B*35:03:02	B*350302	B35	—	102570	FJ619519	MS Leffell
B*35:03:03	B*350303	B35	—	HN-6933607	FJ594718	Histogenetics
B*35:04:01	B*350401	B35	—	AN, RB22, 12.36JK	M86403, U30936, L47986	
B*35:04:02	B*350402	B35	—	NT00591, NT00637, NT00679, NT00716, NT00774, HN-6212559, HN-9792975	DQ096575, DQ096576, DQ334741, DQ334742, DQ984201, EF375696, EU375811, FJ594620, FJ792534	(149), CK Hurley^b^, Histogenetics^b^
B*35:04:03	B*350403	B35	—	JMDP36K007	AB435078	K Tadokoro
B*35:05	B*3505	B35	B35-G	GRC212, KRC032, TOB-115, SZ-92	M84385, L76930, GQ118999	HY Zou^b^
B*35:06	B*3506	B35	B35-K	KRC032, NT01085	M84381, GQ251378	CK Hurley^b^
B*35:07	B*3507	B35	—	#20073	L04695	
B*35:08:01	B*350801	B35	B35TL	#22338, TL, SZ-95	L04696, Z22651, GQ119001	HY Zou^b^
B*35:08:02	B*350802	B35	—	2005/1979, HN-42735-3	AM234615, FJ868466	(185), Histogenetics^b^
B*35:08:03	B*350803	B35	—	NT00699	EF195110	(85)
B*35:08:04	B*350804	B35	—	HN-08149-8, HN-08597-8, HN-06093-0, HN-25909-1	FJ346318, FJ346320, FJ346323, GQ449635	Histogenetics
B*35:09:01	B*350901	B35	—	MA9, 30	U17107, U90565	
B*35:09:02	B*350902	B35	—	WIC-54	L76932	
B*35:10	B*3510	B35^c^	—	JK1.2, JK5.13, JK14.41	L36979	
B*35:11	B*3511	B35	B35v	GRC-187, BY00556	L40599, GU138070	CK Hurley^b^
B*35:12:01	B*351201	B35	B-3504v	BAON, FEME, PNS	L42281, L76094, L49342	
B*35:12:02	B*351202	B35	—	HN-00816-0	FJ346327	Histogenetics
B*35:13	B*3513	B35	2993	RCE80, THA-DCH 0654, THA-DCH 9675	X87268, AF208430, AF208431, AF208432, AF208433	
B*35:14:01	B*351401	B35	B*35M	JLG, JGS	S83195, S83196	
B*35:14:02	B*351402	B35	—	10159026	AJ549200	
B*35:15	B*3515	B35	—	PARMG, NT00784	U30904, EU484045	CK Hurley^b^
B*35:16	B*3516	B35^c^	B*35GAR	GAR, NT00771	U29880, EU330471	CK Hurley^b^
B*35:17	B*3517	B35	B35V1, B*35PNS, B-3505v	JM (G2744), PNS, AMYE, TBC31121	U34618, L49341, L75941, AB256954	M Satake^b^
B*35:18	B*3518	B35	B-3508v	TOB-137	L75942	
B*35:19	B*3519	B35	B-40X35	WIC-54, VTIS43878	L76933, AF387905, AF387906	
B*35:20:01	B*352001	B35	B-3501V	TER-135, NT00732, Scu03771m	U76392, U76393, EF563143, EU516354	CK Hurley^b^, FdP Sanchez Gordo^b^
B*35:20:02	B*352002	B35	—	LUMC-B7, HN-8932462, HN-09326-0, HN-14830-4, HN-7412156	AM749669, FJ594597, FJ594569, FJ594574, FJ594624	(64), Histogenetics
B*35:21	B*3521	—	B-3511H	TER-109, NT01088, JMDP36K043	U76390, U76391, GQ251381, AB536873	CK Hurley^b^, K Tadokoro^b^
B*35:22	B*3522	—	M001B	M001B, NT01084	AF017327, AF009685, GQ251377	CK Hurley^b^
B*35:23	B*3523	B35^c^	MA080B	MA080B, NT01083	AF016301, AF009680, GQ251376	CK Hurley^b^
B*35:24:01	B*352401	—	MA086B	MA086B, NT01082	AF016300, AF009679, GQ251375	CK Hurley^b^
B*35:24:02	B*352402	—	—	HN-6458538	FJ765558	Histogenetics
B*35:25	B*3525	B35^c^	—	GN00215	AF061863, AF061864	
B*35:26	B*3526	—	B15/35 7-1 clone 24	NMDP#027669746, NT00705	AF105031, AF105032, EF375688	CK Hurley^b^
B*35:27	B*3527	B35	B*35JAC	JAC	Y18288, Y18289	
B*35:28	B*3528	—	B*3510Variant	304-00651, 016696205	AF108428, AF108429, AF127808, AF127809, AF132486	
B*35:29	B*3529	B35	B*KG	KG-ARCBS, GN00289	AF117770, AF117771, AF176077, AF176078	
B*35:30	B*3530	B35	B*3517Variant	GN00242, NT00727	AF110504, AF110505, EF536014	CK Hurley^b^
B*35:31	B*3531	—	B*35/40	KYR, KKW, MOV	AF138164, AF138165, AF170577, AJ278744	
B*35:32	B*3532	B35	B*TMUL	BM1 139852, NT01080	AF134866, AF134867, GQ251373	CK Hurley^b^
B*35:33	B*3533	—	B*35New	0000-3034-6, NT01096	AJ238411, AJ238412, GQ251358	CK Hurley^b^
B*35:34	B*3534	—	—	GN00329, JMDP36K029	AF205530, AF205531, AF201762, AM436627	K Tadokoro^b^
B*35:35	B*3535	B35	B3501V1, B35v	JCBT1635, JMDP36K027, BY00506, HN-03375-3	AB032093, AB436625, GQ410091, FJ875661	K Tadokoro^b^, CK Hurley^b^, Histogenetics^b^
B*35:36	B*3536	—	B*3503V	GN00353	AF282765, AF282766	
B*35:37	B*3537	—	B*35KM	DZA1999-16/MHH994949, LUMC-B48	AJ243737, AJ243738, AM946389	JDH Anholts^b^
B*35:38	B*3538	—	—	BSB620, BSB620-MO	AJ312287	
B*35:39	B*3539	—	—	2000-140-1975	AY042688, AY042689	
B*35:40N	B*3540N	Null	—	IBTC-B35N	AJ418040	
B*35:41	B*3541	B35	—	2HT21, WAC1087870, CAP13, 40795, 41405, Halmar	AY045735, AY045736, AF480613, AF497262, AJ606947, AJ606948, AM076816	P Dunn^b^
B*35:42	B*3542	B35	—	MS21871	AJ316289, AJ426469, AJ426468, AJ417680, AJ417669	
B*35:43	B*3543	B35	B15UW1, B35V2, B*1522, B15/35 7-1 clone 27	1274, B503, JC (G2997), FFAJ, NMDP#027669746	U14756, L42506, U34619, U80945, AF106630, AF106631	
B*35:44	B*3544	B35	B*1559	013221023	AF206514, AF206515	
B*35:45	B*3545	—	—	KE-SE	AJ509160, AJ509161	
B*35:46	B*3546	B35	—	HSR107050	AJ554211	
B*35:47	B*3547	—	—	81235	AY445028	
B*35:48	B*3548	—	—	D33202, NT00707	AY484704, AY484705, EF375687	CK Hurley^b^
B*35:49	B*3549	—	—	NF011304IH01, NT00725	AY569160, EF490365	CK Hurley^b^
B*35:50	B*3550	B35	—	TBC41783	AB174782, AB174783, AB174784	
B*35:51	B*3551	B35	—	TBC41839	AB174785, AB174786, AB174787	
B*35:52	B*3552	B35	B*3501V4	TBC45987	AB183524	
B*35:53N	B*3553N	Null	B*3501N1	TBC45366	AB185096	
B*35:54	B*3554	—	—	SanSm	AJ745149	
B*35:55	B*3555	B35	—	88713	AJ862824	
B*35:56	B*3556	—	—	28009	AJ783755	
B*35:57	B*3557	B35	—	DS2004122V1, DS2004122V2	AY841863	D Smith
B*35:58	B*3558	—	—	NT00565	AY907708, AY907709	(147)
B*35:59	B*3559	—	—	R503, NT00627	AY904045, DQ334727, DQ334728	SG Rodriguez-Marino, CK Hurley^b^
B*35:60	B*3560	B35	—	TBC53336	AB211960	M Satake
B*35:61	B*3561	B35	—	MHHZ-00016871, MHHZ-00015289	AM039491	R Blasczyk
B*35:62	B*3562	—	—	WAJ621, NT00687	AM114412, EF173480	K Leeber, CK Hurley^b^
B*35:63	B*3563	—	—	B05-2224	DQ321673	(186)
B*35:64	B*3564	—	—	2005101152, JMDP36K045	DQ301507, DQ301508, AB536874	(187), K Tadokoro^b^
B*35:65Q	B*3565Q	—	B*35MVE0904	MHH999711, MHHZ-0001223, HN-95563-0	AJ278746, AJ278747, AJ879892, FJ868490	(188), Histogenetics^b^
B*35:66	B*3566	—	—	BY00081	DQ401172, DQ401173	(149)
B*35:67	B*3567	—	—	TBC14770	AB248241	M Satake
B*35:68:01	B*356801	—	—	BY00126	DQ888174	(149)
B*35:68:02	B*356802	—	—	BY00280, BY00295, BY00296, HN-6050321, HN-3734675	EU522470, EU555322, EU557367, FJ594618, FJ594550	(36), Histogenetics^b^
B*35:69	B*3569	—	—	HSE-11012(JGS)	EF025767	(111)
B*35:70	B*3570	B35	—	P4881	AM410967	(189)
B*35:71	B*3571	—	—	58277	EF126033	B Salam
B*35:72	B*3572	—	—	BY00142, BY00257	EU173483, EU146146	(163), CK Hurley^b^
B*35:74	B*3574	—	—	NT00704, BY00411, HN-78621-1	EF375689, FJ619485, FJ868464	(85), CK Hurley^b^, Histogenetics^b^
B*35:75	B*3575	—	—	DGLM1	EF426474	L Mele
B*35:76	B*3576	B35/22	—	7032086	AM495256	(190)
B*35:77	B*3577	—	—	DCI-002, HN-70792-1, HN-55225-9, HN-8942156	EF620032, FJ594587, FJ594591, FJ600624	S Fossey, Histogenetics^b^
B*35:78	B*3578	—	—	K112206	AM747815	C Dunne
B*35:79	B*3579	—	—	scu00946	EF679203	(139)
B*35:80	B*3580	B35	—	F866	EU056568	(191)
B*35:81	B*3581	—	—	FFM86952	AM778468	(192)
B*35:82	B*3582	—	—	AKB27410	AM849047	S Schwab
B*35:83	B*3583	—	—	AKB34975	AM849048	S Schwab
B*35:84	B*3584	—	—	BY00263	EU185515	(36)
B*35:85	B*3585	—	—	LUMC-B37	AM904557	(64)
B*35:86	B*3586	—	—	NT00711	EF375686	CK Hurley
B*35:87	B*3587	—	—	MHHZ-00023391, N-564211, HN-451933	AM493308, AM920501, FJ765987	R Blasczyk, Histogenetics^b^
B*35:88	B*3588	—	—	BY00269	EU330466	CK Hurley
B*35:89	B*3589	—	—	VTIS163461	EU589203	(193)
B*35:90	B*3590	—	—	BY00316	EU63600	(36)
B*35:91	B*3591	—	—	BY00319	EU643597	(36)
B*35:92	B*3592	—	—	JMDP36K005	AB434760	K Tadokoro
B*35:93	B*3593	—	—	JMDP36K007x	AB434761	K Tadokoro
B*35:94	B*3594	—	—	JMDP01K014	AB435234	K Tadokoro
B*35:95	B*3595	B35	—	DEMBG-2009398, BY00419	FJ639840, FJ649611	A Parkner, CK Hurley
B*35:96	B*3596	—	—	HN-93873-9, HN-12379-4, HN-64266-0	FJ346269, FJ494828, FJ853789	Histogenetics
B*35:97	B*3597	—	—	BY00434, HN-0361266	FJ688158, FJ765782	CK Hurley, Histogenetics^b^
B*35:98	B*3598	—	—	HN-16151-4, HN-61859-0	FJ560459, GQ468249	Histogenetics
B*35:99	B*3599	—	—	BY00446, BY00449	FJ973351	CK Hurley
B*37:01:01	B*370101	B37	—	KAS011, MG, GU2760, TER-EXT#22	M32320, U11267, AJ310358	
B*37:01:02	B*370102	B37	—	NT00628, HN-84428-3	DQ334729, DQ334730, FJ594557	(149), Histogenetics^b^
B*37:01:03	B*370103	B37	—	BY00082, BY00412, HN-62256-5, HN-108187	DQ436824, DQ436825, FJ619483, FJ594586, FJ594621	(163), CK Hurley^b^, Histogenetics^b^
B*37:01:04	B*370104	B37	—	TBC60885	AB261695	M Satake
B*37:01:05	B*370105	B37	—	SZ-20	FJ644941	(194)
B*37:01:06	B*370106	B37	—	HN-5845263	FJ234996	Histogenetics
B*37:01:07	B*370107	B37	—	HN-22828-0, HN-06775-4, HN-63822-8	FJ346328, FJ346329, FJ765971	Histogenetics
B*37:02	B*3702	—	B27-37	CTM-8958127	U31971	
B*37:03N	B*3703N	Null	B*37OMI	OMI	AJ277845	
B*37:04	B*3704	B37^c^	—	GN00382, H156H2	AF303101, AF303102, AF389378	
B*37:05	B*3705	—	—	CMC2	AF284826, AF284827, AF284828	
B*37:06	B*3706	—	—	G0424040472433, BY00259	AY623603, AY623604, EU185519	CK Hurley^b^
B*37:07	B*3707	—	—	TBC46340, LFC197	AB187126, AM114415	K Lebeer^b^
B*37:08	B*3708	B37	—	166097	AJ829548	
B*37:09	B*3709	B37	—	86494	AY957461	MS Leffell
B*37:10	B*3710	B37	—	108616	AJ969934	(195)
B*37:11	B*3711	—	—	NT00649	DQ401184, DQ401185	(149)
B*37:12	B*3712	—	—	BY00100	DQ473294	(77)
B*37:13	B*3713	—	—	BJ032	EF472970	(196)
B*37:14	B*3714	—	—	2007-1414	AM993049	(197)
B*37:15	B*3715	—	—	HN-85082-6	FJ235023	Histogenetics
B*37:16	B*3716	—	—	HN-13775-2	FJ765548	Histogenetics
B*37:17	B*3717	—	—	HN-78280-7, HN-71035-7, HN-02244-8, HN-04271-5	FJ502328, FJ898492, FJ898490, GQ859545	Histogenetics
B*37:18	B*3718	—	—	HN-78815-7	FJ853773	Histogenetics
B*37:19	B*3719	—	—	BY00526	GQ867220	CK Hurley
B*37:20	B*3720	—	—	HN-8481	FJ765820	Histogenetics
B*37:21	B*3721	—	—	HN-66524-7	FJ765972	Histogenetics
B*38:01:01	B*380101	B38(16)	B16.1	Z, JAP-NF, YAR, JBUSH, TEM, WDV, ELON, LB96-SAR, PAT144, PAT495	M29864, L36591, U40498, DQ249175, DQ249179	(141)^b^
B*38:01:02	B*380102	B38(16)	—	NT00650	DQ401178, DQ401179	(149)
B*38:01:03	B*380103	B38(16)	—	HN-30368-4	FJ346250	Histogenetics
B*38:01:04	B*380104	B38(16)	—	HN-1364251	FJ600631	Histogenetics
B*38:02:01	B*380201	B38(16)	—	RSA-ND, Terasaki EXT#58, 32764	L22028, AJ297317, AJ308991, AJ308992	
B*38:02:02	B*380202	B38(16)	—	GN00155, GN00416	U90240, U90241, AY094134, AY094135	
B*38:03	B*3803	B16	—	CTM-4786786	AF081275, AF081276	
B*38:04	B*3804	—	—	49-TA	AF181857, AF181858	
B*38:05	B*3805	B38(16)	B*38New	CTM-1095139	AF218802, AF218803, AF218804	
B*38:06	B*3806	—	—	GN00357, GN00372	AF262960, AF262961, AF282769, AF282770	
B*38:07	B*3807	—	B*3801New	MCB4	AF281053, AF281054	
B*38:08	B*3808	—	B*SSHAM	SSHAM	AF402320, AF402321	
B*38:09	B*3809	B16^c^	—	TER299	AJ507800	
B*38:10	B*3810	—	—	CBRL 9-23-217	AY601099, AY601100	
B*38:11	B*3811	—	—	NT00566, BY00261	AY956751, AY956753, EU185517	(147), CK Hurley^b^
B*38:12	B*3812	B38(16)	—	F409, F410	DQ239490, DQ239491, DQ239492	(198)
B*38:13	B*3813	—	—	NT00652	DQ436818, DQ436819	(149)
B*38:14	B*3814	—	—	UA-2005-TP-3911	AM261745	M Bengtsson
B*38:15	B*3815	B38(16)	—	VTIS146619	EF088203	BD Tait
B*38:16	B*3816	—	—	BT00710	EF375691	(85)
B*38:17	B*3817	—	—	G042406000536Z	EU030284	S Wallace
B*38:18	B*3818	—	—	SZ-17	FJ561482	(199)
B*38:19	B*3819	—	—	NT01025	FJ797377	CK Hurley
B*38:20	B*3820	—	—	HN-31268-7	FJ765853	Histogenetics
B*38:21	B*3821	—	—	HN-5161976	FJ765541	Histogenetics
B*38:22	B*3822	—	—	HN-732020	FJ468328	Histogenetics
B*39:01:01:01	B*39010101	B3901	B39.1, B16.2	S, JC	M94052, M29865, AB174781	M Satake^b^
B*39:01:01:02L	B*39010102L	Low B39(16)	—	SMF	AB091216	M Satake
B*39:01:03	B*390103	B3901	B39.1J	IT, #591	M94051, AB091218	
B*39:01:04	B*390104	B3901	B*39011New	NM4B380, JCB11331	AF165852, AF165853, AB032096	
B*39:01:05	B*390105	B3901	—	BY00306, HN-6381722	EU643610, FJ765837	(36), Histogenetics^b^
B*39:01:06	B*390106	B3901	—	1346	EU981815	J Liu
B*39:01:07	B*390107	B3901	—	HN-B-176320	FJ235053	Histogenetics
B*39:01:08	B*390108	B3901	—	HN-39295-3, HN-39417-3	FJ235056, FJ866167	Histogenetics
B*39:01:09	B*390109	B3901	—	HN-13530-5, HN-13531-3, HN-98310-6	FJ235065, FJ235066, FJ875580	Histogenetics
B*39:01:10	B*390110	B3901	—	HN-23000-4, HN-31356-8	FJ346315, GQ245750	Histogenetics
B*39:02:01	B*390201	B3902	B39.2	YAM	M94053	
B*39:02:02	B*390202	B3902	B39.2	CL170, NT01046	U04243, FJ797357	CK Hurley^b^
B*39:03	B*3903	B39(16)	—	AUCA#19, VTIS46155	L20088, AF387907, AF387908	
B*39:04	B*3904	B39(16)	B39N	TO ?KO, 13703390	L22649, AM085496	J Rowlands^b^
B*39:05:01	B*390501	B39(16)^c^	ST-16, B*39UW1, B*39JAI	11, HGOM, 12.35JK, 12.63JK, SZ-69	U15638, L36318, L36980, GQ161926	HY Zou^b^
B*39:05:02	B*390502	B39(16)	—	HN-3800450	FJ765561	Histogenetics
B*39:06:01	B*390601	B39(16)	B*39UW2	15, HAA, BA1, TER-102	U15639, L42024, L76640, L76639, U76396, U76397	
B*39:06:02	B*390602	B39(16)	B*39DBU, B39G	DBU, GVA, CVL, RD105, NAVAJO	U16298, L40562, U29083, U32660	
B*39:07	B*3907	—	B*39UW3	1276, NT01081	U15640, GQ251374	CK Hurley^b^
B*39:08	B*3908	B39(16)	—	822, NT00780	L42280, EU484050	CK Hurley^b^
B*39:09	B*3909	B39(16)	B39-143.2	143.2, XAV-50, 072	U29480, L76088, U90580	
B*39:10	B*3910	B39(16)	B39.ZU47	Zu47, GN00110, GB32, MA-31750	U56246, U52175, U52176, Y09058, AJ237703	
B*39:11	B*3911	B39(16)^c^	—	KUNA 20, NT00768	U74387, EU330468	CK Hurley^b^
B*39:12	B*3912	B39(16)	B-3901V	TER-103, BY00558	U76394, U76395, GU138068	CK Hurley^b^
B*39:13:01	B*391301	B39(16)	—	MCDS, C2035U	AJ223282, EU499386	FdP Sanchez Gordo^b^
B*39:13:02	B*391302	B39(16)	—	CTM2097181	AY973956	JL Vicario
B*39:14	B*3914	—	—	GN00217, BY00557	AF061867, AF061868, GU138069	CK Hurley^b^
B*39:15	B*3915	B39(16)^c^	—	178-260, BY00568	AF065640, AF065641, GU144507	CK Hurley^b^
B*39:16	B*3916	—	BA-39V	BAKA	AF098266, AF098267	
B*39:17	B*3917	—	B*39Var	010760981	AF110262, AF110263, AF110264	
B*39:18	B*3918	—	B*39011V	GN00310	AF173875, AF173876	
B*39:19:01	B*391901	B39(16)^c^	B*3901V	GN00293	AF176081, AF176082	
B*39:19:02	B*391902	B39(16)		BY00299, HN-49180-3, HN-1241139	EU555319, FJ594494, FJ875657	(36), Histogenetics^b^
B*39:20	B*3920	—	B*3910V	GN00317, BY00258	AF184216, AF184217, EU185520	CK Hurley^b^
B*39:22	B*3922	—	—	GN00332, scu00946m	AF205536, AF205537, EF679202	(139)^b^
B*39:23	B*3923	B39(16)	B3902V1	JCB12110, TBC62790	AB032097, AB285120	M Satske^b^
B*39:24	B*3924	B39(16)	B*CB2261, B*3903V	NDS-IH, CBu 10474, POHS-397, OC311, OC350, OC311, OC350, C183, CS00009	AF220288, AF220289, AF231101, AF231102, AF293020, AF293021, AF293022, AJ251768, AJ251769, AJ251768, AJ251769, AF428252, GQ260164	K Cao^b^
B*39:25N	B*3925N	Null	—	13W09502	AF363012, AF363013, AF363014	
B*39:26	B*3926	—	—	2000-333-343	AF408162, AF408163	
B*39:27	B*3927	B39(16)	—	MS24987, MS24990	AJ504798, AJ504799	
B*39:28	B*3928	—	—	149877	AJ575564	
B*39:29	B*3929	—	—	CBRL 9-78-156, BY00415	AY596777, AY596778, FJ619480	CK Hurley^b^
B*39:30	B*3930	—	—	CBRL 9-23-418	AY601101, AY601102	
B*39:31	B*3931	—	—	NT00509, VTIS125412, LUMC-B3, HN-37680-0, HN-37755-0, HN-12843-3, HN-15601-2, HN21782-2, HN-81285-3, HN-01934-3	AY607032, AY607033, AY829219, AY829220, AM746506, FJ765828, FJ765829, FJ594607, FJ594608, FJ594610, FJ868474, FJ868480	BD Tait^b^, JDH Anholts^b^, Histogenetics^b^
B*39:32	B*3932	—	—	BY00055	AY607030, AY607031	
B*39:33	B*3933	—	—	85921, NT01092	AY867868, GQ251354	MS Leffell, CK Hurley^b^
B*39:34	B*3934	B39(16)	—	CTM6681254	AY973955	JL Vicario
B*39:35	B*3935	—	—	BY00075	DQ167213, DQ167214	(149)
B*39:36	B*3936	—	—	CB9216	DQ242650, DQ242651, DQ242652	(200)
B*39:37	B*3937	—	—	IR2425, NT00712	AM114413, EF375694	K Lebeer, CK Hurley^b^
B*39:38Q	B*3938Q	—	—	BY00061, SZ-103	DQ105566, DQ105567, DQ105568, GQ161927	(88), HY Zou^b^
B*39:39	B*3939	—	—	NT00632, BY00262, CS00011	DQ334737, DQ334738, EU185516, GQ281055	(149), CK Hurley^b^, K Cao^b^
B*39:40N	B*3940N	Null	—	89951	DQ351214	MS Leffell
B*39:41	B*3941	B39(16)	—	TBC58854	AB248098	M Satake
B*39:42	B*3942	—	—	BJ038186	EF195124	X Shan
B*39:43	B*3943	—	—	LUMC-B10	AM748046	(64)
B*39:44	B*3944	—	—	JMDP36K0079	AB435079	K Tadokoro
B*39:45	B*3945	—	—	JMDP36K008	AB435164	K Tadokoro
B*39:46	B*3946	—	BJ057	BJ57	FJ358707	Z Zhang
B*39:47	B*3947	—	—	BY00417	FJ619478	CK Hurley
B*39:48	B*3948	—	—	BY00431	FJ688161	CK Hurley
B*39:49	B*3949	—	—	CG077022121801	FJ648688	H Hogan
B*39:50	B*3950	B39(16)	—	09000739	FJ785834	(127)
B*39:51	B*3951	—	—	HN-05052-5	FJ765547	Histogenetics
B*39:52	B*3952	—	—	HN-86891-0	FJ346291	Histogenetics
B*39:53	B*3953	—	—	HN-55241-4	FJ489874	Histogenetics
B*39:54	B*3954	—	—	BY00524	GQ867218	CK Hurley
B*39:55	B*3955	—	—	HN-1714885	FJ765826	Histogenetics
B*39:56	B*3956	—	—	HN-7445284	FJ392183	Histogenetics
B*40:01:01	B*400101	B60(40)	—	LB, 1510200307, SZ-73	P01890, U03698, L79937, GQ161930	HY Zou^b^
B*40:01:02	B*400102	B60(40)	B60Ut	Ut-m, JD, #W7079, MT214	M95530, L41628, EU233477	MGJ Tilanus^b^
B*40:01:03	B*400103	B60(40)	B*40(93090)	93090	AJ309573	
B*40:01:04	B*400104	B60(40)	—	112180, NT00567	AJ579628, AY935258, AY935259	CK Hurley^b^
B*40:01:05	B*400105	B60(40)	—	CBRL 9-55-277	AY598424, AY598425	
B*40:01:06	B*400106	B60(40)	—	BJ035	EF612726	Z Zhang
B*40:01:07	B*400107	B60(40)	—	HN-96363-7, HN-82956-3, HN-60049-0	FJ765545, FJ346262, FJ765978	Histogenetics
B*40:01:08	B*400108	B60(40)	—	HN-47393-6, HN-47058-2	FJ346307, FJ853782	Histogenetics
B*40:02:01	B*400201	B61(40)	B40*	SWEIG, CALOGERO, YUKI, 19014, TOB-105, MT456, ND1665	L09736, D14343, L76089, EU233475, EU233474	MGJ Zhang^b^, B Hepkema^b^
B*40:02:02	B*400202	B61(40)	B*4002V7	TBC47363	AB185101	
B*40:02:03	B*400203	B61(40)	—	Tw1	DQ096806, DQ096807, DQ096808	TD Lee
B*40:02:04	B*400204	B61(40)	—	BLA-B40	FM955318	(201)
B*40:02:05	B*400205	B61(40)	—	105479	FJ807734	MS Leffell
B*40:02:06	B*400206	B61(40)	—	HN-06317-3, HN-54215-6	FJ346312, FJ640580	Histogenetics
B*40:02:07	B*400207	B61(40)	—	HN-7668556, HN-N260568	FJ765787, GQ859532	Histogenetics
B*40:02:08	B*400208	B61(40)	—	HN-90110-6	FJ765982	Histogenetics
B*40:02:09	B*400209	B61(40)	—	JMDP36K061	AB536744	K Tadokoro
B*40:03	B*4003	B61(40)^c^	B40-G1	GRC138, SZ-88	M84383, GQ161928	HY Zou^b^
B*40:04	B*4004	B61(40)^c^	B40-G2	GRC212, TOB-0087	M84384, L76090	
B*40:05	B*4005	B4005	BN21	00136, NT01045	M84694, FJ797356	CK Hurley^b^
B*40:06:01:01	B*40060101	B61(40)	B61	Ot-s	M95531, AJ300180	
B*40:06:01:02	B*40060102	B61(40)	B*4006new	Terasaki EXT#58, MT193	AJ292253, EU233476	MGJ Tilanus^b^
B*40:06:02	B*400602	B61(40)	—	TBC50697	AB196427	M Satake
B*40:07	B*4007	B60(40)^c^	B’Fu’	MSU, FTA, KTA	D31816	
B*40:08	B*4008	—	—	4008, NT00779	L41353, EU484051	CK Hurley^b^
B*40:09	B*4009	B61(40)	B-4003V	PIL-117	L76934	
B*40:10	B*4010	B60(40)	B*40MD, B*40Var, B*40011Var, B40New	MD676, GN00160, 10PNG, PK, NMDP#019350966, TER1165, TER1166	U58643, U58644, U93915, U93916, Y15840, Y16636, Y16639, AF106628, AF106629, AJ580500, AJ580501, AJ580502	
B*40:11	B*4011	B61(40)^c^	B*40N	098, UCLA160, JMDP01K063	U75864, U75865, AF016299, AF009682, AB537164	K Tadokoro^b^
B*40:12	B*4012	—	B*40x15	TER-914, TE914, 015740137/467	Y13029, AF017334, AF017335, AF132492, AF132493, AF132494	
B*40:13	B*4013	—	—	NBER, NT01101	U96942, GQ251362	CK Hurley^b^
B*40:14:01	B*401401	B60(40)	—	104B	AF002274, AF017318	
B*40:14:02	B*401402	B60(40)	—	29567, 28967, 28503	AJ508389, AJ508390	
B*40:14:03	B*401403	B60(40)	—	010877, 11169540, D24298	AJ579654, AJ579655, AM114422, AM493904	J Rowlands^b^, O Avinens^b^
B*40:15	B*4015	—	—	M008B	AF002268, AF002269	
B*40:16	B*4016	B61(40)	—	EW, CS25, CS48, 98-00101	Y14606, AF017022, AF017023, AF027296, AF027297, AF110253, AF110254, AF110255	
B*40:18	B*4018	—	RN988B	RN988B	AF017332, AF017333	
B*40:19	B*4019	—	—	329-8016, JMDP01K017	AF065644, AF065645, AB435236	K Tadokoro^b^
B*40:20	B*4020	B61(40)^c^	—	290-596, 010818557	AF065648, AF065649, AF127812, AF127813, AF132017	
B*40:21	B*4021	—	B*15Var	CBP, #6749	AF106686, AF106687	
B*40:22N	B*4022N	Null	B40VN	40FC, KESSRo, RICHFr	AF129291, AF129292, AJ697852, AJ697853, AJ697854	
B*40:23	B*4023	—	B*40Var, B*CB2880	011743051, 702502, CB2880	AF129298, AF129299, AF132489, AJ278749, AJ278750, AF335312, AF335313	
B*40:24	B*4024	—	B*4018 Variant	GN00251	AF102573, AF102574	
B*40:25	B*4025	—	B*BM	BM1 131485	AF134864, AF134865	
B*40:26	B*4026	B21	B40Var	Akbasaim, BY00569	AJ243433, AJ243434, GU256002	CK Hurley^b^
B*40:27	B*4027	B61(40)	B*4002V1	JC12323, GN00316, CS00008	AB030575, AF181471, AF181472, GQ259735	K Cao^b^
B*40:28	B*4028	—	B*4004V	GN00313	AF181842, AF181843	
B*40:29	B*4029	B61(40)	B4002V2, B61v(40)	JC16904, JMDP36K042	AB032599, AB537165	K Tadokoro^b^
B*40:30	B*4030	—	B*40011V	GN00340, GN00352, GN00373	AF226840, AF226841, AF257507, AF257508, AF282767, AF282768	
B*40:31	B*4031	B60(40)	B*40RG	33692	AJ271160	
B*40:32	B*4032	—	B*4016V	GN00361	AF266523, AF266524	
B*40:33	B*4033	—	B*40011V	GN00369	AF279115, AF279116	
B*40:34	B*4034	B60(40)	B*40var	386619	AJ404846	
B*40:35	B*4035	B61(40)	—	ZFI	AJ290949, AJ290950	
B*40:36	B*4036	—	B*RRACH	RRACH	AY034093, AY034094	
B*40:37	B*4037	—	B*4002V	2000-343-446, 2000-343-785	AY034806, AY034807, AY042676, AY042677	
B*40:38	B*4038	—	—	VTIS39243, 45305	AF387901, AF387902, AJ966739	J Rowlands^b^
B*40:39	B*4039	B41	—	BUMC-40v	AY040540	
B*40:40	B*4040	—	—	BY0018, BY0025, BY0022, 32335, SZ-82	AY042680, AY042681, AY050193, AY050194, AY050189, AY050190, AJ629248, AJ629249, GQ161929	HY Zou^b^
B*40:42	B*4042	—	—	2000-350-252, NT00677	AF408164, AF408165, DQ984198	CK Hurley^b^
B*40:43	B*4043	—	—	BY00040	AF494281, AF494282	
B*40:44	B*4044	—	—	GN00417, BY00406	AY094136, AY094137, FJ619490	CK Hurley^b^
B*40:45	B*4045	—	—	GN00423	AY178187, AY178188	
B*40:46	B*4046	—	—	124427	AJ556550	
B*40:47	B*4047	B40	—	SF0001	AJ547815, AJ547816	
B*40:48	B*4048	B60(40)	—	R052, BY00076	AY297539, DQ244135, DQ244136	CK Hurley^b^
B*40:49	B*4049	—	—	TBC41776	AB174788, AB174789, AB174790	
B*40:50	B*4050	B61(40)	—	TBC42321, VTIS125430, HN-1893046	AB174888, AB174889, AB174890, AY829221, AY829222, FJ594729	BD Tait^b^, Histogenetics^b^
B*40:51	B*4051	—	—	HSR114063	AJ634528	
B*40:52	B*4052	B60(40)	B*4001V1	TBC154191, 35285	AB183463, AM050133	J Rowlands^b^
B*40:53	B*4053	B61(40)	B*4001V1	TBC13289	AB183464	
B*40:54	B*4054	B60(40)	B*4001V3	TBC45750, HN-0888695	AB185097, FJ594623	Histogenetics^b^
B*40:55	B*4055	—	B*4001V4	TBC46239	AB185098	
B*40:56	B*4056	B61(40)	B*4002V5	TBC46572	AB185099	
B*40:57	B*4057	—	B*4002V6	TBC46838	AB185100	
B*40:58	B*4058	—	—	TBC54006	AB213378	M Satake
B*40:59	B*4059	—	—	12323, BY00307	AY961619, EU643609	(202), CK Hurley^b^
B*40:60	B*4060	—	—	11763	AY961620	(203)
B*40:61	B*4061	—	—	HZB1538	DQ089628, DQ089629, DQ089630	(204)
B*40:62	B*4062	—	—	2005091432, 2005091665	DQ250652, DQ250653	OJ Kwon
B*40:63	B*4063	—	—	TBC56240, HN-22270-7	AB232526, FJ868481	M Satake, Histogenetics^b^
B*40:64	B*4064	B40	—	VTIS121219, BY00320, HN-21808-1, : HN-5544108, HN-6563529, HN-2672371	DQ400516, DQ500517, EU643596, FJ868462, FJ594576, FJ594595, FJ765809	BD Tait, CK Hurley^b^, Histogenetics^b^
B*40:65	B*4065	—	B*40MSR0206	MHHN-141191	AM233904	R Blasczyk
B*40:66	B*4066	—	—	CB4254	DQ181794	Y Li
B*40:67	B*4067	—	—	TBC35457	AB257503	M Satake
B*40:68	B*4068	—	—	TMO-399	DQ647703	(205)
B*40:69	B*4069	—	—	BY00135	DQ984197	(206)
B*40:70	B*4070	B61(40)	—	273	DQ914641	(207)
B*40:71	B*4071	—	—	KRC12507	EF363033	(208)
B*40:72:01	B*407201	—	—	TAKA8784AN	AM491776	AM Little
B*40:72:02	B*407202	—	—	JMDP01K015, HN-79169-1, HN-6736851	AB435235, FJ765849, FJ502332	K Tadokoro, Histogenetics^b^
B*40:73	B*4073	—	—	2006121483	EF447434	(209)
B*40:74	B*4074	—	—	P0233	EF458488	J Li
B*40:75	B*4075	—	—	BJ033, BJ039117	EF486279, EF195125	(210), X Shan^b^
B*40:76	B*4076	—	—	CTJ-14665	EF495153	(211)
B*40:77	B*4077	—	—	SCU00449	EF521874	(212)
B*40:78	B*4078	—	—	BJ037, HN-33851-9, HN-34166-1	EF679333, FJ600621, FJ600622	W Li, Histogenetics^b^
B*40:79	B*4079	—	—	RVEN	EU233478	B Hepkema
B*40:80	B*4080	—	—	NT00759, BY00276	EU275155, EU522474	(85), CK Hurley^b^
B*40:81	B*4081	—	—	ZHY-2	EU366958	(213)
B*40:82	B*4082	—	—	LUMC-B43	AM932286	(64)
B*40:83	B*4083	—	—	B07-2657	EU399238	(214)
B*40:84	B*4084	—	—	3.205E+11	EU481038	J He
B*40:85	B*4085	—	—	410028	EU434882	(215)
B*40:86	B*4086	—	—	SDBC-1	EDU434746	(216)
B*40:87	B*4087	—	—	BY00271, HN-3043303	EU522479, FJ875658	(36), Histogenetics^b^
B*40:88	B*4088	—	—	ZJCB5764	EU554563	(217)
B*40:89	B*4089	—	—	DEDKM740290	AM980943	A Dormoy
B*40:90	B*4090	—	—	BY00304, NT01027, HN-4038191, HN-732000, HN-8516105, HN-0761779, HN2057856, HN98841-0	EU643612, FJ973375, FJ235013, FJ765838, FJ765807, FJ765821, FJ875677, FJ858891	(36), CK Hurley^b^, Histogenetics^b^
B*40:91	B*4091	—	—	JMDP36K010	AB435080	K Tadokoro
B*40:92	B*4092	B40	—	46531	FM161906	J Rowlands
B*40:93	B*4093	—	—	RitNEW	EU7855971	(218)
B*40:94	B*4094	—	LUMC-B57	LUMC-B40, HN-84016-4, HN-12068-2, BY00508	FM945330, FJ600627, FJ765961, GQ410093	JDH Anholts, Histogenetics^b^, CK Hurley^b^
B*40:95	B*4095	—	—	BY00418	FJ649612	CK Hurley
B*40:96	B*4096	—	—	SDBC-HLA-2	FJ347890	(219)
B*40:97	B*4097	—	—	CG077022121965	FJ648687	H Hogan
B*40:98	B*4098	—	—	JRCKBCA7915	AB494701	Y Kuroda
B*40:99	B*4099	—	—	HN-0397427, BY00480	FJ235025, GQ410099	Histogenetics, CK Hurley
B*41:01	B*4101	B41	—	SGAR, CM4101, BM21, 1510200308	M24035, U90560, AJ309193	
B*41:02:01	B*410201	B41	B41.2	SBD4, GU5175, BM2684, 1510200309	X81363, U17572, X86704, AF126199	
B*41:02:02	B*410202	B41	—	HN-70545-3, HN-57639-1	FJ235051, FJ502330	Histogenetics
B*41:03:01	B*410301	B41^c^	—	GN00182, GN00245	AF028595, AF028596, AF102561, AF102562	
B*41:03:02	B*410302	B41	—	18428	EF467860	A Smith
B*41:04	B*4104	—	—	99126462S	AF258782	
B*41:05	B*4105	—	B*4101V	GN00370	AF279117, AF279118	
B*41:06	B*4106	—	—	UC-B434, 09-S-0029#0001	AJ308547, AY033291, AY033292	
B*41:07	B*4107	—	—	NT00568	AY935260, AY935261	(147)
B*41:08	B*4108	—	—	NT00564	DQ105579, DQ105580	(149)
B*41:09	B*4109	—	—	HN-47239-9, BY00537	FJ234992, GU066749	Histogenetics, CK Hurley^b^
B*41:10	B*4110	—	—	HN-25104-4, HN-90841-9, HN-56535-7, HN-53752431, HN-25103-6	FJ346256, FJ346290, FJ853779, FJ875578, FJ976769	Histogenetics
B*41:11	B*4111	—	—	HN-48401-6	FJ853798	Histogenetics
B*41:12	B*4112	—	—	HN-18541-2	FJ866150	Histogenetics
B*42:01:01	B*420101	B42	—	BB, BJ	M24034, AJ309194	
B*42:01:02	B*420102	B42	—	HN-3914882	FJ792532	Histogenetics
B*42:02	B*4202	B42	B42ANDO, 71B	E-117, E-119, 71B, 31-650, DZA9, SZ-87	D50709, U88249, AF017319, U88407, AJ002677, GQ161931	HY Zou^b^
B*42:04	B*4204	—	—	BY0027	AY050197, AY050198	
B*42:05:01	B*420501	—	—	BY00048, BY00436	AY217666, AY217667, FJ688156	CK Hurley^b^
B*42:05:02	B*420502	—	—	CZ-OL-DNA-10686	AJ716156, AJ716157, AJ716158	
B*42:06	B*4206	—	—	ESKOM105-368-99	AY621107	
B*42:07	B*4207	—	—	MHH0308010	AM040197	(220)
B*42:08	B*4208	—	—	LOFE36391AN	AM114034	AM Little
B*42:09	B*4209	—	—	NT00674, BY00308, HN-5428807	DQ832584, EU643608, FJ594721	(149), CK Hurley^b^, Histogenetics^b^
B*42:10	B*4210	—	—	BY00433, HN-647010, HN-21017-8	FJ688159, FJ765783, GQ468252	CK Hurley, Histogenetics^b^
B*42:11	B*4211			BY00465	FJ976694	CK Hurley
B*42:12	B*4212			60608C7	GQ250938	MdG Bicalho
B*42:13	B*4213			ZAFE568354AN	FN553434	SGE Marsh
B*44:02:01:01	B*44020101	B44(12)	B44.1, B44.2, B44021	FMB, BAU, RG-BR, SSTO	M24038, M15470, AJ309936, BX247310	S Beck^b^
B*44:02:01:02S	B*44020102S	—	—	PIO	AF384095	
B*44:02:02	B*440202	B44(12)	B*4402V	GN00350	AF253326, AF253327, AF386759	
B*44:02:03	B*440203	B44(12)	—	2000-238-831	AY034810, AY034811	
B*44:02:04	B*440204	B44(12)	—	NT00681	DQ987875	CK Hurley
B*44:02:05	B*440205	B44(12)	—	8/003970, HN-43200-6, HN-760344	FM174682, FJ346296, FJ502321	A Dormoy, Histogenetics^b^
B*44:02:06	B*440206	B44(12)	—	HN-20948-6, HN-N261934	FJ235015, GQ491085	Histogenetics
B*44:02:07	B*440207	B44(12)	—	HN-42052-0, HN-57175-4	FJ235024, FJ235035	Histogenetics
B*44:02:08	B*440208	B44(12)	—	NT01072	GQ251350	CK Hurley
B*44:02:09	B*440209	B44(12)	—	09210739, HN-96331-0	GQ375769, GQ245739	D Fuerst, Histogenetics^b^
B*44:02:10	B*440210	B44(12)	—	HN-70899-2, HN-347882, HN-50429-6	FJ392171, GQ859550, GU017931	Histogenetics
B*44:02:11	B*440211	B44(12)	—	HN-01937-9	FJ765963	Histogenetics
B*44:02:12	B*440212	B44(12)	—	HN-35487-4	FJ875569	Histogenetics
B*44:03:01	B*440301	B44(12)	B44.1:New	PITOUT, F24, MM44031, MANN	X64366, U90561, BX927178	S Beck^b^
B*44:03:02	B*440302	B44(12)	—	OBH, SHCHA, CAUC44032, SZ-100	L42282, U58469, U58470, AF056981, GQ161933	HY Zou^b^
B*44:03:03	B*440303	B44(12)	—	93455	EF215530	MS Leffell
B*44:03:04	B*440304	B44(12)	—	BY00472	GQ183832	CK Hurley
B*44:03:05	B*440305	B44(12)	—	HN-0312291	FJ234994	Histogenetics
B*44:03:06	B*440306	B44(12)	—	HN-16857-5	FJ346286	Histogenetics
B*44:03:07	B*440307	B44(12)	—	JMPD01K050	AB512681	K Tadokoro
B*44:03:08	B*440308	B44(12)	—	HN-9347914	FJ866175	Histogenetics
B*44:04	B*4404	B44(12)	B44.4	TAN, BEB, 10130882	X75953, X78426, X78427, AJ550735	
B*44:05:01	B*440501	B44(12)	B44WJG, B44KB	WJG, KB, 14-AS-0013#0001, HSV33, E840, 78502, T283	X78849, X78850, L31798, AF288472, AF288473, AJ535113, AY188943, AY188944, AY188945, AY194116, AY305860	
B*44:05:02	B*440502	B44(12)	—	HN-78228-0	FJ875553	Histogenetics
B*44:06	B*4406	B44(12)	—	GIJM, KARY	X83400, X83401-3, L42345	
B*44:07	B*4407	B44(12)	B*44GB	GB92, NT01091	X90391, GQ251353	CK Hurley^b^
B*44:08	B*4408	B44(12)	B44bo, B*44DM	19662, DM	U64801, AJ132659, AJ132660	
B*44:09	B*4409	B45(12)^c^	B4409	S.A., RG-BR	X99734, AJ309937	
B*44:10	B*4410	B44(12)^c^	—	S32, NT00695	U63559, U63560, EF156375	CK Hurley^b^
B*44:11	B*4411	—	—	GN00220	AF071767, AF071768	
B*44:12	B*4412	B44(12)	B*4402Var	MOV002AN	AJ133267	
B*44:13	B*4413	B44(12)	B*44New1	AMI005AN, 445/01	AJ131118, AJ749639, AJ749640	
B*44:14	B*4414	B44(12)	B44IP	IP	AJ238702	
B*44:15	B*4415	B12	B45New, B*45V	ML1805, 3880, SMN44	AJ133471, AJ133472, AJ251766, AJ251767, AF215918, AF215919	
B*44:16	B*4416	B47	B*4402New	10000009, TER1017	AF190446, AF190447, AJ583160, AJ583161, AJ583162	
B*44:17	B*4417	B44(12)	B*44SR	B1268	AJ249724, AJ249725	
B*44:18	B*4418	—	—	99-2201, BY00554	AF190275, AF190276, AF190277, GU138072	CK Hurley^b^
B*44:19N	B*4419N	Null	B44N	ALBA	AJ251593	
B*44:20	B*4420	—	—	GN00331, 28007	AF205534, AF205535, AJ697949, AJ697950	
B*44:21	B*4421	—	B*TBAL	GN00333, TBAL, Scu03903	AF205538, AF205539, AF231098, AF231099, EU499387	FdP Sanchez Gordo^b^
B*44:22	B*4422	—	—	15-S-0032#0102, NT00751	AY003906, AY003907, EU256487	CK Hurley^b^
B*44:23N	B*4423N	Null	B*44MP	12506397, FH33, 108931180	AJ278766, AJ295293, AF363681, AF363682, AF363683, AJ580409	
B*44:24	B*4424	—	—	GN00383, NT01079	AF310140, AF310141, GQ251372	CK Hurley^b^
B*44:25	B*4425	—	B*CB2913	CB2913	AF335308, AF335309	
B*44:26	B*4426	—	—	MCH48	AF349440	
B*44:27	B*4427	B44(12)	—	E487, FH50, FH48, 28008, CS00017	AF329843, AF329845, AF419293, AF419294, AF419295, AJ783756, GQ301203	K Cao^b^
B*44:28:01	B*442801	—	—	GN00396, GN00397	AY050199, AY050200, AY050201, AY050202	
B*44:28:02	B*442802	—	—	MHHAKB-518404, MHHAKB-518813, HN-97655-4	AM920502, AM920465, FJ594495	P Horn, R Blasczyk, Histogenetics^b^
B*44:29	B*4429	B44(12)^c^	—	GN00406, VTIS125413	AY050212, AY050213, AY829223, AY829224	BD Tait^b^
B*44:30	B*4430	—	—	2000-301-424, BY00438	AF408158, AF408159, FJ688154	CK Hurley^b^
B*44:31	B*4431	B44(12)	—	AKAR	AJ297942, AJ297043	
B*44:32	B*4432	B44(12)	—	VBD25061	AY057404, AY057405	
B*44:33	B*4433	—	—	VTIS70964CG	AY170308, AY170309	
B*44:34	B*4434	—	—	VTIS33455	AY208895, AY208896	
B*44:35	B*4435	—	—	LB66779	AJ539145, AJ539146	
B*44:36	B*4436	—	—	A4599	AY330329, AY330330	
B*44:37	B*4437	—	—	143871	AJ575563	
B*44:38	B*4438	—	—	6B137	AY351272, AY351273	
B*44:39	B*4439	—	B*44SAIN	SAIN	AJ579712, AJ579713, AJ579714	
B*44:40	B*4440	B44(12)	—	2004-04650	AJ784155	
B*44:41	B*4441	—	—	AKB23061, AKB23116, AKB23704	AJ867241	(221)
B*44:42	B*4442	B21	—	CZ-OL-DNA-10909, BY00380	AJ937958, FJ464342	F Mrazek, CK Hurley^b^
B*44:43	B*4443	B44(12)	—	HFT310804, HFT271204	AY988108	(222)
B*44:44	B*4444	—	—	NT00607	DQ120788, DQ120789	(149)
B*44:45	B*4445	—	—	Z-00018416	AM050158	R Blasczyk
B*44:46	B*4446	—	—	5089, JUCH70904AN	DQ185396, AM236591	(223), AM Little^b^
B*44:47	B*4447	—	—	259133	DQ343759	K Hirv
B*44:48	B*4448	—	—	NT00676	DQ832589	(149)
B*44:49	B*4449	—	—	K101008	AM396519	C Dunne
B*44:50	B*4450	—	—	HM-7755(LAS), BY00294	EF025768, EU555323	(111), CK Hurley^b^
B*44:51	B*4451	—	—	92637	EF057103	MS Leffell
B*44:52N	B*4452N	Null	—	1028911	EF199623	D Smillie
B*44:53	B*4453	B44(12)	—	200700049, HN-1660955	AM597557, FJ594555	(224), Histogenetics^b^
B*44:54	B*4454	B44(12)	—	Y40482	EF545134	M Christiansen
B*44:55	B*4455	B44(12)	—	R37148	AM746211	(44)
B*44:56N	B*4456N	Null	—	4Bons	AM850137	(225)
B*44:57	B*4457	—	—	104798, HN-20013-0, HN-59675-0	AM850144, FJ594501, FJ594564	J Enczmann, Histogenetics^b^
B*44:58N	B*4458N	Null	—	BY00266	EU256486	(85)
B*44:59	B*4459	B44(12)	—	Cairjo	AM922196	(226)
B*44:60	B*4460	—	—	BY00274, BY00441	EU522476, FJ688151	(36), CK Hurley^b^
B*44:61N	B*4461N	Null	—	JMDP01K018	AB435544	K Tadokoro
B*44:62	B*4462	—	—	BY00323, BY00414, HN-21289-2	EU682449, FJ619481, FJ346237	CK Hurley, Histogenetics^b^
B*44:63	B*4463	—	—	BY00324	EU682448	CK Hurley
B*44:6401	B*446401	—	—	BY00322	EU682450	CK Hurley
B*44:6402	B*446402	—	—	RIFFMic, HN-88822-1, HN43583-4, HN-59795-1, HN-66408-5	FM160944, FJ346243, FJ346263, FJ765976, FJ853776	A Dormoy, Histogenetics^b^
B*44:65	B*4465	—	—	103013, BY00510	FJ231109, GQ410095	MS Leffell, CK Hurley^b^
B*44:66	B*4466	—	—	C140734	FJ600545	(227)
B*44:67	B*4467	—	—	BY00420	FJ649610	CK Hurley
B*44:68	B*4468	—	—	HN-41637-0, HN-02637-6, HN-07009-3	FJ502323, FJ765543, FJ765544	Histogenetics
B*44:69	B*4469	—	—	NT01026, HN-743688	FJ797376, FJ489873	CK Hurley, Histogenetics^b^
B*44:70	B*4470	—	—	HN-66349-2	FJ234990	Histogenetics
B*44:71	B*4471	—	—	HN-94480-0	FJ235003	Histogenetics
B*44:72	B*4472	—	—	HN-3124406	FJ235031	Histogenetics
B*44:73	B*4473	—	—	HN-38150-0, HN-22849-5	FJ235038, FJ346038	Histogenetics
B*44:74	B*4474	—	—	HN-56735-9, HN-59941-4, HN-56314-7, HN-62281-0, HN-0696372, HN-67794-6	FJ235044, FJ346245, FJ853778, FJ866151, FJ392163, GQ254352	Histogenetics
B*44:75	B*4475	—	—	HN-78758-4	FJ235057	Histogenetics
B*44:76	B*4476	—	—	HN-113911, BY00483	FJ235073, GQ410102	Histogenetics, CK Hurley^b^
B*44:77	B*4477	—	—	HN-11862-8	FJ765864	Histogenetics
B*44:78	B*4478	—	—	HN-04455-0	FJ346238	Histogenetics
B*44:79	B*4479	—	—	HN-98639-7, HN-79306-6, HN-32671-9, HN-79445-2	FJ346239, FJ346240, FJ346241, FJ853774	Histogenetics
B*44:80	B*4480	—	—	HN-55123-4	FJ346258	Histogenetics
B*44:81	B*4481	—	—	JMDO01K051	AB512682	K Tadokoro
B*44:82	B*4482	—	—	BY00477	GQ410096	CK Hurley
B*44:83	B*4483	—	—	192434	FM886991	K Witter
B*44:84	B*4484	—	—	HN-05339-8	FJ346311	Histogenetics
B*44:85	B*4485	—	—	HN-3345779	FJ392166	Histogenetics
B*44:86	B*4486	—	—	HN-87936-3, HN-54829-3, HN-41994-1	FJ594720, FJ875566, GQ254335	Histogenetics
B*44:87	B*4487	—	—	HN-3239576	FJ765833	Histogenetics
B*44:88	B*4488	—	—	HN-05870-9	FJ853771	Histogenetics
B*44:89	B*4489	—	—	HN-77525-6	FJ866161	Histogenetics
B*44:90	B*4490	—	—	HN-07064-2	FJ875671	Histogenetics
B*44:91	B*4491	—	—	HN-4002229	FJ765774	Histogenetics
B*44:92	B*4492	—	—	HN-48769-2	FJ765839, FJ765983	Histogenetics
B*44:93	B*4493	—	—	HN-4857960	FJ765867	Histogenetics
B*44:94	B*4494	—	—	HN-5715940	FJ765930	Histogenetics
B*44:95	B*4495	—	—	HN-33336-6	FJ765966	Histogenetics
B*44:96	B*4496	—	—	HN-33937-0, HN-33582-4	FJ765975, GQ254349	Histogenetics
B*44:97	B*4497	—	—	HN-60214-0, HN-60432-8	FJ765979, FJ765980	Histogenetics
B*44:98	B*4498	—	—	HN-74975-2	FJ765981	Histogenetics
B*44:99	B*4499	—	—	HN-6578988	FJ858898	Histogenetics
B*45:01	B*4501	B45(12)	—	OMW, CM4501, 1510200310, CU45A	X61710, U90562, AJ458992, EF203079	(160)^b^
B*45:02	B*4502	—	—	GN00214	AF061861, AF061862	
B*45:03	B*4503	—	B*4501New	O3499	AJ275937	
B*45:04	B*4504	—	—	PMF	AJ278944	
B*45:05	B*4505	—	—	GN00387	AY016213, AY016214	
B*45:06	B*4506	—	—	013969175	AF469652, AF469653	
B*45:07	B*4507	—	—	ESKOM105-530-33	AY619997	
B*45:08	B*4508	—	—	BY00273	EU522477	(36)
B*45:09	B*4509	—	—	MO-19507	EU517717	G Rampim
B*45:10	B*4510	—	—	HN-18691-0	FJ235068	Histogenetics
B*45:11	B*4511	—	—	HN-8041258	FJ594700	Histogenetics
B*46:01:01	B*460101	B46	—	T7527, THAI742, T7526	M24033, AJ310508	
B*46:01:02	B*460102	B46	—	K9505035	DQ985229	(228)
B*46:01:03	B*460103	B46	—	HN-3681660	FJ765777	Histogenetics
B*46:02	B*4602	B46	B46V1	JCB15113	AB032091	
B*46:03	B*4603	B46	B*4601V2	TBC33457	AB183519	
B*46:04	B*4604	B46	B*4601V3	TBC37863	AB183520	
B*46:05	B*4605	B46	—	TBC54558	AB213260	M Satake
B*46:06	B*4606	—	—	BY00064	DQ105575, DQ105576, DQ105577, DQ105578	(88)
B*46:07N	B*4607N	Null	—	BY00062	DQ105569, DQ105570, DQ105571	(88)
B*46:08	B*4608	—	—	HZ7551	DQ177521, DQ177522, DQ177523	(229)
B*46:09	B*4609	—	—	32094	EF121373	(230)
B*46:10	B*4610	—	—	NT00708	EF375698	(85)
B*46:11	B*4611	—	—	51348043, BY00440	EF419280, FJ688152	W Dong, CK Hurley^b^
B*46:12	B*4612	—	—	B13403	EU081878	(173)
B*46:13:01	B*461301	—	—	B19205	EU515127	(231)
B*46:13:02	B*461302	—	—	BY00397	FJ619497	CK Hurley
B*46:14	B*4614	—	—	ZJCB5857	EU554564	(231)
B*46:15N	B*4615N	Null	—	JMDP01K019	AB435545	K Tadokoro
B*46:16	B*4616	—	—	JMDP-1K040	AB436533	K Tadokoro
B*46:17	B*4617	—	—	34151259a	EU871946	(232)
B*46:18	B*4618	—	—	B18939	FJ222391	(231)
B*46:19	B*4619	—	—	WangweiB46	FJ810061	J He
B*46:20	B*4620	—	—	HN-4246760	FJ234997	Histogenetics
B*46:21	B*4621	—	—	SZGSQ-3	GQ225741	(233)
B*46:22	B*4622	—	—	HN-39288-6, BY00522	FJ866149, GQ867216	Histogenetics, CK Hurley
B*47:01:01:01	B*47010101	B47	—	PLH	M19756, AJ295141	
B*47:01:01:02	B*47010102	B47	—	383008	AJ308398	
B*47:02	B*4702	B47	—	CAL	Y09118	
B*47:03	B*4703	B47	B*47RG, B*47TAIB	DT-32, 29182, TAIB, GN00218, VELT	AF016842, AF016843, Y17193, Y19194, AJ006978, AF071763, AF071764, AJ251003	
B*47:04	B*4704	—	—	05-S-0012#1001	AY033293, AY033294	
B*47:05	B*4705	—	—	m11546, m11673, m11362	AY640119	
B*48:01:01	B*480101	B48	—	KRC103, HS67, CM4801, 26/27	M84380, U66250, AJ309139	
B*48:01:02	B*480102	B48	—	AB896	EF059810	(234)
B*48:02	B*4802	B48	—	AUCA#18, C211	L20089, AJ556172	
B*48:03:01	B*480301	B48^c^	B-48.3	TOB-115, JMDP36K028, SZ-81	L76931, AB436626, GQ161935	K Tadokoro^b^, HY Zou^b^
B*48:03:02	B*480302	B48^c^	—	BY00137, BY00509, HN-1833602, BY00529	EF078989, GQ410094, FJ594559, GU066741	(149), CK Hurley^b^, Histogenetics^b^
B*48:04	B*4804	B48	0328	0328, JC20008, SZ-84	AF017328, AF017329, AB063626, AB063627, AB063628, GQ161934	HY Zou^b^
B*48:05	B*4805	B48	B*40Var	GLAD, 011837630/48	AF096631, AF096632, AF127805, AF129293, AF132490	
B*48:06	B*4806	—	B*4801Variant	234-01069	AF108426, AF108427	
B*48:07	B*4807	B48	B*4801Var	30007, GN00258	AF136393, AF136394, AF135538, AF135539	
B*48:08	B*4808	—	—	Jadob	AJ566211	
B*48:09	B*4809	B48	B*4801V1	TBCT3625	AB183521	
B*48:10	B*4810	B48	B*4801V2	TBCB46130	AB183522	
B*48:11	B*4811	—	—	2004112378	AY879267, AY879268	(235)
B*48:12	B*4812	—	—	NT00521, NT00569, 41697S, 41698S, HN-9904128, HN-2579560	AY874083, AY874084, AY956750, AY956751, AY686614, AY686615, FJ594601, FJ594603	(147), S Adams^b^, Histogenetics^b^
B*48:13	B*4813	—	—	40390S	AY686613	S Adams
B*48:14	B*4814	—	—	Xian4807	DQ238864, DQ238865	(236)
B*48:15	B*4815	—	—	NT00654	DQ436816, DQ436817	(149)
B*48:16	B*4816	—	—	BY00083	DQ455014	(57)
B*48:17	B*4817	B48/40V	—	K13577, K13578	AB292739	E Maruya
B*48:18	B*4818	—	—	20060332CB, 2003032mother	AB303948	H Inoko
B*48:19	B*4819	—	—	BJ043	EU022558	W Li
B*48:20	B*4820	—	—	HN-7740058	FJ866146	Histogenetics
B*48:21	B*4821	—	—	HN-4187485	FJ494825	Histogenetics
B*49:01:01	B*490101	B49(21)	—	AM, GU2092	M24037, U11263, AJ311600	
B*49:01:02	B*490102	B49(21)	—	NT01007	EU924804	CK Hurley
B*49:02	B*4902	B49(21)	B*4901V	MC2918, GN00358	AJ269496, AJ269497, AJ269498, AF262958, AF262959	
B*49:03	B*4903	—	B*RA	29037	AJ288980	
B*49:04	B*4904	—	—	14319	AJ969238	(237)
B*49:05	B*4905	B49(21)	—	147554	AM076839	(238)
B*49:06	B*4906	—	—	HN-02488-5	FJ346280	Histogenetics
B*49:07	B*4907	B47	—	CTM-8004574	GQ454860	(239)
B*49:08	B*4908	—	—	HN-32306-0	FJ765964	Histogenetics
B*49:09	B*4909	—	—	HN-50261-4	FJ765969	Histogenetics
B*50:01:01	B*500101	B50(21)	—	SH.JO, JD, GU2037, PAT541	X61706, U11261, DQ249182	(141)^b^
B*50:01:02	B*500102	B50(21)	—	HN-70644-0	FJ346283	Histogenetics^b^
B*50:02	B*5002	B45(12)	B*50IM, B*45v, B*45ZJ	IMM754, WM1366C, CTM-1983039, GN00173, UBM13129406	U58317, U58318, Y08995, AF006634, AF008926, AF008927, Y14205	
B*50:04	B*5004	B50(21)	—	3011	AF136397, AF136398	
B*50:05	B*5005	—	—	HN-04738-5	FJ235064	Histogenetics
B*50:06	B*5006	—	—	HN-74749-4	FJ346303	Histogenetics
B*50:07	B*5007	—	—	HN-05684-7	FJ346322	Histogenetics
B*50:08	B*5008	—	—	HN-27100-9, HN-85701-2, HN-72963-5	FJ346325, FJ866152, FJ765847	Histogenetics
B*50:09	B*5009	—	—	HN-44810-2, HN-58062-4, HN-44735728	FJ853801, FJ765845, FJ765957	Histogenetics
B*51:01:01	B*510101	B51(5)	—	LKT-2, TO, BM92, CD, LCL721, KRC110, KRC005, BA1, BA6, 1510200303	M32319, M22786, M22787-M22788, M28205, Z46808, L47985, L77204, AJ608261, AJ608262	
B*51:01:02	B*510102	B51(5)	B*51V	GN00106, 12WDCH010, 12WDCH028, UCB-1999-163	U52169, U52170, U90611, U90612, U90613, U90614, AJ278903	
B*51:01:03	B*510103	B51(5)	B*51011V	GN00264	AF135550, AF135551	
B*51:01:04	B*510104	B51(5)	—	DLM, NT01093	AJ249937, AJ249938, AJ505554, AJ505555, GQ251355	CK Hurley^b^
B*51:01:05	B*510105	B51(5)	—	MS22035	AJ426462, AJ426465, AJ426466, AJ426463, AJ426464	
B*51:01:06	B*510106	B51(5)	—	NT00525	AY877251, AY877252	(147)
B*51:01:07	B*510107	B51(5)	—	87271	DQ072941	MS Leffell
B*51:01:08	B*510108	B51(5)	—	Shani004	EF542833	D Zhang
B*51:01:09	B*510109	B51(5)	—	BY00302, HN-53665-6, HN-3535353, HN-4843659, HN-0728433, HN-0609201, HN-1575023	EU555316, FJ594599, FJ594554, FJ594598, FJ594602, FJ600620, FJ875660	CK Hurley, Histogenetics^b^
B*51:01:10	B*510110	B51(5)	—	HN-16784-0, HN-61488-2, HN-27714-4, HN-73186-2	FJ346266, FJ853775, FJ866147, GQ401191	Histogenetics
B*51:01:11	B*510111	B51(5)	—	HN-80470-2	FJ594719	Histogenetics
B*51:01:12	B*510112	B51(5)	—	HN-07547-1	FJ494831	Histogenetics
B*51:01:13	B*510113	B51(5)	—	HN-48106-0	FJ853797	Histogenetics
B*51:01:14	B*510114	B51(5)	—	HN-1575924	FJ765785	Histogenetics
B*51:01:15	B*510115	B51(5)	—	HN-5571489	FJ765836	Histogenetics
B*51:01:16	B*510116	B51(5)	—	HN-41340-7	FJ796990	Histogenetics
B*51:02:01	B*510201	B5102	B5.35	UM, 02627	M68964	
B*51:02:02	B*510202	B5102	—	MY823, 12WDCH011	L41925, U90615, U90616	
B*51:02:03	B*510203	B5102	—	B16573	EF611989	(240)
B*51:02:04	B*510204	B5102	—	HENAN12	EU785342	B Zhang
B*51:03	B*5103	B5103	BTA	30-BY3	M80670	
B*51:04	B*5104	B51(5)^c^	—	GRC150	Z15143	
B*51:05	B*5105	B51(5)	B51v	LK, 10030381	U06697, AJ297934	
B*51:06	B*5106	B51(5)^c^	—	GN097, GN088, 29130, TBC60704	U31334, U32661, AJ511650, AJ511651, AB274956	M Satake^b^
B*51:07	B*5107	B51(5)	B5101v	RCE55, TBC46697, SZ-68	X94481, AB185102, GQ161936	HY Zou^b^
B*51:08	B*5108	B51(5)	B*51FA, B*51GAC	F.A., GN00109, NDS-DG, AS7235	X96473, U52815, U52816, Y08994, Y10031, Y11228, Y11229	
B*51:09:01	B*510901	B51(5)	B*51IM, B*51N	IMM721, NMDP-0004, RN285B, GN00178, GN00205, GN00204, NM4B437	U58319, U58320, U76400, U76401, AF002272, AF017320, AF028599, AF028600, AF054001, AF054002, AF165848, AF165849	
B*51:09:02	B*510902	B51(5)	—	NT00701, BY00409	EF195108, FJ619487	(85), CK Hurley^b^
B*51:10	B*5110	—	HLA-B*51like, B-51v	KUNA 14, 009041674	AF004370, AF056479, AF056480	
B*51:11N	B*5111N	Null	B*51N	HGW6178	Y13566	
B*51:12	B*5112	—	B51Va	RTCV	AF023442, AF023443	
B*51:13:01	B*511301	—	B*51vK60	K60, NT01087	AJ002151, GQ251380	CK Hurley^b^
B*51:13:02	B*511302	—	B*51011V	GN00140, NT01077	AF135534, AF135535, GQ251370	CK Hurley^b^
B*51:14	B*5114	B51(5)^c^	—	GN00207, GN00208	AF054005, AF054006, AF054007, AF054008	
B*51:15	B*5115	—	—	GN00183	AF072445, AF072446	
B*51:16	B*5116	B5	DT51v	DTEC	AF098264, AF098265	
B*51:17	B*5117	B51(5)	—	3010	AF136395, AF136396	
B*51:18	B*5118	B51(5)	B*51New	MEFG	AJ133773, AJ133814	
B*51:19	B*5119	—	—	TN01/1210, NT01074, NT00473	AJ238971, AJ238972, GQ251367, GQ251366	CK Hurley^b^
B*51:20	B*5120	—	B*5108V	GN00285, NT00709	AF140861, AF140862, EF375697	CK Hurley^b^
B*51:21	B*5121	—	B*51011V	GN291	AF176079, AF176080	
B*51:22	B*5122	—	B*51011V	GN00349, GN00355, NT00711a	AF248061, AF248062, AF260975, AF260976, EF375685	CK Hurley^b^
B*51:23	B*5123	—	B*5102V	GN00342, NT01100	AF226844, AF226845, GQ251361	CK Hurley^b^
B*51:24:01	B*512401	B51(5)	B*51New	46643, QC19-7	AJ276995, AJ504400, AJ504401	
B*51:24:02	B*512402	B51(5)	—	Jul-51	EU547797	(241)
B*51:24:03	B*512403	B51(5)	—	HN-30281-0	FJ765860	Histogenetics
B*51:26	B*5126	—	—	GN00385, 33327	AY016209, AY016210, AJ829726	
B*51:27N	B*5127N	Null	—	5761	AF363789, AF363790	
B*51:28	B*5128	B51(5)	—	VTIS40888	AY057400, AY057401	
B*51:29	B*5129	B51(5)	—	FH59, FH38	AY056451, AY056452, AY056453	
B*51:30	B*5130	—	—	CPH-1	AY102648	
B*51:31	B*5131	B51(5)	B*5116V1	TBC-T5139	AB087515, AB087516	
B*51:32	B*5132	—	B*51INA-FA	INA-FA	AJ506045, AJ506052, AJ506054	
B*51:33	B*5133	—	—	R209312	AJ507649, AJ507650	
B*51:34	B*5134	—	—	0427-7059-4, NT01089	AJ507653, AJ507654, GQ251351	CK Hurley^b^
B*51:35	B*5135	B51(5)	B*5101V1	TBC45686	AB183523	
B*51:36	B*5136	—	—	HZCB1958, BY00122, BY00133	AY601729, AY601730, AY601731, DQ832586, DQ924382	CK Hurley^b^
B*51:37	B*5137	—	—	CH03112415, MP3583	AY781783, AY781784	
B*51:38	B*5138	—	—	CB12239, HN-51268-5, HN-29132-2	AY957951, AY957952, FJ594613, FJ868488	S Davey, Histogenetics^b^
B*51:39	B*5139	—	—	BY00063	DQ105572, DQ105573, DQ105574	(88)
B*51:40	B*5140	—	—	NT00630, BY00289	DQ334733, DQ334734, EU555327	(149), CK Hurley^b^
B*51:41N	B*5141N	Null	—	TBC583362	AB247569	M Satake
B*51:42	B*5142	?B44(12)	—	604706	AM260212	M Bengtsson
B*51:43	B*5143	B51(5)	—	73626, NT00766, HN-62470-2, HN-30929-6, HN-4434327, HN-8803-1, HN-30931-2	DQ834251, EU330465, FJ594584, FJ594578, FJ594578, FJ594724, FJ853764, FJ868471	MS Leffell, CK Hurley^b^, Histogenetics^b^
B*51:44N	B*5144N	Null	—	92426	DQ902553	MS Leffell
B*51:45	B*5145	—	—	BY00130	DQ924380	CK Hurley
B*51:46	B*5146	B51(5)	—	142253	DQ885884	K Hirv
B*51:48	B*5148	—	—	BY00150	EF484937	(85)
B*51:49	B*5149	—	—	C138639	EF540341	(242)
B*51:50	B*5150	—	—	49916, HN-98621-5, HN-19150-3, HN-60266-4, HN-77067-8, HN-08720-7, HN-63495-9, HN-44692-4	AM779473, FJ594496, FJ594575, FJ594500, FJ594556, FJ594726, FJ600619, FJ594571	J Enczmann, Histogenetics^b^
B*51:51	B*5151	—	—	LUMC-B32	AM849815	(64)
B*51:52	B*5152	—	—	MHHI-603335	AM906166	R Blasczyk
B*51:53	B*5153	—	—	BY00297	EU555321	(36)
B*51:54	B*5154	—	—	BY00291	EU555326	(36)
B*51:55	B*5155	—	—	BY00287, BY00290, BY00341, HN-21745-9	EU555329, EU557368, EU826135, FJ868479	(36), Histogenetics^b^
B*51:56	B*5156	—	—	BY00305, HN-61747-2	EU643611, FJ765948	(36), Histogenetics^b^
B*51:57	B*5157	—	—	JMDP36K011	AB435165	K Tadokoro
B*51:58	B*5158	—	—	Allcord2423	EU839991	(243)
B*51:59	B*5159	—	—	LuluX01	EU871625	(244)
B*51:60	B*5160	—	—	NT01010	EU924800	CK Hurley
B*51:61	B*5161	—	—	DEDKM2710684, HN-17776-5, HN-25153-7, HN-17770-1	AM493681, FJ346265, FJ853786, FJ866166	(176), Histogenetics^b^
B*51:62	B*5162	—	—	08002710A	EU881364	DK Agbley
B*51:63	B*5163	—	—	CTJ-21971	FJ200656	L Yan
B*51:64	B*5164	—	—	BY00393	FJ619501	CK Hurley
B*51:65	B*5165	—	—	BY00402, HN-7648279, HN-16760-2, HN-40799-5, 267175, HN-N94065, HN-25984-8, HN-N262510, HN-N035688, HN-N261989, HN-N262508	FJ619493, FJ765929, FJ765852, FJ765952, FN422394, GQ245728, GQ449634, GQ859535, GQ914788, GQ914790, GQ914791	CK Hurley, Histogenetics^b^, T Lebedeva^b^
B*51:66	B*5166	—	—	HN-04656-4	FJ346274	Histogenetics
B*51:67	B*5167	—	—	HN-98920-5, HN-90439-1, HN-75991-0	FJ392174, FJ858890, GQ245733	Histogenetics
B*51:68	B*5168	—	—	HN-13359-6, HN-62842-2	FJ392177, GQ914793	Histogenetics
B*51:69	B*5169	—	—	HN-84899-5, HN-98901-1, HN-47753-6, HN-77817-6	FJ392179, FJ346235, FJ765968, GQ240391	Histogenetics
B*51:70	B*5170	—	—	HN-83758-5	FJ502327	Histogenetics
B*51:71	B*5171	—	—	HN-64733-9	FJ234988	Histogenetics
B*51:72	B*5172	—	—	HN-04865-0	FJ235007	Histogenetics
B*51:73	B*5173	—	—	HN-2357049	FJ235030	Histogenetics
B*51:74	B*5174	—	—	HN-78085-2	FJ235058	Histogenetics
B*51:75	B*5175	—	—	HN-58094-4, HN-2234081, HN-83234-7, HN-6573599, HN-0092779, HN-N257741, HN-N263825, HN-N034218	FJ765862, FJ765832, FJ765850, FJ765869, FJ792533, GQ468247, GQ859531, GQ994058	Histogenetics
B*51:76	B*5176	—	—	HN-15659-6	FJ346285	Histogenetics
B*51:77	B*5177	—	—	HN-98465-9	FJ346288	Histogenetics
B*51:78	B*5178	—	—	JMDP36K038, AN188087	AB512683, FN555006	K Tadokoro, F Poli^b^
B*51:79	B*5179	—	—	HN-94220-9, HN-94235-7	FJ468321, FJ468322	Histogenetics
B*51:80	B*5180	—	—	HN-57299-0	FJ494829	Histogenetics
B*51:81	B*5181	—	—	HN-63937-1	FJ765841	Histogenetics
B*51:82	B*5182	—	—	HN-96919-5, HN-91414-5, HN-17946-0, HN-17947-8	FJ853785, FJ765574, GQ449643, GQ449644	Histogenetics
B*51:83	B*5183	—	—	HN-45771-5	FJ866168	Histogenetics
B*51:84	B*5184	—	—	CMV-N10	EF450253	M Lin
B*51:85	B*5185	—	—	HN-1736219	FJ765569	Histogenetics
B*51:86	B*5186	—	—	HN-3623019	FJ765793	Histogenetics
B*51:87	B*5187	—	—	HN-12571-3	FJ640578	Histogenetics
B*51:88	B*5188	—	—	HN-44579-8	FJ765967	Histogenetics
B*51:89	B*5189	—	—	JMDP36K062	AB536745	K Tadokoro
B*52:01:01	B*520101	B52(5)	—	MT, LK707, E4181324, SZ-75	M22793-9, AJ420240, GQ161944	HY Zou^b^
B*52:01:02	B*520102	B52(5)	—	AUCA#2, TOB-137, BA8, 1510200304, SZ-86	L20090, L76091, L47984, L77205, GQ161945	HY Zou^b^
B*52:01:03	B*520103	B52(5)	B*52011V	GN00339, JMDP01K022	AF226838, AF226839, AB435517	K Tadokoro^b^
B*52:01:04	B*520104	B52(5)	—	0416-2859-5	AJ507651, AJ507652	
B*52:01:05	B*520105	B52(5)	—	BY00488	GQ410107	CK Hurley
B*52:02	B*5202	—	B*52012V	GN00314	AF181844, AF181845	
B*52:03	B*5203	—	B*52012V	GN00365	AF281152, AF281153	
B*52:04	B*5204	B52(5)	—	MS23477	AJ316288, AJ426470, AJ426467, AJ417684, AJ417673	
B*52:05	B*5205	—	B*5201V2	TBC-B31953	AB087517, AB087518	
B*52:06:01	B*520601	—	—	18230	AJ749610	
B*52:06:02	B*520602	B52(5)	—	TBC64726, scu01715, HN-01589-2, HN-69037-2	AB327275, EU080976, FJ594570, FJ594585	M Satake, FdP Sanchez Gordo^b^, Histogenetics^b^
B*52:07	B*5207	B52(5)	—	TBC53336	AB211961	M Satake
B*52:08	B*5208	B52(5)	—	NS5957	AB212969, AB212970	T Kinoshita
B*52:09	B*5209	—	—	160039	AM181591	K Witter
B*52:10	B*5210	—	—	601407	DQ537945	C Moehlenkamp
B*52:11	B*5211	—	—	33207, JMDP36K012	EF207232, AB435166	J Li, K Tadokoro^b^
B*52:12	B*5212	B52(5)	—	EPM-17177, LINAPau, JMDP01K020	EU517716, AM950324, AB435516	G Rampin, A Dormoy^b^, K Tadokoro^b^
B*52:13	B*5213	—	—	BY00312	EU643604	(36)
B*52:14	B*5214	—	—	306458	EU672816	(245)
B*52:15	B*5215	—	—	HN-45860-4	FJ235005	Histogenetics
B*52:16	B*5216	—	—	BY00523	GQ867217	CK Hurley
B*52:17	B*5217	—	—	HN-0387022	FJ765823	Histogenetics
B*52:18	B*5218	—	—	HN-3280919	FJ875668	Histogenetics
B*52:19	B*5219	—	—	HN-8583366	FJ765767	Histogenetics
B*53:01:01	B*530101	B53	—	AMAI, AM, 046, 1510200305	M58636, U90566, AJ311599	
B*53:01:02	B*530102	B53	—	CBRL 9-36-374	AY598426, AY598427	
B*53:01:03	B*530103	B53	—	AK42	AY705914	M Lin
B*53:01:04	B*530104	B53	—	NMDP-0414-1934-2	DQ865480	D Crowe
B*53:01:05	B*530105	B53	—	HN-0641111, HN-2565401	FJ866143, FJ765567	Histogenetics
B*53:02	B*5302	—	—	S15(28)	U63561, U63562	
B*53:03	B*5303	—	—	GN00231	AF071769, AF071770	
B*53:04	B*5304	—	B*CD	CD-ARCBS	AF117772, AF117773	
B*53:05	B*5305	—	B*5301V	GN00325, 24961vtis, JMDP01K023	AF198652, AF198653, AF304002, AF304003, AB435518	K Tadokoro^b^
B*53:06	B*5306	—	B*51/53New	SIA	AJ276996	
B*53:07	B*5307	B53	B*53/37	49716	AJ293856, AJ293857	
B*53:08:01	B*530801	—	—	2000-077-189	AY034802, AY034803	
B*53:08:02	B*530802	—	—	HN-83406-0	FJ765858	Histogenetics
B*53:09	B*5309	—	—	BY0023, BY00346	AY050191, AY050192, EU924802	CK Hurley^b^
B*53:10	B*5310	—	—	UCLA-DNA#191	AB196343	M Satake
B*53:11	B*5311	B53	—	CTM7097881	DQ188812	JL Vicario
B*53:12	B*5312	—	—	HSR117324	AM292927	(246)
B*53:13	B*5313	—	—	NT00734	EF563146	(85)
B*53:14	B*5314	—	—	D28023	AM911066	O Avinens
B*53:15	B*5315	—	—	NT00770, BY00311	EU330470, EU643606	CK Hurley
B*53:16	B*5316	—	—	BY00300	EU555318	(36)
B*53:17	B*5317	B53	—	CHE-B53	FM955319	(176)
B*53:18	B*5318	—	—	BY00394, HN-2408596, HN-2686538	FJ619500, FJ765575, FJ765795	CK Hurley, Histogenetics^b^
B*53:19	B*5319	—	—	HN-53187-1	FJ346257	Histogenetics
B*53:20	B*5320	—	—	HN-24211-5	FJ875673	Histogenetics
B*53:21	B*5321	—	—	BY00536	GU066748	CK Hurley
B*54:01	B*5401	B54(22)	—	LKT-3, TTL, SZ-78	M77774, GQ161937	(247)^b^
B*54:02	B*5402	B54(22)	B5401V1	JCBB18561	AB032095	
B*54:03	B*5403	—	—	21010305153947	AJ853298	
B*54:04	B*5404	—	—	TBC52156	AB197050	M Satake
B*54:05N	B*5405N	Null	—	TBC49946	AB197051	M Satake
B*54:06	B*5406	—	—	TBC50621	AB196428	M Satake
B*54:07	B*5407	—	—	TBC53384	AB211245	M Satake
B*54:08N	B*5408N	Null	—	HZB8296	DQ295998, DQ295999, DQ29600	(248)
B*54:09	B*5409	—	—	BY00134	DQ984200	(206)
B*54:10	B*5410	—	—	K34690, CTJ-11362	EF081456, EF136580	(249), L Yan
B*54:11	B*5411	B54(22)	—	CTJ-10063	EF136579	(250)
B*54:12	B*5412	—	—	Shanxi001	EF471993	D Zhang
B*54:13	B*5413	—	—	20051539M, 20051539seq	AB301477	H Inoko
B*54:14	B*5414	—	—	JMDP36K013, K19992	AB435167, AB368299	K Tadokoro, E Maruya^b^
B*54:15	B*5415	—	—	JMDP01K024	AB435519	K Tadokoro
B*54:16	B*5416	—	—	BJ074508	EU939322	X Shan
B*54:17	B*5417	—	—	SZ-16	FJ169946	(247)
B*54:18	B*5418	—	—	CG077022121979	FJ648689	H Hogan
B*54:19	B*5419	—	—	UD5749	GQ2000019	(251)
B*55:01:01	B*550101	B55(22)	—	VEN	M77778, AJ310509	
B*55:01:02	B*550102	B55(22)	—	VTIS120923	AY826760, AY826761	BD Tait
B*55:01:03	B*550103	B55(22)	—	TUWI3303661AN, MSA1, MSB1, MSC1	AM055594, AM412645	AM Little, M Schroeder^b^
B*55:01:04	B*550104	B55(22)	—	33137, BY00145	AM075814, EF370119	(252), CK Hurley^b^
B*55:01:05	B*550105	B55(22)	—	HN-35215-5, HN-58783-8, HN-51942-7, HN-52552-3, HN-48040-6, HN-53197-2	FJ346330, FJ858893, FJ858894, FJ858896, FJ875567, GQ914792	Histogenetics
B*55:01:06	B*550106	B55(22)	—	HN-25089-4, HN-5851238, HN-5775512, HN-22333-3, HN-2102, HN-42409-1	FJ392185, FJ560456, FJ969943, FJ976767, FJ976782, GQ491084	Histogenetics
B*55:02:01	B*550201	B55(22)	—	APA, BY00458, SZ-80	M77777, FJ842972, GQ161946	CK Hurley^b^, HY Zou^b^
B*55:02:02	B*550202	B55(22)	—	N2915	DQ320646, DQ320647	LA Baxter-Lowe
B*55:02:03	B*550203	B55(22)	—	B27257, HN-17598-4, HN-7398230, HN-0381249	FJ222392, FJ235039, FJ235047, FJ765802	(253), Histogenetics^b^
B*55:02:04	B*550204	B55(22)	—	JMDP36K037	AB512684	K Tadokoro
B*55:02:05	B*550205	B55(22)	—	P4176	GQ999613	L Yan
B*55:03	B*5503	B55(22)^c^	B5501v	RCE70	X94482	
B*55:04	B*5504	B55(22)	B-4201v, B55.2	TAGO, 11840Kane, KIW, JMDP01K039	L76225, D85761, D89333, D89334, AB436532	K Tadokoro^b^
B*55:05	B*5505	B22	B5501 W669R	B55W669R	U63653	
B*55:07	B*5507	B54(22)	—	8138, 9070	AF042289, AF042290	
B*55:08	B*5508	B56(22)	B*ER	DIA2 98629, VTIS31300	AF091343, AF091344, AF304004, AF304005	
B*55:09	B*5509	B22	S-PB55	13215	AJ250628, AJ250629	
B*55:10	B*5510	B55(22)	B5502V1, B55v	JCBB1366, BY0028, JMDP36K026	AB032094, AF408166, AF408167, AB436624	K Tadokoro^b^
B*55:11	B*5511	—	—	2000-259-501	AY042674, AY042675	
B*55:12	B*5512	B22	—	10002057	AJ420106	
B*55:13	B*5513	—	—	KSY-AJ23	AY258135, AY258136	
B*55:14	B*5514	—	—	117562	AJ556167	
B*55:15	B*5515	B55(22)	B*55AHU	B55AHU	AJ557018, AJ557019	
B*55:16	B*5516	B22	—	CHD635, MHH0302483	AY339847, AJ966345	P Horn^b^
B*55:17	B*5517	—	—	150113, NT00530	AJ866776, AY903435, AY903436	(254), CK Hurley^b^
B*55:18	B*5518	—	—	50461458, 5045856	AJ871373	(255)
B*55:19	B*5519	—	—	2005010716	AY887663, AY887664	(256)
B*55:20	B*5520	—	—	56352, NT00572	AY504809, AY504810, DQ120786, DQ120787	(147)
B*55:21	B*5521	—	—	BY00088	DQ455012	(57)
B*55:22	B*5522	B55(22)	—	HZB9385	DQ458812, DQ458813, DQ458814	(257)
B*55:23	B*5523	—	—	BY00129, NT00769	DQ924383, EU330469	(149), CK Hurley^b^
B*55:24	B*5524	—	—	K102665	AM396518	C Dunne
B*55:25	B*5525	—	B*55MVE0906	MHHN-189448, HN-19679-3, HN-453055, HN-55322-0	AM407883, FJ-594583, FJ853765, FJ765830	R Blasczyk, Histogenetics^b^
B*55:26	B*5526	—	—	NT00719	EF422080	(85)
B*55:27	B*5527	—	—	Shanxi003	EF542832	D Zhang
B*55:28	B*5528	—	—	105488	AM779472	J Enczmann
B*55:29	B*5529	—	—	2007-4621, BY00507	AM922105, GQ410092	(258), CK Hurley^b^
B*55:30	B*5530	—	—	HZZJCB5723	EU422994	(259)
B*55:31	B*5531	—	—	BY00317, HN-0036645	EU643599, FJ235048	(36), Histogenetics^b^
B*55:32	B*5532	—	—	JMDP36K014	AB435168	K Tadokoro
B*55:33	B*5533	—	—	7218217, HN-01099-0, HN-62070-0	EU445575, FJ346275, FJ969945	A Vigh, Histogenetics^b^
B*55:34	B*5534	—	—	BJ51, BY00443	EU325940, FJ688149	Z Zhang, CK Hurley^b^
B*55:35	B*5535	—	—	63203676	FJ898284	(260)
B*55:36	B*5536	—	—	HN-92198-5, HN-76461-7, HN-74546-7, HN-92199-3	FJ235061, FJ235062, FJ976778, FJ976779	Histogenetics
B*55:37	B*5537	—	—	Xian55	GQ231483	S Ye
B*55:38	B*5538	—	—	HN-86943-7	FJ549411	Histogenetics
B*55:39	B*5539	—	—	HN-1576783	FJ765822	Histogenetics
B*56:01:01	B*560101	B56(22)	—	VOO, NT00688, CU45C, SZ-71	M77776, EF156368, EF203080, GQ161938	CK Hurley^b^, (160)^b^, HN Zou^b^
B*56:01:02	B*560102	B56(22)	—	HN-145136, HN-96819-8, HN-43069-5, HN-87472-3	FJ502334, FJ976770, FJ976772, GQ994062	Histogenetics
B*56:01:03	B*560103	B56(22)	—	HN-699D	FJ796991	Histogenetics
B*56:02	B*5602	B56(22)	—	ENA, BY00553	M77775, GU138073	CK Hurley^b^
B*56:03	B*5603	B22	B22N, B56/46	15630Naka, 01300, 01094, NPC-4, SZ-99	D85762, U67746, U67747, U67749, U73113, GQ161939	HY Zou^b^
B*56:04	B*5604	B56(22)	B*5602Var	5227, 5274, SZ-98	U93911, U93912, U93913, U93914, GQ161940	HY Zou^b^
B*56:05:01	B*560501	B56(22)	B56v	234-1047, CBC11028, TBC63580	AF072767, AF072768, AB030574, AB292219	M Satake^b^
B*56:05:02	B*560502	B56(22)	—	CMC3	AF538968, AF538969	
B*56:06	B*5606	B78	B*7801New	20598, AFM	Y18542, Y18543, AJ276993	
B*56:07	B*5607	B56(22)	B*New B56-Bw4	20193, VTIS45561	Y18544, Y18545, AF387903, AF387904	
B*56:08	B*5608	—	—	1PF6	AY045733, AY045734	
B*56:09	B*5609	—	B*55V2	TBC-T4899	AB087513, AB087514	
B*56:10	B*5610	B55(22)	—	SZ-3, TBC26606	AY134744, AB254370	M Satake^b^
B*56:11	B*5611	B56(22)	—	R24503	AJ514938, AJ514939, AJ514940	
B*56:12	B*5612	B55(22)	—	BMT46M	AY297540	
B*56:13	B*5613	B56(22)	—	WEA	AJ632195, AJ632196	
B*56:14	B*5614	—	—	HZCB1649, BY00125	AY601726, AY601727, AY601728, DQ832583	CK Hurley^b^
B*56:15	B*5615	B56(22)	—	100304, NT00648	AJ864515, AJ864516, AJ864517, DQ401176, DQ401177	CK Hurley^b^
B*56:16	B*5616	—	—	NT00595, NT01075	DQ096573, DQ096574, GQ251368	(147), CK Hurley^b^
B*56:17	B*5617	—	—	BY00105	DQ514600	(149)
B*56:18	B*5618	—	—	SZ-5	EF016753	(183)
B*56:19N	B*5619N	Null	—	TBC62727	AB282749	M Satake
B*56:20	B*5620	B56(22)	—	R06-978	AM422124	(261)
B*56:21	B*5621	B56(22)	—	45112351	EU079373	C Zhang
B*56:22	B*5622	—	—	BY00284	EU522466	(36)
B*56:23	B*5623	—	—	BY00272	EU522478	(36)
B*56:24	B*5624	—	—	08d02619	FM211273	JDH Anholts
B*56:25	B*5625	—	—	HN-56075-6	FJ765856	Histogenetics
B*56:26	B*5626	—	—	BY00479, HN-0340062	FJ765799	CK Hurley, Histogenetics
B*56:27	B*5627	—	—	HN-51990-6	FJ858895	Histogenetics
B*57:01:01	B*570101	B57(17)	—	WIN, MOC, MOLT4, 1510200311, PAT495	X55711, M32318, AJ458991, AF196183, DQ249180	(141)^b^
B*57:01:02	B*570102	B57(17)	—	GN00398	AY050203, AY050204	
B*57:01:03	B*570103	B57(17)	—	NT00590	DQ096571, DQ096572	(149)
B*57:01:04	B*570104	B57(17)	—	HN-95376-9	FJ235001	Histogenetics
B*57:01:05	B*570105	B57(17)	—	HN-25308-1, HN-59275-0	FJ600628, GQ859549	Histogenetics
B*57:01:06	B*570106	B57(17)	—	HN-93081-6	FJ866182	Histogenetics
B*57:02:01	B*570201	B57(17)	Bw57.2	32/32, NT00691	X61707, EF156372	CK Hurley^b^
B*57:02:02	B*570202	B57(17)	—	HN-6130964, HN-4094699, HN-711438	FJ502326, FJ765835, FJ468327	Histogenetics
B*57:03:01	B*570301	B57(17)	B*57SAU	SAU, MAME, GB32	U18790, U39088, Y09157	
B*57:03:02	B*570302	B57(17)	B*57New	E187	AF279663	
B*57:04	B*5704	B57(17)	B-5702v	OPOU, NT00689	L76096, EF156369	CK Hurley^b^
B*57:05	B*5705	—	—	GN00213	AF061859, AF061860	
B*57:06	B*5706	—	B*57New	CTM2988653	AF130734, DQ191316	JL Vicario^b^
B*57:07	B*5707	—	—	GN00327	AF202449, AF202450	
B*57:08	B*5708	B57(17)	—	35980	AJ409214	
B*57:09	B*5709	—	—	2000-245-285	AY034804, AY034805	
B*57:10	B*5710	—	—	NT00604	DQ120782, DQ121783	(149)
B*57:11	B*5711	—	—	K104827, NT00721	AM295982, EF422077	C Dunne, CK Hurley^b^
B*57:12	B*5712	—	—	BY00149	EF484938	(85)
B*57:13	B*5713	—	—	MHHN-414997, MHHN-593828, HN-452007	AM712179, AM778678, FJ594566	R Blasczyk, P Horn^b^, Histogenetics^b^
B*57:14	B*5714	—	—	FD, BY00275, HN-22060-9, HN-22061-7	AM850138, EU522475, FJ594572, FJ594573	(262), CK Hurley^b^, Histogenetics^b^
B*57:15	B*5715	—	—	NT00744, HN-83399-8	EU185513, FJ853769	(85), Histogenetics^b^
B*57:16	B*5716	B57(17)	—	BS723143	AM889027	(261)
B*57:17	B*5717	—	—	BY00315, HN-6965989	EU643601, FJ502333	(36), Histogenetics^b^
B*57:18	B*5718	—	—	0712ID10	FJ174674	WH Chen
B*57:19	B*5719	—	57KEM0808	MHHI-630836	FM207448	(263)
B*57:20	B*5720	—	—	HN-50663-5, HN-42022-5	FJ346299, FJ866155	Histogenetics
B*57:21	B*5721	—	—	HN-42215-6	FJ392176	Histogenetics
B*57:22	B*5722	—	—	HN-52968-5, HN-4824705	FJ234991, FJ765866	Histogenetics
B*57:23	B*5723	—	—	HN-84512-5, HN-6905589	FJ235060, FJ494822	Histogenetics
B*57:24	B*5724	—	—	NT01068	GQ251346	CK Hurley
B*57:25	B*5725	—	—	NT01066	GQ251344	CK Hurley
B*57:26	B*5726	—	—	HN-44152-7, HN-94214-4	FJ494819, GQ859540	Histogenetics
B*57:27	B*5727	—	—	HN-2155559	FJ765827	Histogenetics
B*57:28N	B*5728N	Null	—	An188086	HN555101	F Poli
B*57:29	B*5729	—	—	SZGSQ-4	GU166293	SQ Gao
B*58:01:01	B*580101	B58(17)	—	WT49, DAUDI, GN00107, 1075011, HGN, KBM, 1510200312	M11799, U52813, U52814, U65395, U65396, AB008102, AJ420241, AF196184	
B*58:01:02	B*580102	B58(17)	—	B28287	EU499350	(264)
B*58:01:03	B*580103	B58(17)	—	HN-3412164	FJ792531	Histogenetics
B*58:01:04	B*580104	B58(17)	—	B10020	GQ452848	K Du
B*58:02	B*5802	—	B58v	DAUDI, RCE56, CR-30609	L33923, X86703, AJ133780, AJ133781	
B*58:04	B*5804	—	—	99-2199	AF189245, AF189246, AF189247	
B*58:05	B*5805	—	B*5801V	GN00322	AF201474, AF201475	
B*58:06	B*5806	—	B*5802V	GN003714, BY00407	AF288046, FJ619489	CK Hurley^b^
B*58:07	B*5807	—	—	0461-7571-7	AJ507657, AJ507658	
B*58:08	B*5808	B17	—	SO01-516	AJ548504, AJ548505, Aj548506	
B*58:09	B*5809	—	—	78802	AJ555247, AJ555248, AJ555249	
B*58:10N	B*5810N	Null	—	50-84786	AB176923	
B*58:11	B*5811	—	—	40816S, 40540S, 87861	AY918169, DQ140400	S Adams, MS Leffell^b^
B*58:12	B*5812	—	—	BY00092, BY00086	DQ455013, DQ455020	(57)
B*58:13	B*5813	—	—	2006042530	DQ648737	(265)
B*58:14	B*5814	—	—	VTIS143978	EF088201	BD Tait
B*58:15	B*5815	—	—	93996	EF471361	MS Leffell
B*58:16	B*5816	—	—	BY00260	EU185518	(36)
B*58:17N	B*5817N	Null	—	BY00303	EU564105	(36)
B*58:18	B*5818	—	—	BY00310, HN-6543265, BY00528	EU643605, FJ765806, GU066740	(36), Histogenetics^b^, CK Hurley^b^
B*58:19	B*5819	—	—	JMDP36K016	AB435237	K Tadokoro
B*58:20	B*5820	B58(17)	—	CHET	AM497780	V Dubois
B*58:21	B*5821	—	—	AKB-206504, HN-B-173923	FM992853, FJ235054	R Blasczyk, Histogenetics^b^
B*58:22	B*5822	—	—	HN-6298546	FJ235000	Histogenetics
B*58:23	B*5823	—	—	HN-40574-5, HN-45784-7	FJ346244, FJ346301	Histogenetics
B*58:24	B*5824	—	—	BY00482, HN-0954615	GQ410101, FJ765824	CK Hurley, Histogenetics^b^
B*58:25	B*5825	—	—	HN-0516510	FJ765786	Histogenetics
B*58:26	B*5826	—	—	LAAL686132AN	FN555526	SGE Marsh
B*58:27	B*5827	—	—	DL-BK	GU071234	BS Ke
B*58:28	B*5828	—	—	BY00573	GU256006	CK Hurley
B*59:01	B*5901	B59	—	AT, KY, MAS, SZ-90	L07743, D50300, GQ161941	HY Zou^b^
B*59:02	B*5902	B59	—	TBC60261	AB257504	M Satake
B*59:03	B*5903	—	—	JMDP01K025	AB435520	K Tadokoro
B*59:04	B*5904	—	B*5901V	KPUM88, JMDP01K054	AB467317, AB537166	(266), K Tadokoro^b^
B*59:05	B*5905	—	—	JMDP36K034	AB477103	K Tadokoro
B*67:01:01	B*670101	B67	—	HS67, #591, #W7079, PVR, SZ-70	L17005, L76252, GQ161947	HY Zou^b^
B*67:01:02	B*670102	B67	B*67LAV	LAV, TBC63059, SZ-90	U18789, AB292220, GQ161948	M Satake^b^, HY Zou^b^
B*67:02	B*6702	—	—	BY00014, BY0026, JH66203	AF321834, AF321835, AY050195, AY050196, AF487379	
B*73:01	B*7301	B73	—	LK707, LE023, HL	U04787, X77658, L24373, AJ311601	
B*78:01	B*7801	B78	—	B’SNA’,Bx1, SNA, 32/32, Terasaki Ext#69	X61708, M33573, AJ309192	
B*78:02:01	B*780201	B78	—	RC654	L41214	
B*78:02:02	B*780202	B78	B78Hen	Hen	X96534, X96533	
B*78:03	B*7803	—	—	GN00209	AF061855, AF061856	
B*78:04	B*7804	—	B*78New	COH#1058, NT01094	AJ012471, AJ132713, AJ132714, GQ251356	CK Hurley^b^
B*78:05	B*7805	—	B52 variant	B5859, TBC24079	AB051357, AB254369	M Satake^b^
B*78:06	B*7806	—	—	JMDP01K026	AB435521	K Tadokoro
B*81:01	B*8101	B81	B’DT', B*7x48GB, B56b	AP630, GB92, 56B	L37880, X90390, U34810	
B*81:02	B*8102	B81	—	TER-1157, 82488	AJ580912, AJ580913, AJ580914, AY769916	
B*81:03	B*8103	—	—	ShanXi005	EU048339	D Zhang
B*81:04N	B*8104N	Null	—	BY00279	EU522471	(36)
B*82:01	B*8201	B82	B22x45, B45v, B*82new-64B	MAME, MAMA, MAPA, RB22, VWAR, 64B	U29241, U38800, U36492, U43337, AF017321	
B*82:02	B*8202	—	B*8201New	CEK008AN, VTIS68967	AJ251755, AF525409, AF525410	
B*82:03	B*8203	—	—	HN-7535450	FJ765800	Histogenetics
B*83:01	B*8301	—	B*5603V	GN00298, GN00298, CUI-36	AF176083, AF176084, AF275748, AF275749, FJ175384	J Martinez-Laso^b^

a Allele names given in bold type have been assigned since the 2004 Nomenclature report.

b This reference is to a confirmatory sequence.

c HLA specificity provided from the HLA dictionary (22–26).

**Table 4:** Designations of HLA-C, -E, -F and -G alleles

HLA allele^a^	Pre 2010 designation	HLA specificity	Previous equivalents	Individual or cell line from which the sequence was derived	Accession number	References or submitting author(s)
C*01:02:01	Cw*010201	Cw1	Cw1.2, C1J1	T7527, AP, LCL721, KRC005, TTY, BRUG, LCL721, 08009391, 009386	M84171, Z46809, D50852, M16272, AJ420242, FJ515900, FJ827032	(267), Y Xu^b^
C*01:02:02	Cw*010202	Cw1	Cw*01LET	LET	AJ579644, AJ579645, AJ579646	
C*01:02:03	Cw*010203	Cw1	—	2004102038	AY789181, AY789182	
C*01:02:04	Cw*010204	Cw1	—	37109	AM397243	T Gervais
C*01:02:05	Cw*010205	Cw1	—	277745	AM422978	(268)
C*01:02:06	Cw*010206	Cw1	—	80549	FM179946	M Danzer
C*01:02:07	Cw*010207	Cw1	—	HN-76798-8, HN-89138-1, HN-69617-8, HN-74501-7, HN-85504-9, HN-62015-7, HN-27629-4, HN-61305-8, HN-34543-8, HN-83954-8, HN-81703-5, HN-27905-9, HN-28170-6	FJ594542, FJ614589, FJ614594, FJ614595, FJ765748, FJ765761, FJ976825, FJ976828, FJ976835, FJ976849, FJ976879, GQ180267, GQ180445, GQ254397	Histogenetics
C*01:02:08	Cw*010208	Cw1	—	HN-11284-8, HN-06147-1, HN-14364-7, HN-72579-7, HN-91940-5, HN-05038-3, HN-12199-9, HN-86405-6, HN-11354-9, HN-77895-3, HN-20485-5, HN-02906-7, HN-55932-2, HN-53240-0, HN-35686-3, HN-57785-8, HN-99074-6, HN-87701-9	FJ618921, FJ614588, FJ614613, FJ614614, FJ765745, FJ765747, FJ765754, FJ765766, FJ976805, FJ976809, GQ491100, GU128019, GQ180241, GQ180248, GQ180389, GQ180411, GQ2404887, GQ254390	Histogenetics
C*01:02:09	Cw*010209	Cw1	—	HN-67204-1	FJ875627	Histogenetics
C*01:03	Cw*0103	Cw1	C1J2	ITOU, 41761, 7550800434	D64145, FM999987, GQ472834	T Gervais^b^, (269)
C*01:04	Cw*0104	—	Cw*01/12	J.V	AJ133100	
C*01:05	Cw*0105	—	Cw*01variant	607990	AJ300765, AJ300766	
C*01:06	Cw*0106	—	—	SWC231, BY00451, SZ-28	AJ418708, AJ418709, FJ842962, GQ304758	CK Hurley^b^, HY Zou^b^
C*01:07	Cw*0107	—	—	VTIS67160	AF525405, AF525406	
C*01:08	Cw*0108	—	—	Woo*2, BY00072, 7550800556	AF535211, AF535212, DQ244131, DQ244132, GQ472835	CK Hurley^b^, (269)^b^
C*01:09	Cw*0109	Cw1	—	VTIS78432	AF539618, AF539619	
C*01:10	Cw*0110	—	—	231396, BY00574	AJ621024, AJ621025, GU256007	CK Hurley^b^
C*01:11	Cw*0111	—	—	200412012	AY887665, AY887666, AY887667	(270)
C*01:12	Cw*0112	—	—	VTIS121253	DQ400524, DQ400525	BD Tait
C*01:13	Cw*0113	—	—	NT00651, NT01116	DQ401182, DQ401183, GQ867210	(271), CK Hurley^b^
C*01:14	Cw*0114	—	—	200tp2343	AM418559	M Bengtsson
C*01:15	Cw*0115	—	—	241551	AM422968	T Lebedeva
C*01:16	Cw*0116	—	—	250226	AM422972	(268)
C*01:17	Cw*0117	—	—	255993, HN-82549-9	AM489405, FJ792517	(268), Histogenetics^b^
C*01:18	Cw*0118	—	—	277100	AM422975	(268)
C*01:19	Cw*0119	—	—	HanChineseA78	EF189140	(272)
C*01:20	Cw*0120	—	—	7213718	EU428006	J Mytilineos
C*01:21	Cw*0121	—	—	SZ-12	EU617015	(273)
C*01:22	Cw*0122	—	—	37-12333	AB378484	N Araki
C*01:23	Cw*0123	—	—	015298X	FJ594417	Z Goburdhun
C*01:24	Cw*0124	—	—	SZ-19	FJ644940	(274)
C*01:25	Cw*0125	—	—	JOSDan	FM998813	A Dormoy
C*01:26	Cw*0126	—	—	NT01038, HN-726956	FJ797364, FJ792496	CK Hurley, Histogenetics^b^
C*01:27	Cw*0127	—	—	NT01032, HN-683991, HN-2854299, CMHD296.4, HN-35341-6	FJ797370, FJ792507, FJ765875, GQ402147, GU128046	CK Hurley, Histogenetics^b^, M Yu^b^
C*01:28	Cw*0128	—	—	Nt01059	Fj976703	CK Hurley
C*01:29	Cw*0129	—	—	BJYGX	GQ376196	Z Zhang
C*01:30	Cw*0130	—	—	SZBM170	GQ373181	(275)
C*01:31	Cw*0131	—	—	HN-06842-8	FJ392205	Histogenetics
C*01:32	Cw*0132	—	—	HN-98676-0, HN-81926-9, HN-59114-5, HN-10461-1, HN-58910-7, HN-93291-2, HN-65647-0, HN-01902-2, HN-29077-1, HN-45369-1, HN-12267-3, HN-14507-0, HN-81436-2, HN-39457-9, HN-05381-9, HN-89122-6, HN-01820-8	FJ614610, FJ614618, FJ875585, FJ976798, FJ976875, FJ976876, FJ976882, GQ149303, GQ345071, GQ401221, GQ859570, GU017959, GQ161036, GQ180254, GQ180418, GQ180420, GQ240482	Histogenetics
C*01:33	Cw*0133	—	—	HN-30085-6	FJ614617	Histogenetics
C*01:34	Cw*0134	—	—	PBMC-JQZ	GQ365731	JQ Zhang
C*02:02:01	Cw*020201	Cw2	Cw2.2	MVL	M24030	
C*02:02:02	Cw*020202	Cw2	Cw2.2	SWEIG, BDG, BRUG, SWEIG007, 7550800714	M26712, D83029, M16273, AJ420243, GQ472836	Y Xu^b^
C*02:02:03	Cw*020203	Cw2	—	KACD	Z72007	
C*02:02:05	Cw*020205	Cw2	—	1177	AY028705, AY028706	
C*02:02:06	Cw*020206	Cw2	—	MHHUDS-587930	AM778451	R Blasczyk
C*02:02:07	Cw*020207	Cw2	—	HN-85664-1, HN-60463-7, HN-709325, HN-55574-4, HN-46896-4, HN-41928-9, HN-34692-0, HN-99447-2, HN-13976-5, HN-58481-2, 09215494, HN-06181-7, HN-42878-4, HN-15322-6, HN-55708-8, HN-16328-8, HN-11201-0, HN-83016-5, HN-56299-2, HN-75876-6	FJ594543, FJ594540, FJ792502, FJ858900, FJ858905, FJ875586, FJ875618, FJ917725, FJ976785, FJ976827, GQ850384, GQ245365, GQ401220, GQ859557, GQ859571, GU128039, GQ161064, GQ161067, GQ161069, GQ180442	Histogenetics, D Fuerst^b^
C*02:02:08	Cw*020208	Cw2	—	BY00460, HN-01975-6, HN-83001-7, HN-01916-5, HN-05717-7	FJ976696, FJ392216, FJ875624, FJ976884, GQ180244	CK Hurley, Histogenetics^b^
C*02:02:09	Cw*020209	Cw2	—	HN-70709-9, HN-28218-5, HN-97117-5	FJ640584, FJ976830, FJ976839	Histogenetics
C*02:02:10	Cw*020210	Cw2	—	HN-25390-3	FJ594638	Histogenetics
C*02:02:11	Cw*020211	Cw2	—	HN-66330-9	FJ765993	Histogenetics
C*02:03	Cw*0203	—	—	NM3340	AF037449, AF037450	
C*02:04	Cw*0204	—	—	PRC32	AF281055, AF281056	
C*02:05	Cw*0205	—	—	1206	AY028707, AY028708	
C*02:06	Cw*0206	—	—	LB65799, LB65894, LB65920	AJ510090, AJ510091	
C*02:07	Cw*0207	—	—	GN00428	AY229981, AY229982	
C*02:08	Cw*0208	Cw2	—	E805	AY2308567, AY230857	
C*02:09	Cw*0209	—	—	218979	AJ616298, AJ616299	
C*02:10	Cw*0210	Cw2	Cw*020204	HEL299, NM155, NM233, NM239, NM303, NM366, NM72, 19215, MAN527	U88838, U88839, U97346, U97347, Z96924, AJ011881, Y18660, Y18661, Y18144, Y18145, Z96924	EM vd Berg Loonen, SGE Marsh^b^
C*02:11	Cw*0211	—	—	CTM4900800	AY973961	JL Vicario
C*02:12	Cw*0212	—	—	BY00066	DQ086792, DQ086793	(271)
C*02:13	Cw*0213	—	—	JUNYa	AM180649	A Dormoy
C*02:14	Cw*0214	—	02NEE1205	MHHNEE144, NT00658	AM180651, DQ465614	(276), CK Hurley^b^
C*02:15	Cw*0215	—	—	US-1-0446-2875-8	AM260635	A Wölpl
C*02:16:01	Cw*021601	—	—	NT00671	DQ648007	(271)
C*02:16:02	Cw*021602	—	—	PARAC, I-643356, HN-79217-5, HN-96848-6, HN-58658-8	FJ542024, FM991870, FJ976790, FJ976838, GU017962	(277), R Blasczyk^b^, Histogenetics^b^
C*02:17	Cw*0217	—	—	C-HLA-370, C-HLA-493	AM419012	(278)
C*02:18	Cw*0218	—	—	250052	AM422969	(268)
C*02:19	Cw*0219	—	—	DN1277.4	EU003584	M Yu
C*02:20	Cw*0220	Cw2	—	CTM-45697391	EU080975	(279)
C*02:21	Cw*0221	—	—	Scu00977	EU499385	(119)
C*02:22	Cw*0222	Cw2	—	41873	FM164942	(280)
C*02:23	Cw*0223	—	Cw*02new	CTM-2001095	FJ415311	(46)
C*02:24	Cw*0224	—	—	HN-73058-3, HN-52158-1	FJ594525, FJ640583	Histogenetics
C*02:25Q	Cw*0225Q	—	—	HN-24736-9, HN-24492-7	FJ594546, GQ449662	Histogenetics
C*02:26	Cw*0226	—	—	NT001041, HN-749974	FJ797361, FJ792494	CK Hurley, Histogenetics^b^
C*02:27	Cw*0227	—	—	NT01036, HN-699260, HN-41133-6, HN-46734-8	FJ797366, FJ792501, FJ875588, GQ900570	CK Hurley, Histogenetics^b^
C*02:28	Cw*0228	—	—	HN-4042631	EU887013	Histogenetics
C*02:29	Cw*0229	—	—	DEWS108	FN430834	V Dubois
C*02:30	Cw*0230	—	—	HN-29634-4, HN-19588-2, HN-09354-8	FJ392212, GQ161065, GQ994080	Histogenetics
C*02:31	Cw*0231	—	—	HN-58665-1, HN-42278-2	FJ594510, GQ180394	Histogenetics
C*03:02:01	Cw*030201	Cw10(w3)	—	AP, JG, SZ-104	M84172, AJ011884, FJ973626	HY Zou^b^
C*03:02:02	Cw*030202	Cw10(w3)	—	DAUDI, 08009411	AJ318865, FJ827033	(281)^b^
C*03:02:03	Cw*030203	Cw10(w3)	—	CTM-SCU-423	EF370045	(46)
C*03:02:04	Cw*030204	Cw10(w3)	—	HN-11752-7	FJ594514	Histogenetics
C*03:02:05	Cw*030205	Cw10(w3)	—	HN-46531-4	FJ594517	Histogenetics
C*03:02:06	Cw*030206	Cw10(w3)	—	HN-759098	FJ792493	Histogenetics
C*03:03:01	Cw*030301	Cw9(w3)	C3J1	GRC150, SJK, 08009372	M99390, D50853, FJ515902	(282)
C*03:03:02	Cw*030302	Cw9(w3)	—	NM2688, NM3499	AF036554, AF036555	
C*03:03:03	Cw*030303	Cw9(w3)	—	TER#1054	AJ298837	
C*03:03:04	Cw*030304	Cw9(w3)	—	149740, 226727, 220952	AJ616300, AJ616301, AJ616302, AJ616303, AJ620459, AJ620460	
C*03:03:05	Cw*030305	Cw9(w3)	—	BY00111	DQ514597	(271)
C*03:03:06	Cw*030306	Cw9(w3)	—	HN-12246-5	FJ594534	Histogenetics
C*03:03:07	Cw*030307	Cw9(w3)	—	HN-14862-8, HN-22864-4, HN-27728-6, HN-35608-0, HN-98335-0, HN-38356-3, HN-37529-6, HN-25247-5, HN-35739-3, HN-26451-7, HN-18083-7	FJ392222, FJ554597, FJ554598, FJ554599, FJ554600, FJ554601, FJ594533, FJ619435, FJ858906, GQ491098, GU128023	Histogenetics
C*03:03:08	Cw*030308	Cw9(w3)	—	HN-11662-3, HN16099-3, HN-53315-0	FJ554605, FJ554606, GQ994082	Histogenetics
C*03:03:09	Cw*030309	Cw9(w3)	—	HN-30371-8	FJ538259	Histogenetics
C*03:03:10	Cw*030310	Cw9(w3)	—	HN-11329-0, HN-16461-3	FJ594505, FJ765999	Histogenetics
C*03:03:11	Cw*030311	Cw9(w3)	—	HN-01895-0	FJ614587	Histogenetics
C*03:04:01:01	Cw*03040101	Cw10(w3)	C3J2	KRC110, JD, SKA, JG, PAT218, MCF	M99389, D64150, U44064, U31372, U31373, DQ249177, CR759828	(141)^b^, S Beck^b^
C*03:04:01:02	Cw*03040102	Cw10(w3)	—	08009378	FJ515903	Y Xu
C*03:04:02	Cw*030402	Cw10(w3)	—	NM233, NM303, NM366, ML1805, BG222, FH13	U97344, U97345, AJ133473, AJ133474, AY530953	
C*03:04:03	Cw*030403	Cw10(w3)	Cw*03TER1101	TER1101	AJ580000, AJ580001, AJ580002	
C*03:04:04	Cw*030404	Cw10(w3)	—	CM975, HN-4800309	DQ200948, DQ200949, FJ792513	(283), Histogenetics^b^
C*03:04:05	Cw*030405	Cw10(w3)	—	VTIS142034	DQ417102, DQ417103	BD Tait
C*03:04:06	Cw*030406	Cw10(w3)	—	275410	AM489409	(268)
C*03:04:07	Cw*030407	Cw10(w3)	—	277614	AM422977	(268)
C*03:04:08	Cw*030408	Cw10(w3)	—	HN-40440-2, HN-51167-7	FJ538266, FJ594528	Histogenetics
C*03:04:09	Cw*030409	Cw10(w3)	—	HN-06007-3	FJ554590	Histogenetics
C*03:04:10	Cw*030410	Cw10(w3)	—	HN-01439-0, HN-78828-1, HN-24757-6, HN-06759-3	FJ554596, FJ554603, FJ538260, FJ614592	Histogenetics
C*03:04:11	Cw*030411	Cw10(w3)	—	HN-34943-0, HN-21008-0, HN-87718-5, HN-53119-1, HN-98763-2	FJ554608, FJ538267, FJ594531, GQ240477, GQ345091	Histogenetics
C*03:04:12	Cw*030412	Cw10(w3)	—	HN-55005-2	FJ538262	Histogenetics
C*03:04:13	Cw*030413	Cw10(w3)	—	HN-87784-9	FJ392223	Histogenetics
C*03:04:14	Cw*030414	Cw10(w3)	—	1003824	FJ875295	E Palou
C*03:04:15	Cw*030415	Cw10(w3)	—	HN-20607-9	FJ619432	Histogenetics
C*03:04:16	Cw*030416	Cw10(w3)	—	HN-67433-1	FJ766000	Histogenetics
C*03:04:17	Cw*030417	Cw10(w3)	—	HN-60094-6, HN-56321-9, HN-51699-3, HN-51054-1, HN-50983-2	FJ765995, FJ875591, FJ875592, FJ875593, FJ875594	Histogenetics
C*03:04:18	Cw*030418	Cw10(w3)	—	HN-718581	FJ792503	Histogenetics
C*03:05	Cw*0305	—	MA083C, Cw*03MAC	MA083C, NM3214, NM3222, PAM	AF016303, AF009683, AJ005199	
C*03:06	Cw*0306	Cw10(w3)^c^	—	NM133, NM627, NM2203, NM2415, NM2616, QC-397-UCLA, CS00019	AF003283, AF003284, AY536515, GQ301204	K Cao^b^
C*03:07	Cw*0307	Cw3	—	CTM-7980718, NT01049	AF039198, FJ976705	CK Hurley^b^
C*03:08	Cw*0308	—	—	NM1931, TER0171	AF037074, AF037075, Y16411, Y16412, Y18656, Y16411, Y16412, Y18142, Y18143	
C*03:09	Cw*0309	Cw3^c^	—	NM4305	AF037076, AF037077	
C*03:10	Cw*0310	Cw3	Cw*03041New	NM4C187, DKM	AF138276, AF138277, AF147701, AF147702	
C*03:11:01	Cw*031101	—	Cw*03xx	NMDP0187-1868-4	AF145466, AF145467	
C*03:11:02	Cw*031102	Cw3	—	11845457, 14695022	AM087957	(284)
C*03:12	Cw*0312	—	—	UCLA022679917	AF172867, AF172868	
C*03:13	Cw*0313	—	Cw*03031var	10050195	AJ298116	
C*03:14	Cw*0314	—	Cw*KCULL	KCULL, LB58440, LB58441, LB58480	AF335314, AF335315, AJ506199, AJ506198	
C*03:15	Cw*0315	—	—	N322	AY078078, AY078079	
C*03:16	Cw*0316	—	—	MS14725	AJ504803, AJ504804	
C*03:17	Cw*0317	—	—	217217, BY00070	AJ635293, AJ635294, DQ244127, DQ144128	CK Hurley^b^
C*03:18	Cw*0318	—	—	NT00511	AY607027, AY607028	
C*03:19	Cw*0319	Cw3	—	243379, MIBe, 14905442, HN-3182240	AJ876752, AJ879090, AM087019, FJ875638	T Lebedeva, A Dormoy, J Rowlands^b^, Histogenetics^b^
C*03:20N	Cw*0320N	Null	—	CTM5745799	DQ188807	JL Vicario
C*03:21	Cw*0321	—	—	BY00074, BY00570	DQ244133, DQ244134, GU256003	(88), CK Hurley^b^
C*03:22Q	Cw*0322Q	—	—	C3Var	AM157129	(285)
C*03:23	Cw*0323	—	—	TBC56206	AB247153	M Satake
C*03:24	Cw*0324	Cw3	—	VTIS87520	DQ400530, DQ400531	BD Tait
C*03:25	Cw*0325	—	—	M2005-00321, M2005-00575	AY965259, AY965250, AY965261, AY965262	TM Williams
C*03:26	Cw*0326	Cw10(w3)	—	TBC59902, HN-38895-2	AB253624, FJ594630	M Satake, Histogenetics^b^
C*03:27	Cw*0327	—	—	NT00672	DQ648006	(271)
C*03:28	Cw*0328	Cw10(w3)	—	TBC60311, HN-75682-8	AB257505, FJ875635	M Satake, Histogenetics^b^
C*03:29	Cw*0329	—	—	TBC34836	AB257506	M Satake
C*03:30	Cw*0330	—	—	MT-10-06	DQ780571, DQ784564	OJ Kwon, M Yu
C*03:31	Cw*0331	—	—	380047	AM384883	(286)
C*03:32	Cw*0332	—	—	NT00678, HN-82153-9	DQ984199, FJ600656	CK Hurley, Histogenetics^b^
C*03:33	Cw*0333	—	—	M268N	AM408917	(278)
C*03:34	Cw*0334	—	—	TBC62026	AB274957	M Satake
C*03:35	Cw*0335	—	—	261327, 237313, HN-41088-7	DQ924972, AM422965, FJ600668	K Hirv, T Lebedeva^b^, Histogenetics^b^
C*03:36	Cw*0336	—	—	277287, HN-01171-8	AM422976, FJ594632	(268), Histogenetics^b^
C*03:37	Cw*0337	—	—	250072	AM422970	(268)
C*03:38:01	Cw*033801	—	—	280091, NT00999, HN-03300-1	AM422980, EU872417, FJ614586	(268), CK Hurley^b^, Histogenetics^b^
C*03:38:02	Cw*033802	—	—	200737, BY00571	EU005272, GU256004	(287), CK Hurley^b^
C*03:39	Cw*0339	Cw3	—	P973	EF422359	C Wang
C*03:40	Cw*0340	Cw3	—	SZ-7, HN-33303-0, SZ-105	EF453695, FJ600670, FJ973625	(288), Histogenetics^b^, HY Zou^b^
C*03:41	Cw*0341	—	—	K16155	AB300723	E Maruya
C*03:42	Cw*0342	—	—	LUMC-C8, HN-00342-4, HN-82778-1, HN-81801-7, HN-67558-1, HN-30063-0	AM746340, FJ594625, FJ594629, FJ594635, FJ600669, FJ875633	(64), Histogenetics^b^
C*03:43:01	Cw*034301	—	—	LUMC-C11, HN-62928-0	AM746341, FJ875636	(64), Histogenetics^b^
C*03:43:02	Cw*034302	—	—	APTGar	FM865853	A Dormoy
C*03:44	Cw*0344	—		LUMC-C20, 71156, HN-40784-0, HN-35750-8, HN-10455-3, HN-54110-4, HN-77802-1, HN-80741-6, HN-72263-1, HN-99786-3, HN-15878-1, HN-86144-4, HN-57227-2, HN-46636-8, HN-46637-6, HN-46669-9, HN-70518-7, HN-72921-1, HN-21520-3, HN-57401-3, HN-89027-8, HN-72796-7, HN-30607-7, HN-18426-8, HN-80871-9, HN-11657-3, HN-28840-5	AM746481, AM779475, FJ594626, FJ594627, FJ594628, FJ594631, FJ594633, FJ594634, FJ594636, FJ594637, FJ600655, FJ600657, FJ600659, FJ600660, FJ600661, FJ600662, FJ600663, FJ600665, FJ600666, FJ600667, FJ600658, FJ600664, FJ792514, FJ792515, FJ792516, FJ792518, FJ875632	(64), J Enczmann^b^, Histogenetics^b^
C*03:45	Cw*0345	—	—	RA1243	EU003583	M Yu
C*03:46	Cw*0346	Cw10(w3)	—	UCLADNAExt#466	EF650024	(289)
C*03:47	Cw*0347	—	—	NT00748	EU256491	(85)
C*03:48	Cw*0348	—	—	SZ-10	EU442891	(290)
C*03:49	Cw*0349	—	—	UD130000	EU669571	(291)
C*03:50	Cw*0350	—	—	25089	AB439293	Y Kuroda
C*03:51	Cw*0351	—	—	HN-11322-9, HN-47650-5, HN-90446-6	FJ594513, FJ554593, FJ619427	Histogenetics
C*03:52	Cw*0352	—	—	HN-92965-7	FJ594520	Histogenetics
C*03:53	Cw*0353	—	—	HN-25274-5, HN-32447-5, HN-35684-9, HN-90575-2, HN-45534-0	FJ594521, FJ554591, FJ554592, FJ554604, GQ900584	Histogenetics
C*03:54	Cw*0354	—	—	HN-64893-3	FJ594529	Histogenetics
C*03:55	Cw*0355	—	—	HN-21141-7, HN-84164-4, HN-33333-1	FJ594535, FJ554602, GQ401226	Histogenetics
C*03:56	Cw*0356	—	—	HN-51959-5, HN-61018-8, HN-13606-8	FJ594539, FJ594541, GQ149300	Histogenetics
C*03:57	Cw*0357	—	—	BY0450	FJ797349	CK Hurley
C*03:58	Cw*0358	—	—	BY00444	FJ797358	CK Hurley
C*03:59	Cw*0359	—	—	BY00461	FJ976697	CK Hurley
C*03:60	Cw*0360	—	—	NT01056	FJ976700	CK Hurley
C*03:61	Cw*0361	—	—	SZ-32	GQ140233	(292)
C*03:62	Cw*0362	—	—	LUMC-C62, 09d02439	FN544087	JDH Anholts
C*03:63	Cw*0363	—	—	HN-92008-1, HN-76315-0	FJ392190, FJ554609	Histogenetics
C*03:64	Cw*0364	—	—	HN-87585-5, HN-11079-3	FJ392197, GQ491096	Histogenetics
C*03:65	Cw*0365	—	—	HN-70463-9, HN-74817-2	FJ538265, FJ554594	Histogenetics
C*03:66	Cw*0366	—	—	HN-57549-0	FJ554595	Histogenetics
C*03:67	Cw*0367	—	—	HN-23272-7	FJ554607	Histogenetics
C*03:68	Cw*0368	—	—	HN-83814-3	FJ554610	Histogenetics
C*03:69	Cw*0369	—	—	HN-4891-8	FJ554612	Histogenetics
C*03:70	Cw*0370	—	—	HN-38623-4	FJ538261	Histogenetics
C*03:71	Cw*0371	—	—	HN-80024-4	FJ538263	Histogenetics
C*03:72	Cw*0372	—	—	HN-93775-5	FJ838264	Histogenetics
C*03:73	Cw*0373	—	—	HN-3024551	FJ392229	Histogenetics
C*03:74	Cw*0374	—	—	HN-09232-0, HN-36900-8	FJ594504, GU128026	Histogenetics
C*03:75	Cw*0375	—	—	HN-23957-4	FJ594507	Histogenetics
C*03:76	Cw*0376	—	—	HN-56900-4	FJ594509	Histogenetics
C*03:77	Cw*0377	—	—	HN-75897-9	FJ765749	Histogenetics
C*03:78	Cw*0378	—	—	HN-38248-9	FJ765876	Histogenetics
C*03:79	Cw*0379	—	—	HN-51098-8, HN-22447-9	FJ858899, GQ994083	Histogenetics
C*04:01:01:01	Cw*04010101	Cw4	C4J1, BeWo C.1	C1R, KSE, BeWo, CJO-A, 39726, MHHZ-00013039, UCLA1999#105, Caki-1, 08009387	M84386, X58536, D83030, AJ238694, AJ292559, M26432, AJ617511, AJ879943, AJ558126, AJ558127, AJ557904, AJ557935, AJ557966, AJ557997, AY918170, FJ515901	R Blasczyk^b^, (293)^b^, S Adams^b^, Y Xu^b^
C*04:01:01:02	Cw*04010102	Cw4	—	Tersaki EXT40	AJ278494	
C*04:01:01:03	Cw*04010103	Cw4	—	08009370	FJ515899	(294)
C*04:01:02	Cw*040102	Cw4	Cw*04N	RN1238C	AF002271, AF017322	
C*04:01:03	Cw*040103	Cw4	—	UCLA-DNA#253	AB196345	M Satake
C*04:01:04	Cw*040104	Cw4	—	NEE138	AM180722	(276)
C*04:01:05	Cw*040105	Cw4	—	x47	EU369696	(295)
C*04:01:06	Cw*040106	Cw4	—	440033	EU382733	(296)
C*04:01:07	Cw*040107	Cw4	—	HN-44391-1	FJ594536	Histogenetics
C*04:01:08	Cw*040108	Cw4	—	HN-46140-0, HN-80972-2, HN-98875-5, HN-23236-9, HN-34103-4, HN-62461-4, HN-69805-2, HN-03781-6, AKB-651323	FJ594538, FJ619422, FJ875613, GQ401215, GQ900576, GQ900580, GU017945, GU180269, FN430727	Histogenetics, R Blasczyk^b^
C*04:01:09	Cw*040109	Cw4	—	HN-4383123, HN-57446-9	EU887014, GQ401227	Histogenetics
C*04:01:10	Cw*040110	Cw4	—	J-08-7194, HN-89705-7, HN-94570-0, HN-25425-2, HN-63876-1, HN-93035-2, HN-81046-9, HN-92882-9, HN-94285-3, HN-79793-7, HN-24206-6, HN-29226-6, HN-28626-5, HN-70551-5, HN-01633-5, HN-74313-0, HN-81687-4, HN-41497-5, HN-22340-3, HN-13432-6, HN-22063-7, HN-15468-3, HN-11404-8, HN-80358-7, HN-63809-2, HN-03817-1	FJ655417, FJ392198, FJ392201, FJ392215, FJ392225, FJ489880, FJ594519, FJ614590, FJ614600, FJ619428, FJ765750, FJ875621, FJ875626, FJ765921, FJ765997, FJ976853, GQ180388, GQ254380, GQ401210, GQ401217, GQ449656, GU128038, GQ161049, GQ180259, GQ180270, GQ254388	(297), Histogenetics^b^
C*04:01:11	Cw*040111	Cw4	—	NT01114, HN-05538-3	GQ985505, GQ401211	CK Hurley, Histogenetics^b^
C*04:01:12	Cw*040112	Cw4	—	HN-24278-8	FJ594516	Histogenetics
C*04:01:13	Cw*040113	Cw4	—	HN-27574-4, HN-68911-4, HN-26496-7, HN-68703-0, HN-17952-4	FJ619434, FJ765994, FJ766001, GU017944, GU128022	Histogenetics
C*04:01:14	Cw*040114	Cw4	—	HN-769287	FJ792490	Histogenetics
C*04:01:15	Cw*040115	Cw4	—	93065477, HN-73887-7	GU120093, GQ994086	D Fuerst, Histogenetics^b^
C*04:03	Cw*0403	—	Cw4NM, Cw4x6	KWO010, 75505703	L54059, GQ472837	Y Xu^b^
C*04:04:01	Cw*040401	—	rn126C, Cw*0401new	rn126C, NM157, NM187, CS00015	U88251, AF017323, U96786, U96787, GQ292547	K Cao^b^
C*04:04:02	Cw*040402	—	—	033924383	AY429603, AY429604	
C*04:05	Cw*0405	—	Cw*0401New	NM2602	AF036556, AF036557	
C*04:06	Cw*0406	—	TREC1, Cw4x6	DM4, MP3	AF062587, AF062588, AF076476	
C*04:07	Cw*0407	Cw4^c^	Cw*0401Variant	ML1805, NT01048	AJ133475, AJ133476, FJ976704	CK Hurley^b^
C*04:08	Cw*0408	—	Cw*04new	NMDP-0196-1628-3, NT01051	AF284582, AF284583, FJ976708	CK Hurley^b^
C*04:09N	Cw*0409N	Null	Cw4New	CTM6991383, LCL13W09501, 38279, 38554	AF196489, AF405691, AJ515217, AJ515489, AJ617512	
C*04:10	Cw*0410	Cw4	—	VTIS64141	AF525407, AF525408	
C*04:11	Cw*0411	—	—	GN00426	AY217668, AY217669	
C*04:12	Cw*0412	—	—	9032426	AY368503, AY368504	
C*04:13	Cw*0413	—	—	214501, 226865, 230908, NT00993	AJ616764, AJ616765, AJ620461, AJ620462, EU847244	CK Hurley^b^
C*04:14	Cw*0414	—	—	221174	AJ616766, AJ616767	
C*04:15:01	Cw*041501	—	—	229707	AJ617785, AJ617786	
C*04:15:02	Cw*041502	—		NT00673	DQ648008	CK Hurley
C*04:16	Cw*0416	—	Cw*04MVE1204	MHHZ-00013010, MHHZ-00013011, MHHZ-00013012	AJ871635	R Blasczyk
C*04:17	Cw*0417	—	—	NT00593	DQ086798, DQ086799	(271)
C*04:18	Cw*0418	—	—	MHHNEE110	AM180629	(276)
C*04:19	Cw*0419	—	—	06-00632	DQ453525	C Moehlenkamp
C*04:20	Cw*0420	Cw4	—	VTIS143819	DQ417108, DQ417109	BD Tait
C*04:23	Cw*0423	—	—	CB4737	DQ788797	W Dong
C*04:24	Cw*0424	—	—	A016658	DQ788798	W Dong
C*04:25	Cw*0425	—	—	238305	AM422966	T Lebedeva
C*04:26	Cw*0426	—	—	266302	AM489408	(268)
C*04:27	Cw*0427	—	—	277012	AM422974	(268)
C*04:28	Cw*0428	—	—	LUMC-C26	AM748048	(64)
C*04:29	Cw*0429	—	—	70214	AM747469	(298)
C*04:30	Cw*0430	—	—	LUMC-31	AM849814	(64)
C*04:31	Cw*0431	—	—	7280167	AM849469	S Schwab
C*04:32	Cw*0432	—	—	MHHC-602813	AM931046	R Blasczyk
C*04:33	Cw*0433	—	—	LUMC-C51, HN-02072-7, HN-02226-8, HN-14161-5	AM991287, FJ619426, FJ976848, FJ976852	(64), Histogenetics^b^
C*04:34	Cw*0434	—	—	41718	FM164941	(280)
C*04:35	Cw*0435	—	—	8200445	EU445578	A Vigh
C*04:36	Cw*0436	—	—	8041801	EU874896	(299)
C*04:37	Cw*0437	—	—	8130104, HN-70426-3	FM212459, FJ618912	A Wölpl, Histogenetics^b^
C*04:38	Cw*0438	—	—	08d05807, 08d04850, 08d04817, 08d05359	FM995500	JDH Anholts
C*04:39	Cw*0439	—	—	HN-46107-9, HN-56645-7, HN-62534-5, HN-80328-7	FJ594537, GQ345077, GQ345080, GQ240486	Histogenetics
C*04:40	Cw*0440	—	—	NT01040, HN-742987	FJ797362, FJ792499	CK Hurley, Histogenetics^b^
C*04:41	Cw*0441	—	—	BY00470	FJ985988	CK Hurley
C*04:42	Cw*0442	—	—	50.1	GQ365192	(300)
C*04:43	Cw*0443	—	—	HN-38325-6, HN-38328-0, HN-01753-2	FJ392196, FJ619425, FJ875581	Histogenetics
C*04:44	Cw*0444	—	—	HN-86445-4	FJ392200	Histogenetics
C*04:45	Cw*0445	—	—	HN-63313-5, HN-33261-1	FJ392226, FJ594527	Histogenetics
C*04:46	Cw*0446	—	—	HN-52837-2	FJ594508	Histogenetics
C*04:47	Cw*0447	—	—	HN-20551-2, HN-11600-8, HN-23641-3, HN-60806-9	FJ594515, FJ618934, FJ976832, GQ859574	Histogenetics
C*04:48	Cw*0448	—	—	HN-26083-7, HN-50961-3	FJ614622, GQ254395	Histogenetics
C*04:49	Cw*0449	—	—	328333	FN430611	T Lebedeva
C*04:50	Cw*0450	—	—	HN-1252167	FJ392211	Histogenetics
C*04:51	Cw*0451	—	—	HN-9832463	FJ392221	Histogenetics
C*04:52	Cw*0452	—	—	HN-90881-3, HN-25199-0	FJ640585, GQ994087	Histogenetics
C*04:53	Cw*0453	—	—	HN-0650688	FJ792488	Histogenetics
C*05:01:01:01	Cw*05010101	Cw5	Cw5N	QBL, RC, JME, QBL, LB129-SCLC, QBL	M58630, L24491, D64148, D83742, AJ010748, Y18146, AJ420244, BX322539	S Beck^b^
C*05:01:01:02	Cw*05010102	Cw5	—	SSTO, 7550800776	BX248310, GQ895733	S Beck, Y Xu^b^
C*05:01:02	Cw*050102	Cw5	—	130269	AJ810176	
C*05:01:03	Cw*050103	Cw5	—	9600958	AF538310, AF541997	TM Williams
C*05:01:04	Cw*050104	Cw5	—	LUMC-C40	AM922328	(64)
C*05:01:05	Cw*050105	Cw5	—	CS00021	GQ325308	K Cao
C*05:01:06	Cw*050106	Cw5	—	VIBU601426AN	FN432836	SGE Marsh
C*05:01:07	Cw*050107	Cw5	—	HN-86342-3, HN-90539-8, HN-28304-6	FJ392203, FJ614609, GU017961	Histogenetics
C*05:01:08	Cw*050108	Cw5	—	HN-3736-3, HN-04402-3, HN-47132-3, HN-62608-4, HN47465-9, HN80863-4, HN-26042-3, HN-79718-7, HN-81198-4, HN-06390-8, HN-50218-6, HN-N257957, HN-35804-0, HN-62913-6, HN-65756-1	FJ618922, FJ614611, FJ765924, FJ969925, FJ969926, FJ969960, FJ969931, FJ969934, FJ969936, GQ345073, GQ449654, GQ859556, GQ859563, GQ859564, GU128049	Histogenetics
C*05:01:09	Cw*050109	Cw5	—	HN-56594-4	FJ614591	Histogenetics
C*05:01:10	Cw*050110	Cw5	—	HN-35223-6	FJ614597	Histogenetics
C*05:01:11	Cw*050111	Cw5	—	329141	FN422380	T Lebedeva
C*05:03	Cw*0503	—	Cw*05DZ	BB90-MEL	AF168611	
C*05:04	Cw*0504	—	Cw5New	CTM-4990904	AF173007, AF173008	
C*05:05	Cw*0505	—	—	609648	AJ440717, AJ440718	
C*05:06	Cw*0506	—	—	LB63359, LB63360	AJ506196, AJ506197	
C*05:07N	Cw*0507N	Null	—	033940693, 17382, BY00147	AY429605, AY429606, AJ889916, EF422082	C Vilches^b^, CK Hurley^b^
C*05:08	Cw*0508	—	—	NT00505, 30393S	AY429724, AY429725, AY509614	
C*05:09	Cw*0509	—	Cw5New, Cw*0502	CTM-5957411, 223724, CTM2682049, CS00022	AF047366, AF047367, AJ616768, AJ616769, AY973960, GQ387160	JL Vicario^b^, K Cao^b^
C*05:10	Cw*0510	—	—	233585, NT01000	AJ635297, AJ635298, EU972419	CK Hurley^b^
C*05:11	Cw*0511	—	—	9795279	AY927414, AY927415	TM Williams
C*05:12	Cw*0512	—	—	NT00625	DQ289051, DQ289052	(271)
C*05:13	Cw*0513	—	—	255461	DQ327710	K Hirv
C*05:14	Cw*0514	—	—	NT00667, BY00114	DQ535033, DQ535035	(271)
C*05:15	Cw*0515	—	—	255961	DQ885883	K Hirv
C*05:16	Cw*0516	—	—	BY00144	EF173478	(85)
C*05:17	Cw*0517	—	—	C138944	EF650025	(301)
C*05:18	Cw*0518	—	—	109342	AM931012	J Enczmann
C*05:19	Cw*0519	—	—	LUMC-C42	AM932285	(64)
C*05:20	Cw*0520	—	—	303305-C5new	AM980450	T Lebedeva
C*05:21	Cw*0521	—	—	8004131	FJ041909	A Vigh
C*05:22	Cw*0522	—	—	413358	FM213457	T Gervais
C*05:23	Cw*0523	—	—	FRFLY15266	FM865852	A Dormoy
C*05:24	Cw*0524	—	—	DEDKM-2421974, HN-37600-3, HN-37480-6	FM992687, FJ969924, GQ914782	(302), Histogenetics^b^
C*05:25	Cw*0525	—	—	SCU-505	FJ750479	(127)
C*05:26	Cw*0526	—	—	HN-08932-7, N-671307, HN-08336-1	FJ618925, FN430728, GQ240509	Histogenetics, R Blasczyk^b^
C*05:27	Cw*0527	—	—	HN-60008-0	FJ594511	Histogenetics
C*05:28	Cw*0528	—	—	HN-79327-7, HN-51622-5	FJ594518, GQ254377	Histogenetics
C*05:29	Cw*0529	—	—	HN-13240-6, HN-14146, HN-14147, HN-54907-5	FJ614608, GQ410228, GQ401229, GQ180263	Histogenetics
C*05:30	Cw*0530	—	—	HN-43424-5	FJ618930	Histogenetics
C*05:31	Cw*0531	—	—	309439	FN422381	T Lebedeva
C*05:32	Cw*0532	—	—	HN-06590-2, HN-74682-6	FJ619423, FJ969929	Histogenetics
C*05:33	Cw*0533	—	—	HN-76528-1, HN-87304-0	FJ765751, FJ765922	Histogenetics
C*05:34	Cw*0534	—	—	HN-36886-9, HN-75619-4, HN-76641-9, HN-27839-1	FJ392193, FJ875589, FJ969921, GU017940	Histogenetics
C*06:02:01:01	Cw*06020101	Cw6	Cw6(W), C6J1	MS, G088, DJS, JOE, JD, TTU, 10030006, PAT495, DBB, 08009397	M28206, X70857, Z22752, Z22753, Z22754, M28160, D64147, AJ420245, DQ249180, M28206, CR388229, FJ785734	(141)^b^, S Beck^b^, Y Xu^b^
C*06:02:01:02	Cw*06020102	Cw6	—	PAT541, 08009402	DQ249182, FJ785735	(141), Y Xu^b^
C*06:02:03	Cw*060203	Cw6	—	LUMC-C28	AM749672	(64)
C*06:02:04	Cw*060204	Cw6	—	HN-55541-6	FJ392189	Histogenetics
C*06:02:05	Cw*060205	Cw6	—	HN-64431-0, HN-59397-1, HN-06177-5	FJ392202, FJ618911, GQ240473	Histogenetics
C*06:03	Cw*0603	—	—	NM779	AF019567, AF019568	
C*06:04	Cw*0604	—	Cw6V	MA43, MA95, NT01001	AB008136, EU872420	CK Hurley^b^
C*06:05	Cw*0605	Cw6	Cw*06NF	NF	AF105240, AF105241	
C*06:06	Cw*0606	—	—	675/99, LUMC-C50, NT00988	AJ277100, AJ277101, AJ277102, AJ277103, AM991286, EU847249	JDH Anholts^b^, CK Hurley^b^
C*06:07	Cw*0607	—	Cw*06DKM	DEDKM	AJ293511	
C*06:08	Cw*0608	—	—	2002-3582	AF529190, AF529191	
C*06:09	Cw*0609	—	—	77625, IHW09462	AY158887, AY158888, AY093609, AY093610	
C*06:10	Cw*0610	—	—	40287492	AY354907, AY354908	
C*06:11	Cw*0611	—	—	231949	AJ628741, AJ628742	
C*06:12	Cw*0612	—	—	2005031636, 2005040419	DQ003052, DQ003053	(303)
C*06:13	Cw*0613	—	—	BY00067	DQ086794, DQ086795	(271)
C*06:14	Cw*0614	—	—	VTIS133092, HN-94717-8	EF088199, FJ614578	BD Tait, Histogenetics^b^
C*06:15	Cw*0615	—	—	250288	AM422973	(268)
C*06:16N	Cw*0616N	Null	—	279827	AM422979	(268)
C*06:17	Cw*0617	—	—	SJ280/32	AM285029	(304)
C*06:18	Cw*0618	—	—	7215243, HN-3171-5, HN-20198-1, HN-84253-6, HN-24848-6, HN-82240-5, HN-80013-9, HN-76014-2	EU445577, FJ392218, FJ618926, FJ618932, FJ765753, FJ765760, FJ594526, FJ765759	A Vigh, Histogenetics^b^
C*06:19	Cw*0619	—	—	CDC09302008-2	FJ236989	S Cordovado
C*06:20	Cw*0620	—	—	1835-08	FM958448	F Emmerich
C*06:21	Cw*0621	—	—	HN-72910-6	FJ594524	Histogenetics
C*06:22	Cw*0622	—	—	RV156-101196	EU707573	(305)
C*06:23	Cw*0623	—	—	NT01033, HN-65149-5, HN-31137-6, HN-686424, HN-91462-1, HN-58547-6, HN-10661-9, HN-68335-1, HN-35876-3	FJ797369, FJ392208, FJ618918, FJ792508, GQ468255, GQ491101, GQ900574, GU128024, GQ161071	CK Hurley, Histogenetics^b^
C*06:24	Cw*0624	—	—	SZBM8943	FJ804766	(306)
C*06:25	Cw*0625	—	—	HN-80796-8	FJ392220	Histogenetics
C*06:26	Cw*0626	—	—	HN-58228-9, HN-74025-2, HN-72178-4	FJ618914, GQ180265, GQ254381	Histogenetics
C*06:27	Cw*0627	—	—	HN-5145243, HN-09020-9, HN-28905-8	FJ392204, FJ392213, GQ180431	Histogenetics
C*06:28	Cw*0628	—	—	HN-43890-2, HN-40595-5, HN-65697-3, HN-96000-1, HN-71664-3	FJ614604, FJ765765, FJ554611, GQ240474, GQ254379	Histogenetics
C*06:29	Cw*0629	—	—	OUAL	FN597419	V Dubois
C*07:01:01	Cw*070101	Cw7	—	MF, LCL721, CGM1, COX, PAT135, PAT144, 7550800619	M28207, Z46810, Y16418, D84394, AL662833, DQ249172, DQ249174, GQ472838	H Inoko^b^, (29)^b^, (141)^b^, Y Xu^b^
C*07:01:02	Cw*070102	Cw7	Cw*07New	19323, 7550800303	Y18499, Y18533, Y18534, Y18535, Y18536, AY162382, AY162383, AY162384, AY162385, gq472839	Y Xu^b^
C*07:01:03	Cw*070103	Cw7	—	230187	AJ617783, AJ617784	
C*07:01:04	Cw*070104	Cw7	—	12410767	AF538309, AF541998	J Wu
C*07:01:05	Cw*070105	Cw7	Cw*07NEE1205	ML1954, MHHNEE329	DQ314860, AM180941	(307), (276)
C*07:01:06	Cw*070106	Cw7	—	VTIS141767	DQ417104, DQ417105	BD Tait
C*07:01:07	Cw*070107	Cw7	—	BY00121	DQ782328	(271)
C*07:01:08	Cw*070108	Cw7	—	R37397, HN-97799-4, HN-06262-1	AM749066, FJ600671, FJ614579	H Tran, Histogenetics^b^
C*07:01:09	Cw*070109	Cw7	—	LUMC-C22, 07d01093	AM749670, AM749670	(64), JDH Anholts^b^
C*07:01:10	Cw*070110	Cw7	—	HN-74648-8, HN-00368-1, HN-62495-1	FJ618915, FJ614620, GQ161041	Histogenetics
C*07:01:11	Cw*070111	Cw7	—	HN-44780-4	FJ614602	Histogenetics
C*07:02:01:01	Cw*07020101	Cw7	JY328, Cw7J1, Cw7.5	JY, TID, KOK, WEHO, SAM, 08009370	D38526, Z49112, AJ293016, AY603356, FJ515904	Y Xu^b^
C*07:02:01:02	Cw*07020102	Cw7	—	Terasaki EXT48	AJ293017	
C*07:02:01:03	Cw*07020103	Cw7	—	30393S, PGF, PAT541, 08009410	AY509618, AL671883, DQ249181, FJ785731	(29)^b^, (141)^b^, Y Xu^b^
C*07:02:02	Cw*070202	Cw7	—	LUMC-C44	AM934701	(64)
C*07:02:03	Cw*070203	Cw7	—	LUMC-C54	FM201491	JDH Anholts
C*07:02:04	Cw*070204	Cw7	—	BASTClai, HN-66407-8	FM994937, GQ180390	A Dormoy, Histogenetics^b^
C*07:02:05	Cw*070205	Cw7	—	SZ-23	FJ811898	(308)
C*07:02:06	Cw*070206	Cw7	—	SZBM655	GQ266700	(309)
C*07:02:07	Cw*070207	Cw7	—	HN-21224-9	FJ932188	Histogenetics
C*07:02:08	Cw*070208	Cw7	—	HN-56799-0, HN-16997-8, HN-47366-8, HN-30555-9, HN-78134-6, HN-96617-4, HN-696860, HN-98559-5, HN-71268-4, HN-61034-4, HN-08086-6, HN-84483-3, HN-17069-5, HN-46385-6, HN-74466-9	FJ392191, FJ391199, FJ392228, FJ765755, FJ765755, FJ765757, FJ538258, FJ792504, FJ976872, GQ161056, GQ180204, GQ240475, GQ254376, GQ345069, GQ401218, GQ900573	Histogenetics
C*07:02:09	Cw*070209	Cw7	—	HN-54433-7	FJ392195	Histogenetics
C*07:02:10	Cw*070210	Cw7	—	HN-04243-6, HN-4640038, HN-16894-0, HN-86223-6, HN-34565-8, HN-78126-6, HN-13035-6, HN-95859-1, HN-45647-3, HN-04512-1, HN-25864-1, HN-20592-7	FJ392217, FJ594512, FJ619429, FJ619430, FJ792489, FJ875583, GQ345075, GQ345076, GU017943, GU128018, GQ161070, GQ994089	Histogenetics
C*07:02:11	Cw*070211	Cw7	—	HN-70377-7, HN-29724-2, HN-13689-5	FJ614612, FJ875623, GQ1801446	Histogenetics
C*07:02:12	Cw*070212	Cw7	—	HN-8224910	FJ392227	Histogenetics
C*07:02:13	Cw*070213	Cw7	—	HN-2762021	FJ792505	Histogenetics
C*07:03	Cw*0703	—	HLA-4	—	M11886	
C*07:04:01	Cw*070401	Cw7	Cw7/8v	LB33-MEL, KRO3/4, SSA, 40C, 10050195, 7550800423	U09853, X83394, D49552, U38976, AJ291815, FJ785728	Y Xu^b^
C*07:04:02	Cw*070402	Cw7	—	NDS-HM	AF220290, AF220291, AY064404	CK Hurley^b^
C*07:04:03	Cw*070403	Cw7	—	CTM-0001222	FJ264200	(310)
C*07:05	Cw*0705	Cw7^c^	39C	39C, NT00976	U38975, EU716071	CK Hurley^b^
C*07:06	Cw*0706	Cw7	Cw*07GB	GB92, 7550800507	X97321, FJ785732	(267)^b^
C*07:07	Cw*0707	—	Cw7v	HAUP	Z79751	
C*07:08	Cw*0708	—	RN2157C	RN2157C	AF017330, AF017331	
C*07:09	Cw*0709	—	—	NM388	AF015556, AF015557	
C*07:10	Cw*0710	—	—	NM1279, NT00987	AF038573, AF038574, EU847251	CK Hurley^b^
C*07:11	Cw*0711	—	Cw*0704x	LB129-SCLC	AJ010749	
C*07:12	Cw*0712	Cw7	Cw-0704N	TER#877, TER#878, TER#857	U60217, U60218	
C*07:13	Cw*0713	—	Cw*JFOR	JFOR, PFOR	AF144664, AF144665	
C*07:14	Cw*0714	Cw7	—	14783D3	AJ242661	
C*07:15	Cw*0715	—	—	500900	AF316035, AF316036	
C*07:16	Cw*0716	Cw7	—	NY00000850, E770	AF480614, EF640739	(311)^b^
C*07:17	Cw*0717	—	Cw*07POC	POC, NT00646	AJ506097, AJ506150, AJ506151, DQ401174, DQ401175	CK Hurley^b^
C*07:18	Cw*0718	—	—	T500PC, LUMC-C38	AF509720, AF509721, AF509722, AF509723, AF509724, AF509725, AF509726, AF509727, AM904560	JDH Anholts^b^
C*07:19	Cw*0719	—	U8918.Cw	U8918, NT00991	AY233977, AY233978, AY233979, EU847246	CK Hurley^b^
C*07:20	Cw*0720	—	—	15911, 10943	AJ440716	
C*07:21	Cw*0721	—	—	N1651	AY434499, AY434500	
C*07:22	Cw*0722	—	—	NT00506, NT00609	AY429726, AY429727, DQ135945, DQ135946	(271)^b^
C*07:23	Cw*0723	—	—	219067	AJ616770, AJ616771	
C*07:24	Cw*0724	—	—	227018	AJ616772, AJ616773	
C*07:25	Cw*0725	—	—	218321	AJ628733, AJ628734	
C*07:26	Cw*0726	—	—	24973S, BY00071, 79475	AY509615, DQ244129, DQ244130	CK Hurley^b^, JDH Anholts^b^
C*07:27:01	Cw*072701	—	—	224953	AJ635295, AJ635296	
C*07:27:02	Cw*072702	—	—	BY00265	EU256489	CK Hurley
C*07:28	Cw*0728	—	—	Bougpet, NT00634	AJ566949, AJ566950, DQ334739, DQ334740	CK Hurley^b^
C*07:29	Cw*0729	—	—	AG44	AJ831405, AJ971029	M Sutton^b^
C*07:30	Cw*0730	Cw7	—	688P03	AY929155	(312)
C*07:31	Cw*0731	—	—	28829	AJ878877	EM vd Berg Loonen
C*07:32N	Cw*0732N	Null	—	CTM8689384, NT00597	DQ188806, DQ372911, DQ372912, DQ372913	JL Vicario, CK Hurley^b^
C*07:33N	Cw*0733N	Null	—	NT00594, NT00608	DQ145936, DQ145937, DQ145938, DQ145939	(271)
C*07:35	Cw*0735	—	—	NT00636, HN-79968-5	DQ334743, DQ334744, FJ614570	(271), Histogenetics^b^
C*07:36	Cw*0736	Cw7	—	CTM-3691986	DQ359691	(155)
C*07:37	Cw*0737	Cw7	—	TBC58855	AB248242	M Satake
C*07:38	Cw*0738	Cw7	—	VTIS137941	DQ400514, DQ400515	BD Tait
C*07:39	Cw*0739	—	—	BY00112	DQ514599	(271)
C*07:40	Cw*0740	Cw7	—	2681943, HN-65522-9, HN-29126-1, HN-34146-3, HN-51134-7, NT01115	AM261864, FJ614572, FJ614574, FJ614581, FJ61458, GQ867211	(313), Histogenetics^b^, CK Hurley^b^
C*07:41	Cw*0741	—	—	VEGJC1	AM237285	A Dormoy
C*07:42	Cw*0742	—	—	CTM-0694504	DQ983641	(314)
C*07:43	Cw*0743	—	—	UCLA-DNAExt#364, 250097, BJchenhua	AM402967, AM422971, EF195154	R Blasczyk, (268)^b^, (315)^b^
C*07:44	Cw*0744	Cw7	—	VTIS135203	EF088200	BD Tait
C*07:45	Cw*0745	Cw7	—	TBC62255	AB274958	M Satake
C*07:46	Cw*0746	—	—	6705660, 238921, LUMC-C24, CS00016, HN-51148-6, HN-44896-0, HN-22796-8, HN-31880-0, HN-34176-0, HN-10120-8	AM409243, AM422967, AM748047, FJ614571, FJ614573, FJ614575, FJ614577, FJ614582, FJ614585	(316), T Lebedeva^b^, JDH Anholts^b^, K Cao^b^, Histogenetics^b^
C*07:47	Cw*0747	—	—	257299	AM489406	(268)
C*07:48	Cw*0748	—	—	281146	AM422964	(268)
C*07:49	Cw*0749	Cw7	—	TEER	AM696284	B Hepkema
C*07:50	Cw*0750	—	—	LUMC-C4	AM748044	(64)
C*07:51	Cw*0751	Cw7	—	89, NT01004	EU017384, EU872423	(317), CK Hurley^b^
C*07:52	Cw*0752	—	—	US050018878	AM850146	A Dormoy
C*07:53	Cw*0753	—	—	7260265	AM884150	S Schwab
C*07:54	Cw*0754	—	—	266653	EU410614	J Mytilineos
C*07:55N	Cw*0755N	Null	—	MHHZ-00020178, HN-74337-8	AM945965, FJ875630	R Blasczyk, Histogenetics^b^
C*07:5601	Cw*075601	—	—	BJ53	EU594580	Z Zhang
C*07:5602	Cw*075602	—	—	SZBM033, 300878	FJ804765, FN422382	(318), T Lebedeva^b^
C*07:57	Cw*0757	Cw7	—	CTM-9699551	EU683684	(310)
C*07:58	Cw*0758	—	—	BY00342	EU847250	CK Hurley
C*07:59	Cw*0759	—	—	LUMC-C52	FM178479	JDH Anholts
C*07:60	Cw*0760	—	—	NT01002, HSP-Cw7, HN-50519-9, HN-73447-9, HN-23230-7, HN-83996-1, HN-33890-7, HN-355887-7, HN-77749-01	EU872421, FM201315, FJ618916, FJ618931, FJ976806, FJ976810, FJ976820, FJ976821, FJ976880	CK Hurley, (319)^b^, Histogenetics^b^
C*07:61N	Cw*0761N	Null	—	249317, HN-13959-2	AM980449, FJ875629	T Lebedeva, Histogenetics^b^
C*07:62	Cw*0762	Cw7	—	71572	FM179945	M Danzer
C*07:63	Cw*0763	—	—	UCLA0565	FJ009630	K Cao
C*07:64	Cw*0764	—	—	BY00268	EU275153, EU484047	CK Hurley
C*07:65	Cw*0765	—	—	MSA2, MSD1	FM865869	M Schroeder
C*07:66	Cw*0766	—	—	SZBM02, 7550800383	FJ629179, FJ785729	(320), Y Xu^b^
C*07:67	Cw*0767	—	—	SZBM02, ChengyizeC7, 7550800383	FJ629180, FJ810062, FJ785730	(320), J He^b^, Y Xu^b^
C*07:68	Cw*0768	—	—	NT01039, HN-727525, HN-58409-4, HN-16429-0, HN-58361-9, HN-81747-5, HN-08339-9, HN-74665-8, HN-03908-0, HN-53344-0, HN-28940-6, HN-76839-2, HN-91790-5, HN-40383-4	FJ797363, FJ792498, FJ858901, FJ858902, FJ976822, GQ149302, GQ240566, GQ345090, GQ345093, GU017950, GQ180252, GQ240505, GQ994073, GQ994084	CK Hurley, Histogenetics^b^
C*07:69	Cw*0769	—	—	NT01031, HN-694436	FJ797371, FJ792500	CK Hurley, Histogenetics^b^
C*07:70	Cw*0770	—	—	NT01034, HN-690939	FJ797368, FJ792509	CK Hurley, Histogenetics^b^
C*07:71	Cw*0771	—	—	NT01037	FJ797365	CK Hurley
C*07:72	Cw*0772	—	—	NT01042, NT01035, HN-764015, HN-14296-7, HN-49344-4	FJ797360, FJ797367, FJ792495, FJ997968, GQ401206	CK Hurley, Histogenetics^b^
C*07:73	Cw*0773	—	—	NT01060, 33371, HN-36623-6, HN-01145-2	FJ976690, FN430612, GU128037, GQ240515	CK Hurley, T Lebedeva^b^, Histogenetics^b^
C*07:74	Cw*0774	—	—	C143474	GQ260846	(321)
C*07:75	Cw*0775	—	—	93056022, HN-69363-9, HN-42676-6	GQ375768, FJ619424, FJ976846	D Fuerst, Histogenetics^b^
C*07:76	Cw*0776	—	—	LISU617232AN, HN-38523-1	FN433487, GQ161045	SGE Marsh, Histogenetics^b^
C*07:77	Cw*0777	—	—	HN-75322-8	FJ392206	Histogenetics
C*07:78	Cw*0778	—	—	HN-11891-3	FJ392224	Histogenetics
C*07:79	Cw*0779	—	—	HN-05903-0, HN-62794-3, HN-25815-9, HN-10178-1	FJ554620, FJ875620, FJ858903, FJ976796	Histogenetics
C*07:80	Cw*0780	—	—	HN-64803-1, HN-69866-0, HN-N-251395	FJ618913, FJ765920, GQ240559	Histogenetics
C*07:81	Cw*0781	—	—	HN-33323-8	FJ618920	Histogenetics
C*07:82	Cw*0782	—	—	HN-08575-4	FJ618924	Histogenetics
C*07:83	Cw*0783	—	—	HN-39787-0, HN-66430-1	FJ618928, FJ619433	Histogenetics
C*07:84	Cw*0784	—	—	HN-61981-8	FJ392210	Histogenetics
C*07:85	Cw*0785	—	—	HN-98823-8, HN-24541-5	FJ594544, GQ161031	Histogenetics
C*07:86	Cw*0786	—	—	HN-52422-2	FJ624599	Histogenetics
C*07:87	Cw*0787	—	—	HN-64331-1	FJ614601	Histogenetics
C*07:88	Cw*0788	—	—	HN-46631-7, HN-46632-5	FJ614605, FJ614606	Histogenetics
C*07:89	Cw*0789	—	—	HN-05830-4	FJ614607	Histogenetics
C*07:90	Cw*0790	—	—	HN-85255-9, HN-22024-9	FJ614619, GQ449658	Histogenetics
C*07:91	Cw*0791	—	—	HN-59183-6, HN-39440-4, HN-55690-4, HN-27500-6	FJ618927, GQ161072, GQ180250, GQ240481	Histogenetics
C*07:92	Cw*0792	—	—	356442	FN430613	T Lebedeva
C*07:93	Cw*0793	—	—	LUMC-C65	FN555606	JDH Anholts
C*07:94	Cw*0794	—	—	HN-08894-8	FJ619431	Histogenetics
C*07:95	Cw*0795	—	—	HN-61294-5, HN-86379-4	FJ765746, FJ976843	Histogenetics
C*07:96	Cw*0796	—	—	HN-4723-8	FJ765752	Histogenetics
C*07:97	Cw*0797	—	—	HN-16390-1, HN-58064-0	FJ765763, FJ875622	Histogenetics
C*07:98N	Cw*0798N	Null	—	HN-12269-0	FJ875631	Histogenetics
C*07:99	Cw*0799	—	—	HN-30334-7	FJ392209	Histogenetics
C*08:01:01	Cw*080101	Cw8	C8J1	02627, KNM, SFK, HTS, 26/27, 08009391	M84174, D64151, AJ420246, FJ785724	Y Xu^b^
C*08:01:02	Cw*080102	Cw8	—	SWN8, PU03	AJ438882, AJ438883, AF510721	
C*08:02:01	Cw*080201	Cw8	—	CGM1, LWAGS, WT51, PAT135, PAT218, 7550800507	M59865, M84173, DQ249173, DQ249176, FJ785733	(141)^b^, (267)
C*08:02:02	Cw*080202	Cw8	—	HN-30027-8, HN-47110-8, HN-47121-5	FJ614615, GQ345088, GQ345089	Histogenetics
C*08:02:03	Cw*080203	Cw8	—	311562	FN422383	T Lebedeva
C*08:03:01	Cw*080301	Cw8	C8J2	KRC103, SSK, 7550800777	Z15144, D50854, GQ472840	Y Xu^b^
C*08:03:02	Cw*080302	Cw8	—	BY00447	FJ797353	CK Hurley
C*08:04	Cw*0804	Cw8^c^	—	NM313, NM914, C03, TER#876	U96784, U96785, AF016304, AF009684, U60321, U60322	
C*08:05	Cw*0805	—	Cw*08Var	NEQ2A10/97	Y15842	
C*08:06	Cw*0806	—	—	EC22, NT00990	AF082800, AF082801, EU847247	CK Hurley^b^
C*08:07	Cw*0807	—	Cw*CCAI	CCAI	AF179631, AF179632	
C*08:08	Cw*0808	—	Cw*0801V	CSR	AF245437	
C*08:09	Cw*0809	—	—	Kolla 34	AJ278509	
C*08:10	Cw*0810	—	—	2002-8269, D33202	AY429570, AY429571, AY484702, AY484703	
C*08:11	Cw*0811	—	—	226080	AJ616774, AJ616775	
C*08:12	Cw*0812	—	—	229547	AJ628737, AJ628738	
C*08:13	Cw*0813	—	—	NT00600	DQ105583, DQ105584	(107)
C*08:14	Cw*0814	—	—	TBC56207	AB247154	M Satake
C*08:15	Cw*0815	—	—	LUMC-C27	AM749671	(64)
C*08:16	Cw*0816	—	—	FBCCB5992	AB426906	Y Kuroda
C*08:17	Cw*0817	—	—	C139470	EU850411	(322)
C*08:18	Cw*0818	—	—	306061-C8new	AM980453	T Lebedeva
C*08:19	Cw*0819	Cw8	—	42270	FM177892	(323)
C*08:20	Cw*0820	—	—	SZBM383	FJ481115	(324)
C*08:21	Cw*0821	—	—	SZBM04, 7550800423	FJ6502400, FJ785726	(325), Y Xu^b^
C*08:22	Cw*0822	—	—	08009402	FJ785727	(326)
C*08:23	Cw*0823	—	—	NT01030, HN-680161	FJ797372, FJ792506	CK Hurley, Histogenetics^b^
C*08:24	Cw*0824	—	—	SZ-21	FJ825144	(327)
C*08:25	Cw*0825	—	—	CTM-1003396	FJ868794	(127)
C*08:26N	Cw*0826N	Null	—	NT01061	FJ976689	CK Hurley
C*08:27	Cw*0827	—	—	SZBM270	GQ241930	(328)
C*08:28	Cw*0828	—	—	HN-83480-4, HN-86260-9, HN-08552-2, HN-26339-9, HN-76931-4, HN-89291-8, HN-92569-9, HN-39991-7, HN-13242-5, HN-30318-9, HN-48864-2	FJ392207, FJ554621, FJ614621, FJ858904, FJ976837, FJ976878, GQ161055, GQ468257, GU017957, GQ161061, GQ240491	Histogenetics
C*08:29	Cw*0829	—	—	HN-4810647	FJ392214	Histogenetics
C*08:30	Cw*0830	—	—	347187	FN422384	T Lebedeva
C*08:31	Cw*0831	—	—	081P08, 902P08	FN568089	S Nesci
C*12:02:01	Cw*120201	—	Cb-2	MT	M28172	
C*12:02:02	Cw*120202	—	Cw*1202gyp, C12J1	G085, MSU, AKIBA, E4181324, 7550800375	X70856, D64152, D83741, M21963, D12471, D12472, AJ420247, GQ472841	Y Xu**b**
C*12:02:03	Cw*120203	—	Cw*PBAG	PBAG	AF189725, AF189726	
C*12:02:04	Cw*120204	—	—	HN-5264226	FJ765988	Histogenetics
C*12:03:01:01	Cw*12030101	—	Cw12New, C12J12	D0208915, WDV, YAR, GB002, HNT, JBUSH, PAT144, PAT495, 7550800556	U06695, U06696, X82122, D64146, AJ420248, DQ249175, DQ249179, GQ472842	(141)^b^, (269)^b^
C*12:03:01:02	Cw*12030102	—	—	BGC	AM412763	(329)
C*12:03:02	Cw*120302	—	—	PI151	AF289031	
C*12:03:03	Cw*120303	—	—	232879	AJ629313, AJ629314	
C*12:03:04	Cw*120304	—	—	LEUVio	AM180647	A Dormoy
C*12:03:05	Cw*120305	—	—	AKB4943, HN-55314-8, HN-22751-4	AM849049, FJ614569, FJ614580	S Schwab, Histogenetics^b^
C*12:03:06	Cw*120306	—	—	LUMC-C46	AM946387	(64)
C*12:03:07	Cw*120307	—	—	HN-54935-3	FJ618917	Histogenetics
C*12:03:08	Cw*120308	—	—	HN-37048-5, HN-20583-0, HN-9052770, HN-N252973, HN-38754-6, HN-84987-5	FJ614596, FJ614598, FJ765991, GQ345068, GQ900567, GQ240483	Histogenetics
C*12:03:09	Cw*120309	—	—	HN-98468-9	FJ765762	Histogenetics
C*12:04:01	Cw*120401	—	Sy/9-2	M.H(9-2)	X99704	
C*12:04:02	Cw*120402	—	Cw*12JD,	NDS-JD, NM2018	Y11843, AF015558, AF015559	
C*12:05	Cw*1205	—	Cw12x16	ANDP	Z80228, Z83247	
C*12:06	Cw*1206	—	—	NM1699	AF036552, AF036553	
C*12:07	Cw*1207	—	—	Atuwagu, Atuwaogu	AJ249163, AJ249164	
C*12:08	Cw*1208	—	Cw*12new	10030006	AJ304496	
C*12:09	Cw*1209	—	—	Sgh, Vrk, JAWA2	AJ507431, AJ507432, AJ550622, AJ550623	
C*12:10	Cw*1210	—	—	N039	AY323834	
C*12:11	Cw*1211	—	Cw*12TYP	TYP	AJ579647, AJ579648, AJ579649	
C*12:12	Cw*1212	—	—	224926	AJ628735, AJ628736	
C*12:13	Cw*1213	—	—	009765371, 39529S, BY00065	AY643836, AY643837, AY623606, DQ086790, DQ086791	S Adams^b^, CK Hurley^b^
C*12:14:01	Cw*121401	—	—	225372	AJ635299, AJ635300	
C*12:14:02	Cw*121402	—	—	77-2438-2412	AB441824	C Horie
C*12:15	Cw*1215	—	—	133334	AJ697650	
C*12:16	Cw*1216	—	—	87864, NT00647, HN-72238-2	DQ112223, DQ401180, DQ401081, FJ614584	MS Leffell, (271)^b^, Histogenetics^b^
C*12:17	Cw*1217	—	—	CM924	DQ206990, DQ206991	(283)
C*12:18	Cw*1218	—	—	BY00098	DQ465613	(271)
C*12:19	Cw*1219	—	—	GAGI8381AN, NT01003	AM261030, EU872422	AM Little, CK Hurley^b^
C*12:20	Cw*1220	—	—	ANPA86329AN	AM413042	AM Little
C*12:21	Cw*1221	—	—	BY00148	EF422081	(85)
C*12:22	Cw*1222	—	—	SZ-22	FJ811899	(330)
C*12:23	Cw*1223	—	—	YC040609A, YC040609B	GQ200571	Y Chang
C*12:24	Cw*1224	—	—	JATH473491AN	FN538998	SGE Marsh
C*12:25	Cw*1225	—	—	HN-38781-9	FJ392187	Histogenetics
C*12:26	Cw*1226	—	—	HN-35603-4	FJ392194	Histogenetics
C*12:27	Cw*1227	—	—	HN-06391-8, HN-57019-7, HN-52660-0, HN-74768-3	FJ594532, GQ491103, GQ859576, GQ149306	Histogenetics
C*12:28	Cw*1228	—	—	HN-39457-6	FJ614593	Histogenetics
C*12:29	Cw*1229	—	—	HN-08004-5	FJ619436	Histogenetics
C*12:30	Cw*1230	—	—	HN-20602-2, HN-40116-2, HN-62540-6	FJ619437, FJ976874, FJ976876	Histogenetics
C*12:31	Cw*1231	—	—	HN-23222-7	FJ765764	Histogenetics
C*14:02:01	Cw*140201	—	—	LUY, TC106, LKT2, UCLA1999#074, 08008411, SZ-52	U06487, Z47377, U41386, D49820, M28171, AJ558128, AJ558129, AJ557919, AJ557950, AJ557981, AJ558012, FJ785736, FJ973628	(293)^b^, (281)^b^, HY Zou^b^
C*14:02:02	Cw*140202	—	—	NM1991	AF015554, AF015555	
C*14:02:03	Cw*140203	—	—	UCLA#53/#344, ML1806	AJ535690, AJ535691, EU095650	M Luo^b^
C*14:02:04	Cw*140204	—	—	F227	DQ020589, DQ020590, DQ020591	(331)
C*14:03	Cw*1403	—	Cx44	TID, DK1	D31817, AJ420249	
C*14:04	Cw*1404	—	—	CTM-1986765, NT00992	AF104218, AF104219, EU847245	CK Hurley^b^
C*14:05	Cw*1405	—	Cw*1402v	NMDP0121-0146-5, NT00975	AJ306617, AJ306618, EU716070	CK Hurley^b^
C*14:06	Cw*1406	—	—	UCLA-DNA#217	AB196344	M Satake
C*14:07N	Cw*1407N	Null	—	MHHZ-00009438	AM39493	R Blasczyk
C*14:08	Cw*1408	—	—	ML270	DQ314861	(307)
C*14:09	Cw*1409	—	—	LUMC-C30, 3987, 07d04084	AM849813	(64)
C*14:10	Cw*1410	—	—	NT00989, NT01050	EU847248, FJ976707	CK Hurley
C*14:11	Cw*1411	—	—	319348-C14new, HN-35285-5	AM980455, FJ976841	T Lebedeva, Histogenetics^b^
C*14:12	Cw*1412	—	—	BY00462	FJ976691	CK Hurley
C*14:13	Cw*1413	—	—	HN-11562-7	FJ618919	Histogenetics
C*14:14	Cw*1414	—	—	HN-87423-0, HN-09762-5	FJ614603, GQ254392	Histogenetics
C*14:15	Cw*1415	—	—	HN-12308-0	FJ765923	Histogenetics
C*15:02:01	Cw*150201	—	C*X, Cw*6.2, Cl.9, Cw15J1	AUCA#2, G085, G088, KUE, GM637, BOB, 08009397	L20091, X67818, D83031, M24096, AJ420250, FJ785737	Y Xu^b^
C*15:02:02	Cw*150202	—	Cw*1502new	NM4C376	AF139727, AF139728	
C*15:02:03	Cw*150203	—	—	DED, HN-30696-2, HN-89402-4	AM180648, FJ614576, FJ875637	A Dormoy, Histogenetics^b^
C*15:02:04	Cw*150204	—	—	BJ049, HN-50447-2	EU169936, FJ875634	N Liu, Histogenetics^b^
C*15:02:05	Cw*150205	—	—	HN-50699-6	FJ765998	Histogenetics
C*15:03	Cw*1503	—	—	GRC150	M99388	
C*15:04	Cw*1504	—	Cw*15Sp	C047, 27289	X73518, AM234714	T Gervais^b^
C*15:05:01	Cw*150501	—	Cw*15v	LE023	X78343	
C*15:05:02	Cw*150502	—	Cw*1505v	L7901, 7550800776	X87841, GQ895734	Y Xu^b^
C*15:05:03	Cw*150503	—	Cw*15TER1125	TER1125	AJ579997, AJ579998, AJ579999	
C*15:05:04	Cw*150504	—	—	234615	AJ635367, AJ635368	
C*15:06	Cw*1506	—	Cw*15N	M001C, NM2732, JF	AF002270, AF017324, AF036550, AF036551, Y15745, Y15746, AJ011882, Y15746, Y15745, Y18140, Y18141	
C*15:07	Cw*1507	—	—	PUSPAN, BY00572	Y17064, Y17065, GU256005	CK Hurley^b^
C*15:08	Cw*1508	—	Cw*15P	Peru-15	AJ010322, AJ010323	
C*15:09	Cw*1509	—	Cw*1504New	NM4C159, NT01047	AF165850, AF165851, FJ976706	CK Hurley^b^
C*15:10:01	Cw*151001	—	—	SLGJ	AF302133, AF302134	
C*15:10:02	Cw*151002	—	Cw*1514, HLA-Cw*1520V1	TBC17874	AB196346	M Satake
C*15:11	Cw*1511	—	Cw*KDILL	CBM2598, NT00974	AF335316, AF335317, EU716069	CK Hurley^b^
C*15:12	Cw*1512	—	—	230307	AJ629315, AJ629316	
C*15:13	Cw*1513	—	—	ITGRO, LUMC-C39, SZ-24	AJ851863, AJ851864, AJ851865, AM904561, GQ304757	JDH Anholts^b^, HY Zou^b^
C*15:15	Cw*1515	—	—	NT00598	DQ289053, DQ289054	(271)
C*15:16	Cw*1516	—	—	SHWA39771AN	AM158319	AM Little
C*15:17	Cw*1517	—	—	NT00638, CTM-2693858	DQ354442, DQ354443, DQ833439	(271), A Balas^b^
C*15:18	Cw*1518	—	—	258119	AM489407	(268)
C*15:19	Cw*1519	—	—	280144	AM422981	T Lebedeva
C*15:20	Cw*1520	—	—	280716	AM422983	(268)
C*15:21	Cw*1521	—	—	BJxeuwenlong	EF379939	Z Zhang
C*15:22	Cw*1522	—	—	NT01058	FJ976698	CK Hurley
C*15:23	Cw*1523	—	—	93066088, HN-25224-7, HN-30227-5	GQ979631, FJ875625, GQ240516	D Fuerst, Histogenetics^b^
C*15:24	Cw*1524	—	—	HN-30608-6, HN-28600-9	FJ765756, FJ976807	Histogenetics
C*15:25	Cw*1525	—	—	G18106156010P	GU133628	D Niokou
C*15:26	Cw*1526	—	—	SZBM685	GU232859	Z Deng
C*16:01:01	Cw*160101	—	Cl.10	GM637, TC106, PITOUT, MANN	M24097, U41420, U56259, U56260, AJ420251, BX927178	S Beck^b^
C*16:01:02	Cw*160102	—	—	12762-SN	AJ865288	
C*16:01:03	Cw*160103	—	—	0502962, 0502914, 0502922	EF469770	E Palou
C*16:01:04	Cw*160104	—	—	HN-751293, HN-4604700, HN-N264212	FJ792491, FJ792511, GQ994077	Histogenetics
C*16:02:01	Cw*160201	—	Cw*16v	C073, 37771	X76189, AM419439	T Gervais^b^
C*16:02:02	Cw*160202	—	—	HN-758777	FJ792492	Histogenetics
C*16:04:01	Cw*160401	—	rn183C, wt30L	BOJ, rn183C, wt30C, NM290, NM633, 4136, 7550800701	Z75172, U88252, AF017326, U88253, AF017325, U96788, U96789, AJ011883, Y18657, Y18658, Y18659, Y18139, GQ472843	Y Xu^b^
C*16:06	Cw*1606	—	—	CTBT-1	AJ537578	
C*16:07	Cw*1607	—	—	NT00592	DQ086796, DQ086797	(271)
C*16:08	Cw*1608	—	—	NT00599	DQ105585, DQ105586	(107)
C*16:09	Cw*1609	—	—	E355	DQ916147	(332)
C*16:10	Cw*1610	—	—	IM0600637	EU085530	E Palou
C*16:11	Cw*1611	—	—	7221287	EU431984	J Mytilineos
C*16:12	Cw*1612	—	—	7217636	EU445576	A Vigh
C*16:13	Cw*1613	—	—	HN-68928-4, HN-02519-1	FJ594523, FJ618929	Histogenetics
C*16:14	Cw*1614	—	—	HN-08233-7, HN-83251-3, HN-99449-3, HN-65879-2	FJ594545, FJ618933, FJ618923, GQ180444	Histogenetics
C*16:15	Cw*1615	—	—	HN-02524-8, HN-19152-0, HN-10995-9, HN-25373-9	FJ392219, FJ976823, GQ180434, GQ254384	Histogenetics
C*16:16	Cw*1616	—	—	HN-75443-4, HN-43823-8	FJ594530, GQ161050	Histogenetics
C*16:17	Cw*1617	—	—	HN-87906-5	FJ614616	Histogenetics
C*16:18	Cw*1618	—	—	HN-44614-8	FJ765758	Histogenetics
C*16:19	Cw*1619	—	—	HN-3034950	FJ792512	Histogenetics
C*17:01:01:01	Cw*17010101	—	Cw16New	RSH, GB86, BM21, 98807B	U06835, X98742, Y10520, AJ420252, AJ558130, AJ558131, AJ558132, AJ557924, AJ557955, AJ557986, AJ558017	R Blasczyk^b^
C*17:01:01:02	Cw*17010102	—	—	7550800440	GQ472844	Y Xu
C*17:01:02	Cw*170102	—	—	HN-727152	FJ792497	Histogenetics
C*17:01:03	Cw*170103	—	—	HN-679965, HN-69216-8	FJ792510, FJ875596	Histogenetics
C*17:02	Cw*1702	—	Cw17N	KSU	D64149	
C*17:03	Cw*1703	—	Cw*17New	17767	Y18537, Y18538, Y18539, Y18540, Y18541	
C*17:04	Cw*1704	—	—	NT00602	DQ135947, DQ135948	(107)
C*17:05	Cw*1705	—	Cw*17new	CTM-0890009	FJ434674	(310)
C*17:06	Cw*1706	—	—	HN-72806-2	FJ765996	Histogenetics
C*18:01	Cw*1801	—	Cw*04GB, Cw4x6	GB92, DIJL, TERASAKI926	X96582, Z80227, AJ420253	
C*18:02	Cw*1802	—	Cw*18GB	GB32	Y09156	
C*18:03	Cw*1803	—	—	280415	AM422982	(268)
						
E*01:01:01:01	E*01010101	—	JTW15, HLA-6.2	JT (JOE), YN, HF, SPAARN70, LCL721.45, JHUAA0393, JHUAA0394, JHUAA0396, JHUAA0527, JHUAA0528, JHUAA0529, JHUAA0530, IHW01141, IHW01143, IHW01152, IHW01173, IHW01175, IHW01182, IHW01184, HAN19-02, HAN19-04, HAN19-05, LBF, STEINLIN, ARBO, 1199, COX, RPC15, QBL, DBB, SSTO	M20022, L78934, AJ715787, AJ293264, M21533, AY645724, AY645725, AY645726, AY645728, AY645729, AY645730, AY645732, AY645734, AY645735, AY645739, AF523275, AF523276, AF523281, AF523283, AL662822, AB014080, AL844213, CR762481, BX936369	(333)^b^, (29)^b^, (334)^b^, (105)^b^, S Beck^b^
E*01:01:01:02	E*01010102	—	—	WT24	AF523277	(333)
E*01:01:01:03	E*01010103	—	—	LKT3	AB088094	H Inoko
E*01:03:01:01	E*01030101	—	M32507, E*01C230	MT, MH, TK, SPAARN70, CHI009, JFE, CR, H58002, JHUAA0527, IHW01143, IHW01173	M32507, L78455, X87678, X87679, L78455, AJ002533, AJ002534, AY216681, AY645731, AY645733, AY645736	(333)^b^
E*01:03:01:02	E*01030102	—	—	1471	AF523282	(333)
E*01:03:02	E*010302	—	E*01T230	MSC, CHI004, 17771, JHUAA0393, JHUAA0396, IHW01181, IHW01182, SCHU, WDV, BM15, MT14B, HAN19-01, HAN19-04, HAN19-05, PGF, MANN	X87680, X87681, L79943, AY645727, AY645737, AY645740, AY645741, AF523274, AF523278, AF523279, AF523280, AL662873, CR382280	(333)^b^, (29)^b^, S Beck^b^
E*01:03:03	E*010303	—	—	CD	AJ293263	
E*01:03:04	E*010304	—	—	IHW01175, IHW01181	AY645738	(333)
E*01:04	E*0104	—	M32508	KS	M32508	
						
F*01:01:01:01	F*01010101	—	HLA-5.4	LCL721.144, H58002, WT24, WDV	X17093, AY253270, AY253271, AF523287, AF523288	(333)^b^
F*01:01:01:02	F*01010102	—	—	595	AF523295	(333)
F*01:01:01:03	F*01010103	—	—	JHUAA0394, JHUAA0396, IHW01141, HAN19-01, HAN19-04	AY645742, AY645750, AY645758	(333)
F*01:01:01:04	F*01010104	—	—	IHW01175, IHW01184, HAN19-01, HAN19-05	AY645753, AY645757	(333)
F*01:01:01:05	F*01010105	—	—	HAN19-02, HAN19-04, HAN19-05	AY645759	(333)
F*01:01:01:06	F*01010106	—	—	JHUAA0528	AY645746	(333)
F*01:01:01:07	F*01010107	—	—	IHW01141, IHW01152	AY645751	(333)
F*01:01:01:08	F*01010108	—	—	QBL, BOLETH, DBB, MANN	AL844851, AF055066, CR753818, BX927250	(105), (42)^b^, S Beck^b^
F*01:01:02:01	F*01010201	—	—	H58002, H58001, JHUAA0393	AY216682, AY253269, AY645743	(333)^b^
F*01:01:02:02	F*01010202	—	—	JHUAA0394	AY645745	(333)
F*01:01:02:03	F*01010203	—	—	IHW01175, IHW01181	AY645756	(333)
F*01:01:02:04	F*01010204	—	—	1471	AF523294	(333)
F*01:01:02:05	F*01010205	—	—	JHUAA0393, JHUAA0396, JHUAA0527	AY645744, AY645749	(333)
F*01:01:03:01	F*01010301	—	—	LKT3, LBF, STEINLIN, BM15, COX, RPC15	AB088082, AF523285, AF523286, AF523289, AL669813, AB023058	H Inoko, (333), (29)^b^, (334)^b^
F*01:01:03:02	F*01010302	—	—	IHW01143, IHW01152	AY645752	(333)
F*01:01:03:03	F*01010303	—	—	MT14B	AF523290	(333)
F*01:01:03:04	F*01010304	—	—	435, 1350, 1199	AF523293, AF523296, AF523297	(333)
F*01:02	F*0102	—	—	JHUAA0527, JHUAA0529, JHUAA0530	AY645747	(333)
F*01:03:01:01	F*01030101	—	—	SCHU, HOM-2, ARBO, PGF	AF523284, AF523291, AF523292, AL645939	(333), (29)^b^
F*01:03:01:02	F*01030102	—	—	JHUAA0528, JHUAA0529, JHUAA0530, IHW01173, IHW01182, IHW01184	AY645748, AY645754	(333)
F*01:04	F*0104	—	—	IHW01181, IHW01182	AY645755	(333)
						
G*01:01:01:01	G*01010101	—	HLA-6.0, G*I, GCO1	LCL721.144, ASR53, MOU, SPO010, YRK, HT68, IHW01141, IHW01182, IHW01181, IHW01175, IHW01184, HAN19-01, HAN19-05, DBB, MCF, BCI-08, BCI-09, BCI-13, BCI-17, BCI-18, CUI-61	J03027, X17273, L27836, L27837, D77998, D77999, D78000, U76216, U76217, AY645768, AY645770, AY645771, AY645774, CR759769, CR788234, CT009517, GQ996557, GQ996563	(333)^b^, S Beck^b^, J Martinez-Laso^b^
G*01:01:01:02	G*01010102	—	—	JHUAA0394, JHUAA0396	AY645762	(333)
G*01:01:01:03	G*01010103	—	—	HAN19-02, HAN-19-04	AY645775	(333)
G*01:01:01:04	G*01010104	—	—	JHUAA0393, MANN	AY645760, BX927171	(333), S Beck^b^
G*01:01:01:05	G*01010105	—	—	JHUAA0393, JHUAA0396, JHUAA0528, JHUAA0529, JHUAA0530, PGF, BCI-10, CUI-61, CUI-80	AY645761, AY645764, AF523298, AL645929, GQ996565	(333), (29)^b^, J Martinez-Laso^b^
G*01:01:01:06	G*01010106	—	—	CUI-79	FJ449755	(335)
G*01:01:02:01	G*01010201	—	BeWo G7, G*II, GJ2, GCO2	BeWo, COX, DHIF, WT47, STK, HT43, TB250, IHW01141, IHW01143, IHW01152, COX, RPC15, QBL, BOLETH, BCI-13, BCI-14, BCI-19, CUI-80	M32800, X60983, L07784, L41392, D85032, D67009, D67010, D67011, U65245, U65246, U88244, AY645769, AL671561, AB023057, BX001005, AF055066, GQ996569, GQ996566	(333)^b^, (29)^b^, (334)^b^, (105)^b^, (42)^b^, J Martinez-Laso^b^
G*01:01:02:02	G*01010202	—	—	JHUAA0528, HAN19-01, HAN19-04	AY645766, AY645776	(333)
G*01:01:03:01	G*01010301	—	G*IV, GJ4, GCO5	BeWo, KKH, HT147, CUI-65	L07784, L41363, D67003-5, D85033, U65235, U65236, FJ805838	(336)^b^
G*01:01:03:02	G*01010302	—	—	CUI-65	FJ805839	(336)
G*01:01:04	G*010104	—	G*0101d, GCO4	HT180	U65233, U65234	
G*01:01:05	G*010105	—	CEPH G1	1305	U58024	
G*01:01:06	G*010106	—	CEPH G5	2702, JHUAA0394	U58027, AY645763	(333)^b^
G*01:01:07	G*010107	—	CEPH G6	3101	U58028	
G*01:01:08	G*010108	—	CEPH G7	3102, CUI-62	U58029, GQ996568	J Martinez-Laso^b^
G*01:01:09	G*010109	—	—	JHUAA0527, JHUAA0529, JHUAA0530	AY645765	(333)
G*01:01:11	G*010111	—	—	HLAG005	EF565823	(337)
G*01:01:12	G*010112	—	—	JKKB-55, SSTO	EF218653, BX247949	(338), S Beck^b^
G*01:01:13	G*010113	—	—	JKKB-87	EF218654	(339)
G*01:01:14	G*010114	—	—	TXH12-2R3	EU220991	(340)
G*01:01:15	G*010115	—	—	BCCR-565, BCCR-1020, 7b031, 7b061	FJ460463	(341)
G*01:01:16	G*010116	—	—	BCCR-1753	FJ464334	(341)
G*01:01:17	G*010117	—	—	BCCR-773, BCCR-1502	FJ460464	(341)
G*01:01:18	G*010118	—	—	BCCR-991	FJ469162	(341)
G*01:01:19	G*010119	—	—	Rog338, Rog342, Rog366, Rog425	FJ464336	(341)
G*01:01:20	G*010120	—	—	BCI-04	GU070582	J Martinez-Laso^b^
G*01:02	G*0102	—	Ice 6.23-5.4H	ICE 6	S69897	
G*01:03	G*0103	—	G*III, GCO9	LWAGS, HT59, IHW01175, IHW01181, BCI-06, BCI-08	L20777, U65241, U65242, AY645772, GQ996561, GQ996562	(333)^b^, J Martinez-Laso^b^
G*01:04:01	G*010401	—	GJ3, GCO7, CEPH G2	KMR, CHI525, HT98, 1302, LKT3, BCI-19	D67006, D67007, D67008, L78072, U65237, U65238, U58025, AB088083, GQ996559	H Inoko^b^, J Martinez-Laso^b^
G*01:04:02	G*010402	—	CEPH G3	2701	U58094	
G*01:04:03	G*010403	—	CEPH G4	2701, CUI-62	U58026	J Martinez-Laso^b^
G*01:04:04	G*010404	—	—	IHW01182, IHW01184, IHW01173, BCI-18	AY645773, GQ996558	(333), J Martinez-Laso^b^
G*01:04:05	G*010405	—	—	Rog482, Rog500, Rog504	FJ464335	(341)
G*01:05N	G*0105N	Null	G*1.5	DCH027, LBF	L78073, AF523299	(333)^b^
G*01:06	G*0106	—	—	050900cA537, APD, BCI-09	AF312697, CR925767, GQ996564	S Beck^b^, J Martinez-Laso^b^
G*01:07	G*0107	—	—	JHUAA0527	AY645767	(333)
G*01:08	G*0108	—	—	D8	EF375550	(342)
G*01:09	G*0109	—	—	JKKB-28	EF218655	(343)
G*01:10	G*0110	—	—	ML1699	EU290672	M Luo
G*01:11	G*0111	—	—	ML1837	EU290673	M Luo
G*01:12	G*0112	—	—	Roger535	EU750733	(344)
G*01:13N	G*0113N	Null	—	7a095	EU750734	(344)
G*01:14	G*0114	—	—	BCCR498, 9b007, HLA4	EU750735	(344)
G*01:15	G*0115	—	—	BCCR1016	EU750736	(344)
G*01:16	G*0116	—	—	BCCR996	EU750737	(344)
G*01:17	G*0117	—	—	8203	GQ374478	M Luo

a Allele names given in bold type have been assigned since the 2004 Nomenclature report.

b This reference is to a confirmatory sequence.

c HLA specificity provided from the HLA dictionary (22–26).

**Table 5:** Designations of HLA-H, -J, -K, L, P, and -V alleles

HLA allelesa	Pre 2010 designation	HLA specificity	Previous equivalents	Individual or cell line from which the sequence was derived	Accession number	References or submitting author(s)
H*01:01:01:01	H*01010101	—	5.4-LCL.1, 168E5 10.1	LCL721, BOLETH, DBB	M32106, AF055066, CR388220	(345), (42), S Beck
H*01:01:01:02	H*01010102	—	JY8	JY	X12432	(346)
H*01:01:01:03	H*01010103	—	CosRS6	3.1.0	M31944	(347)
H*01:02	H*0102	—	—	QBL	AL845454	(105)
H*02:01:01:01	H*02010101	—	PAC779F20	COX, APD, RPC15	AL671561, CT009496, AB023057	(29), S Beck, (334)
H*02:01:01:02	H*02010102	—	HLA54-13.2	LCL721	M96336	(348)
H*02:02	H*0202	—	—	MANN, CGM1	BX927141, AC004194	S Beck, DE Geraghty
H*02:03	H*0203	—	—	SSTO	BX284699	S Beck
H*02:04	H*0204	—	—	PGF	AL645929	(29)
H*02:05	H*0205	—	5.4-LBF	LBF	M32105	(345)
H*02:06	H*0206	—	pHLA 12.4	JG	J00191	(349)
H*03:01	H*0301	—	5.4-BB	BB	M32104	(345)
						
J*01:01:01:01	J*01010101	—	—	CGM1, MOU	AF055066, AC005404, BX927229	(42), DE Geraghty, S Beck
J*01:01:01:02	J*01010102	—	—	COX, RPC15, APD, DBB	AL645935, BA000025, BA000026, CR759960, CR388205	(29), (334), S Beck
J*01:01:01:03	J*01010103	—	—	CD	M80469	(350)
J*01:01:01:04	J*01010104	—	—	PGF	AL669914, AL671277	(29)
J*01:01:01:05	J*01010105	—	—	MCF	CR759763	S Beck
J*01:01:01:06	J*01010106	—	—	SSTO	BX005091, BX120004, BX088647	S Beck
J*01:01:01:07	J*01010107	—	—	MOLT-4	M80468	(350)
J*01:01:01:08	J*01010108	—	—	QBL	AL845454	(105)
J*02:01	J*0201	—	—	LCL721	M80470, M96337	(350), (348)
						
K*01:01:01:01	K*01010101	—	—	PRC15, PGF	AB023056, AL671277	(334), (29)
K*01:01:01:02	K*01010102	—	—	COX, APD	AL645935, CR759913	(29), S Beck
K*01:01:01:03	K*01010103	—	—	QBL	AL845454	(105)
K*01:01:01:04	K*01010104	—	—	SSTO	BX005091	S Beck
K*01:02	K*0102	—	—	BOLETH, CGM1, DBB	AF055066, CR388220	(42), S Beck
K*01:03	K*0103	—	—	CGM1, MOU, SPLEN2	AC004203, CR392333, AK092921	DE Geraghty, S Beck, (351)
						
L*01:01:01:01	L*01010101	—	—	DBB, SSTO	CR388382, BX248419	S Beck
L*01:01:01:02	L*01010102			RPC15, QBL	AB014088, AL844220	(334), (105)
L*01:01:01:03	L*01010103			PGF	AL662782	(29)
L*01:01:02	L*010102			COX	AL662832	(29)
L*01:02	L*0102			CGM1, MOU	AC004191, BX927189	DE Geraghty, S Beck
						
P*01:01:01:01	P*01010101	—	—	APD, RPC15, COX	CR925767, AB023058, AL671561	S Beck, (334), (29)
P*01:01:01:02	P*01010102	—	—	QBL	AL844851	(105)
P*02:01:01:01	P*02010101	—	—	MANN, DBB, MLF, CGM1	BX927141, CR759769, CR788234, AC004172, AC004192	S Beck, DE Geraghty
P*02:01:01:02	P*02010102	—	—	PGF, SSTO, RPCI-3	AL645939, BX005428, AL022723	(29), S Beck
						
V*01:01:01:01	V*01010101	—	—	BOLETH, CGM1, MANN, DBB, MLF, PGF, SSTO, RPCI-3	AF055066, AC004192, BX927182, CR759769, CR788234, AL645939, BX005428, AL022723	(42), DE Geraghty, S Beck, (29)
V*01:01:01:02	V*01010102	—	—	RPCI5, APD, COX	BA000025, AB023058, CR925767, AL671561	(334), S Beck, (29)
V*01:01:01:03	V*01010103	—	—	QBL	AL844851	(105)

a Allele names given in bold type have been assigned since the 2004 Nomenclature report.

**Table 6:** Designations of HLA-DRA and -DRB alleles

HLA allele^a^	Pre 2010 designation	HLA-DR serological specificities	HLA-D Associated (T-cell defined) specificities	Previous equivalents	Individual or cell line from which the sequence was derived	Accession number	References or submitting author(s)
DRA*01:01	DRA*0101	DR1	—	DRα,PDR-α-2	JY, RAJI, F.G., QBL	J00194, J00196, J00203, AL935032	(105)^b^
DRA*01:02:01	DRA*010201	—	—	DR-H	JY	J00201, AF481359	
DRA*01:02:02	DRA*010202	—	—	—	HSF7	Z84814	
							
DRB1*01:01:01	DRB1*010101	DR1	Dw1	—	45.1, LG2, JSA, DRH, CHG, HOM-2	X03069, M11161, AF029288, AM493435	SGE Marsh^b^
DRB1*01:01:02	DRB1*010102	DR1	Dw1	—	9380965	AF479570	
DRB1*01:01:03	DRB1*010103	DR1	Dw1	—	OLLI	AY271987	
DRB1*01:01:04	DRB1*010104	DR1	—	—	OT11400	AM931296	S Tavoularis
DRB1*01:01:05	DRB1*010105	DR1	—	—	CTM-5099261	EU683685	A Balas
DRB1*01:01:06	DRB1*010106	DR1	—	—	36573, HN-49780-2, HN-85493-3, HN-31251-0, HN-84578-0	AM992171, FJ392254, FJ392260, FJ392231, FJ875598	J Enczmann, Histogenetics^b^
DRB1*01:01:07	DRB1*010107	DR1	—	—	47995, HN-10439-8, HN-13937-9, HN-93534-0, HN-61741-5, HN-14364-3, HN-76700-4, HN-81295-9, HN-96267-1, HN-99986-3, HN-46163-3, HN-14489-0, HN-64829-7, HN-70297-4	AM992172, FJ392240, FJ392245, FJ392246, FJ392234, FJ392237, FJ392239, FJ392241, FJ392242, FJ392243, FJ392249, FJ392252, FJ392256, FJ875601	J Enczmann, Histogenetics^b^
DRB1*01:01:08	DRB1*010108	DR1	—	—	51202	FM162178	J Enczmann
DRB1*01:01:09	DRB1*010109	DR1	—	—	28249	FM196527	J Enczmann
DRB1*01:01:10	DRB1*010110	DR1	—	—	2718193, HN-55044-1	FN395287, FJ392236	S Schuett, Histogenetics^b^
DRB1*01:01:11	DRB1*010111	DR1	—	—	HN-78998-0	FJ392230	Histogenetics
DRB1*01:01:12	DRB1*010112	DR1	—	—	HN-01410-0	FJ392238	Histogenetics
DRB1*01:01:13	DRB1*010113	DR1	—	—	HN-77198-3	FJ392244	Histogenetics
DRB1*01:01:14	DRB1*010114	DR1	—	—	HN-07537-5	FJ392247	Histogenetics
DRB1*01:01:15	DRB1*010115	DR1	—	—	HN-8610748	FJ538282	Histogenetics
DRB1*01:01:16	DRB1*010116	DR1	—	—	HN-27144-5, BY00456	FJ392251, GU066757	Histogenetics, CK Hurley
DRB1*01:01:17	DRB1*010117	DR1	—	—	HN-38082-8	FJ392259	Histogenetics
DRB1*01:02:01	DRB1*010201	DR1	Dw20	DR1-NASC	NASC, 1568, MUM, NA01018	AF029293, AY663400	(352)^b^
DRB1*01:02:02	DRB1*010202	DR1	Dw20	DRB1*01DMT	TO0973	Z50871	
DRB1*01:02:03	DRB1*010203	DR1	Dw20	—	376P01	AJ430382	
DRB1*01:02:04	DRB1*010204	DR1	Dw20	—	84308	AY730638	
DRB1*01:02:05	DRB1*010205	DR1	—	—	115664	FM162176	J Enczmann
DRB1*01:03	DRB1*0103	DR103	Dw’BON’	DR1-CETUS, DRB1*BON	RAI, BG, BON	M33600	
DRB1*01:04	DRB1*0104	DR1	—	DRB1*01New	L.R., LAUTH J	X70261, X99896	
DRB1*01:05	DRB1*0105	—	—	DRB1*0101V1	JC10218	AB015184	
DRB1*01:06	DRB1*0106	—	—	—	MGM14106	AJ089723	
DRB1*01:07	DRB1*0107	DR1	—	DRB1*New	ZAE, IOL Gae, IOL Ire	AJ276206, AJ303118	
DRB1*01:08	DRB1*0108	—	—	DR1-BCN	HSP934010	AY034875	
DRB1*01:09	DRB1*0109	DR1	—	—	G044601003890Z	AJ430192	
DRB1*01:10	DRB1*0110	—	—	DRB1*01CBRL	CBRL9-58-288	AY152671	
DRB1*01:11	DRB1*0111	—	—	—	03-2215	AJ581747	
DRB1*01:12	DRB1*0112	—	—	—	MHH0401996	AJ870972	
DRB1*01:13	DRB1*0113	DR1	—	—	CTM-1096976	DQ002917	(353)
DRB1*01:14	DRB1*0114	—	—	—	Xian8576	DQ412558	(354)
DRB1*01:15	DRB1*0115	—	—	—	VTIA89969	DQ133167	BD Tait
DRB1*01:16	DRB1*0116	—	—	—	2006-038-07126, NT00740	AM286281, EU146149	B Norvell, CK Hurley^b^
DRB1*01:17	DRB1*0117	—	—	—	BY00214	EU029796	(36)
DRB1*01:18	DRB1*0118	—	—	—	BY00212	EU029794	(36)
DRB1*01:19	DRB1*0119	—	—	—	BY00209, BY00208	EU029791, EU029790	(36)
DRB1*01:20	DRB1*0120	—	—	—	JMDP36K017, 191387, HN-81195-8	AM436794, FM212659, FJ766010	K Tadokoro, K Witter^b^, Histogenetics^b^
DRB1*01:21	DRB1*0121	—	—	—	BY00426	FJ688167	CK Hurley
DRB1*01:22	DRB1*0122	—	—	—	0870	GQ148551	ZM Kashi
DRB1*01:23	DRB1*0123	—	—	—	BY00499	GQ410118	CK Hurley
DRB1*01:24	DRB1*0124	—	—	—	HN-02099-8	FJ392232	Histogenetics
DRB1*01:25	DRB1*0125	—	—	—	HN-04555-7	FJ392233	Histogenetics
DRB1*01:26	DRB1*0126	—	—	—	HN-7826, HN-5410	FJ392235, FJ549418	Histogenetics
DRB1*01:27	DRB1*0127	—	—	—	HN-65603-4	FJ392248	Histogenetics
DRB1*01:28	DRB1*0128	—	—	—	HN-27911-7	FJ392250	Histogenetics
DRB1*01:29	DRB1*0129	—	—	—	HN-50700-6, HN-83987-5	FJ392255, FJ898494	Histogenetics
DRB1*01:30	DRB1*0130	—	—	—	HN-05817-4	FJ392257	Histogenetics
DRB1*01:31	DRB1*0131	—	—	—	HN-07825-7	FJ392258	Histogenetics
DRB1*03:01:01:01	DRB1*03010101	DR17(3)	Dw3	dJ93N13	RAJI, AVL, WT49, DM24, DM28, DM29, CMCC, HSF7, APR, ALL, MVJ, MUR, U-STH, QBL, COX, NA010118, NA10923	M17379, X04054, Z84489, AF029265, AF152843, AL929581, AL662842, AY663399, AY663407	(29)^b^, (352)^b^
DRB1*03:01:01:02	DRB1*03010102	DR17(3)	—	—	QBL	AL929581	(105)
DRB1*03:01:02	DRB1*030102	DR17(3)	Dw3	DRB1*IMR	21, M.R., CTM-2095057	M91807, L07767, AM961064	(353)^b^
DRB1*03:01:03	DRB1*030103	DR17(3)	—	—	D2259	AM410992	O Avinens
DRB1*03:01:04	DRB1*030104	DR17(3)	—	—	VTIS65942	DQ133168	BD Tait
DRB1*03:01:05	DRB1*030105	DR17(3)	—	—	NT00735	EF591034	(355)
DRB1*03:01:06	DRB1*030106	DR17(3)	—	—	MHHN-593833	AM777659	R Blasczyk
DRB1*03:01:07	DRB1*030107	DR17(3)	—	—	HN-36534-8	FJ205634	Histogenetics
DRB1*03:01:08	DRB1*030108	DR17(3)	—	—	09d05193	FN563146	JDH Anholts
DRB1*03:02:01	DRB1*030201	DR18(3)	Dw’RSH’	—	2041, 1563, 24A1	M27689, AF029266	
DRB1*03:02:02	DRB1*030202	DR18(3)	Dw’RSH’	—	GN055, GMONT	U29342, U82403	
DRB1*03:03	DRB1*0303	DR18(3)	—	—	RBL B25, GN00265	M81743, AY429722	
DRB1*03:04	DRB1*0304	DR17(3)	—	03MIT	MIT3758, 35919, TER336	X75441, AJ409216, AM109967	R Blasczyk^b^
DRB1*03:05:01	DRB1*030501	DR3	—	DR3New	U-HFI, TTO5607	L29807, U26557	
DRB1*03:05:02	DRB1*030502	DR3	—	—	LAHRE	AF335318	
DRB1*03:05:03	DRB1*030503	DR3	—	—	FP177512, 42856	FN252190, FN313509	F Poli, T Gervais
DRB1*03:06	DRB1*0306	DR3	—	—	JV1094, CTM-2096757	X90644, AY961067	(353)^b^
DRB1*03:07	DRB1*0307	DR3^c^	—	—	GN073, NT00982	U37433, EU812544	CK Hurley^b^
DRB1*03:08	DRB1*0308	—	—	—	GN090	U47028	
DRB1*03:09	DRB1*0309	—	—	—	D438	X93315	
DRB1*03:10	DRB1*0310	DR17(3)^c^	—	—	PMR	U65585	
DRB1*03:11:01	DRB1*031101	DR17(3)^c^	—	—	UWE02	U79028	
DRB1*03:11:02	DRB1*031102	DR17(3)^c^	—	—	BY00543	GU066754	CK Hurley
DRB1*03:12	DRB1*0312	DR3	—	DRB1*03AGC	WVN	Y17274	
DRB1*03:13:01	DRB1*031301	—	—	—	DELAT	AJ012424	
DRB1*03:13:02	DRB1*031302	—	—	—	N-634755	FM998808	R Blasczyk
DRB1*03:14	DRB1*0314	DR3	—	DR’KW’	KW	Y17863	
DRB1*03:15	DRB1*0315	DR3	—	DRB1*0301A	DKMS 585607, TER317	AJ237899, AM109968	R Blasczyk^b^
DRB1*03:16	DRB1*0316	—	—	—	09343336	AF169240	
DRB1*03:17	DRB1*0317	—	—	DRB1*13KM	SMS202-147-KerHut	AJ238154	
DRB1*03:18	DRB1*0318	—	—	DRB1*03XX	RSA036575, MSA058812	AJ279010	
DRB1*03:19	DRB1*0319	—	—	—	GCASS, 133983	AF343002, AM409244	H Dunckley^b^
DRB1*03:20	DRB1*0320	—	—	DRB1*03011var	NT0022	AF352294	
DRB1*03:21	DRB1*0321	—	—	—	Patient#17839	AJ297266	
DRB1*03:22	DRB1*0322	—	—	—	MAWE0816AN, LB64975, LB62448	AJ420288, AJ506788	
DRB1*03:23	DRB1*0323	DR3	—	—	DNA6060	AY116505	
DRB1*03:24	DRB1*0324	—	—	—	ULM13352	AY179833	
DRB1*03:25	DRB1*0325	—	—	—	DKM544293	AY188946	
DRB1*03:26	DRB1*0326	—	—	—	BELF	AY299386	
DRB1*03:27	DRB1*0327	—	—	—	5994, BY00119	AY536008, DQ782330	CK Hurley^b^
DRB1*03:28	DRB1*0328	—	—	—	NT00510	AY607029	
DRB1*03:29	DRB1*0329	—	—	—	NT00626, BY00247	DQ334726, EU095402	CK Hurley
DRB1*03:30	DRB1*0330	—	—	DRB1*03MSC0506	MHHZ-00020533	AM269468	R Blasczyk
DRB1*03:31	DRB1*0331	—	—	—	TZDR03	DQ666333	(356)
DRB1*03:32	DRB1*0332	—	—	—	VTIS103774	DQ133169	BD Tait
DRB1*03:33	DRB1*0333	—	—	—	VTIS200629, VTIS200663, VTIS200630, VTIS200631	EF471909	BD Tait
DRB1*03:34	DRB1*0334	—	—	—	OT14365	AM493690	(357)
DRB1*03:35	DRB1*0335	—	—	—	34162	EF495129	J Li
DRB1*03:36	DRB1*0336	DR3	—	—	LUMC-DRB15	AM746479	(64)
DRB1*03:37	DRB1*0337	—	—	—	BY00232, BY00254, 289746	EU071682, EU095396, FN550804	(36), EKL Yang^b^
DRB1*03:38	DRB1*0338	—	—	—	BY00237	EU084397	(36)
DRB1*03:39	DRB1*0339	—	—	—	BY00253	EU095397	(36)
DRB1*03:40	DRB1*0340	DR3	—	—	60648330	EU660882	R Kelsch
DRB1*03:41	DRB1*0341	—	—	—	BY00423	FJ688164	CK Hurley
DRB1*03:42	DRB1*0342	—	—	—	P-643995, 1044283	FM998809, FJ877151	R Blasczyk, E Palou
DRB1*03:43	DRB1*0343	DR17(3)	—	—	49784, CS00007	FN392687, FJ986467	J Johnson, K Cao^b^
DRB1*03:44	DRB1*0344	—	—	—	HN-32510-1, 5528727076	FJ197336, GQ862779	Histogenetics, D Smillie^b^
DRB1*03:45	DRB1*0345	—	—	—	HN-78699-5	FJ205617	Histogenetics
DRB1*03:46	DRB1*0346	—	—	—	HN-44447-1	FJ205618	Histogenetics
DRB1*03:47	DRB1*0347	—	—	—	HN-17865-8, HN-21128-5	FJ205638, FJ205639	Histogenetics
DRB1*03:48	DRB1*0348	—	—	—	HN-01618-2	FJ217730	Histogenetics
DRB1*03:49	DRB1*0349	—	—	—	JEFE658240AN	FN551180	SGE Marsh
DRB1*03:50	DRB1*0350	—	—	—	93051421	EU445580	A Vigh
DRB1*03:51	DRB1*0351	—	—	—	BY00538	GU066750	CK Hurley
DRB1*03:52	DRB1*0352	—	—	—	HN-80114-2	FJ858908	Histogenetics
DRB1*04:01:01	DRB1*040101	DR4	Dw4	—	WT51, PRIESS, MJ4, BOLETH, LTC	K02776, M17381, M20548-50, AF029267	
DRB1*04:01:02	DRB1*040102	DR4	Dw4	—	MC	X96851	
DRB1*04:01:03	DRB1*040103	DR4	—	—	BY00238	EU084396	(36)
DRB1*04:01:04	DRB1*040104	DR4	—	—	HN-88028-8	FJ438928	Histogenetics
DRB1*04:01:05	DRB1*040105	DR4	—	—	HN-20315-2	FJ438930	Histogenetics
DRB1*04:01:06	DRB1*040106	DR4	—	—	HN-57737-8	FJ549416	Histogenetics
DRB1*04:02	DRB1*0402	DR4	Dw10	—	FS, DM24, MMCC, LPB, YAR	M15068, AF029268, AJ245881, AJ297586	
DRB1*04:03:01	DRB1*040301	DR4	Dw13	DR4 Dw13A, 13.1	SSTO, TAS, NBP	AF029269, BX296568	(358)^b^
DRB1*04:03:02	DRB1*040302	DR4	Dw13	DRB1*SD	BM1 116040, 32891, TER337	AF112876, AJ295845, AM109969	R Blasczyk^b^
DRB1*04:03:03	DRB1*040303	DR4	—	—	173171	AM409261	H Dunckley
DRB1*04:03:04	DRB1*040304	DR4	—	—	NT00760	EU275154	(355)
DRB1*04:03:05	DRB1*040305	DR4	—	—	HN-09532-5	FJ438927	Histogenetics
DRB1*04:04:01	DRB1*040401	DR4	Dw14	DR4 Dw14A, 14.1	BIN40, LS40, DM29, RGR	X02902, M15069, M15073, M15074, AF029270	
DRB1*04:04:02	DRB1*040402	DR4	—	—	BY00501	GQ410120	CK Hurley
DRB1*04:04:03	DRB1*040403	DR4	—	—	HN-37506-5, HN-78268-8	FJ438924, FJ594733	Histogenetics
DRB1*04:04:04	DRB1*040404	DR4	—	—	HN-00728-6	FJ438929	Histogenetics
DRB1*04:05:01	DRB1*040501	DR4	Dw15	—	KT3, JML, AHC, CRP, DOS, SW	M15070, L13875, AF029271, AY626552	
DRB1*04:05:02	DRB1*040502	DR4	Dw15	DRB1*KOM	KOM	D50889, D49952	
DRB1*04:05:03	DRB1*040503	DR4	Dw15	DRB1*JVASA	JVASA, 140383	AF450094, AM409248	H Dunckley^b^
DRB1*04:05:04	DRB1*040504	DR4	Dw15	—	GN00419, HAN-1025	AY094139, EU678648	G Wohlwend^b^
DRB1*04:05:05	DRB1*040505	DR4	—	—	BY00236, R030242430, BY00251	EU084398, DQ836315, EU095399	(36), L Smith
DRB1*04:05:06	DRB1*040506	DR4	—	—	JMDP01K028	AB436777	K Tadokoro
DRB1*04:05:07	DRB1*040507	DR4	—	—	JMDP36K070	AB512679	K Tadokoro
DRB1*04:05:08	DRB1*040508	DR4	—	—	BY00497	GQ410116	CK Hurley
DRB1*04:05:09	DRB1*040508	DR4	—	—	HN-8629540	FJ766004	Histogenetics
DRB1*04:06:01	DRB1*040601	DR4	Dw’KT2’	—	KT2, 43A3	AF029272	
DRB1*04:06:02	DRB1*040602	DR4	—	—	CTM-9096471	AY961074	(353)
DRB1*04:06:03	DRB1*040603	DR4	—	—	BY00500	GQ410119	CK Hurley
DRB1*04:07:01	DRB1*040701	DR4	Dw13	DR4 Dw13B, 13.2	JHF, R88, JRR, CTM-0096476	M37771, AF029273, AY961075	(353)^b^
DRB1*04:07:02	DRB1*040702	DR4	Dw13	DRB1*0407var	NT0019	AF352291	
DRB1*04:07:03	DRB1*040703	DR4	Dw13	—	CH112	AY158889	
DRB1*04:07:04	DRB1*040704	DR4	Dw13	—	HN-18682-5, HN-763322	FJ438923, FJ489881	Histogenetics
DRB1*04:08:01	DRB1*040801	DR4	Dw14	DR4-CETUS, Dw14B, 14.2	M36, RA1, SUDNA0254, RGR, CTM1096458	M37770, L78169, AF029274, AY961068	(353)^b^
DRB1*04:08:02	DRB1*040802	DR4	—	—	JMDP36K049	AB512675	K Tadokoro
DRB1*04:09	DRB1*0409	DR4	—	—	R80, LC284, GN00057	M64794, AJ536121, AY277386, AY504881	
DRB1*04:10	DRB1*0410	DR4	—	DR4.CB, STF22421	CB, ABCC60, EGR, T.D.DNA#22421, CTM-6097095	M81670, M80192, AF029275, AY174183, AY961062	(353)^b^
DRB1*04:11	DRB1*0411	DR4	—	DR4.EC	EC, HV846, HAA, JMJ, C211	M81700, M55615, L42143, L79973, AJ556173	
DRB1*04:12	DRB1*0412	—	—	AB2	ABO1078, 132935	M77672, AM409255	H Dunckley^b^
DRB1*04:13	DRB1*0413	DR4	—	DRB1*LEV	LEV, BY00474	M94460, GQ373163	CK Hurley^b^
DRB1*04:14	DRB1*0414	DR4	—	DR4 Dw10.2	VK, NT00980	X65031, EU812546	CK Hurley^b^
DRB1*04:15	DRB1*0415	DR4	—	—	NIC, HOU, TER308	X68272, AM109970	R Blasczyk^b^
DRB1*04:16	DRB1*0416	DR4	—	DR4-BELF	BEL5GB, Terasaki B-Cell 260	X70788, AY296122, AM109971	R Blasczyk^b^
DRB1*04:17:01	DRB1*041701	DR4	—	DRB1*04SAM	TOB-0070, BY00255	L14481, EU095395	CK Hurley^b^
DRB1*04:17:02	DRB1*041702	DR4	—	—	BY00541	GU066752	CK Hurley
DRB1*04:18	DRB1*0418	—	—	DRB1*04.N	AI7, AI8, 74DR, GN0222	X71610, U38974, AY504814	
DRB1*04:19	DRB1*0419	DR4	—	DR4FK	FK, G.A.DNA#7881	L21985, AY179370	
DRB1*04:20	DRB1*0420	DR4	—	DRB1*04MC	AD-7863, BM29/92	L27217	
DRB1*04:21	DRB1*0421	DR4	—	DR4New	SMH	X80288	
DRB1*04:22	DRB1*0422	DR4	—	DR4New	D18002	U17014	
DRB1*04:23	DRB1*0423	DR4	—	—	MAG	Z68503	
DRB1*04:24	DRB1*0424	DR4	—	DRB1*Mi	Mi	Z71541	
DRB1*04:25	DRB1*0425	DR4	—	DRB1*04ISA	RI, HB	Y09211	
DRB1*04:26	DRB1*0426	DR4	—	DRB1*04CMT	T010148, HAN-1024	AJ001252, EU678647	G Wohlwend^b^
DRB1*04:27	DRB1*0427	—	—	—	NOR03	AF030439	
DRB1*04:28	DRB1*0428	DR4	—	DRB1*0405V1	JC4772	AB007635	
DRB1*04:29	DRB1*0429	DR4	—	DRB1*0405V2	JC7616	AB007636	
DRB1*04:30	DRB1*0430	—	—	DRB1*0405V3	JC9227	AB015185	
DRB1*04:31	DRB1*0431	DR4	—	DRB1*04New	GE47192,	AJ009755, AM109972	R Blasczyk^b^
DRB1*04:32	DRB1*0432	DR4^c^	—	DRB1*04-A	NIE	Y17273	
DRB1*04:33	DRB1*0433	—	—	DRB1*04_7468	WBD7468, 177927	AF023153, AM409254	H Dunckley^b^
DRB1*04:34	DRB1*0434	—	—	DRB1*04new	CB1653, DWEVE488	AJ133492, AM109973	R Blasczyk
DRB1*04:35	DRB1*0435	—	—	DRB1*04New	NT0009	AF242355	
DRB1*04:36	DRB1*0436	—	—	—	BN61, 175586	AF240637, AM409253	H Dunckley^b^
DRB1*04:37	DRB1*0437	—	—	DRB1*04nv	MDPH0002764	AY007565	
DRB1*04:38	DRB1*0438	—	—	—	SLTA, VTIS72428	AF235034, AF489510	
DRB1*04:39	DRB1*0439	—	—	DRB1*04031var	NT0024, 166438	AF352296, AM409252	H Dunckley^b^
DRB1*04:40	DRB1*0440	—	—	DRB1*0404var	NT0020, 101330, HAN-1023	AF352292, AM409251, EU678646	H Dunckley^b^, G Wohlwend^b^
DRB1*04:41	DRB1*0441	—	—	DRB1*04031var	NT0021, NT00791	AF352293, EU643613	CK Hurley^b^
DRB1*04:42	DRB1*0442	DR4	—	—	JH71321	AF304866	
DRB1*04:43	DRB1*0443	—	—	—	OORCH18	AY042678, AF349316	
DRB1*04:44	DRB1*0444	—	—	—	satt44124	AF497643	
DRB1*04:45	DRB1*0445	—	—	—	HY-LHB, HY-LBG	AY191224	
DRB1*04:46	DRB1*0446	—	—	—	TBC-22381	AB106128	
DRB1*04:47	DRB1*0447	—	—	—	119244	AJ574788	
DRB1*04:48	DRB1*0448	—	—	—	TBC27636	AB087874	
DRB1*04:49	DRB1*0449	—	—	—	SR289	AY374099	
DRB1*04:50	DRB1*0450	—	—	—	0298-8458-2	AY265414	
DRB1*04:51	DRB1*0451	—	—	—	CH04021606	AY776333	
DRB1*04:52	DRB1*0452	—	—	—	TBC50414	AB196528	
DRB1*04:53	DRB1*0453	—	—	—	13267	AY947537	(359)
DRB1*04:54	DRB1*0454	—	—	—	NT00603, BY00239	DQ112548, EU084395	CK Hurley
DRB1*04:55	DRB1*0455	—	—	—	NT00581	DQ112549	CK Hurley
DRB1*04:56	DRB1*0456	—	—	—	FASO	DQ130065	L Mele
DRB1*04:57	DRB1*0457	—	—	—	TBC58426	AB248283	M Satake
DRB1*04:58	DRB1*0458	—	—	—	BY00101	DQ473290	(57)
DRB1*04:59	DRB1*0459	—	—	—	NT00660	DQ525631	(355)
DRB1*04:60	DRB1*0460	—	—	—	157748	AM261439	(360)
DRB1*04:61	DRB1*0461	—	—	—	28471	EF059809	(361)
DRB1*04:62	DRB1*0462	—	—	—	BY00139	EF078990	(163)
DRB1*04:63	DRB1*0463	—	—	—	ADN6428, ADN6429	AM412363	F Hau
DRB1*04:64	DRB1*0464	DR4	—	—	C136004	EF158822	(362)
DRB1*04:65	DRB1*0465	—	—	—	KHM2/171877	AM765992	(363)
DRB1*04:66	DRB1*0466	—	—	—	JAMC7746AN	AM773422	AM Little
DRB1*04:67	DRB1*0467	—	—	—	BJ042	EF687782	L Wang
DRB1*04:68	DRB1*0468	—	—	—	BY00241, BY00242	EU095409, EU095408	(36)
DRB1*04:69	DRB1*0469	—	—	—	BY00245	EU095405	(36)
DRB1*04:70	DRB1*0470	—	—	—	BY00248	EU095401	(36)
DRB1*04:71	DRB1*0471	—	—	—	BY00250	EU095400	(36)
DRB1*04:72:01	DRB1*047201	—	—	—	ESGR11535AN	AM899948	AM Little
DRB1*04:72:02	DRB1*047202	—	—	—	BY0544	GU066755	CK Hurley
DRB1*04:73	DRB1*0473	—	—	—	LUMC-DR35	AM904555	(64)
DRB1*04:74	DRB1*0474	—	—	—	SI772, FFM5175	AM947655, FM886832	(364), V Brixner^b^
DRB1*04:75	DRB1*0475	—	—	—	HAN-1022	AY766103	G Wohlwend
DRB1*04:76	DRB1*0476	—	—	—	HAN-1026	EU678649	G Wohlwend
DRB1*04:77	DRB1*0477	—	—	—	JMDP01K027	AB436776	K Tadokoro
DRB1*04:78	DRB1*0478	—	—	DRB1*04	SZGSQ2	FJ547092	(365)
DRB1*04:79	DRB1*0479	—	—	—	NT01105, BY00545	GQ373170, GU066756	CK Hurley
DRB1*04:80	DRB1*0480	—	—	—	JMDP01K055	AB511893	K Tadokoro
DRB1*04:81N	DRB1*0481N	Null	—	—	JMDP36K069	AB512504	K Tadokoro
DRB1*04:82	DRB1*0482	—	—	—	JMDP36K051	AB512677	K Tadokoro
DRB1*04:83	DRB1*0483	—	—	—	BY00498	GQ410117	CK Hurley
DRB1*04:84	DRB1*0484	—	—	—	BY00502	GQ410121	CK Hurley
DRB1*04:85	DRB1*0485	—	—	—	KBCA7117	AB471013	Y Kuroda
DRB1*04:86	DRB1*0486	—	—	—	HN-52485-0	FJ438911	Histogenetics
DRB1*04:87	DRB1*0487	—	—	—	HN-47294-4	FJ438913	Histogenetics
DRB1*04:88	DRB1*0488	—	—	—	HN-01694-8, HN-04356-1	FJ438916, FJ438917	Histogenetics
DRB1*04:89	DRB1*0489	—	—	—	HN-6790922	FJ858914	Histogenetics
DRB1*07:01:01:01	DRB1*07010101	DR7	Dw17, Dw’DB1’	—	BURKHARDT, MANN, LBF	M16941, M17384, U09201, CR753835	S Beck^b^
DRB1*07:01:01:02	DRB1*07010102	DR7	—	—	DBB	CR753309	S Beck
DRB1*07:01:02	DRB1*070102	DR7	—	DRB1*07New	CBM500	AJ243327	
DRB1*07:01:03	DRB1*070103	DR7	—	—	CTM0000474	EU835481	A Balas
DRB1*07:03	DRB1*0703	DR7	—	DRB1*07RMT	ED01436	Y13785	
DRB1*07:04	DRB1*0704	DR7	—	DRB1*07ROS	12827878	Y16224, AM109974	R Blasczyk^b^
DRB1*07:05	DRB1*0705	—	—	—	NT0012, NT01109	AF327742, GQ373167	CK Hurley^b^
DRB1*07:06	DRB1*0706	—	—	—	13765	AJ311892	
DRB1*07:07	DRB1*0707	—	—	—	AD65697, 41618	AJ441130, FN556019	T Gervais^b^
DRB1*07:08	DRB1*0708	—	—	—	K80504	AJ566209	
DRB1*07:09	DRB1*0709	DR7	—	—	CTM	AY770514	
DRB1*07:10N	DRB1*0710N	Null	—	DRB1*ERN0405N	DEHAN210681	AJ968418	(366)
DRB1*07:11	DRB1*0711	—	—	—	2005-228-01385, BY00192	AM233523, EU029789	B Norvell, CK Hurley^b^
DRB1*07:12	DRB1*0712	—	—	—	HABH6660AN, ABRO6838AN	AM411359	AM Little
DRB1*07:13	DRB1*0713	—	—	—	G046706009456V	EF362805	C Cormack
DRB1*07:14	DRB1*0714	—	—	—	25313-06	EF550999	F Schmitt
DRB1*07:15	DRB1*0715	—	—	—	SCU-1533	EU131777	(367)
DRB1*07:16	DRB1*0716	—	—	—	NT01011	FJ407051	CK Hurley
DRB1*07:17	DRB1*0717	—	—	07MVE0808	AKB-642962	FM998810	(368)
DRB1*08:01:01	DRB1*080101	DR8	Dw8.1	DRB1*0801	MADURA, SUDNA0140, U-STH, BM9, MTP1 134873, MULRe, 1823-T, BTB	M17386, L78166, AF144105, AF121971, AJ249626, AF278701, AY028514, AY028515, AY028516, AY028517, AY028518, AY028519	
DRB1*08:01:02	DRB1*080102	DR8	—	—	GN00415	AF491843	
DRB1*08:01:03	DRB1*080103	DR8	—	—	CTM-3095762	DQ090958	(353)
DRB1*08:01:04	DRB1*080104	DR8	—	—	BY00463	FJ976692	CK Hurley
DRB1*08:01:05	DRB1*080105	DR8	—	—	JMDP36K052	AB512678	K Tadokoro
DRB1*08:02:01	DRB1*080201	DR8	Dw8.2	DRw8-SPL	SPL, 24A2	AF029277	
DRB1*08:02:02	DRB1*080202	DR8	Dw8.2	DRw8b	OLL, C-78	AF029278	
DRB1*08:02:03	DRB1*080203	DR8	—	—	NT0014	AF327743	
DRB1*08:03:02	DRB1*080302	DR8	Dw8.3	DRw8-TAB	KT, FO, POPE, TAB089	M27511, AJ001094	
DRB1*08:04:01	DRB1*080401	DR8	—	RB1066-1, DR8-V86	1066, 1127, PM, MTR, TER323	M84446, M34315, AF029279, AM109975	R Blasczyk^b^
DRB1*08:04:02	DRB1*080402	DR8	—	—	CAY3, CAY5, CAY92, CAY96	L10402	
DRB1*08:04:03	DRB1*080403	DR8	—	—	UWEH03	U88135	
DRB1*08:04:04	DRB1*080404	DR8	—	—	NT0016	AF330103	
DRB1*08:05	DRB1*0805	DR8	—	DR8-A74	MS, NT01118	M84357, GQ892575	CK Hurley^b^
DRB1*08:06	DRB1*0806	DR8	—	DR8.6	RBL B24, RBL B124, SET, BOU, ALG, C.R., SUDNA0095, CTM-3096152	M87543, M86590, Z32685, L78165, AY961076	(353)^b^
DRB1*08:07	DRB1*0807	DR8	—	DR8BZ	AG, RG, L2, L4, TIC03, TIC04, TIC06, M.D.DNA#15923, TER323	L22341, L28096, AY179369, AM109976	R Blasczyk^b^
DRB1*08:08	DRB1*0808	—	—	08New	ETH3754, NT00979	X75443, EU812543	CK Hurley^b^
DRB1*08:09	DRB1*0809	DR8	—	DR8.7, DRB1*8.2V	BRI-10, JB44585	L23987, D45046, AB046526	
DRB1*08:10	DRB1*0810	DR8	—	LP10-1	K.R., R.R., TH10559	L19054, X82553	
DRB1*08:11	DRB1*0811	DR8	—	DR8TL, DR8New	ARA016, ARAC25, JR, ARA016	L29082, L32810, AM109977	R Blasczyk^b^
DRB1*08:12	DRB1*0812	DR8	—	DRB#52	4390, DRB#52	X88854, U36836	
DRB1*08:13	DRB1*0813	—	—	DRB#47	DRB#47, 29168, GN00422, CTM-2096080	U36571, AJ495001, AY122288, AY961077	(353)^b^
DRB1*08:14	DRB1*0814	DR8	—	DR8WE	WE, KE	U24179, AM109978	R Blasczyk^b^
DRB1*08:15	DRB1*0815	—	—	DRB1*08Taree	TDS-023, 161024	U63802, AM409250	H Dunckley^b^
DRB1*08:16	DRB1*0816	DR8	—	DRB1*08JST	ML0273, 24131	X99840, AJ309930	
DRB1*08:17	DRB1*0817	DR8	—	DRB1*08LRT	RV0253	Y09665	
DRB1*08:18	DRB1*0818	—	—	HLAAL1, HLA-DR8.5va	DKM379804, dJAE-0173, DU32971, CTM-1097277	U96926, Z99006, AJ223124, AY961078	(353)^b^
DRB1*08:19	DRB1*0819	—	—	DRB1*08YF, DRB1*08BL	VBD21599B, RP-BL046	AF016225, AF028011	
DRB1*08:20	DRB1*0820	—	—	DRB182624	82624	AJ000927	
DRB1*08:21	DRB1*0821	—	—	—	ROD01	AF049875	
DRB1*08:22	DRB1*0822	—	—	DRB1*08New	R9846, R9028	AJ276711	
DRB1*08:23	DRB1*0823	—	—	DRB1*08032V1	JCB13444	AB049829	
DRB1*08:24	DRB1*0824	—	—	DRB1*08022var	GN00391	AF363728	
DRB1*08:25	DRB1*0825	—	—	DR08GRS	CBRL 9-07-115	AY259125	
DRB1*08:26	DRB1*0826	—	—	—	0318965	AJ579780	
DRB1*08:27	DRB1*0827	—	—	—	W15820310531200E	AY428811	
DRB1*08:28	DRB1*0828	—	—	—	MMMBR	AY504815	
DRB1*08:29	DRB1*0829	—	—	—	TBC25160	AB176444	
DRB1*08:30:01	DRB1*083001	—	—	—	YZ	AJ850053	
DRB1*08:30:02	DRB1*083002	—	—	—	JMDP01K057	AB511950	K Tadokoro
DRB1*08:31	DRB1*0831	DR11(5)	—	—	CAPTN	AM000026	S Day
DRB1*08:32	DRB1*0832	—	—	—	EN120	DQ014541	(369)
DRB1*08:33	DRB1*0833	—	—	—	BJ045	EU079376	W Li
DRB1*08:34	DRB1*0834	—	—	—	BY00240	EU084394	(36)
DRB1*08:35	DRB1*0835	—	—	—	JMDP01K041	AB436800	K Tadokoro
DRB1*08:36	DRB1*0836	—	—	—	BJ56	FJ167389	L Wang
DRB1*08:37	DRB1*0837	—	—	—	JMDP01K056	AB511894	K Tadokoro
DRB1*08:38	DRB1*0838	—	—	—	JMDP36K050	AB512676	K Tadokoro
DRB1*08:39	DRB1*0839	—	—	—	42595	FN433880	T Gervais
DRB1*09:01:02	DRB1*090102	—	—	—	DKB, 09012, PMR, ISK, A6	M17387, U66826, D89917, DQ140279	KB Kwack^b^
DRB1*09:01:03	DRB1*090103	DR9	Dw23	—	D073	AY724685	
DRB1*09:01:04	DRB1*090104	DR9	—	—	JMDP36K018	AB436795	K Tadokoro
DRB1*09:01:05	DRB1*090105	DR9	—	—	BY00454	FJ842965	CK Hurley
DRB1*09:01:06	DRB1*090106	DR9	—	—	NT01106	GQ373169	CK Hurley
DRB1*09:02:01	DRB1*090201	—	—	—	J69	AY043181	
DRB1*09:02:02	DRB1*090202	—	—	—	2200308921182	FJ442950	(370)
DRB1*09:03	DRB1*0903	—	—	—	DE196	AY465114	
DRB1*09:04	DRB1*0904	—	—	—	78000829, CH04042702	AY772553	
DRB1*09:05	DRB1*0905	DR9	—	—	OPH11907	AB207269	Y Hisayama
DRB1*09:06	DRB1*0906	—	—	—	NT00680, NT00728, NT00741	DQ987876, EF536015, EU146148	(355)
DRB1*09:07	DRB1*0907	—	—	—	AS2054	AM947068	X Liu
DRB1*09:08	DRB1*0908	—	—	—	FJ167397	FJ167397	(371)
DRB1*09:09	DRB1*0909	—	—	—	BY00548	GU066759	CK Hurley
DRB1*10:01:01	DRB1*100101	DR10	—	—	RAJI, NASC	M20138	
DRB1*10:01:02	DRB1*100102	DR10	—	DRB1*10New	AW10-LCL	AF225565	
DRB1*10:01:03	DRB1*100103	DR10	—	—	18794	FM162177	J Enczmann
DRB1*10:02	DRB1*1002	—	—	—	BY00228	EU071686	(36)
DRB1*10:03	DRB1*1003	—	—	—	55328	AM992173	J Enczmann
DRB1*11:01:01	DRB1*110101	DR11(5)	Dw5	DRw11.1	SWEIG, 182590, NA00576	M11867, AM409247, AY663412	H Dunckley^b^, (352)^b^
DRB1*11:01:02	DRB1*110102	DR11(5)	Dw5	—	1180, 1249, 159548, NA01960	M34316, AM409246, AY663397	H Dunckley^b^, (352)^b^
DRB1*11:01:03	DRB1*110103	DR11(5)	Dw5	DR11.MD, DRB1*11DCT	DR11MDA, DR11MDB, BV3402	X86803, Y07590	
DRB1*11:01:04	DRB1*110104	DR11(5)	—	—	NT0015	AF329281	
DRB1*11:01:05	DRB1*110105	DR11(5)	—	—	TBC-33607, MHH-0107122	AB106129, AJ878425	P Horn^b^
DRB1*11:01:06	DRB1*110106	DR11(5)	—	—	MHHN-00009083	AJ871009	R Blasczyk
DRB1*11:01:07	DRB1*110107	DR11(5)	—	—	CGE691, 6460211	AM689932, AM712089	(261), A Wölpl^b^
DRB1*11:01:08	DRB1*110108	DR11(5)	—	—	194854	FN390946	K Witter
DRB1*11:01:09	DRB1*110109	DR11(5)	—	—	HN-704680-4	FJ205627	Histogenetics
DRB1*11:01:10	DRB1*110110	DR11(5)	—	—	HN-63310-7, HN-87124-6, HN-37349-5	FJ205637, FJ5205640, FJ538280	Histogenetics
DRB1*11:01:11	DRB1*110111	DR11(5)	—	—	BY00492, HN-3808, HN-0258	GQ410111, FJ766006, FJ8785	CK Hurley
DRB1*11:02:01	DRB1*110201	DR11(5)	Dw’JVM’	DRw11.2	JVM, LTI, 72393	M17382, AF029280, FM209426, FM995499	J Enczmann^b^, JDH Anholts^b^
DRB1*11:02:02	DRB1*110202	DR11(5)	—	—	NT00583, BY00267	DQ060440, EU256484	CK Hurley
DRB1*11:03	DRB1*1103	DR11(5)	—	DRw11.3	UA-S2, 23442	M21966, M22047-49, AJ507425	
DRB1*11:04:01	DRB1*110401	DR11(5)	Dw’FS'	—	FPA (FPF), 34A2, FPF, NA14661	AF029281, AJ297587, AY663394	(352)^b^
DRB1*11:04:02	DRB1*110402	DR11(5)	—	—	2094, 17A1	M34317, AF029282	
DRB1*11:04:03	DRB1*110403	DR11(5)	—	—	115376	AM000036	(372)
DRB1*11:04:04	DRB1*110404	DR11(5)	—	—	28807	AM491357	(373)
DRB1*11:04:05	DRB1*110405	DR11(5)	—	—	HN-70606-3	FJ217728	Histogenetics
DRB1*11:05	DRB1*1105	DR11(5)	—	—	DBUG, 163635	M84188, AM109979, AM409245	R Blasczyk^b^, H Dunckley^b^
DRB1*11:06:01	DRB1*110601	DR11(5)	—	DR11.CCY, 11PMH	CCY, PMH161, U210.DNA#5156, 43539, 92414	M98436, D14352, AY179367, AJ867236, FM994148	J Enczmann^b^
DRB1*11:06:02	DRB1*110602	DR11(5)	—	STF2432DRB1	O.G.DNA#24327, NT01117	AY170862, GQ892574	CK Hurley^b^
DRB1*11:07	DRB1*1107	DR11(5)^c^	—	DR11+3	BEL6KG, RMS21, GN00086	X73027, X82507, AY504812	
DRB1*11:08:01	DRB1*110801	DR11(5)	—	DR11JL	JL	L21984, AM109980	R Blasczyk^b^
DRB1*11:08:02	DRB1*110802	DR11(5)	—	DR11HW	HW	L21983, AM109981	R Blasczyk^b^
DRB1*11:09	DRB1*1109	DR11(5)	—	DRB1*MON	BEL7MON, GN00060	X75347, AY277387, AM109982	R Blasczyk^b^
DRB1*11:10:01	DRB1*111001	—	—	DR11.5	BRI-6, GN0094	L23986, AY504813	
DRB1*11:10:02	DRB1*111002	DR11(5)	—	—	LA177441	FM179954,	F Poli
DRB1*11:11:01	DRB1*111101	DR11(5)	—	DR11.6, DR11BRA, STF15775	BRI-7, 1082, S.D.DNA#15775, Terasaki B-Cell 144	L23990, L26306, AY174184, AY296120	
DRB1*11:11:02	DRB1*111102	DR11(5)	—	—	CTM-3095924	AY961065	(353)
DRB1*11:12:01	DRB1*111201	—	—	DR11.7	BRI-9, 008, GN00175, 44397	L23988, AF234175, AY277391, FM994152	J Enczmann^b^
DRB1*11:12:02	DRB1*111202	—	—	—	SWP71	AJ251984	
DRB1*11:13:01	DRB1*111301	DR11(5)	—	DR11-14, DR11+14	PAL-6117, 30251, EmKa, SB, BV0595, JOK, TER327	X76194, L29081, U09200, U03291, Z37162, X87677, AM109983	R Blasczyk^b^
DRB1*11:13:02	DRB1*111302	DR11(5)	—	—	97708, 185405	FM878944, FN295562	J Enczmann, K Witter^b^
DRB1*11:14:01	DRB1*111401	DR11(5)	—	F1363, 115T, 94-09865	BRI-11, HN0605, DJB, BEN, 12762, G.J.DNA#7201	U08932, Z37161, U25639, Z50187, AJ245714, AY179368, AM109984	R Blasczyk^b^
DRB1*11:14:02	DRB1*111402	DR11(5)	—	—	ALX0006, ALX0007	AY877348, AY884215	E Ball
DRB1*11:15	DRB1*1115	DR11(5)	—	DR1101v	Z.S., Z.Z., Z.Z.V., GN041, GN037, H282, CTM-1096184	Z34824, U17380, AJ697892, AY961066	(374)^b^
DRB1*11:16	DRB1*1116	DR11(5)^c^	—	DRB1*OULA, DR11+13	OULA, HB7542AKG	U13009, X87200	
DRB1*11:17	DRB1*1117	—	—	UCSF-D3152, DR11-14N, 0104D0335	D3152, D3153, GN032, 950104-D0335	X77776, U17379, U33474	
DRB1*11:18	DRB1*1118	—	—	RMS16	RMS16, 126512	X82211, FM994149	J Enczmann^b^
DRB1*11:19:01	DRB1*111901	DR11(5)	—	RMS117, DR11Loel	RMS117, MB, KBD, TER278, CS00020	X82210, Z47353, U26558, AM109985, GQ301205	R Blasczyk^b^, K Cao^b^
DRB1*11:19:02	DRB1*111902	DR11(5)	—	—	CBHLA-DRB1-01	AY750899	
DRB1*11:20	DRB1*1120	DR11(5)	—	—	CV	U25442	
DRB1*11:21	DRB1*1121	DR11(5)	—	—	MUL, Terasaki B-Cell 251, MUL, NT00790	X86976, AY296121, AM109986, EU643614	R Blasczyk^b^, CK Hurley^b^
DRB1*11:22	DRB1*1122	—	—	—	ZL3096	Z49113	
DRB1*11:23	DRB1*1123	DR11(5)	—	DRB1*11OS	YAS	D49468	
DRB1*11:24	DRB1*1124	—	—	7CGCE	JB, DZA95-7C	X89193, Z50746	
DRB1*11:25	DRB1*1125	DR11(5)	—	DR11x08	SimE, TAR	X91823, X97291	
DRB1*11:26	DRB1*1126	DR11(5)	—	DRB.W11	WAN	X94350	
DRB1*11:27:01	DRB1*112701	DR11(5)	—	2166/1018	M.K., 64449	X95656, fm994151	J Enczmann^b^
DRB1*11:27:02	DRB1*112702	DR11(5)	—	DRB1*11New	E404, E405, E434, NMDP0361-0724-1	AF186407, AF186408, AJ401148	
DRB1*11:28:01	DRB1*112801	—	—	DRB1*11Var	LELIEAM, 980102	X97722, AF047350	
DRB1*11:28:02	DRB1*112802	—	—	—	SZ-30	FJ870104	(375)
DRB1*11:29	DRB1*1129	DR11(5)	—	DRB1*11PBT	CL1281, 21690	X99841, AJ245715, AM109987	R Blasczyk^b^
DRB1*11:30	DRB1*1130	—	—	—	GN00153	U79027	
DRB1*11:31	DRB1*1131	—	—	DRB1*VIC	CTM4065412	U72064	
DRB1*11:32	DRB1*1132	—	—	MANDRAY Arlette	MA96401984, NT00742, 37479	AF011786, EU146147, FM994150	CK Hurley^b^, J Enczmann^b^
DRB1*11:33	DRB1*1133	—	—	DR11New, DRB1*JG	DU13673, BM1, 101910, NT00984	AF034858, AF112877, EU826133	CK Hurley^b^
DRB1*11:34	DRB1*1134	—	—	—	GN00236, 67364	AF081676, FM994153	J Enczmann^b^
DRB1*11:35	DRB1*1135	—	—	DRB1*TG	DIA3 128504, NT00986, 45982	AF112878, EU826131, FM994154	CK Hurley^b^, J Enczmann^b^
DRB1*11:36	DRB1*1136	—	—	DRB1*1102v	NT0001, BY00428	AF144081, FJ688169	CK Hurley^b^
DRB1*11:37	DRB1*1137	DR11(5)	—	DRB1*11LF	LIFU, HK-V	AJ249726, AJ252281	
DRB1*11:38	DRB1*1138	—	—	DRB1*CB3202	CB3202	AF247534	
DRB1*11:39	DRB1*1139	—	—	DRB1*CB1801	CB1801, DKM649157, 43096	AF267639, AJ404618, FM994155	J Enczmann^b^
DRB1*11:40	DRB1*1140	—	—	DRB1*11MMK	TO05334	AJ289124	
DRB1*11:41	DRB1*1141	—	—	DRB1*1103v	NT0011, NT00978	AF280436, EU812542	CK Hurley^b^
DRB1*11:42	DRB1*1142	—	—	—	FPO	AJ306404	
DRB1*11:43	DRB1*1143	—	—	DRB1*CB4551	CB4551	AF450093	
DRB1*11:44	DRB1*1144	—	—	SNOB-2	11167	AJ512947	
DRB1*11:45	DRB1*1145	—	—	—	19583, NT00504, DE168	AY307897, AY429723, AY465115	
DRB1*11:46	DRB1*1146	—	—	—	SARK, SARK-F	AJ534885	
DRB1*11:47	DRB1*1147	—	—	—	WLQ, WFQ, WWQ	AY305859	
DRB1*11:48	DRB1*1148	—	—	—	NT00507	AY429728	
DRB1*11:49	DRB1*1149	—	—	—	16006, 16005, 24115	AY172512	
DRB1*11:50	DRB1*1150	—	—	—	94274	AJ627279	
DRB1*11:51	DRB1*1151	—	—	—	R0921, BY00057	AY545466, AY664401	
DRB1*11:52	DRB1*1152	DR11(5)	—	—	4337, 4334	AY574194	
DRB1*11:53	DRB1*1153	—	—	—	2004_0843	AY641577	
DRB1*11:54:01	DRB1*115401	—	—	—	BY00056	AY664400	
DRB1*11:54:02	DRB1*115402	—	—	—	BY00110	DQ514604	(77)
DRB1*11:55	DRB1*1155	—	—	—	16068	AM231063	(376)
DRB1*11:56	DRB1*1156	—	—	—	NT00666	DQ525634	(355)
DRB1*11:57	DRB1*1157	—	—	—	NT00668	DQ535034	(163)
DRB1*11:58:01	DRB1*115801	—	—	—	100763, BY00252	AM409260, EU095398	H Dunckley, CK Hurley^b^
DRB1*11:58:02	DRB1*115802	—	—	—	HN-31092-9	FJ766005	Histogenetics
DRB1*11:59	DRB1*1159	—	—	—	169843	AM409259	H Dunckley
DRB1*11:60	DRB1*1160	—	—	—	174307	AM409258	H Dunckley
DRB1*11:61	DRB1*1161	—	—	—	157429	AM490640	(373)
DRB1*11:62	DRB1*1162	—	—	—	MHHN-158338	AM493248	R Blasczyk
DRB1*11:63	DRB1*1163	—	—	—	1103SV	AM493433	(357)
DRB1*11:64	DRB1*1164	—	—	—	BJ036	EF660334	N Liu
DRB1*11:65:01	DRB1*116501	—	—	—	BY00235, BY00234, BY00244, BY00243	EU084399, EU084400, EU095406, EU095407	(36)
DRB1*11:65:02	DRB1*116502	—	—	—	MMarsh1	EU090251	(377)
DRB1*11:66	DRB1*1166	—	—	—	BY00246, BY00249	EU095403, EU095404	(36)
DRB1*11:67	DRB1*1167	—	—	—	IME1278-06ROMA	EF640930	(378)
DRB1*11:68	DRB1*1168	—	—	—	HO013276	AM931006	(379)
DRB1*11:69	DRB1*1169	—	—	—	BY00327, HN-48662-3, BY00503, BY00550	EU716064, FJ217725, GQ410122, GU066761	CK Hurley, Histogenetics^b^
DRB1*11:70	DRB1*1170	—	—	—	31485	FM205011	J Enczmann
DRB1*11:72	DRB1*1172	—	—	—	BY00425, BY00527	FJ688166, GU066739	CK Hurley
DRB1*11:73	DRB1*1173	—	—	—	WangyiDR11	FJ810063	J He
DRB1*11:74	DRB1*1174	—	—	—	HN-51123-7, HN-51223-5	FJ205621, FJ205622	Histogenetics
DRB1*11:75	DRB1*1175	—	—	—	HN-50189-0	FJ205623	Histogenetics
DRB1*11:76	DRB1*1176	—	—	—	HN-07646-3, HN-41815-3, HN-62807-4, HN-77466-2, HN-87457-4	FJ205629, FJ205631, FJ205632, FJ217722, FJ952589	Histogenetics
DRB1*11:77	DRB1*1177	—	—	—	HN-62473-5	FJ205630	Histogenetics
DRB1*11:78	DRB1*1178	—	—	—	HN-74879-2, HN-96208-3	FJ205635, FJ766003	Histogenetics
DRB1*11:79	DRB1*1179	—	—	—	HN-27416-7	FJ217727	Histogenetics
DRB1*11:80	DRB1*1180	—	—	—	HN-9179433	FJ217729	Histogenetics
DRB1*11:81	DRB1*1181	—	—	—	HN-07650-3	FJ205616	Histogenetics
DRB1*11:82	DRB1*1182	—	—	—	JMDP01K058	AB512502	K Tadokoro
DRB1*11:83	DRB1*1183	—	—	—	BY00494	GQ410113	CK Hurley
DRB1*11:84	DRB1*1184	—	—	—	BY00496	GQ410115	CK Hurley
DRB1*11:85	DRB1*1185	—	—	—	BY00551	GU066762	CK Hurley
DRB1*11:86	DRB1*1186	—	—	—	BY00547	GU066758	CK Hurley
DRB1*11:87	DRB1*1187	—	—	—	BY00539	GU066751	CK Hurley
DRB1*11:88	DRB1*1188	—	—	—	HN-55677-7	FJ549415	Histogenetics
DRB1*11:89	DRB1*1189	—	—	—	09-2843	GU060679	M Curcio
DRB1*12:01:01	DRB1*120101	DR12(5)	Dw”DB6’	DRB1*EBROW	HERLUF, FO, HK, POPE, SWS53, EBROW, NA14660	M27635, M27509, S48645, AJ293695, AJ293696, AF335319, AF335320, AY663396	(352)^b^
DRB1*12:01:02	DRB1*120102	DR12(5)	—	—	BS464263	AJ293725, AJ302075	
DRB1*12:01:03	DRB1*120103	DR12(5)	—	—	1057830	GQ302514	E Palou
DRB1*12:02:01	DRB1*120201	DR12(5)	—	DRw12b	KI, C.V.DNA#24698	M27510, AY174091	
DRB1*12:02:02	DRB1*120202	DR12(5)	—	DRB1*1202X	BP-9, BP-21	L34353	
DRB1*12:02:03	DRB1*120203	DR12(5)	—	—	JMDP36K021	AB436796	K Tadokoro
DRB1*12:02:04	DRB1*120204	DR12(5)	—	—	B1753	EU088012	(380)
DRB1*12:03:02	DRB1*120302	DR12(5)	—	DRB1*12JBT	T00341	X83455	
DRB1*12:04	DRB1*1204	—	—	MHT#12v	MHT#918, GN00252	U39087, AY339246	
DRB1*12:05	DRB1*1205	DR12(5)	—	—	JC2862	D86503	
DRB1*12:06	DRB1*1206	DR12(5)	—	DRB1*12XX	K-KT	U95989, AF017439	
DRB1*12:07	DRB1*1207	—	—	DRB1*TCOX	TCOX	AF315825, AF316619	
DRB1*12:08	DRB1*1208	—	—	DRB1*12 variant	13365831, BY00335	AY033428, EU812538	CK Hurley^b^
DRB1*12:09	DRB1*1209	—	—	—	22540	AB112911	
DRB1*12:10	DRB1*1210	—	—	—	SW	AY626551	
DRB1*12:11	DRB1*1211	—	—	—	MHH0208244, CS00023	AJ870927, GQ845416	K Cao^b^
DRB1*12:12	DRB1*1212	—	—	—	B4550	AY899825	(381)
DRB1*12:13	DRB1*1213	—	—	—	2005081802	DQ250650	(382)
DRB1*12:14	DRB1*1214	—	—	—	KS9407622	DQ343834	(383)
DRB1*12:15	DRB1*1215	—	—	—	KS9504263	DQ533486	(383)
DRB1*12:16	DRB1*1216	—	—	—	45112875	EF688603	C Zhang
DRB1*12:17	DRB1*1217	—	—	—	049-B6	EU375850	(384)
DRB1*12:18	DRB1*1218	—	—	DRB1*12	SZK1	FJ481086	(385)
DRB1*12:19	DRB1*1219	—	—	—	SDBC-HLA-zwy	Fj374889	(386)
DRB1*12:20	DRB1*1220	—	—	—	BY00475	GQ373162	CK Hurley
DRB1*13:01:01	DRB1*130101	DR13(6)	Dw18	DRw6a I, DR1301Var	HHKB, APD, W468R, W468D, NA03715	M17383, X04056, U83583, AY663415	(352)^b^
DRB1*13:01:02	DRB1*130102	DR13(6)	—	DRB1*13new	19783VO	AJ271206	
DRB1*13:01:03	DRB1*130103	DR13(6)	—	—	GN00424	AY178184	
DRB1*13:01:04	DRB1*130104	DR13(6)	—	—	HN-82107-5	FJ205628	Histogenetics
DRB1*13:01:05	DRB1*130105	DR13(6)	—	—	HN-52132-3	FJ217723	Histogenetics
DRB1*13:01:06	DRB1*130106	DR13(6)	—	—	BY00542	GU066753	CK Hurley
DRB1*13:02:01	DRB1*130201	DR13(6)	Dw19	DRw6c I, DR1302Var	WT46, CMCC, AS, W556R, W556D, NA14663	L76133, U83584, AY663413	(352)
DRB1*13:02:02	DRB1*130202	DR13(6)	—	DRB1*RMAY, FM99/810	RMAY, FM99/810	AF176834, AF217961	
DRB1*13:02:03	DRB1*130203	DR13(6)	—	—	CB19616	DQ658416	A Anand
DRB1*13:03:01	DRB1*130301	DR13(6)	Dw’HAG’	—	HAG, MRS, EGS, OSC, MGA, JRS, 1181, 1183, 2708, IH, JS, MD, SK, TER275	X52451, X16649, M59798, M57599, AM109988	R Blasczyk^b^
DRB1*13:03:02	DRB1*130302	DR13(6)	Dw’HAG’	—	11118-CMN, 22127-EC	U41634, U34602	
DRB1*13:03:03	DRB1*130303	DR13(6)	—	—	BY00512	GQ426487	CK Hurley
DRB1*13:03:04	DRB1*130304	DR13(6)	—	—	HN-64842-5	FJ858909, FJ858910	Histogenetics
DRB1*13:04	DRB1*1304	DR13(6)	—	RB1125-14	1124, 1125, 40267	M59803, AM949862	T Gervais^b^
DRB1*13:05:01	DRB1*130501	DR13(6)	—	DRw6’PEV’,	TA, JP, HS, BP, DES.DI, SUDNA0165, 17A2, H305M	M57600, L78167, AF029283, AJ697893	
DRB1*13:05:02	DRB1*130502	DR13(6)	—	—	NT00610	DQ135944	(107)
DRB1*13:06	DRB1*1306	DR13(6)	—	DRB1*13.MW	MW, 26481, GN00089, TER331	M81343, AJ507382, AY277389, AM109989	R Blasczyk^b^
DRB1*13:07:01	DRB1*130701	DR13(6)	—	DRB1*JJY, DRB1*SHN	JJY, SHN, SLIR1-13	L06847, D13189, AF305212	
DRB1*13:07:02	DRB1*130702	DR13(6)	—	—	GN00185	AF036944	
DRB1*13:08	DRB1*1308	DR13(6)	—	—	THA	L03531, AM109990	R Blasczyk^b^
DRB1*13:09	DRB1*1309	—	—	DRB1*YUN	MJD, NT01111	L23534, GQ373164	CK Hurley^b^
DRB1*13:10	DRB1*1310	DR13(6)	—	13NEW	ARA, 13345532, 13976036, GN00084, TER343	X75442, AJ245716, AJ409215, AY277388, AM109991	R Blasczyk^b^
DRB1*13:11:01	DRB1*131101	DR13(6)	—	1303-Like	H108, HER-2698, 1083933x	X74313, X75445, AJ243898, AM109992	R Blasczyk^b^
DRB1*13:11:02	DRB1*131102	DR13(6)	—	—	BY00337, HN-98756-7	EU812540, FJ907390	CK Hurley, Histogenetics^b^
DRB1*13:12	DRB1*1312	DR13(6)^c^	—	DR13BRA, DR13.7	650, 651, 681, BRI-8, N170, CC75, AD-6168, DNAQC012, RMS103	L25427, L23989, D29836, L27216, X82508	
DRB1*13:13	DRB1*1313	DR13(6)^c^	—	DRB1*13/8	NORH01, NORH02, XX406	U79025, U79026, Y17272	
DRB1*13:14:01	DRB1*131401	DR13(6)	—	1101A58, 13New	BRI-12, YAS, 11684232, GN00270	U08274, X82239, AJ245717, AY277393	
DRB1*13:14:02	DRB1*131402	DR13(6)	—	DRB1*13MJ	31854	AJ243897, AM109993	R Blasczyk^b^
DRB1*13:14:03	DRB1*131403	DR13(6)	—	—	HO007970	AM931007	(379)
DRB1*13:15	DRB1*1315	—	—	83-7601, STF9107	BRI-14, GN070, T.D.DNA#9107	U08276, U32325, AY174182	
DRB1*13:16	DRB1*1316	DR13(6)	—	DRB1*D86	BRI-15, JA, GN00233	U08277, U25638, AY277392	
DRB1*13:17	DRB1*1317	DR13(6)	—	RB1194 13/12	R.B	U03721, AM109994	R Blasczyk^b^
DRB1*13:18	DRB1*1318	DR13(6)	—	DRB1*13HZ	K27418, TH10913, ZAN FR	Z36884, X82549, Z48631	
DRB1*13:19	DRB1*1319	DR13(6)^c^	—	DR1308V	GN033, NT00772	U17381, EU375813	CK Hurley^b^
DRB1*13:20	DRB1*1320	DR13(6)	—	DRB1*13VHT, DRB1*13PL	SR0300, 10843566	Z48803, Y17695, AM109995	R Blasczyk^b^
DRB1*13:21:01	DRB1*132101	—	—	DR13TAS	ATAS, 186105	L41992, AM409249	H Dunckley^b^
DRB1*13:21:02	DRB1*132102	—	—	—	HN-50459-4, HN-64798-8, HN-65133-7, HN-55271-7, HN-50682-0, HN-57306-9	FJ766012, FJ858912, FJ875605, FJ875606, FJ875607, FJ858911	Histogenetics
DRB1*13:22	DRB1*1322	DR13(6)^c^	—	—	GvdP, LI3936	X86326, X87886	
DRB1*13:23	DRB1*1323	—	—	—	GN079	U36827	
DRB1*13:24	DRB1*1324	—	—	—	GN039	U36825	
DRB1*13:25	DRB1*1325	—	—	—	MRN5981	X93924	
DRB1*13:26	DRB1*1326	—	—	DRB1*16WIL, DRB1*14/16New	WIL3966, B.A-B	X96396, Y11462	
DRB1*13:27	DRB1*1327	DR13(6)	—	DRB1*13MS, DRB1*13NW	NVE 802, CTM-6095542	Z71289, U59691, X97601, AY961070	(353)^b^
DRB1*13:28	DRB1*1328	—	—	—	DU25503	X97407	
DRB1*13:29	DRB1*1329	DR13(6)^c^	—	—	JC6267, 29447, TER320	D87822, AJ506752, AM109996	R Blasczyk^b^
DRB1*13:30	DRB1*1330	—	—	DRB1*13DAS	DAS-094	U72264	
DRB1*13:31	DRB1*1331	—	—	—	GN00133, GN00138, NT00661, BY00223	U88133, U88134, DQ525629, EU071691	CK Hurley^b^
DRB1*13:32	DRB1*1332	—	—	DR13MC	AD-2111	U97554	
DRB1*13:33:01	DRB1*133301	—	—	DRB1*13TMT	OTO1567	AJ001254	
DRB1*13:33:02	DRB1*133302	—	—	—	HN-10912-6	FJ205642	Histogenetics
DRB1*13:33:03	DRB1*133303	—	—	—	HN-015227-4, P-658554	FJ205643, FN430726	Histogenetics, R Blasczyk^b^
DRB1*13:34	DRB1*1334	—	—	—	974770	AF048688	
DRB1*13:35	DRB1*1335	—	—	DRB1*13Var	GN00266-FV2397	AF136155	
DRB1*13:36	DRB1*1336	DR13(6)	—	DR’RD’, DRB1*JSMA	RD-DJ, AA-DJ, JSMA, 30638, IND-1	AF089719, AF195786, AJ293898, AM109997	R Blasczyk^b^
DRB1*13:37	DRB1*1337	—	—	DRB1*13New	GN00256, NT0003	AF169238, AF164346	
DRB1*13:38	DRB1*1338	—	—	DRB1*13New	031188956, NT00983, HN-32101-5, HN-61399-0, HN-8904109, HN-74601-2, HN-11283-8	AF169239, EU826134, FJ594734, FJ766009, FJ875597, FJ875608, FJ952588	CK Hurley^b^, Histogenetics^b^
DRB1*13:39	DRB1*1339	—	—	DRB1*13PSB	KMDP01-415	AF170582, AF104018	
DRB1*13:40	DRB1*1340	—	—	DRB1*13JP	NE3114, NE3005	AJ237964	
DRB1*13:41	DRB1*1341	—	—	DRB1*Laton	Laton, NT00763	AJ249591, EU330462	CK Hurley^b^
DRB1*13:42	DRB1*1342	DR13(6)	—	DRB1*1318V	NT0010, AN3SP6	AF243537, AF288212	
DRB1*13:43	DRB1*1343	—	—	DRB1*14New	GN00221	AF243538	
DRB1*13:44	DRB1*1344	—	—	DRB1*GDES	GDES	AF247533	
DRB1*13:45	DRB1*1345	—	—	—	SG606319	AJ276873	
DRB1*13:46	DRB1*1346	—	—	DRB1*AHAW	AHAW	AF306862	
DRB1*13:47	DRB1*1347	—	—	DRB1*1307V1	JCB12184	AB049459	
DRB1*13:48	DRB1*1348	—	—	—	20281	AJ401236	
DRB1*13:49	DRB1*1349	—	—	DRB1*1312var	NT0023	AF352295	
DRB1*13:50:01	DRB1*135001	—	—	—	1DM4038S1, BY00138	AY048687, EF078987	CK Hurley^b^
DRB1*13:50:02	DRB1*135002	—	—	—	CB5046	EU290674	(387)
DRB1*13:51	DRB1*1351	—	—	—	LPC14	AF441789	
DRB1*13:52	DRB1*1352	DR13(6)	—	—	R.171, CBS1246M	AF499445, AJ421519	
DRB1*13:53	DRB1*1353	—	—	—	119676	AJ488066	
DRB1*13:54	DRB1*1354	DR14(6)	—	—	AM-1354, 70289	AJ491301, AB112913	
DRB1*13:55	DRB1*1355	—	—	—	C.D.DNA#8837	AY179366	
DRB1*13:56	DRB1*1356	—	—	DR13JJJ	G042402008760X, CBRL 9-23-010, NT00500, CTM-1097120	AY257483, AY259128, AY396024, AY961069	(353)^b^
DRB1*13:57	DRB1*1357	DR13(6)	—	—	030126Koe	AJ555156	
DRB1*13:58	DRB1*1358	—	—	DR13AAC	CBRL 6-07-172	AY259126	
DRB1*13:59	DRB1*1359	—	—	DR13HYC	CBRL 6-09-223, 292/03_R933	AY259127, AJ627565	
DRB1*13:60	DRB1*1360	—	—	—	SL06	AY178845	
DRB1*13:61	DRB1*1361	—	—	—	BY00049	AY339247	
DRB1*13:62	DRB1*1362	—	—	—	234715	AY379480	
DRB1*13:63	DRB1*1363	—	—	—	ULM 12662	AY502108	
DRB1*13:64	DRB1*1364	—	—	—	Rosario-SR	AJ634529	
DRB1*13:65	DRB1*1365	—	—	—	HSR113418	AJ783982	
DRB1*13:66	DRB1*1366	DR13(6)	—	—	242-04ANZALONE	AY765349	
DRB1*13:67	DRB1*1367	—	—	DRB1*13MM	A0425169	AJ853708	
DRB1*13:68	DRB1*1368	—	—	—	952An	AY963587	(388)
DRB1*13:69	DRB1*1369	—	—	—	R222	AY225520	SG Rodriguez-Marino
DRB1*13:70	DRB1*1370	—	—	DRB1*13MVE0805	MHHZ-00014883, MHHZ-0001484	AM086025	(389)
DRB1*13:71	DRB1*1371	—	—	—	ALSM4092AN	AM158254	(390)
DRB1*13:72	DRB1*1372	—	—	—	BY00102	DQ473293	(57)
DRB1*13:73	DRB1*1373	—	—	—	BY00108	DQ514602	(77)
DRB1*13:74	DRB1*1374	—	—	—	K107946	AM279417	C Dunne
DRB1*13:75	DRB1*1375	—	—	—	2551087	EF053230	(391)
DRB1*13:76	DRB1*1376	—	—	—	SP-32720	EF196873	(99)
DRB1*13:77	DRB1*1377	—	—	—	TO23117	AM494414	(357)
DRB1*13:78	DRB1*1378	—	—	—	VTIS77100	EF493832	BD Tait
DRB1*13:79	DRB1*1379	—	—	—	VTIS124307, BY00505	EF493833, GQ410090	BD Tait, CK Hurley^b^
DRB1*13:80	DRB1*1380	—	—	—	BY00221	EU029803	(36)
DRB1*13:81	DRB1*1381	—	—	—	BY00230, BY00429	EU071684, FJ688170	(36), CK Hurley^b^
DRB1*13:82	DRB1*1382	—	—	—	LUMC-DR36, BY00329, BY00336	AM904556, EU716066, EU812539	(64), CK Hurley^b^
DRB1*13:83	DRB1*1383	—	—	—	MHHN-611087	AM931066	R Blasczyk
DRB1*13:84	DRB1*1384	—	—	—	BY00338	EU812541	CK Hurley
DRB1*13:85	DRB1*1385	—	—	—	BY00340	EU826130	CK Hurley
DRB1*13:86	DRB1*1386	—	—	—	BY00339	EU826129	CK Hurley
DRB1*13:87	DRB1*1387	—	—	—	30195, HN-66888-8	FM196526, FJ205619	J Enczmann, Histogenetics^b^
DRB1*13:88	DRB1*1388	—	—	—	BY00422	FJ688163	CK Hurley
DRB1*13:89	DRB1*1389	—	—	—	HN-80814-7	FJ205624	Histogenetics
DRB1*13:90	DRB1*1390	—	—	—	HN-98148-0, HN-98052-4, 184892	FJ205626, FJ217721, FN423501	Histogenetics, E Keller^b^
DRB1*13:91	DRB1*1391	—	—	—	HN-23595-3, HN-62612-1	FJ205636, FJ205641	Histogenetics
DRB1*13:92	DRB1*1392	—	—	—	HN-1563225	FJ217724	Histogenetics
DRB1*13:93	DRB1*1393	—	—	—	BY00491	GQ410110	CK Hurley
DRB1*13:94	DRB1*1394	—	—	—	BY00493	GQ410112	CK Hurley
DRB1*13:95	DRB1*1395	—	—	—	HN-67038-8	FJ640587	Histogenetics
DRB1*13:96	DRB1*1396	—	—	—	HN-66141-1	FJ640586	Histogenetics
DRB1*13:97	DRB1*1397	—	—	—	HN-26392-8	FJ766011	Histogenetics
DRB1*14:01:01	DRB1*140101	DR14(6)	Dw9	DRw6b I	4/w6, TEM, 15B1	X04057, AF029284, AJ297582	
DRB1*14:01:02	DRB1*140102	DR14(6)	Dw9	DRB1*14ML	BV17214	AJ289123	
DRB1*14:01:03	DRB1*140103	DR14(6)	—	—	TW3	DQ021915	TD Lee
DRB1*14:02	DRB1*1402	DR14(6)	Dw16	—	AMALA (LIA,AZL)^e^, 15B3	AF029285, AJ297583	
DRB1*14:03:01	DRB1*140301	DR1403	—	JX6	MI	AJ297584	
DRB1*14:03:02	DRB1*140302	DR1403		—	17057	AB112912	
DRB1*14:04	DRB1*1404	DR1404	—	DRB1*LY10, DRw6b.2	CEPH-137502, KGU	M58632, AJ297585	
DRB1*14:05:01	DRB1*140501	DR14(6)	—	DRB1*14c	36M, 38M, SUDNA0503, GN00402, GN00404, EBSM, NA04535	M60209, L78168, AY050209, AY050210, AB062112, AY663408	(352)^b^
DRB1*14:05:02	DRB1*140502	DR14(6)	—	—	2247077-4	AY129430	
DRB1*14:05:03	DRB1*140503	DR14(6)	—	—	2006071961	EF103190	(392)
DRB1*14:06:01	DRB1*140601	DR14(6)	—	DRB1*14.GB, 14.6	GB, SAS5041, SAS9080, SUDNA0164, 24A3, GN00405, GN00407, TER313	M63927, M74032, L78164, AF029286, AY050211, AY050214, AM109998	R Blasczyk^b^
DRB1*14:06:02	DRB1*140602	DR14(6)	—	—	1085896	GQ302516	E Palou
DRB1*14:07:01	DRB1*140701	DR14(6)	—	14.7	PNG141, PNG196, 43A1, GN00400, GN00401, HAY, BD	M74030, AF029287, AY050207, AM109999	R Blasczyk^b^
DRB1*14:07:02	DRB1*140702	DR14(6)	—	—	GN00403	AY052549	
DRB1*14:08	DRB1*1408	DR14(6)^c^	—	AO1,14.8	HV178, PNG198, PNG202, GN00409	M77673, M74031, AY052550	
DRB1*14:09	DRB1*1409	—	—	AB4, STF10173	1103, C.D.DNA#10173	M77671, AY174181	
DRB1*14:10	DRB1*1410	DR14(6)	—	AB3	ABCC31, TER283	M77670, AM110000	R Blasczyk^b^
DRB1*14:11	DRB1*1411	DR14(6)	—	DRw14x11	MARBrun, MARMari, MARMarg, CTM-7095922	M84238, AY961071	(353)^b^
DRB1*14:12	DRB1*1412	DR14(6)	—	DRB1*YOS	YOS, AS03092603	D16110, AY770520	
DRB1*14:13	DRB1*1413	DR14(6)	—	—	GRC138	L21755, AM110001	R Blasczyk^b^
DRB1*14:14	DRB1*1414	DR14(6)	—	DRB1*14N	AD-2927, AD-3798, IHL AD036	L17044, AM110002	R Blasczyk^b^
DRB1*14:15	DRB1*1415	DR8	—	DRB1*14af	D.M., R03-353	U02561, AJ581744	
DRB1*14:16	DRB1*1416	DR6	—	DR13+14	FVA-0166, 18573	X76195, AJ508388	
DRB1*14:17	DRB1*1417	DR6	—	1412T	#15310-LN, 12499	X76938, AJ543433	
DRB1*14:18	DRB1*1418	DR6	—	81-4641	BRI-13, TH6994, DR14BBD, GN00096	U08275, X82552, U37264, AY277390	
DRB1*14:19	DRB1*1419	DR14(6)	—	DRB1*14MA, DRB.14a	MA-TE, AKKAL	Z38072, X86973	
DRB1*14:20	DRB1*1420	DR14(6)	—	DRB.14o	OND-52971	X86974	
DRB1*14:21	DRB1*1421	DR14(6)^c^	—	DRB.14t	TGI	X86975, AM110003	R Blasczyk^b^
DRB1*14:22	DRB1*1422	DR14(6)^c^	—	DRB1*BA	LS005, BA	Z50730, Z71275	
DRB1*14:23:01	DRB1*142301	—	—	DRB1*14	#66820, SAR	X91640, Z84375	
DRB1*14:23:02	DRB1*142302	—		—	169255	AJ812566	
DRB1*14:23:03	DRB1*142303	—	—	—	JMDP36K048	AB512506	K Tadokoro
DRB1*14:24	DRB1*1424	—	—	BY14V, BRAVOG, DRB1*14Pal	BY00002, HDB, PALT, SERL, TER411	U41489, AJ000900, AF052574, FM179681	A Dormoy^b^
DRB1*14:25	DRB1*1425	—	—	HL14V, STF16406	HL.BWH, MF.BWH, H.S.DNA#16406	U41490, U41491, AY174092	
DRB1*14:26	DRB1*1426	DR14(6)	—	—	JC1980	D86502, D50865	
DRB1*14:27	DRB1*1427	DR14(6)	—	—	MO52	D86504	
DRB1*14:28	DRB1*1428	—	—	DRB1*14DKT	TO4138	X99839	
DRB1*14:29	DRB1*1429	DR14(6)	—	—	JC6094, NT00985	D88310, EU826132	CK Hurley^b^
DRB1*14:30	DRB1*1430	—	—	DRB1*14CB	CB-254, 87816	U95115	H Dunckley^b^
DRB1*14:31	DRB1*1431	—	—	DRB1*14JV	RP-JV129	AF028010	
DRB1*14:32:01	DRB1*143201	—	—	DRB1*14JW	GAIB	AJ010982	
DRB1*14:32:02	DRB1*143202	—	—	—	MHHN-159365	AM493247	R Blasczyk
DRB1*14:33	DRB1*1433	—	—	DRB1*LAM	CB1 116643, LB65292	AF112879, AJ506201	
DRB1*14:34	DRB1*1434	—	—	—	R98-333250Q	AF172071	
DRB1*14:35	DRB1*1435	—	—	DRB1*SDAV	SDAV	AF177215	
DRB1*14:36	DRB1*1436	—	—	DRB1*New	IHL	AJ242985	
DRB1*14:37	DRB1*1437	—	—	DRB1*1309New	SWP43	AJ251985	
DRB1*14:38	DRB1*1438	—	—	DRB1*1401V1	JCB14069	AB049830	
DRB1*14:39	DRB1*1439	—	—	DRB1*1401V2	JCB15932	AB049831	
DRB1*14:40:01	DRB1*144001	—	—	DRB1*1403V2	JCB24742	AB049832	
DRB1*14:40:02	DRB1*144002	—	—	—	BY00215	EU029797	(36)
DRB1*14:41	DRB1*1441	—	—	—	04RCH28	AY050186, AF339884	
DRB1*14:42	DRB1*1442	—	—	—	GN00411	AY054375	
DRB1*14:43	DRB1*1443	—	—	—	P87043M1	AF400066	
DRB1*14:44	DRB1*1444	—	—	DRB1*1405V1	TBC24477	AB087875	
DRB1*14:45	DRB1*1445	—	—	DRB1*1405V2	TBC28533	AB087876	
DRB1*14:46	DRB1*1446	—	—	—	19687, CTM-9097443	AJ515905, AY935719	(353)^b^
DRB1*14:47	DRB1*1447	—	—	—	BY00050	AY267905	
DRB1*14:48	DRB1*1448	—	—	—	BY00051, NT00981	AY267906, EU812545	CK Hurley^b^
DRB1*14:49	DRB1*1449	DR14(6)	—	—	NJ5627, BY00127	AY912075, DQ882243	(393), CK Hurley^b^
DRB1*14:50	DRB1*1450	DR14(6)	—	—	14oem	AJ969417	(394)
DRB1*14:51	DRB1*1451	—	—	—	NT00584	DQ060439	CK Hurley
DRB1*14:52	DRB1*1452	—	—	—	NT00582	DQ060441	CK Hurley
DRB1*14:53	DRB1*1453	DR13(6)	—	—	BISLo	AM084908	A Dormoy
DRB1*14:54	DRB1*1454	—	—	DRB1*14MAC0905	KOSE, EK, 32511, TER293, NA10540, B07-27, B07-1330	AM087553, AY663405, FJ379259	(395), (352)^b^, KW Lee^b^
DRB1*14:55	DRB1*1455	—	—	—	259316	DQ327711	K Hirv
DRB1*14:56	DRB1*1456	—	—	—	2005111516	DQ333353	(396)
DRB1*14:57	DRB1*1457	—	—	—	12973	DQ235685, DQ390459, DQ390460	(164)
DRB1*14:58	DRB1*1458	—	—	—	PL4737	DQ358688	(397)
DRB1*14:59	DRB1*1459	—	—	—	WEIFr	AM233907	A Dormoy
DRB1*14:60	DRB1*1460	DR14(6)	—	—	DR14VEDA	AM259285	(398)
DRB1*14:61	DRB1*1461	—	—	—	BJ003, BY00213	DQ494324, EU029795	(399), CK Hurley^b^
DRB1*14:62	DRB1*1462	—	—	—	BY00118	DQ782331	(57)
DRB1*14:63	DRB1*1463	—	—	—	Xian7454	DQ643390	S Ye
DRB1*14:64	DRB1*1464	—	—	—	BY00136	EF078986	(355)
DRB1*14:65	DRB1*1465	—	—	—	HE-AJM	EF199810	(99)
DRB1*14:67	DRB1*1467	—	—	—	CTJ-15231	EF495154	(400)
DRB1*14:68	DRB1*1468	—	—	—	NT00729, BY00217	EF536016, EU029799	(355), CK Hurley^b^
DRB1*14:69	DRB1*1469	—	—	—	BY00210	EU029792	(36)
DRB1*14:70	DRB1*1470	—	—	—	BY00207, BY00504	EU029788, GQ410089	(36), CK Hurley^b^
DRB1*14:71	DRB1*1471	—	—	—	BY00206	EU029787	(36)
DRB1*14:72	DRB1*1472	—	—	—	BY00211, BY00330	EU029793, EU716067	(36)
DRB1*14:73	DRB1*1473	—	—	—	BY00216	EU029798	(36)
DRB1*14:74	DRB1*1474	—	—	—	BS640629, 39756	AM774161, AM982721	(261), T Gervais^b^
DRB1*14:75	DRB1*1475	—	—	—	2647934	AM922324	(401)
DRB1*14:76	DRB1*1476	—	—	—	HO013533	AM933133	S Tavoularis
DRB1*14:77	DRB1*1477	—	—	—	CTJ-18807	EU545181	L Yan
DRB1*14:78	DRB1*1478	—	—	—	HistoCB69685	EU588391	(402)
DRB1*14:79	DRB1*1479	—	—	—	NT00788	EU643616	CK Hurley
DRB1*14:80	DRB1*1480	—	—	—	JMDP01K030	AB436778	K Tadokoro
DRB1*14:81	DRB1*1481	—	—	—	BY00334	EU812536	CK Hurley
DRB1*14:82	DRB1*1482	—	—	—	45161, HN-53927-1	FM196525, FJ205633	J Enczmann, Histogenetics^b^
DRB1*14:83	DRB1*1483	—	—	—	BY00347	EU924810	CK Hurley
DRB1*14:84	DRB1*1484	—	—	—	HB080101205	FJ594768	TD Lee
DRB1*14:85	DRB1*1485	—	—	—	K26647	AB485773	E Maruya
DRB1*14:86	DRB1*1486	—	—	—	021084-CB	FN186134	F Poli
DRB1*14:87	DRB1*1487	—	—	—	HN-47902-1, HN-452094	FJ205620	Histogenetics
DRB1*14:88	DRB1*1488	—	—	—	HN-93263-2	FJ205625	Histogenetics
DRB1*14:89	DRB1*1489	—	—	—	HN-10516-7	FJ205644	Histogenetics
DRB1*14:90	DRB1*1490	—	—	—	HN-37025-8	FJ205645	Histogenetics
DRB1*14:91	DRB1*1491	—	—	—	073069	GQ202277	B Zhang
DRB1*14:92N	DRB1*1492N	Null	—	—	0601638	GQ302515	E Palou
DRB1*14:93	DRB1*1493	—	—	—	BY00549	GU066760	CK Hurley
DRB1*14:94	DRB1*1494	—	—	—	BY00540	GU066764	CK Hurley
DRB1*14:95	DRB1*1495	—	—	—	HN-51663-1	FJ502336	Histogenetics
DRB1*14:96	DRB1*1496	—	—	—	09-2790	GU186854	KW Kim
DRB1*15:01:01:01	DRB1*15010101	DR15(2)	Dw2	DR2B Dw2	PGF, ROF-NL, 192762	M17378, M16957, M20430, AL713966, FM955270	(29)^b^, K Witter^b^
DRB1*15:01:01:02	DRB1*15010102	DR15(2)	—	—	NA10923	AY663406	(352)
DRB1*15:01:02	DRB1*150102	DR15(2)	Dw2	DRB1*15MT	LD0797	Z48359	
DRB1*15:01:03	DRB1*150103	DR15(2)	Dw2	DRB1*15011var	BY00017	AF363727	
DRB1*15:01:04	DRB1*150104	DR15(2)	Dw2	—	R24489	AJ431718	
DRB1*15:01:05	DRB1*150105	DR15(2)	Dw2	—	TBC-B23250	AB106127	
DRB1*15:01:06	DRB1*150106	DR15(2)	—	—	BY00109, BY00107, BY00140, BY00143	DQ514603, DQ514601, EF078985, EF156366	(77), CK Hurley^b^
DRB1*15:01:07	DRB1*150107	DR15(2)	—	—	BY00495	GQ410114	CK Hurley
DRB1*15:01:08	DRB1*150108	DR15(2)	—	—	HN-13337-9, HN-04135-0, HN-63876-6, HN-09686-6, HN-99481-4, HN-77509-8, HN-69270-9, HN-30621-9, HN-53372-1, HN-92340-3, HN-52503-7, HN-41926-3, HN-88103-3, HN-81593-3	FJ358153, FJ358164, FJ358154, FJ358161, FJ358162, FJ358163, FJ358172, FJ358173, FJ358174, FJ358177, FJ766013, FJ875604, FJ888507, FJ917720	Histogenetics
DRB1*15:01:09	DRB1*150109	DR15(2)	—	—	HN-04748-1	FJ358171	Histogenetics
DRB1*15:01:10	DRB1*150110	DR15(2)	—	—	HN-30368-4	FJ358155	Histogenetics
DRB1*15:01:11	DRB1*150111	DR15(2)	—	—	HN-55222-3, HN-62717-0	FJ358157, FJ875603	Histogenetics
DRB1*15:01:12	DRB1*150112	DR15(2)	—	—	HN-17926-7	FJ358169	Histogenetics
DRB1*15:02:01	DRB1*150201	DR15(2)	Dw12	DR2B Dw12	BGE, DHO, 20A1, NA03715	M16958, M30180, M28584, AF029289, AY663414	(352)^b^
DRB1*15:02:02	DRB1*150202	DR15(2)	Dw12	DR2MU	CMURD, THA	L23964, AM110004	R Blasczyk^b^
DRB1*15:02:03	DRB1*150203	DR15(2)	—	DRB1*15JMT	HN08729	AJ001253	
DRB1*15:02:04	DRB1*150204	DR15(2)	—	—	99593, NT00683	AM409257, EF078988	H Dunckley, (163)
DRB1*15:02:05	DRB1*150205	DR15(2)	—	—	P2976	EU523122	(403)
DRB1*15:02:06	DRB1*150206	DR15(2)	—	—	JMDP36K022	AB436797	K Tadokoro
DRB1*15:02:07	DRB1*150207	DR15(2)	—	—	JMDP01K059	AB512503	K Tadokoro
DRB1*15:03:01:01	DRB1*15030101	DR15(2)	—	—	G247, M851, M848, 20A2, CTM-7096536, NA14660	M35159, AF010142, AF029290, AY961072, AY663395	(353)^b^, (352)^b^
DRB1*15:03:01:02	DRB1*15030102	DR15(2)	—	—	NA14663	AY663411	(352)
DRB1*15:04	DRB1*1504	DR15(2)^c^	—	DR2DAI	D13, D53, HM, CTM-3096474	L23963, L34025, AY96073	(353)^b^
DRB1*15:05	DRB1*1505	DR15(2)	—	DRB1*15KY	K.W.	D49823	
DRB1*15:06	DRB1*1506	DR15(2)	—	STF22025	JB317836, RP, CANSIN009, INDRAN001, INDRAN003, P.M.DNA#22025	D63586, U45999, X98256, AY174180	
DRB1*15:07	DRB1*1507	DR15(2)^c^	—	DRB1*15LJM	UBM12218693	Y15404, AM110005	R Blasczyk^b^
DRB1*15:08	DRB1*1508	DR2	—	DRB1*15021V	JC3399	AB007634	
DRB1*15:09	DRB1*1509	—	—	—	R98-903841B	AF172070	
DRB1*15:10	DRB1*1510	—	—	—	98-2028, 98-2500, GN00320, BY00427	AF191104, AF243536, FJ688168	CK Hurley^b^
DRB1*15:11	DRB1*1511	—	—	—	NR-GLW, BY00452	AJ293861, FJ842963	CK Hurley^b^
DRB1*15:12	DRB1*1512	—	—	—	VTIS24502	AF373015	
DRB1*15:13	DRB1*1513	—	—	DRB1*TT68	TT68	AF239244	
DRB1*15:14	DRB1*1514	—	—	—	BY00052, NT000508	AY429729, AY525097	
DRB1*15:15	DRB1*1515	—	—	—	TBC44279, BY00220	AB176445, EU0298002	CK Hurley^b^
DRB1*15:16	DRB1*1516	—	—	—	D086	AY714542	
DRB1*15:17N	DRB1*1517N	Null	—	—	54101738	AJ875013	(404)
DRB1*15:18	DRB1*1518	—	—	—	DORFra, 1053612	AM056024, EU375479	A Dormoy, G Wohlwend^b^
DRB1*15:19	DRB1*1519	—	—	—	29477	AM040719	EM vd Berg Loonen
DRB1*15:20	DRB1*1520	—	—	—	DR15donN, BY00231	AM258966, EU071683	(405), CK Hurley^b^
DRB1*15:21	DRB1*1521	—	—	—	BY00113	DQ525633	CK Hurley
DRB1*15:22	DRB1*1522	—	—	—	NT00665, I077864	DQ525632, EU375478	(355), G Wohlwend^b^
DRB1*15:23	DRB1*1523	—	—	—	BY00222, BY00233	EU029804, EU071681	(36)
DRB1*15:24	DRB1*1524	—	—	—	BY00218, BY00224, NT00789	EU029800, EU071690, EU643615	(36), CK Hurley^b^
DRB1*15:25	DRB1*1525	—	—	—	BY00225	EU071689	(36)
DRB1*15:26	DRB1*1526	—	—	—	BY00229	EU071685	(36)
DRB1*15:27	DRB1*1527	—	—	—	BY00256, I982583	EU146150, EU375477	(36), G Wohlwend^b^
DRB1*15:28	DRB1*1528	—	—	—	JMDP01K031	AB436801	K Tadokoro
DRB1*15:29	DRB1*1529	—	—	—	JMDP01K032	AB436798	K Tadokoro
DRB1*15:30	DRB1*1530	—	—	—	JMDP01K033	AB436799	K Tadokoro
DRB1*15:31	DRB1*1531	—	—	—	BY00328	EU716065	CK Hurley
DRB1*15:32	DRB1*1532	—	—	—	1-6117	FJ668016	(406)
DRB1*15:33	DRB1*1533	—	—	—	DEMBG-5010128	FJ827122	(407)
DRB1*15:34	DRB1*1534	—	—	—	JMDP36K046	AB512505	K Tadokoro
DRB1*15:35	DRB1*1535	—	—	—	HN-93955-5, HN-23198-2	FJ358156, FJ549414, FJ766002	Histogenetics
DRB1*15:36	DRB1*1536	—	—	—	HN-20633-3, HN-06776-0, HN-93465-5	FJ358158, FJ358167, FJ358170	Histogenetics
DRB1*15:37	DRB1*1537	—	—	—	HN-13676-1, HN-53517-7, HN-81929-2	FJ358159, FJ502335, FJ875599	Histogenetics
DRB1*15:38	DRB1*1538	—	—	—	HN-4746371, HN-7435594, HN-0448998	FJ358160, FJ358165, FJ358166	Histogenetics
DRB1*15:39	DRB1*1539	—	—	—	HN-0663604	FJ358168	Histogenetics
DRB1*15:40	DRB1*1540	—	—	—	HN-86092-8	FJ358175	Histogenetics
DRB1*15:41	DRB1*1541	—	—	—	HN-89246-5	FJ358176	Histogenetics
DRB1*15:42	DRB1*1543	—	—	—	HN-39373-2	FJ766007	Histogenetics
DRB1*15:43	DRB1*1543	—	—	—	HN-44736290	FJ858913	Histogenetics
DRB1*16:01:01	DRB1*160101	DR16(2)	Dw21	DR2B Dw21	AZH, MN-2, FJO, W692D, W738D, 20A3	M16959, M30179, M28583, U56640, AF029291	
DRB1*16:01:02	DRB1*160102	DR16(2)	Dw21	—	GN00150	U59686	
DRB1*16:02:01	DRB1*160201	DR16(2)	Dw22	DR2B Dw22	REM (RML), 20A4	M20504, AF029292	
DRB1*16:02:02	DRB1*160202	DR16(2)	Dw22	DRB1*16MADANG	MAD009	U38520	
DRB1*16:03	DRB1*1603	DR2	—	—	JWR	L02545	
DRB1*16:04	DRB1*1604	DR16(2)	—	DRB1*16x8	BONA, FORE	L14852, AM110006	R Blasczyk^b^
DRB1*16:05:01	DRB1*160501	DR16(2)^c^	—	16PRET	EH.B., PRET4149	X74343, X75444	
DRB1*16:05:02	DRB1*160502	DR16(2)^c^	—	—	NT00502	AY428805	
DRB1*16:07	DRB1*1607	—	—	DR2Mut	USH	U26659	
DRB1*16:08	DRB1*1608	—	—	DRB1*(Gi+Pi)	Gi, Pi	Z72424	
DRB1*16:09	DRB1*1609	DR16(2)	—	—	NJ7479, NT01110	AY912074, GQ373165	(408), CK Hurley^b^
DRB1*16:10	DRB1*1610	—	—	—	HZ8028, BY00227	DQ192647, EU071687	L Yan, CK Hurley^b^
DRB1*16:11	DRB1*1611	—	—	—	K9505074	DQ837166	(409)
DRB1*16:12	DRB1*1612	—	—	—	BY00219, BY00226	EU029801, EU071688	(36)
DRB1*16:13N	DRB1*1613N	Null	—	—	MDA-DR16N	EU078908	(410)
DRB1*16:14	DRB1*1614	—	—	—	NT01107	GQ373168	CK Hurley
DRB1*16:15	DRB1*1615	—	—	—	SCU-675, BY00552	GU014286, GU066763	(411), CK Hurley^b^
							
DRB2*01:01	DRB2*0101	—	—	—	AVL	M86691, M86694, M16274, M16275	
							
DRB3*01:01:02:01	DRB3*01010201	DR52	Dw24	DR3 III, DRw6a III dJ172K2, DRB3*01012, DRB3*010101	AVL, HHKB, DM28, DM29, CMCC, U-STH, PMR, HSF7, W461R, COX	X04055, X04058, AF152844, U66825, Z84814, AF000448, AL662842	(29)^b^
DRB3*01:01:02:02	DRB3*01010202	DR52	Dw24	—	GN00199, 23054638	AF092089, AF092176, AF199236	
DRB3*01:01:03	DRB3*010103	DR52	Dw24	DRB3*MOBD	MO, BD	X99771	
DRB3*01:01:04	DRB3*010104	DR52	—	DRB3*01BTT	TO02021	Y10553	
DRB3*01:01:05	DRB3*010105	DR52	—	—	B07-2819, B07-1737, BM-4028, H-05046	EU873151	(412)
DRB3*01:02	DRB3*0102	—	—	DRB3*N409	409/96-UKN	Y08063	
DRB3*01:03	DRB3*0103	—	—	DRB3*DF	DF	U94590	
DRB3*01:04	DRB3*0104	—	—	—	GN00139	AF026467	
DRB3*01:05	DRB3*0105	—	—	—	GN00234	AF081677	
DRB3*01:06	DRB3*0106	DR52	—	DRB3*01EGT	EG-OT	AJ242860	
DRB3*01:07	DRB3*0107	DR52	—	DRB3*01ABT	AB-OT	AJ242862	
DRB3*01:08	DRB3*0108	—	—	—	1507-33405	AF361865	
DRB3*01:09	DRB3*0109	—	—	—	GN00394	AY042679	
DRB3*01:10	DRB3*0110	DR52	—	DRB3*01MGT	CL06453	AJ315477	
DRB3*01:11	DRB3*0111	—	—	—	612650	AJ564210	
DRB3*01:12	DRB3*0112	—	—	—	B07-1953	EU873152	(412)
DRB3*01:13	DRB3*0113	—	—	—	B07-2896	EU873153	(412)
DRB3*01:14	DRB3*0114	—	—	—	A30210172	FN424162	(413)
DRB3*02:01	DRB3*0201	DR52	Dw25	DRw6b III	4/w6, DM24	M17380, V00522	
DRB3*02:02:01	DRB3*020201	DR52	Dw25	pDR5b.3	SWEIG, WT49, U-STH, QBL	X99690, AF152845, AL929581	(105)^b^
DRB3*02:02:02	DRB3*020202	DR52	—	DRB3*02CVT	CV-OT	AJ242861	
DRB3*02:02:03	DRB3*020203	DR52	—	DRB3*SSOM	SSOM	AF177216	
DRB3*02:02:04	DRB3*020204	DR52	—	—	GN00418	AY094138	
DRB3*02:02:05	DRB3*020205	DR52	—	—	9791	DQ309438	(414)
DRB3*02:03	DRB3*0203	DR52	—	DRB3.02p	POS	X86977	
DRB3*02:04	DRB3*0204	—	—	—	SCHT	X91639	
DRB3*02:05	DRB3*0205	—	—	DRB3*02-03v	GN068	U36826	
DRB3*02:06	DRB3*0206	—	—	DRB3*02MT	BV1661	X95760	
DRB3*02:07	DRB3*0207	DR52	—	DRB3 new	BML	Y10180	
DRB3*02:08	DRB3*0208	DR52	—	DRB3*02HMT	BV02755	AJ001255	
DRB3*02:09	DRB3*0209	DR52	—	DRB3*02New	p1454/bg287, Orietta Q.C.16/98	AF148518, AF132810	
DRB3*02:10	DRB3*0210	DR52	—	DRB3*02KM	SMS145263 Diakon, CTM-9991295, NMDP#0236-9013-4	AJ238155, AF192259, AB035378	
DRB3*02:11	DRB3*0211	DR52	—	DRB3*02NEW-A	CTM-9991121	AF192258	
DRB3*02:12	DRB3*0212	—	—	DRB3*JWOO	JWOO	AF208484	
DRB3*02:13	DRB3*0213	—	—	DRB3*HMAR	HMAR	AF208485	
DRB3*02:14	DRB3*0214	—	—	—	00F03, 00F10, 00F13	AJ290395	
DRB3*02:15	DRB3*0215	—	—	—	VTIS45001, VTIS45004	AF427138, AF427139	
DRB3*02:16	DRB3*0216	—	—	—	74356	AF455114	
DRB3*02:17	DRB3*0217	—	—	DRB3*VNGAZ	VNGAZ, emanuela, PB-MID 65347, FR-MID 65690, FR-MID 65691	AF461431, AY033875, AJ441058	
DRB3*02:18	DRB3*0218	—	—	DRB3*02New	8997607	AY291205	
DRB3*02:19	DRB3*0219	—	—	—	DKM777xxx	AY271986	
DRB3*02:20	DRB3*0220	—	—	—	cr01-3558, 63140	AY958608, FN556018	A Malagoli, JDH Anholts^b^
DRB3*02:21	DRB3*0221	—	—	—	120720	DQ311653	A Amoroso
DRB3*02:22	DRB3*0222	—	—	—	KHYRHib	AM413002	A Dormoy
DRB3*02:23	DRB3*0223	—	—	—	NSB971105	AM747470	(298)
DRB3*02:24	DRB3*0224	—	—	DRB3*02New	W153	FJ515276	E Rozemuller
DRB3*02:25	DRB3*0225	—	—	—	408330201	Fn424163	A Dormoy
DRB3*03:01:01	DRB3*030101	DR52	Dw26	—	WT46, CMCC, EMJ	AY138123	
DRB3*03:01:02	DRB3*030102	DR52	Dw26	DRB3*KL044	RP-KL044	AF242306	
DRB3*03:01:03	DRB3*030103	DR52	Dw26	DRB3New	THAI742	FJ515277	E Rozemuller
DRB3*03:02	DRB3*0302	DR52	—	DRB3*03KLT	SJ00198	Y13715	
DRB3*03:03	DRB3*0303	—	—	DRB3*03SM	RP-SM073, 48280, 48733	AF028012, FN556017	JDH Anholts^b^
							
DRB4*01:01:01:01	DRB4*01010101	DR53	—	—	MANN, LBF, DKB, BURKHARDT, KT3, PRIESS, FS, DM24, DM29, MMCC	M16942, M17385, M17388, M15071, K02775	
DRB4*01:02	DRB4*0102	—	—	DRB4*ICML	C.M.L., CML	L08621, D89879	
DRB4*01:03:01:01	DRB4*01030101	DR53	—	dJ93N13	MJ4, BOLETH, HSF7, G081	M15178, M20555, M19556, Z84477, AF361548	
DRB4*01:03:01:02N	DRB4*01030102N	Null	—	DRB4 null	DBB	D89918	
DRB4*01:03:02	DRB4*010302	DR53	—	DRB4W778R	W778R	AF048707	
DRB4*01:03:03	DRB4*010303	DR53	—	DRB4GL	MG-CV, FOA2362, G081, MUDGH	AJ242833, AJ297503, AF207709, AF361549, AJ252282	
DRB4*01:03:04	DRB4*010304	DR53	—	—	14242	AJ292564	
DRB4*01:04	DRB4*0104	—	—	DRB4*CR210	69-218, 76-394, TBC-4975	X92712, AB107960	
DRB4*01:05	DRB4*0105	DR53	—	DRB4New	17345	Y09313	
DRB4*01:06	DRB4*0106	—	—	—	MKOST	AF450316, AF450317	
DRB4*01:07	DRB4*0107	—	—	—	X0002601, X0002652, X0002630	AY394720	
DRB4*01:08	DRB4*0108	—	—	—	Tokushima	AB510591	E Lee
DRB4*02:01N	DRB4*0201N	Null	—	DRB4*VI	GN016	U50061, U70543, U70544	
DRB4*03:01N	DRB4*0301N	Null	—	DRB4*v2	GN017	U70542	
							
DRB5*01:01:01	DRB5*010101	DR51	Dw2	DR2A Dw2	PGF, ROF-NL, MHHZ-00020722	M17377, M16954, M20429, AM159646	R Blasczyk^b^
DRB5*01:01:02	DRB5*010102	DR51	Dw2	—	GN00152	U66721	
DRB5*01:02	DRB5*0102	DR51	Dw12	DR2A Dw12	BGE, DHO	M16955, M30182, M16086	
DRB5*01:03	DRB5*0103	—	—	DRB5.Oli	IND-24, IND-59, NT0002	X86978, AF122887	
DRB5*01:04	DRB5*0104	—	—	DRB5*0101V	GN045	U31770	
DRB5*01:05	DRB5*0105	—	—	—	CP5570	X87210	
DRB5*01:06	DRB5*0106	—	—	DRB5*New	ZL4062	Z83201	
DRB5*01:07	DRB5*0107	DR51	—	DRB5*01CBT	WI01846	Y09342	
DRB5*01:08N	DRB5*0108N	Null	—	—	ES	Y10318, Y17819	
DRB5*01:09	DRB5*0109	—	—	DRB5*01ART	BV08663	Y13727	
DRB5*01:10N	DRB5*0110N	Null	—	DRB5*0102Null, DRB5*CB848	JAS, CB848	AF097680, AF314541	
DRB5*01:11	DRB5*0111	—	—	DRB5*DELIJ	DELIJ	AY141137	
DRB5*01:12	DRB5*0112	—	—	—	Barpay	AJ427352	
DRB5*01:13	DRB5*0113	—	—	—	117631, 44584, 119156	AY457037	
DRB5*01:14	DRB5*0114	—	—	—	LUMC-DRB5n61	FN430425	JDH Anholts
DRB5*02:02	DRB5*0202	DR51	Dw21, Dw22	DR2A Dw21, DR2A Dw22	REM (RML), FJO, MN-2, AZH	M16956, M30181, M20503, M15992, M32578, X99939	
DRB5*02:03	DRB5*0203	—	—	DRB5*HK	HK55	M91001	
DRB5*02:04	DRB5*0204	—	—	—	GN00151	U59685	
DRB5*02:05	DRB5*0205	—	—	DRB5*02 variant	TT030822	AJ271159	
							
DRB6*01:01	DRB6*0101	—	—	DRBσ*0101, DRBX11	BAC, BRO-2, HOM-2, KAS116, MZ070782, HON, SAS6211	X53357, M83892	
DRB6*02:01	DRB6*0201	—	—	DRBX21, DRBVI	PGF, D0208915, CGG, BA, E4181324	M77284-7, X53358, M83893	
DRB6*02:02	DRB6*0202	—	—	DRBσ*0201, DRBX22, DRB6III	RML, KAS011	M83204, M83894	
							
DRB7*01:01:01	DRB7*010101	—	—	DRBψ1	BOLETH, BH13	K02772-4, L31617	
DRB7*01:01:02	DRB7*010102	—	—	—	PITOUT	L31618	
							
DRB8*01:01	DRB8*0101	—	—	DRBψ2	BOLETH	M20556, M20557	
							
DRB9*01:01	DRB9*0101	—	—	M4.2 β exon	MOU	M15563	

a Allele names given in bold type have been assigned since the 2004 Nomenclature report.

b This reference is to a confirmatory sequence.

c HLA specificity provided from the HLA dictionary (22–26).

**Table 14:** List of allele names that have been deleted

Old name now deleted	New name	Reason for change
A*0105N	A*01:04N	Sequence named in error
A*020116	A*02:134	Sequence named in error
A*020120	A*02:01:18	Sequence named in error
A*0223	A*02:22:01	Sequence named in error
A*0298	A*02:96	Sequence named in error
A*1128	A*11:15:02	Sequence renamed
A*2401	—	Sequence shown to be in error
A*2412	A*24:08	Sequence named in error
A*2416	A*31:08	Sequence renamed
A*2465	A*24:13:02	Sequence renamed
A*3005	A*30:04	Sequence shown to be in error and renamed
A*3021	A*30:11:02	Sequence renamed
A*31011	A*31:01:02	Sequence shown to be in error and renamed
A*3302	A*33:03	Sequence shown to be in error and renamed
B*0701	—	Sequence shown to be in error
B*1305	B*13:04	Sequence submitted with errors
B*1324	B*13:22:02	Sequence renamed
B*150105	B*15:120	Sequence shown to be in error and renamed
B*1522	B*35:43	Sequence renamed
B*1541	B*15:39	Sequence named in error
B*1559	B*35:44	Sequence renamed
B*9530	B*15:27:02	Sequence renamed
B*1816	B*18:14	Sequence named in error
B*27051	B*27:05:02	Sequence shown to be in error and renamed
B*2722	B*27:06	Sequence named in error
B*3573	B*35:08:03	Sequence renamed
B*39012	B*39:01:01	Sequence shown to be in error and renamed
B*3921	B*39:24	Sequence submitted with errors
B*4017	B*40:16	Sequence named in error
B*4041	B*40:40	Sequence named in error
B*4203	B*42:02	Name never officially assigned
B*4401	B*44:02	Sequence shown to be in error and renamed
B*5003	B*50:02	Sequence named in error
B*5125	B*51:22	Sequence named in error
B*5147	B*51:09:02	Sequence renamed
B*5506	B*55:04	Sequence submitted with errors and renamed
B*5803	—	Name never officially assigned
B*7901	B*15:18	Sequence renamed
Cw*0101	Cw*01:02	Sequence shown to be in error and renamed
Cw*0201	Cw*02:02:02	Sequence shown to be in error and renamed
Cw*020204	Cw*02:10	Sequence named in error
Cw*021603	Cw*02:16:02	Sequence named in error
Cw*0301	Cw*03:04	Sequence shown to be in error and renamed
Cw*0402	Cw*04:01:01	Sequence named in error
Cw*0421	Cw*04:51:02	Sequence renamed
Cw*0422	Cw*04:21	Sequence named in error
Cw*0502	Cw*05:09	Sequence named in error
Cw*0601	Cw*06:02	Sequence shown to be in error and renamed
Cw*060202	Cw*06:17	Sequence extended and renamed
Cw*0734	Cw*07:27:02	Sequence renamed
Cw*1101	—	Sequencing artefact
Cw*1201	Cw*12:02:02	Sequence shown to be in error and renamed
Cw*1301	—	Sequence shown to be in error
Cw*1401	Cw*14:02	Sequence shown to be in error and renamed
Cw*1501	Cw*15:02	Sequence shown to be in error and renamed
Cw*1514	Cw*15:10:02	Sequence renamed
Cw*1603	Cw*14:03	Sequence shown to be in error and renamed
Cw*16042	Cw*16:04:01	Sequence named in error
Cw*1605	Cw*16:04:01	Sequence named in error
E*0102	E*01:01	Sequence named in error
G*010120	G*01:04:04	Sequence renamed
DRB1*0702	DRB1*07:01	Sequence shown to be in error and renamed
DRB1*08031	DRB1*08:03:02	Sequence shown to be in error and renamed
DRB1*09011	DRB1*09:01:02	Sequence shown to be in error and renamed
DRB1*1171	DRB1*11:02:01	Sequence named in error
DRB1*12031	DRB1*12:01	Sequence shown to be in error and renamed
DRB1*1466	DRB1*14:32:02	Sequence renamed
DRB1*1606	DRB1*16:05	Sequence shown to be in error and renamed
DRB3*010101	DRB3*01:01:02:01	Sequence named in error
DRB4*0101102N	DRB4*01:03:01:02N	Sequence named in error
DRB5*0201	DRB5*02:02	Sequence shown to be in error and renamed
DQA1*03012	DQA1*03:02	Sequence shown to be in error and renamed
DQA1*05013	DQA1*05:05	Additional coding polymorphism detected. Sequence renamed
DQB1*03031	DQB1*03:03:02	Sequence shown to be in error and renamed
DPA1*0101	DPA1*01:03	Sequence shown to be in error and renamed
DPA1*0102	DPA1*01:03	Sequence shown to be in error and renamed
DPB1*02011	DPB1*02:01:02	Sequence shown to be in error and renamed
DPB1*0701	—	Name never assigned
DPB1*1201	—	Name never assigned
DPB1*4201	DPB1*31:01	Sequence shown to be in error and renamed
DPB1*4301	DPB1*28:01	Sequence shown to be in error and renamed
MICA*003	—	Name never assigned
MICA*021	MICA*012:03	Sequence renamed
MICB*017	MICB*005:05	Sequence renamed

**Table 13:** Numbers of alleles with official names at each locus by 31st December 2009

Locus	Number of alleles
HLA-A	965
HLA-B	1543
HLA-C	626
HLA-E	9
HLA-F	21
HLA-G	46
HLA-DRA	3
HLA-DRB1	762
HLA-DRB2	1
HLA-DRB3	52
HLA-DRB4	14
HLA-DRB5	19
HLA-DRB6	3
HLA-DRB7	2
HLA-DRB8	1
HLA-DRB9	1
HLA-DQA1	35
HLA-DQB1	107
HLA-DPA1	28
HLA-DPB1	138
HLA-DOA	12
HLA-DOB	9
HLA-DMA	4
HLA-DMB	7
TAP1	7
TAP2	4
MICA	68
MICB	30

**Table 8:** Designations of HLA-DPA1 and -DPB1 alleles

HLA allele^a^	Pre 2010 designation	Associated HLA–DP specificities	Previous equivalents	Individual or cell line from which the sequence was derived	Accession number	References or submitting author(s)
DPA1*01:03:01	DPA1*010301	—	DPw4α1	BOLETH, 3.1.0, LG2, PRIESS, LB	X03100, X82390, X82392, X82389	
DPA1*01:03:02	DPA1*010302	—	DPA1	933-302-2	AF074848	
DPA1*01:03:03	DPA1*010303	—	—	KOR10099	AY618552	
DPA1*01:03:04	DPA1*010304	—	—	BM4129, BM4066	DQ274060	(429)
DPA1*01:04	DPA1*0104	—	01New	SK	X78198, X81348, X82391	
DPA1*01:05	DPA1*0105	—	DPA1*RK	DNA-RK	X96984	
DPA1*01:06:01	DPA1*010601	—	DPA1*Indian-024	I024	U87556	
DPA1*01:06:02	DPA1*010602	—	—	ML2560	EU729350	(430)
DPA1*01:07	DPA1*0107	—	DPA1*0103New	#913	AF076284	
DPA1*01:08	DPA1*0108	—	—	936-563-6	AF346471	
DPA1*01:09	DPA1*0109	—	—	9214065	AY650051	TM Williams
DPA1*01:10	DPA1*0110	—	—	BM3950	DQ274061	(429)
DPA1*02:01:01	DPA1*020101	—	DPA2, pDAα13B	DAUDI, AKIBA	X82394, X82393, X78199	
DPA1*02:01:02	DPA1*020102	—	DPA1*TF	A371, L67, LB0410278	L31624, X83610	
DPA1*02:01:03	DPA1*020103	—	DPA1-CAM024, DPA1*Cameroon2	CAM024, CAM241, #63	U94838, AF015295, AF076285	
DPA1*02:01:04	DPA1*020104	—	DPA1	533-2929, 922-485-8	AF074847	
DPA1*02:01:05	DPA1*020105	—	DPA1*PERR	CC109	AF098794	
DPA1*02:01:06	DPA1*020106	—	—	A.L.	AF165160	
DPA1*02:01:07	DPA1*020107	—	—	2840	GQ374477	M Luo
DPA1*02:02:01	DPA1*020201	—	2.21	CB6B	M83906, L11642, X79475, X80482, X79479	
DPA1*02:02:02	DPA1*020202	—	2.22	LKT3, KT17, WI-L2 NS, CT46, EsSm, GIWh	M83907, L11641, X79476, X80484, X79480	
DPA1*02:02:03	DPA1*020203	—	DPA1*0202New	#904	AF092049	
DPA1*02:03	DPA1*0203	—	DPA1*TC48	TC48	Z48473	
DPA1*02:04	DPA1*0204	—	—	DP56	EU304462	(431)
DPA1*03:01	DPA1*0301	—	3.1	AMAI	M83908, X79477, X81347, X79481	
DPA1*03:02	DPA1*0302	—	DPA1*Cameroon	CAM48, CAM59, CAM66, CAM100, CAM151	AF013767	
DPA1*03:03	DPA1*0303	—	—	KOR10120	AY618553	
DPA1*04:01	DPA1*0401	—	4.1	T7526	M83909, L11643, X79478, X80483, X78200	
						
DPB1*01:01:01	DPB1*010101	DPw1	DPB1, DPw1b	LUY, RSH, P0077, FB11	M83129, M83664, M62338, X72070, AY804143	
DPB1*01:01:02	DPB1*010102	DPw1	DPB1*WA6	LINE 101, AH1457, IBW9	L19220, L27662, AY804133	
DPB1*01:01:03	DPB1*010103	DPw1	—	SJ21504	AJ810847	
DPB1*02:01:02	DPB1*020102	DPw2	DPB2.1	45.1, WJR076, LB, JY, BSM, Daudi, FH4	M62328, X03067, X99689, AY804134	
DPB1*02:01:03	DPB1*020103	DPw2	DPB2.1	CJ	X94078	
DPB1*02:01:04	DPB1*020104	DPw2	—	CQ930-SEQ1643	AF326565	
DPB1*02:01:05	DPB1*020105	DPw2	—	27D	AF462072	
DPB1*02:01:06	DPB1*020106	DPw2	—	UCLA-344	AF517128	
DPB1*02:01:07	DPB1*020107	DPw2	—	SHNA4805AN	FM211033	AM Little
DPB1*02:02	DPB1*0202	DPw2	DPB2.2	QBL, DUCAF, 99101422, HM-K	M62329, X72071, AF492642, AY656678, AY831404	
DPB1*03:01:01	DPB1*030101	DPw3	DPB3	SLE, PRIESS, ETH9-0226	M62334, X02964, X03023, X78044	
DPB1*03:01:02	DPB1*030102	DPw3	DPB1*03var	POHS-161	AF234538	
DPB1*04:01:01	DPB1*040101	DPw4	DPB4.1, DPw4a	HHKB, BOLETH, PRIESS, LC11, KAS011, HOM-2, MGAR, PITOUT, WIN	M62326, M23675, K01615, M23906-8, L29174, X03022, X030025-8, X02228, X72072, AY804136	
DPB1*04:01:02	DPB1*040102	DPw4	—	K80940	AJ563604	
DPB1*04:02	DPB1*0402	DPw4	DPB4.2, DPw4b	APD, BURKHARDT, FH1	M62327, M21886, AY831402	
DPB1*05:01:01	DPB1*050101	DPw5	DPB5	HAS, LKT3, 99101467, HM-K, YH-K, JRPAY	M62333, X72073, AF492638, AY656679, AY804138	
DPB1*05:01:02	DPB1*050102	DPw5	—	166240	FM992365	K Witter
DPB1*06:01	DPB1*0601	DPw6	DPB6	JMOS, FB11	M62335, X72074	
DPB1*08:01	DPB1*0801	—	DPB8	PIAZ	M62331	
DPB1*09:01	DPB1*0901	—	DPB9, DP’Cp63’	TOKUNAGA, 99100402, JRPAY, RA54	M62341, X72075, AF492637, AY804139, DQ148298, DQ148299, DQ148300, DQ148301	R Rani^b^
DPB1*10:01	DPB1*1001	—	DPB10	BM21, SAVC, 99101332, 194229	M85223, M62342, X72076, AF492640, FN594947	K Witter^b^
DPB1*11:01:01	DPB1*110101	—	DPB11	CRK, AVE G, H348, 171880	M62336, X78046, AM887530, FN598969	C Vilches^b^, K Witter^b^
DPB1*11:01:02	DPB1*110102	—	—	AH696	L23399	
DPB1*13:01	DPB1*1301	—	DPB13	NB, KAS116, YH-K, WIN	M62337, X72077, AY656680, AY831403	
DPB1*14:01	DPB1*1401	—	DPB14	8268, KAS011, FH4	M31778, M62343, X72078, AY804140	
DPB1*15:01	DPB1*1501	—	DPB15	PLH, 99100835	M31779, M62339, X72079, AF492636, AY804141	
DPB1*16:01	DPB1*1601	—	DPB16	JRA, WT46, 99101659, 168621, 143058, 197119	M31780, M62332, X72080, AF492641, FM992366, FM992080, FN552548	K Witter^b^
DPB1*17:01	DPB1*1701	—	DPB17	JRAB, LBUF, 99101046, 169622, UCLA0584	M31781, M62344, X72082, AF492643, FM882212, FM882212	K Witter^b^
DPB1*18:01	DPB1*1801	—	DPB18	JCA, 169716	M62340, FM991729	K Witter^b^
DPB1*19:01	DPB1*1901	—	DPB19	CB6B, 99101467	M62330, X72081, AF492639, AY804142	
DPB1*20:01:01	DPB1*200101	—	Oos, DPB-JA	OOS, ARENT, BEL8-CC, 191833	M58608, M63508, FN256255	K Witter^b^
DPB1*20:01:02	DPB1*200102	—	DPB1*BRI6	NT	M97685	
DPB1*21:01	DPB1*2101	—	DPB-GM, DPB30, NewD	GM, PEI52, PEI74, C1, T7527	M77659, M83915, M84621, M80300	
DPB1*22:01	DPB1*2201	—	DPB1*AB1, NewH	HV152, HV385, SAS60103, SAS60106	M77674, M83919	
DPB1*23:01	DPB1*2301	—	DPB32, NewB	D0208915, UK3082, UK5496, PT35, IT22, I132, 151979	M83913, M84014, FM956650	K Witter^b^
DPB1*24:01	DPB1*2401	—	DPB33, NewC	UK7430	M83914	
DPB1*25:01	DPB1*2501	—	DPB34, NewE	PEI46	M83916	
DPB1*26:01:01	DPB1*260101	—	DPB31, WA2	LINE70	M86229	
DPB1*26:01:02	DPB1*260102	—	DPB1*WA8	4-BEN NO2, 193322	L24387, FN252853	K Witter^b^
DPB1*27:01	DPB1*2701	—	DPB23, WA3	LINE92, H033	M84619, M86230	
DPB1*28:01	DPB1*2801	—	DPB21, JAVA2	I57, I147, JOG1489, 166240	M84617, L00599, FM992364	K Witter^b^
DPB1*29:01	DPB1*2901	—	DPB27, NewG	RBLB66, NG105, NG113, PNG112, PNG177, SCZ244	M84625, M83918, L01467	
DPB1*30:01	DPB1*3001	—	DPB28	AH1377, EB5, ETH-0245, 170324	M84620, X78045, FN594948	K Witter^b^
DPB1*31:01	DPB1*3101	—	DPB22, NewF, JAVA1	I68, I147, I6, PEI03, JOG1427, JOG1471	M84618, M83917, L00598	
DPB1*32:01	DPB1*3201	—	DPB24, NewI	NG78, PNG167	M84622, M85222	
DPB1*33:01	DPB1*3301	—	DPB25	HO23	M84623	
DPB1*34:01	DPB1*3401	—	DPB26	HO26, DH67	M84624	
DPB1*35:01:01	DPB1*350101	—	DPB29	AH1450, AH521	M84626	
DPB1*35:01:02	DPB1*350102	—	—	193318	FN256252	K Witter
DPB1*36:01	DPB1*3601	—	New A, SSK2	SASBE41, THM1, KT	M83912, D10479, D10882	
DPB1*37:01	DPB1*3701	—	DPB1*WA4	LINE41	M87046	
DPB1*38:01	DPB1*3801	—	SSK1	THKK	D10478	
DPB1*39:01	DPB1*3901	—	DPB1*BRI4	EM, ETH-0203	M97686, X78043	
DPB1*40:01	DPB1*4001	—	DPB1*BRI5, WA5	5D, LINE103, LINE105, LINE116, LINE117, LINE119, EB39, HO62	M97684, L19219, L23400	
DPB1*41:01:01	DPB1*410101	—	DPB2.3	HT	D13174	
DPB1*41:01:02	DPB1*410102	—	—	TZ-V603	EU153252	M Lin
DPB1*44:01	DPB1*4401	—	STCZ	SCZ259, SCZ244	L01466	
DPB1*45:01	DPB1*4501	—	DPB1*NM	C212, 193320	L09236, FN256251	K Witter^b^
DPB1*46:01	DPB1*4601	—	DPB1*NIB	V.E.C., R130	L07768, L31817	
DPB1*47:01	DPB1*4701	—	DPB1*02KY, *SUT	SAJ008, SAJ119, SUT	D14344, D10834	
DPB1*48:01	DPB1*4801	—	—	SE107	L17314	
DPB1*49:01	DPB1*4901	—	—	HO21	L17313	
DPB1*50:01	DPB1*5001	—	—	DIEDE	L17311	
DPB1*51:01	DPB1*5101	—	DPB1*WA7, *EA1, *JYO	C2#3, 15-BEN, NMDP#00800-2553-8, JYO	L17310, L19219, L27073, D28809	
DPB1*52:01	DPB1*5201	—	—	HO82	L22076	
DPB1*53:01	DPB1*5301	—	—	EB26	L22077	
DPB1*54:01	DPB1*5401	—	DPB1 New2	ETH-0222	X78042	
DPB1*55:01	DPB1*5501	—	DPB1 New3, DPBGUY	ETH-0271, J.M., 176633	X78041, X80331, FN256253	K Witter^b^
DPB1*56:01	DPB1*5601	—	DPB1-R90	R90	L31816	
DPB1*57:01	DPB1*5701	—	DPBMYT4220	H.R.	X80752	
DPB1*58:01	DPB1*5801	—	DPB1newAW	HAM006	X82123, X85966	
DPB1*59:01	DPB1*5901	—	—	GA Au, HBO1242, HBO1243, HBO1244, 0000-5922-0	Z47806, U29534, U59422	
DPB1*60:01	DPB1*6001	—	—	JN, BPN	U22313	
DPB1*61:01N	DPB1*6101N	Null	—	ZN, Nel., Nan	U22312, AJ002530	
DPB1*62:01	DPB1*6201	—	—	LE, CT	U22311	
DPB1*63:01	DPB1*6301	—	DPB1*IsOr	IsOr	U34033	
DPB1*64:01N	DPB1*6401N	Null	DPB1*IsAr	IsAr	U34032	
DPB1*65:01	DPB1*6501	—	—	E.L.	X91886	
DPB1*66:01	DPB1*6601	—	DPB1*BR	DNA-128	X96986	
DPB1*67:01	DPB1*6701	—	DPB1*TF	DNA-TF	X96985	
DPB1*68:01	DPB1*6801	—	DPB1*BAC	BAC1283, 902-258-3	Z70731, U59440	
DPB1*69:01	DPB1*6901	—	—	SBD3497	X97406	
DPB1*70:01	DPB1*7001	—	—	900-132-2	U59441	
DPB1*71:01	DPB1*7101	—	—	905-967-6, I045	U59438	
DPB1*72:01	DPB1*7201	—	—	0014-3022-2	U59439	
DPB1*73:01	DPB1*7301	—	—	0076-0684-1	U59437	
DPB1*74:01	DPB1*7401	—	DPB1-512ld	512ld	U94839	
DPB1*75:01	DPB1*7501	—	0402-GA	U73	Y09327	
DPB1*76:01	DPB1*7601	—	DPB1*14new	19835	Z92523	
DPB1*77:01	DPB1*7701	—	DPBnewBR	U.R.	Y14230	
DPB1*78:01	DPB1*7801	—	DPBNew	M541	Y13900	
DPB1*79:01	DPB1*7901	—	DPB1New	1197	Y16095	
DPB1*80:01	DPB1*8001	—	DPB1	18055285, CC	AF074845, FM210785	AM Little^b^
DPB1*81:01	DPB1*8101	—	DPB1*dre	009340662, dre	AF074846, AJ245640	
DPB1*82:01	DPB1*8201	—	DPB1*04New	19045	Y18498	
DPB1*83:01	DPB1*8301	—	—	GM-CV	AJ238005	
DPB1*84:01	DPB1*8401	—	DPB1*PERR	CC109	AF077015	
DPB1*85:01	DPB1*8501	—	DPB1*27New	MGD, UCLA212, 173643	AF184168, AF211979, FN256254	K Witter^b^
DPB1*86:01	DPB1*8601	—	DP New	605861, 606165	AJ271373	
DPB1*87:01	DPB1*8701	—	DPB1*2001new	#014738363	AF288354	
DPB1*88:01	DPB1*8801	—	DPB1*3701new	#009519430, 193326	AF288355, FN256250	K Witter^b^
DPB1*89:01	DPB1*8901	—	DPB1*MO	MOP	AJ297820	
DPB1*90:01	DPB1*9001	—	DPBnew	608050	AJ292074	
DPB1*91:01	DPB1*9101	—	DP14New	VTIS20927	AY029777	
DPB1*92:01	DPB1*9201	—	—	VTIA71787	AF489518	
DPB1*93:01	DPB1*9301	—	—	Lahu061	AF536241	
DPB1*94:01	DPB1*9401	—	—	MCH4376	AF549409	
DPB1*95:01	DPB1*9501	—	—	MCH4904	AF549410	
DPB1*96:01	DPB1*9601	—	—	UCLA356	AJ514871	
DPB1*97:01	DPB1*9701	—	—	627-S3262	AY033075	
DPB1*98:01	DPB1*9801	—	—	19503	AJ563603	
DPB1*99:01	DPB1*9901	—	—	000342683	AY425707	
DPB1*100:01	DPB1*0102	—	—	D-5189	AY374100	
DPB1*101:01	DPB1*0203	—	—	04-9525505	AY572830	
DPB1*102:01	DPB1*0302	—	—	03-82	AY618897	
DPB1*103:01	DPB1*0403	—	—	CBD1	AY823995	
DPB1*104:01	DPB1*0502	—	—	FH1	AY804135	
DPB1*105:01	DPB1*0602	—	—	RSH	AY804137	
DPB1*106:01	DPB1*0802	—	—	DAUDI	AY804132	
DPB1*107:01	DPB1*0902	—	—	T7526	AY831401	
DPB1*108:01	DPB1*1002	—	—	2004/0716	AY855160	A Chiesa
DPB1*109:01	DPB1*1102	—	—	2004/1097	AY855161	L Garbarino
DPB1*110:01	DPB1*1302	—	—	4023005	DQ089022	(432)
DPB1*111:01	DPB1*1402	—	—	ANAN324819AN	AM039828	AM Little
DPB1*112:01	DPB1*1502	—	—	DACO41672AN	AM158320	AM Little
DPB1*113:01	DPB1*1602	—	—	TBCS197	AB247517	M Satake
DPB1*114:01	DPB1*1702	—	—	GZ7272	DQ206450	(433)
DPB1*115:01	DPB1*1802	—	—	CDC022206	DQ386161	S Cordovado
DPB1*116:01	DPB1*1902	—	—	BS169	DQ485789	(434)
DPB1*117:01	DPB1*2002	—	—	MITH93933AN	AM408787	AM Little
DPB1*118:01	DPB1*2102	—	—	JERA34315AN	AM698036	AM Little
DPB1*119:01	DPB1*2202	—	—	JUST1407AN	FM211032	AM Little
DPB1*120:01N	DPB1*2302N	Null	—	LECL647AN	FM211034	AM Little
DPB1*121:01	DPB1*2402	—	DPB1*04new	191849	FM883600	(435)
DPB1*122:01	DPB1*2502	—	—	—	BY00464	CK Hurley
DPB1*123:01	DPB1*2602	—	—	194863	FN393829	K Witter
DPB1*124:01	DPB1*2702	—	—	196660, 197150, 197151	FN555432	K Witter
DPB1*125:01	DPB1*2802	—	—	UMKA9360A1	FN555527	U Kanga

a Allele names given in bold type have been assigned since the 2004 Nomenclature report.

b This reference is to a confirmatory sequence.

**Table 9:** Designations of HLA-DOA and -DOB alleles

HLA allele^a^	Pre 2010 designation	Previous equivalents	Individual or cell line from which the sequence was derived	Accession number	References or submitting author(s)
DOA*01:01:01	DOA*010101	DZα, DNA1.2a	JG, MANN, DBB	X02882, Z81310, AB005994	
DOA*01:01:02:01	DOA*01010201	pII-α-6, DNA1.1b	SPL, TOK	M26039, AB005992	
DOA*01:01:02:02	DOA*01010202	PGDZ1, DNA1.1a	PGF, SA	M31525, AB005991	
DOA*01:01:02:03	DOA*01010203	DNA1.1c	SPO101	AB005993	
DOA*01:01:03	DOA*010103	DNA1.2b	DKB	AB005995	
DOA*01:01:04:01	DOA*01010401	DNA1.3a	U937	AB005996	
DOA*01:01:04:02	DOA*01010402	DNA1.3b	U937	AB005997	
DOA*01:01:05	DOA*010105	DNA1.4	COX	AB005998	
DOA*01:01:06	DOA*010106	—	GRB2012722	AY947479	(436)
DOA*01:02	DOA*0102	—	GRB2024703	AY947480	(436)
DOA*01:03	DOA*0103	—	GRB2022544	AY947481	(436)
DOA*01:04N	DOA*0104N	Null	GRB2010154	AY947482	(436)
					
DOB*01:01:01:01	DOB*01010101	DO, pII-b-9	45.1, SPL, SA, LCL721	X03066, M26040, AB035249	
DOB*01:01:01:02	DOB*01010102	—	WT100BIS, LCL721	AB035250	
DOB*01:01:02	DOB*010102	DOB1.6	SR117	AB035254	
DOB*01:01:03	DOB*010103	—	GRB1070098	AY645723	
DOB*01:02:01	DOB*010201	DOB	BOLETH	L29472	
DOB*01:02:02	DOB*010202	DOB1.3	AKIBA	AB035251	
DOB*01:03	DOB*0103	HA14	MANN	X87344	
DOB*01:04:01:01	DOB*01040101	DOB1.4	PEA	AB035252	
DOB*01:04:01:02	DOB*01040102	DOB1.5	SPO010	AB035253	

a Allele names given in bold type have been assigned since the 2004 Nomenclature report.

**Table 10:** Designations of HLA-DMA and -DMB alleles

HLA allele^a^	Pre 2010 designation	Previous equivalents	Individual or cell line from which the sequence was derived	Accession number	References or submitting author(s)
DMA*01:01	DMA*0101	RING6	JY, MANN	X62744	
DMA*01:02	DMA*0102	DMA-Ile 140	AZL	Z24753	
DMA*01:03	DMA*0103	DMA3.2	HOM-2	U04878	
DMA*01:04	DMA*0104	DMA3.4	BM21	U04877	
					
DMB*01:01	DMB*0101	RING7	JY, MANN	Z23139	
DMB*01:02	DMB*0102	DMB-Glu 143	YAR	Z24750	
DMB*01:03	DMB*0103	DMB-Thr 179	BM16	Z24751	
DMB*01:04	DMB*0104	DMB3.4	CEPH 23-01	U00700	
DMB*01:05	DMB*0105	HY595, DMB*KV1	HY595, H.S.K.	D32055, U16762	
DMB*01:06	DMB*0106	DMB*PERR	CC44	AF134890, AF072680	
DMB*01:07	DMB*0107	—	GRB1070061	AY645722	

a Allele names given in bold type have been assigned since the 2004 Nomenclature report.

**Table 11:** Designations of TAP alleles

TAP allele^a^	Pre 2010 designation	Previous equivalents	Individual or cell line from which the sequence was derived	Accession number	References or submitting author(s)
TAP1*01:01	TAP1*0101	RING4, PSF(Y3), TAP1A	U937, LCL721.45, HB00028, HB00032	X57522, X57521, L21204	
TAP1*01:02N	TAP1*0102N	TAP1*0101Null	KMW	AB012644, AB012645	
TAP1*02:01:01	TAP1*020101	TAP1B	CK	L21206	
TAP1*02:01:02	TAP1*020102	TAP1E	HEH	L21205	
TAP1*03:01	TAP1*0301	TAP1C	JT	L21208	
TAP1*04:01	TAP1*0401	TAP1D	HB00031	L21207	
TAP1*05:01	TAP1*0501	—	HOLTLN-096	AY523970	
					
TAP2*01:01	TAP2*0101	RING11A, TAP2A	CEM-CCRF	M84748	
TAP2*01:02	TAP2*0102	TAP2E	JY	Z22936	
TAP2*01:03	TAP2*0103	TAP2F	S-2	U07844	
TAP2*02:01	TAP2*0201	RING11B, TAP2B	DX3	Z22935	

a Allele names given in bold type have been assigned since the 2004 Nomenclature report.

**Table 12:** Designations of MICA and MICB alleles

MICA/MICB allele^a^	Pre 2010 designation	Previous equivalents	Individual or cell line from which the sequence was derived	Accession number	References or submitting author(s)
MICA*001	MICA*001	MICA001, PERB11.1-18.2, MICA-EIBA	IMR90, EJ32B, DUCAF, EVA, SP, QBL	L14848, U56940, L29406, U69965, AF085059, AF085060, AF085061, AF085062, AF336085, AF336086, AL845443	(105)^b^
MICA*002:01	MICA*00201	MICA002, MICA-BEBF	YAR, AMAI, WT49, TEM, JBUSH, 9-2,ZR75-1	U56941, AF085043, AF085044, AF085045, AF085046, AF336063, AF336083	
MICA*002:02	MICA*00202	MICA-BEE, MICA042	Individual1	AF011877, AF011878, AF011879	
MICA*002:03	MICA*00203	—	Allo4250	FJ790821	D Pidwell
MICA*004	MICA*004	MICA004, MICA-AJCD	MOU, BM15, PF97387, MANN, RSH, Individual2, JPKO	U56943, X92841, AF085031, AF085032, AF085033, AF085034, AB202108	(437)^b^
MICA*005	MICA*005	MICA005	U373	U56944	
MICA*006	MICA*006	MICA006, MICA-ADCD	KAS116	U56945, AF085023, AF085024, AF085025, AF085026, AF336065, AF336066	
MICA*007:01	MICA*00701	MICA007, MICA-CEEA	JESTHOM, BM92, WT24	U56946, AF085047, AF085048, AF085049, AF085050	
MICA*007:02	MICA*00702	MICA-CEB, MUC-22, MICA023	A34, B27-ci, SchS(child1)-MUC	AF011880, AF011881, AF011882, Y16805	
MICA*007:03	MICA*00703	—	U181	AJ580806	(438)
MICA*008:01	MICA*00801	MICA008, PERB11.1-44.1, PERB11.1-8.1, PERB11.1-60.3, PERB11.1-47.1, MICA-AAAC	SCHU, MGAR, SAVC, LB, JY, R90/7379, REE,GD, EMJ, PLH, DKB, LBF, WT8, APD, MADURA, SAM, COX	U56947, U69624, U69967, L29409, U69977, U69628, L29411, U69625, U69970, U69976, AF085015, AF085016, AF085017, AF085018, AF336067, AF336068, AY603357, AL669854	(29)^b^
MICA*008:02	MICA*00802	MICA-AAD, MICA-AN23, MUC-26, MICA026, MICA-silent B	Individual3, GUA-ND, BrI(f)-MUC, MLA-MUC, BrID(child1)-MUC, Thai-DCH019, 01083208, 01065930, 0183074	AF011883, AF011884, AF011885, AJ250499, AJ250500, Y16809, AF106650, AF106651, AF106652	
MICA*008:03	MICA*00803	MICA-silent C, MICA054	01083082	AF106653, AF106654, AF106655	
MICA*008:04	MICA*00804	—	PGF	AL645933	(29)
MICA*009:01	MICA*00901	MICA009, PERB11.1-52.1, MICA-ABCD	RML, AKIBA, HARA, BOB, C1R, JHAF, LUY, Individual2, E4181324, AKIBA	U56948, U69626, U69971, AF085019, AF085020, AF085021, AF085022, AF336069, AB103614	(437)^b^
MICA*009:02	MICA*00902	MICA-AFC, MICA-TAND, MICA020, MUC-20	MANIKA, TAA, AE(F)-MUC, AS(Child2)-MUC, DZA 97-19	AF011886, AF011887, AF011888, AF097419, AF079420, AF079421, AF079406, Y16803, AY029762, AY029763	
MICA*010	MICA*010	MICA010, PERB11.1-62.1, PERB11.1-46.1, MICA-DGAB, MUC-18	AMALA, BOLETH, T7526, BSM, KAS011, TAB089, EM(M)-MUC, EM(Child1)-MUC, EK-MUC, ES-MUC, T7526	U56949, U69629, U69974, L29408, U69969, AF085055, AF085056, AF085057, AF085058, Y16801, AF336071, AF336072	
MICA*011	MICA*011	MICA011, PERB11.1-65.1, MICA-BCGE	LWAGS, T47D	U56950, U69630, U69975, AF085035, AF085036, AF085037, AF085038, AF336073, AF336074	
MICA*012:01	MICA*01201	MICA012, PERB11.1-54.1	LKT3, HOKKAIDO, TA94	U56951, U69627, U69972, AF336081, AF336082	
MICA*012:02	MICA*01202	MICA-silent A, MICA053	01082123	AF106647, AF106648, AF106649	
MICA*012:03	MICA*01203	MUC-17, MICA021, MICA*021	AA-MUC, AM(child1)-MUC, AS(child2)-MUC	Y18110	
MICA*013	MICA*013	MICA013	PAR1	U56952	
MICA*014	MICA*014	MICA014	PAR2	U56953	
MICA*015	MICA*015	MICA015, MICA-39	OMW	U56954, AF136157, AF136158, AF136159, AF264738, AF264739, AF264740	
MICA*016	MICA*016	MICA016, PERB11.1-35.1, MICA-AGFB, MUC-19	J0528239, FPAF, Q85/8086, NR(M)-MUC, NR(Child1)-MUC, NM(Child2)-MUC, TISI	U56955, U69623, U69966, AF085027, AF085028, AF085029, AF085030, Y16802	
MICA*017	MICA*017	MICA-KMCE, MICA017, MUC-27, MICA-AN31	KSM, DBB, DEU, WJR076, DEM, FD(F)-MUC, FM(child1)-MUC, HF(M)-MUC, HS(child1)-MUC, HT(child2)-MUC, Thai-DCH013, Thai-DCH020, Thai-DCH024	AF079413, AF079414, AF079415, AF097403, AJ250803, Y16810, AF264735, AF264736, AF264737	
MICA*018:01	MICA*01801	MICA-EEBA, MICA-GKIT, MICA018, MUC-23, MICA-AN22	31227ABO, BM16, CBA, DO208915, SE(F)-MUC, KU(F)-MUC, KF(child1)-MUC, Thai-DCH036, DZA 97-8, DZA 97-18, DZA 97-20, BM16, DO208915, NIH_MGC_89	AF011874, AF011875, AF011876, AF093116, AF079425, AF079426, AF079427, AF097404, Y16806, AJ250805, AF336077, BC016929	(439)^b^
MICA*018:02	MICA*01802	—	U180	AJ580805	
MICA*019	MICA*019	MICA-AMW, MICA-AGAB, MICA-DPCA, MICA019, MICA-AN26	SSA, HSB27, OLL, WEWAK1, DPCA, CF996, DHIF, WT51, HANGZHOUA31	AB015600, AF011835, AF011836, AF011837, AF093113, AF079416, AF079417, AF079418, AF097405, AJ250804, AF336079, AF336080, EU267602	L Yan^b^
MICA*020	MICA*020	MICA-AN33	25/1506	AJ249394	
MICA*022	MICA*022	MICA-BGA, MUC-21, MICA022	Individual10, Thai-DCH021	AF011856, AF011857, AF011858, Y16804	
MICA*023	MICA*023	MICA-BEBC	WDV	AF085039, AF085040, AF085041, AF085042	
MICA*024	MICA*024	MICA-AAC, MUC-24, MICA024	BT594, Individual7, DZA 97-17	AF011832, AF011833, AF011834, Y16807	
MICA*025	MICA*025	MICA-DEB, MUC-25, MICA025	BT20, Thai-DCH032	AF011853, AF011854, AF011855, Y16808	
MICA*026	MICA*026	MICA-CEED	HOM-2	AF085051, AF085052, AF085053, AF085054	
MICA*027	MICA*027	MICA-AAAB, MICA-AN21	SWEIG007, HSB27	AF085011, AF085012, AF085013, AF085014, AJ250802	
MICA*028	MICA*028	MICA-AABC, MUC-29, MICA028	DKB, KUR-MUC	AF011829, AF011830, AF011831, AF093115, Y18111	
MICA*029	MICA*029	MUC-30, MICA-AN27, MICA029	DZA 97-08, MFO-ND	Y18112, AJ250503, AJ250504	
MICA*030	MICA*030	MICA-KWHT, MICA036	WKD	AF079422, AF079423, AF079424	
MICA*031	MICA*031	MICA-AIB, MICA037	MCF7	AF011838, AF011839, AF011840	
MICA*032	MICA*032	MICA-AKB, MICA038	CAR, NS2TA, NS2TA1, S2T2	AF011841, AF011842, AF011843	
MICA*033	MICA*033	MICA-ALAB, MICA039, MICA-AN24	WEWAK1	AF011844, AF011845, AF011846, AF093114, AJ250505	
MICA*034	MICA*034	MICA-BCC, MICA040	Individual18	AF011847, AF011848, AF011849	
MICA*035	MICA*035	MICA-BEA, MICA041	SK-BR3	AF011850, AF011851, AF011852	
MICA*036	MICA*036	MICA-BHB, MICA043	EHM	AF011859, AF011860, AF011861	
MICA*037	MICA*037	MICA-CEA, MICA044	AVE G, GRE G, LS40, LH, IHL, AD031, Individual10, B7Qui, 8TB	AF011862, AF011863, AF011864	
MICA*038	MICA*038	MICA-CEC, MICA045	Individual12, Individual14	AF011865, AF011866, AF011867	
MICA*039	MICA*039	MICA-CEF, MICA046	Individual13	AF011868, AF011869, AF011870	
MICA*040	MICA*040	MICA-CIB, MICA047	A34	AF011871, AF011872, AF011873	
MICA*041	MICA*041	MICA-AN25, MICA048, MICA-newA	M7, 01083098, 01083208, 01081318, 01065894	AJ271789, AF106632, AF106633, AF106634	
MICA*042	MICA*042	MICA-newB, MICA049	01065869	AF106635, AF106636, AF106637	
MICA*043	MICA*043	MICA-AN32, MICA050, MICA-newC	RB22, 01084383	AJ250990, AJ250991, AF106638, AF106639, AF106640	
MICA*044	MICA*044	MICA-newD, MICA051	01083114	AF106641, AF106642, AF106643	
MICA*045	MICA*045	MICA-AN30, MICA052, MICA-newE	DEW-ND, 01083268, 01065876	AJ250506, AJ250507, AF106644, AF106645, AF106646	
MICA*046	MICA*046	MICA-AN28	M7	AJ250501, AJ250502	
MICA*047	MICA*047	MICA-055D	COYA3408, KM	AJ295250, AJ295251, AF286732	
MICA*048	MICA*048	—	TA21	AF264741, AF264742, AF264743	
MICA*049	MICA*049	—	LUY	AF264744, AF264746, AF264747	
MICA*050	MICA*050	MICA-GR	SMGM-1.3C	AY095537	
MICA*051	MICA*051	—	G367	AJ563426	
MICA*052	MICA*052	—	D15	AY748458, AY748459, AY748460, AY748461	V Mas
MICA*053	MICA*053	MICA-U044	U044	AJ871399	(438)
MICA*054	MICA*054	—	IDAM73501AN	AM899996	(440)
MICA*055	MICA*055	—	P218	EU254723	(441)
MICA*056	MICA*056	—	MYUL82208AN	AM944063	(440)
MICA*057	MICA*057	MICA*CHAH	AH-WT/PF, AH-WT/PF2	AF411923, AF411924, AF411925	(442)
MICA*058	MICA*058	—	Allo3587	FJ790820	D Pidwell
MICA*059	MICA*059	—	XW	GQ351696	W Tian
					
MICB*001	MICB*001	MICB	HeLa, IMR90	X91625	
MICB*002:01:01	MICB*0020101	MICB, MICB002, MICB0103101, MICB0103102, MICB-002	BOLETH, DKB, OMW, RSH, TEM, VAVY, WT100BIS, OMW, BOLETH, H1905, E-117, E-5, KAS011, YAR, RSH, DO208915, MICBPat#3	U65416, U95729, AB003602, AB003612, AB003603, AB003613, AF021222	
MICB*002:01:02	MICB*0020102	—	MCF	CR753864	S Beck
MICB*003	MICB*003	PERB11.2-IMX, MICB003, MICB0105	HSB2G6, DBB, TISI, DBB, DEM, DBB	U69978, U95730, AB003606, AB003616, CR753820	S Beck^b^
MICB*004:01:01	MICB*0040101	MICB007, MICB0104	BM14, DHIF, SA, SCHU, SAVC, H0724, PLH, PGF	U95734, AB003605, AB003615, AL663061	(29)^b^
MICB*004:01:02	MICB*0040102	—	APD	CR788288	S Beck
MICB*005:01:01	MICB*0050101	MICB005	FPAF, J0528239	U95732	
MICB*005:02:01	MICB*0050201	MICB004, MICB01021, MICB-003	BM15, BM92, BTB, EHM, EJ32B, HOM-2, JESTHOM, JVM, KAS116, LWAGS, PF97387, PITOUT, PMG075, TAB089, WT24, SPO010, H2380, H2418, H0936, H1607, BM21, IBW9, HO301, BM16, LKT3, KOSE, AMAI, CB6B, SPO010, LBUF, LUY, WT51, MANIKA, QBL, MICBPat#1	U95731, AB003599, AB003609, AF021223, BX001040	(105)^b^
MICB*005:02:02	MICB*0050202	—	PITOUT, MANN	EF051580, BX927320	J Martinez-Borra, S Beck
MICB*005:02:03	MICB*0050203	—	SSTO	BX005469	S Beck
MICB*005:02:04	MICB*0050204	—	WT51	EF051579	(443)
MICB*005:03	MICB*00503	MICB01022	H1500, E4181324, AKIBA	AB003600, AB003610, AB103613	(437)^b^
MICB*005:04	MICB*00504	MICB01023	RML	AB003601, AB003611	
MICB*005:05	MICB*00505	MICB-H0170101, MICB*017	PUM324-95	AJ606909, AJ606910, AJ606911	(444)
MICB*006	MICB*006	MICB006	SPACH	U95733	
MICB*007	MICB*007	MICB0103103	H0409	AB003604, AB003614	
MICB*008	MICB*008	MICB0106, MICB-001, MICB0106v	COX, MICBPat#4, WT49	AB003607, AB003617, AF021221, AJ251158, AJ251159, AL662866	(29)^b^
MICB*009N	MICB*009N	MICB0107N	H2520	AB003608, AB003618	
MICB*010	MICB*010	MICB-004	MICBPat#2	AF021224	
MICB*011	MICB*011	MICB-005	MICBPat#5	AF021225	
MICB*012	MICB*012	MICB-006	MICBPat#6	AF021226	
MICB*013	MICB*013	MICB01022V	SWEIG007	AJ251160, AJ251161	
MICB*014	MICB*014	MICB0103101v, MICB*H0180101	EMJ, PUM326-95	AJ251156, AJ251157, AJ606912, AJ606913, AJ606914	(444)^b^
MICB*015	MICB*015	MICB	B692	AJ563706	
MICB*016	MICB*016	MICB*H0160101	PUM300-95	AJ606906, AJ606907, AJ606908	(444)
MICB*018	MICB*018	MICB*H0190101	PUM259-95	AJ606915, AJ606916, AJ606917	(444)
MICB*019	MICB*019	MICB*H020010	PUM219-95	AJ606918, AJ606919, AJ606920	(444)
MICB*020	MICB*020	MICB*H0210101	PUM368-95	AJ606921, AJ606922, AJ606923	(444)
MICB*021N	MICB*021N	MICB*H0140101N	PUM607-94	AJ606926, AJ606927, AJ606928	(444)
MICB*022	MICB*022	MICB*H0150101	PUM246-95	AJ606929, AJ606930, AJ606931	(444)

a Allele names given in bold type have been assigned since the 2004 Nomenclature report.

b This reference is to a confirmatory sequence.

**Table 2:** Designations of HLA-A alleles

HLA allele^a^	Pre 2010 designation	HLA specificity	Previous equivalents	Individual or cell line from which the sequence was derived	Accession number	References or submitting author(s)
A*01:01:01:01	A*01010101	A1	—	LCL721, MOLT4, PP, COX, APD, B4702	X55710, M24043, Z93949, AJ278305 AL645935, CR759913, EU44570	(29)^b^, S Beck^b^, (30)^b^
A*01:01:01:02N	A*01010102N	Null	A*01ne	CTM7681276	AY973959	JL Vicario
A*01:01:02	A*010102	A1	—	GN00348	AF248059, AF248060	
A*01:01:03	A*010103	A1	—	27720, 27785	AJ697951, AJ697952, AJ697953, AJ697954	
A*01:01:04	A*010104	A1	—	DA894.3	DQ485722	(31)
A*01:01:05	A*010105	A1	—	99706	AM850141	J Enczmann
A*01:01:06	A*010106	A1	—	HN-54982-5, HN-04477-1	FJ765908, GQ180284	Histogenetics
A*01:01:07	A*010107	A1	—	HN-34603-1	FJ392136	Histogenetics
A*01:01:08	A*010108	A1	—	HN-2874373	FJ224239	Histogenetics
A*01:01:09	A*010109	A1	—	HN-98001-3	FJ619449	Histogenetics
A*01:01:10	A*010110	A1	—	HN-64111-0, HN-74486-0, HN-99884-6, HN-21154-4	FJ619453, FJ765917, FJ898483, FJ976757	Histogenetics
A*01:01:11	A*010111	A1	—	HN-1066965	FJ392158	Histogenetics
A*01:01:12	A*010112	A1	—	HN-74212-0	FJ765916	Histogenetics
A*01:01:13	A*010113	A1	—	HN-00401-6	FJ875535	Histogenetics
A*01:02	A*0102	A1	—	DAUDI, NT00610	U07161, EF156371	CK Hurley^b^
A*01:03	A*0103	A1^c^	—	04VC, UCLA 144, BONIFACE, FU-GP, JF-GP, BR-GP, RENOPhil, SZ-59	Y12469, Y12470, AJ002528, AJ002529, AF098160, FM882249, FJ969856	A Dormoy^b^, HY Zou^b^
A*01:04N	A*0104N	Null	A*01N, A*01N-Ca	PELa, PEFr, PEPi, PEPa, CAFL, CB1280	Z93776, Z97027, AJ011125, AJ011126, AJ011127	
A*01:06	A*0106	—	A*0101V	GN00280, NT00730	AF143231, AF143232, EF563145	CK Hurley^b^
A*01:07	A*0107	A1	BLP-N	BLP-N	AF219632, AF219633	
A*01:08	A*0108	A1	A*01	34040	AJ277792	
A*01:09	A*0109	—	—	T110	AJ315641	
A*01:10	A*0110	—		BY00053	AY562128, AY562129	
A*01:11N	A*0111N	Null	—	AHPEN	AY836150, AY574934	(32)
A*01:12	A*0112	—	—	NT00524, NT00586	AY870930, AY870931, DQ086786, DQ086787	(33)
A*01:13	A*0113	—	—	NT00519	AY870932, AY870933	(33)
A*01:14	A*0114	—	—	K95443	AJ829713	(34)
A*01:15N	A*0115N	Null	—	GDA1N, BY00202, FRFPO06277	AY362881, AY362882, AY362883, EU029771, FM164485	(35), (36)^b^, A Dormoy^b^
A*01:16N	A*0116N	Null	—	42989	AM040978	J Rowlands
A*01:17	A*0117	—	—	NT00621, NT00645	DQ227987, DQ227988, DQ354430, DQ354431	(33)
A*01:18N	A*0118N	Null	—	GH	AY174762, AY174763	(37)
A*01:19	A*0119	A1	—	AML145, FPX3606	AM183219, AM183220	(38)
A*01:20	A*0120	—	—	MOHA43089AN	AM183561	AM Little
A*01:21	A*0121	—	—	MHHAKB-266882	AM493249	R Blasczyk
A*01:22N	A*0122N	Null	—	73891	EF471359	MS Leffell
A*01:23	A*0123	—	—	BY00151, BY00157	EF484940	(36)
A*01:24	A*0124	—	—	BY00154	EF484943	(36)
A*01:25	A*0125	—	—	BY00159, BY00186, HN-225073, HN-2623, HN-9288880	EF484948, EF656471, FJ765878, FJ594673, FJ875683	(36), Histogenetics^b^
A*01:26	A*0126	—	—	BY00162, NT00736	EF536018, EF591033	(36)
A*01:27N	A*0127N	Null	—	156519SR	AM503963	(39)
A*01:28	A*0128	—	—	BJ038	EF672087	(40)
A*01:29	A*0129	—	—	BY00201, HN-08587-2	EU029775, FJ594683	(36), Histogenetics^b^
A*01:30	A*0130	—	—	BY00200	EU029776	(36)
A*01:31N	A*0131N	Null	—	CTJ-23589	FJ200655	L Yan
A*01:32	A*0132	—	—	CDC09262008	FJ227105	S Cordovado
A*01:33	A*0133	—	—	BY00386, HN-3282501	FJ517146, FJ392140	CK Hurley, Histogenetics^b^
A*01:34N	A*0134N	Null	—	37935	FM209476, GQ281054	(41), K Cao^b^
A*01:35	A*0135	—	—	BY01014, HN-756979, HN-36829-1	FJ797388, FJ619457, FJ976746	C Hurley, Histogenetics^b^
A*01:36	A*0136	—	—	HN-13116-0	FJ224148	Histogenetics
A*01:37	A*0137	—	—	BY00469	FJ985987	C Hurley
A*01:38	A*0138	—	—	HN-06971-5, HN-76157-8, HN-26803-9	FJ224151, FJ224178, FJ976756	Histogenetics
A*01:39	A*0139	—	—	HN-74052-2, HN-88851-1	FJ224171, GQ160995	Histogenetics
A*01:40	A*0140	—	—	HN-70102-1, HN-67074-7, HN-70277-6, 09211161, HN-98150-3, HN-32298-1, HN-09864-1, HN-90444-8, HN-23474-6, HN-33290-4, HN-33390-2, HN-41184-5, HN-28548-2	FJ224194, FJ785213, FJ940763, GQ351347, FJ997957, GQ160976, GQ161011, GQ345028, GQ401177, GQ401178, GQ401179, GQ859516, GQ859526	Histogenetics, D Fuerst^b^
A*01:41	A*0141	—	—	HN-75146-5	FJ224196	Histogenetics
A*01:42	A*0142	—	—	HN-42569-4	FJ224240	Histogenetics
A*01:43	A*0143	—	—	BY00515	GQ426484	CK Hurley
A*01:44	A*0144	—	—	HN-5235988	FJ392147	Histogenetics
A*01:45	A*0145	—	—	ELTU444829AN	FN546184	SGE Marsh
A*01:46	A*0146	—	—	HN-5357	FJ392149	Histogenetics
A*01:47	A*0147	—	—	HN-8045382	FJ619460	Histogenetics
A*01:48	A*0148	—	—	HN-2260722	FJ619470	Histogenetics
A*01:49	A*0149	—	—	HN-46950-9, HN47555-4	FJ765918, FJ875541	Histogenetics
A*01:50	A*0150	—	—	HN-1446866	FJ765935	Histogenetics
A*01:51	A*0151	—	—	HN-384D	FJ785207	Histogenetics
A*02:01:01:01	A*02010101	A2	A2.1	LCL721, JY, GM637, GRC138, T5-1, JD, BOLETH, B9537, 08009402	K02883, M84379, X02457, AJ555412, AF055066, EU445471, GQ996941	(42)^b^, (30)^b^, Y Xu^b^
A*02:01:01:02L	A*02010102L	Low A2	HLA-A2 Low	Gaff, CTM-77, PER (II.2)	AJ575565, U02935, AM286552	(43)^b^
A*02:01:01:03	A*02010103	A2	—	HSR131658	AM943368	(44)
A*02:01:02	A*020102	A2	—	CHI564, CHI557	Y14624, Y14625	
A*02:01:03	A*020103	A2	A*02DKP	DKP, 19673946	AF108449, AF108450, AF255333, AF190713, AF190714, AF190715	
A*02:01:04	A*020104	A2	A*02New	NM4a189	AF139832, AF139833	
A*02:01:05	A*020105	A2	A*02AR	32711	AJ277793	
A*02:01:06	A*020106	A2	A0201V3	JCB11458	AB032595, AB048347	
A*02:01:07	A*020107	A2	A*02011V6	TBC30114	AB087507, AB087508, AB087509	
A*02:01:08	A*020108	A2	A*02LESA	LESA	AJ506046, AJ506053, AJ506055	
A*02:01:09	A*020109	A2	—	72970, 0200402901, 0200402597, 0200402708	AY158885, AY158886, AM040715	S Ulrich^b^
A*02:01:10	A*020110	A2	—	TBC44183	AB167821, AB167822, AB167823	
A*02:01:11	A*020111	A2	A*0201V11	TBC48658	AB187509	
A*02:01:12	A*020112	A2	A*02ERN0605	Z-00016417	AM050155	(45)
A*02:01:13	A*020113	A2	A*02AHU0805	MHHDynal-55	AM114060	R Blasczyk
A*02:01:14	A*020114	A2	—	D54464, D54467	EF495117	LA Baxter-Lowe
A*02:01:15	A*020115	A2	—	CTM-6696126	EF526079	(46)
A*02:01:17	A*020117	A2	—	KILIMir	AM712907	A Dormoy
A*02:01:18	A*020118	A2	—	MHHI-603528, A15111, HN-93790-8, HN-81995-4	AM906168, EU376018, FJ594664, FJ594671	R Blasczyk, A Amoroso^b^, Histogenetics^b^
A*02:01:19	A*020119	A2	—	LUMC-A41	AM932284	JDH Anholts
A*02:01:21	A*020121	A2	—	CDC09282008	FJ227107	S Cordovado
A*02:01:22	A*020122	A2	—	BY00362, HN-70104-9, HN-53152-5, HN-54826-1	FJ358710, FJ224202, FJ594711, FJ619455	CK Hurley, Histogenetics^b^
A*02:01:23	A*020123	A2	—	HN-43008-1, HN-04209-3, HN00116-4, HN-32507-0, HN-62729-8, HN-50658-9, HN-77485-1, HN31306-3, HN45398-1, HN38885-6	FJ224141, FJ224157, FJ224174, FJ392155, FJ600644, FJ224185, FJ765931, FJ785204, FJ875542, GQ240539	Histogenetics
A*02:01:24	A*020124	A2	—	HN-13730-6, HN-60958-8, HN-1224804, HN-26306-8, HN-85084-9, HN-07368-0, HN-01844-6	FJ224149, FJ392151, FJ392130, FJ594706, FJ898481, FJ898482, GQ240547	Histogenetics
A*02:01:25	A*020125	A2	—	HN-95311-7, HN-02855-5, HN-03558-4, HN-37024-8, HN-51902-7	FJ224154, FJ224165, FJ224166, FJ224167, GQ859517	Histogenetics
A*02:01:26	A*020126	A2	—	HN-94748-1, HN-46549-4, HN-22363-0, HN-54671-6, HN-43494-7, HN61784-1, HN-52186-0	FJ224163, FJ224190, FJ224211, FJ538271, FJ619452, FJ785201, FJ875538	Histogenetics
A*02:01:27	A*020127	A2	—	HN-51440-6, HN30847-7, HN-68729-5	FJ392134, FJ392132, FJ392152	Histogenetics
A*02:01:28	A*020128	A2	—	HN-B-181556	FJ224198	Histogenetics
A*02:01:29	A*020129	A2	—	HN-76995-8	FJ224213	Histogenetics
A*02:01:30	A*020130	A2	—	HN-41590-1, HN20662-3	FJ224218, FJ392141	Histogenetics
A*02:01:31	A*020131	A2	—	HN-3341893, HN-01102-3, HN-2119456	FJ224237, FJ224244, FJ765889	Histogenetics
A*02:01:32	A*020132	A2	—	HN-00748-4, HN-15296-7, HN-56401-03	FJ392135, FJ619446, FJ765911	Histogenetics
A*02:01:33	A*020133	A2	—	HN-30259-5	FJ619441	Histogenetics
A*02:01:34	A*020134	A2	—	HN-79594-0	FJ619451	Histogenetics
A*02:01:35	A*020135	A2	—	HN-92009-0	FJ619454	Histogenetics
A*02:01:36	A*020136	A2	—	HN-51291-4	FJ765907	Histogenetics
A*02:01:37	A*020137	A2	—	HN-66993-7, HN-60669-8, HN58101-4	FJ538268, FJ549404, FJ875539	Histogenetics
A*02:01:38	A*020138	A2	—	HN-81213-1	FJ549402	Histogenetics
A*02:01:39	A*020139	A2	—	HN-72740-4	FJ549403	Histogenetics
A*02:01:40	A*020140	A2	—	HN-673307	FJ594708	Histogenetics
A*02:01:41	A*020141	A2	—	HN-55832-0	FJ600650	Histogenetics
A*02:01:42	A*020142	A2	—	HN-86940-0	FJ875534	Histogenetics
A*02:02	A*0202	A2	A2.2F	M7, 951314	M17566, M17568, X94566	
A*02:03:01	A*020301	A203	A2.3	DK1, 951315, SZ-56	U03863, M17567, M19670, X94567, FJ969859	HY Zou^b^
A*02:03:02	A*020302	A203	A*0203V1	TBC8212	AB179756, AB179757, AB179758	
A*02:04	A*0204	A2	—	RML, AN, 951316	X57954, M86404, X94568 AJ297476	
A*02:05:01	A*020501	A2	A2.2Y	WT49, AM, SUS-NF, 951317	U03862, L76290, X94569	
A*02:05:02	A*020502	A2	—	HN-85564-3, HN-68494-4, HN04903-3, HN-94313-4, HN-35238-4, HN65045-3, HN55205-5, HN-78802-2, HN-71835-0, HN-83850-2, HN-52933-5, HN02597-9	FJ224147, FJ224159, FJ224191, FJ224238, FJ619439, FJ785202, FJ785203, FJ875537, FJ952578, GQ180304, GQ180321, GQ240548	Histogenetics
A*02:05:03	A*020503	A2	—	HN-50922-4	FJ224235	Histogenetics
A*02:06:01	A*020601	A2	A2.4a	CLA, T7526, 951318, SZ-52	M24042, X94570, FJ969860	HY Zou^b^
A*02:06:02	A*020602	A2	A*0206V3	TBC31680	AB179825, AB179826, AB179827	
A*02:06:03	A*020603	A2	—	TBC103851	AB180084, AB180085, AB180086	
A*02:06:04	A*020604	A2	—	BY00164	EF536017	(36)
A*02:06:05	A*020605	A2	—	BY00193, BY00264	EU029783, EU185509	(36), CK Hurley^b^
A*02:06:06	A*020606	A2	—	JMDP01K001	AB427093	K Tadokoro
A*02:06:07	A*020607	A2	—	HN-32257-7	FJ600645	Histogenetics
A*02:06:08	A*020608	A2	—	HN-2356550	FJ538269	Histogenetics
A*02:06:09	A*020609	A2	—	HN-2111765	FJ792529	Histogenetics
A*02:07	A*0207	A2	A2.4b	KNE, KTO, SZ-57, 7550800684	D50458, FJ969857, GQ996942	HY Zou^b^, Y Xu^b^
A*02:08	A*0208	A2	A2.4c	KLO	X94571	
A*02:09	A*0209	A2	A2-OZB	OZB	AJ249241	
A*02:10	A*0210	A210	A2-LEE	XLI-ND, 951322, SZ-58	Z23071, X94572, FJ969858	HY Zou^b^
A*02:11	A*0211	A2	A2.5	KIME, GRC138, 951366	X60764, M84377, X94573	
A*02:12	A*0212	A2	—	KRC033, KRC005	M84378	
A*02:13	A*0213	A2	A2SLU	SLUGEO, 30047	Z27120, AJ544281, AJ544282, AJ544283	
A*02:14	A*0214	A2	A2’1S'	1S, ML1260	Z30341, AF305699	
A*02:15N	A*0215N	Null	HLA-Anull	TSU	D38525	
A*02:16	A*0216	A2	A2’TUB’	TUBO	Z46633	
A*02:17:01	A*021701	A2	A*New	AMALA, LZL, C.S.	U18930, L43526, L43527, X89707, X89708	
A*02:17:02	A*021702	A2	—	H.K	Y13267	
A*02:18	A*0218	A2	A*2K	ENDO	D83515	
A*02:19	A*0219	—	A-02X09	TOB-81	L76936	
A*02:20:01	A*022001	A2	—	BI, 23675	X96724, AJ544284, AJ544285, AJ544286	
A*02:20:02	A*022002	A2	A*02New	MT-SN	AJ276069	
A*02:21	A*0221	A2	A206W331R	W331R	U56825	
A*02:22:01	A*022201	A2	A-02x28	TER-109, OCA1/4	U76398, U76399, Y11441	
A*02:22:02	A*022202	A2	—	NT01044	FJ797359	CK Hurley
A*02:24	A*0224	A2	A*02JG	11952547, 13041452, RP122	Y11201, Y11202, AF036921, AF001956, AF001957	
A*02:25	A*0225	A2	—	NP814, 970551	U70863, Y13028	
A*02:26	A*0226	—	—	C.C	AF008933, U90138, U90139	
A*02:27	A*0227	—	A*02TK	TRK	AJ001269	
A*02:28	A*0228	—	—	NM3298, TBC50544	AF041365, AF041366, AB213259	M Satake^b^
A*02:29	A*0229	A2	—	RAG, NT01112	AF053479, AF053480, AF012766, GQ373161	CK Hurley^b^
A*02:30	A*0230	—	A*02WP	NM332, CL154, WP	AF101162, AF101163, AF116215, AF133091, AF133092	
A*02:31	A*0231	A2	A*02011V	19703222	AF113923, AF113924	
A*02:32N	A*0232N	Null	A*02xxN	NDS-AN	AF117228	
A*02:33	A*0233	—	A*0201New	CL-PPA	AF140506	
A*02:34	A*0234	A2	A*AAT	AAT	AF129429, AF129430, AF129431	
A*02:35:01	A*023501	A2	A*0201V	GN00279, GN00300, NT00697	AF140600, AF140601, AF157310, AF157311, EF195107	CK Hurley^b^
A*02:35:02	A*023502	A2	—	UKE-Fox	AJ704546	
A*02:35:03	A*023503	A2	—	BY00168	EF563137	(36)
A*02:36	A*0236	—	A*02011V	GN00297	AF157308, AF157309	
A*02:37	A*0237	—	A*0212Variant	GN00303	AF157563, AF157564	
A*02:38	A*0238	—	A*0213V	GN00260, GN00286, GN00346	AF135542, AF135543, AF181101, AF181102, AF232705, AF232706	
A*02:39	A*0239	—	A*02011V	GN00308, 99-2203	AF173873, AF173874, AF198352, AF198353	
A*02:40	A*0240	—	A*CB2406	CB2406, CB2406(MUM)	AF194531, AF194532	
A*02:41	A*0241	A2	A*02CIS	KMP01-636	AF170580, AF170581	
A*02:42	A*0242	A2	A0201V2	JCB6898, JMDP36K030	AB032594, AB474566	K Tadokoro^b^
A*02:43N	A*0243N	Null	A*02ROUB	ROUB	AJ251960	
A*02:44	A*0244	—	—	GN00337, TBC12381	AF226834, AF226835, AB183315	
A*02:45	A*0245	—	—	1998-302-2581	AF251354, AF251355	
A*02:46	A*0246	A2	A*02COL	COL	AJ289156	
A*02:47	A*0247	—	—	GN00378	AF291839, AF291840	
A*02:48	A*0248	—	—	GN00381, BY00349	AF299250, AF299251, FJ174581	CK Hurley^b^
A*02:49	A*0249	A2	A*02new	22697, 05TP1337	AJ291697, AJ291698, AJ971317	M Bengtsson^b^
A*02:50	A*0250	A2	A*02X68	A02X68	AF162678, AF162679	
A*02:51	A*0251	—	—	2000-7-206, Taramahara31	AF372047, AF372048, AJ457988	
A*02:52	A*0252	—	—	JSILV	AF417237, AF417238	
A*02:53N	A*0253N	Null	—	Yanli, VTIS25793, CMC4, NS0657	AF416455, AF479485, AF479486, AY310876, AY310875, AH012987, AB233410	T Kinoshita^b^
A*02:54	A*0254	—	—	2000-084-3329, NT00746	AF440104, AF440105, EU185511	CK Hurley^b^
A*02:55	A*0255	—	—	1PFA8, NT00783	AY045739, AY045740, EU484046	CK Hurley^b^
A*02:56:01	A*025601	—	—	MYTCZA-A202x	AJ430523, AJ430524, AJ431714, AJ431715	
A*02:56:02	A*025602	—	—	HB08053124	EU998646	TD Lee
A*02:57	A*0257	—	—	Taramahara35	AJ457989	
A*02:58	A*0258	—	—	RL*D	AY100700, AY100701	
A*02:59	A*0259	A2	A*02011V4	TBC16525	AB086836, AB086837, AB086838	
A*02:60	A*0260	—	—	A3674, NT00703, BRABRI, HN-29051-0	AY128677, EF370118, EU580406, FJ594655	CK Hurley^b^, (47)^b^, Histogenetics^b^
A*02:61	A*0261	—	—	B02-4183	AY206696, AY206697	
A*02:62	A*0262	—	—	MAR02V	AJ538192, AJ538193	
A*02:63	A*0263	—	—	GN00430	AY267907, AY267908	
A*02:64	A*0264	—	—	7A356	AY297166, AY297167	
A*02:65	A*0265	—	A2x19	Weedan	AJ566139	
A*02:66	A*0266	A2	—	28373	AY330170	
A*02:67	A*0267	A2	—	Cam1	AJ579651, AJ579652, AJ579653	
A*02:68	A*0268	A2	—	IaPa21424	AJ621243	
A*02:69	A*0269	—	—	34617S	AY509616	
A*02:70	A*0270	A2	—	TBC10647	AB176446, AB176447, AB176448	
A*02:71	A*0271	A2	A*0201V7	TBC316014	AB179714, AB179715, AB179716	
A*02:72	A*0272	A2	A*0206V4	TBC561095	AB179863, AB179864, AB179865	
A*02:73	A*0273	A2	—	TBC24400	AB183420	
A*02:74:01	A*027401	A2	—	F154	AY796418, AY796419, AY796420	
A*02:74:02	A*027402	A2	—	VTIS12570	AY829217, AY829218	BD Tait
A*02:75	A*0275	—	—	TBC50206	AB196347	
A*02:76	A*0276	—	—	VTIS116362, JMDP01K005	AY826750, AY826751, AB429497	BD Tait, K Tadokoro^b^
A*02:77	A*0277	A2	—	VTIS118387, CTM1689326	AY826752, AY826753, DQ188811	BD Tait, JL Vicario^b^
A*02:78	A*0278	A2	—	SH2681	AY834199	(48)
A*02:79	A*0279	A2	—	LYZ	AY856830	(49)
A*02:80	A*0280	—	—	NT00529	AY956754, AY956755	(33)
A*02:81	A*0281	—	—	DKM326178	AY973958	(50)
A*02:82N	A*0282N	Null	—	TER-313, TER-314, BY00095	AY974249, AM040196, DQ465610	D Smith, R Blasczyk^b^, CK Hurley^b^
A*02:83N	A*0283N	Null	—	Z-00013003	AJ965497	(51)
A*02:84	A*0284	—	—	87089, NT00747	DQ072942, EU185510	MS Leffell, CK Hurley^b^
A*02:85	A*0285	—	—	PC10, NT00656, HN-3232099	AJ972405, DQ436822, DQ436823, FJ792530	M Bengtsson, CK Hurley^b^, Histogenetics^b^
A*02:86	A*0286	—	—	2005052734, NT00618	DQ089701, DQ089702, DQ205715, DQ205716	(52), CK Hurley^b^
A*02:87	A*0287	—	—	2005061422, BY00382	DQ139310, DQ139311, FJ464340	(53), CK Hurley^b^
A*02:88N	A*0288N	Null	—	UKE-J-DD	AM076769	T Binder
A*02:89	A*0289	A2	—	CTM5689590	DQ188810	(50)
A*02:90	A*0290	A2	—	Xian11519, BY00093	DQ234081, DQ234082, DQ234083, DQ455021	(54), CK Hurley^b^
A*02:91	A*0291	—	—	2005102723	DQ348073, DQ348074	(55)
A*02:92	A*0292	—	—	NT00641	DQ354438, DQ354439	(33)
A*02:93	A*0293	—	—	VTIS116760, HN-06745-5	DQ400528, DQ400529, FJ594651	BD Tait, Histogenetics^b^
A*02:94N	A*0294N	Null	—	VTIS138070	DQ400512, DQ400513	BD Tait
A*02:95	A*0295	A2	—	VTIS119848	AY505488, AY505489	BD Tait
A*02:96	A*0296	—	—	U9121.A, X0003339, HN-14542-6, HN-71569-0, HN-74939-1, HN-92153-0, HN-1125835, HN-23667-9	AB121736, AY897620, FJ594675, FJ594681, FJ594657, FJ594663, FJ594669, FJ594678	P Paul, L Mele^b^, Histogenetics^b^
A*02:97:01	A*029701	A2	—	A6793	DQ409217	(56)
A*02:97:02	A*029702	A2	—	I2009-1365	GQ215661	S Pereira
A*02:99	A*0299	—	—	BY00094	DQ465611	(57)
A*02:101	A*9201	—	—	NT00659	DQ473292	(33)
A*02:102	A*9202	—	—	m1210, m1270, ML1359	DQ494174, EU095649	(58), M Lao^b^
A*02:103	A*9203	—	—	K9503143	DQ485737	(59)
A*02:104	A*9204	—	—	90340	DQ902554	MS Leffell
A*02:105	A*9205	A2	—	0601119, 0601318	AM404080	E Palou
A*02:106	A*9206	—	—	HZB13619	EF062306	(60)
A*02:107	A*9207	—	—	VTIS138774	EF088206	BD Tait
A*02:108	A*9208	—	—	B13680	EF092417	(61)
A*02:109	A*9209	—	—	152173	DQ885885	K Hirv
A*02:110	A*9210	—	A*02MVE1006	MHHN-189776, HN-145436, HN-21668-1	AM412221, FJ594662, FJ594676	R Blasczyk, Histogenetics^b^
A*02:111	A*9211	—	A*02MVE0906	MHHN-189515	AM407886	R Blasczyk
A*02:112	A*9212	—	—	TBC63782	AB292417	M Satake
A*02:113N	A*9213N	Null	—	1416999, BY00184	AM422607, AM422608, AM422609, EF656469	(62), CK Hurley^b^
A*02:114	A*9214	—	—	BY00158, HN-6373184	EF484947, FJ594659	(36), Histogenetics^b^
A*02:115	A*9215	—	—	BY00160	EF490363	(36)
A*02:116	A*9216	—	—	S100	EF468681	(63)
A*02:117	A*9217	—	—	Henan10	EF526712	B Zhang
A*02:118	A*9218	—	—	BY00171	EF563140	(36)
A*02:119	A*9219	—	—	BY00170	EF563139	(36)
A*02:120	A*9220	—	—	BY00169	EF563138	(36)
A*02:121	A*9221	—	—	SCCA-1, B8972	EF534104, EF632301	M Sprague, L Yan
A*02:122	A*9222	—	—	BY00178, BY00183, BY00344	EF591030, EF656468, EU924808	(36)
A*02:123	A*9223	—	—	BY00176, DCI29186, HN-13677-0	EF591028, EU031440, FJ594652	(36), S Fossey^b^, Histogenetics^b^
A*02:124	A*9224	—	—	SCU00927	EF579799	(50)
A*02:125N	A*9225N	Null	—	06d05372	AM711559	(64)
A*02:126	A*9226	—	—	2001238PD	AB303947	H Inoko
A*02:127	A*9227	—	—	BJ041	EF687770	Z Wang
A*02:128	A*9228	—	—	BY00199	EU029777	(36)
A*02:129	A*9229	—	—	BJ040, BY00351	EF685282, FJ187799	L Ni, CK Hurley^b^
A*02:130	A*9230	A2	—	K18125	AB354233	E Maruya
A*02:131	A*9231	—	—	76460	AM778130	J Enczmann
A*02:132	A*9232	—	—	ESPUCVAL	AM849479	A Dormoy
A*02:133	A*9233	—	—	99533, HN-8542-4, HN-21691-3	AM850142, FJ594648, FJ765882	J Enczmann, Histogenetics^b^
A*02:134	A*9234	—	—	LUMC-A21	AM746782	(64)
A*02:135	A*9235	—	—	FBCCB5942	AB355648	Y Kuroda
A*02:136	A*9236	—	—	BY00174	EU256485	(36)
A*02:137	A*9237	A2	—	4034892	EU167538	(65)
A*02:138	A*9238	A2	—	CambsA2	AM922173	R Goodman
A*02:139	A*9239	—	—	7221358, HN-20053-8	EU431985, FJ594650	J Mytilineos, Histogenetics^b^
A*02:140	A*9240	A2	—	A11962	EU376019	A Amoroso
A*02:141	A*9241	—	—	CS00001, CS00002, HN-80638-1	EU559334, EU559335, FJ594649	K Cao, Histogenetics^b^
A*02:142	A*9242	—	—	JMDP01K002	AB427094	K Tadokoro
A*02:143	A*9243	—	—	JMDP01K003	AB429008	K Tadokoro
A*02:144	A*9244	—	—	JMDP01K004	AB429218	K Tadokoro
A*02:145	A*9245	—	—	BJ54	EU649706	Z Zhang
A*02:146	A*9246	—	A*0201V1	JMDP36K001, BY00384, HN-7435974	AB434692, FJ464338, FJ765884	K Tadokoro, CK Hurley^b^, Histogenetics^b^
A*02:147	A*9247	—	—	NT00793	EU682451	CK Hurley
A*02:148	A*9248	—	—	BY00332	EU812537	CK Hurley
A*02:149	A*9249	—	—	7215356, HN-03878-5, HN-53007-3	EU445572, FJ224162, FJ976758	A Vigh, Histogenetics^b^
A*02:150	A*9250	—	—	G048	EU812119	(66)
A*02:151	A*9251	—	—	7080859, HN-95852-8, HN-09534-7, HN-30146-7, HN-90554-1, HN-18603-5, HN-16225-7, HN-42729-6, HN-90336-8, HN-74229-4	FM204792, FJ594703, FJ594640, FJ594644, FJ594646, FJ600654, FJ765903, FJ538272, FJ538273, FJ600632, FJ600641, FJ765895, FJ765899, FJ765942	(67), Histogenetics^b^
A*02:152	A*9252	—	—	BY00348	FJ174579	CK Hurley
A*02:153	A*9253	—	—	BY00375, HN-0168836	FJ464347, FJ765885	CK Hurley, Histogenetics^b^
A*02:154	A*9254	—	—	BY00388, HN-05973-4, HN-47750-2, HN-50748-0	FJ517144, FJ765945, FJ765939, FJ785210	CK Hurley, Histogenetics^b^
A*02:155	A*9255	—	—	BY00389	FJ517143	CK Hurley
A*02:156	A*9256	—	—	BY00391	FJ517141	CK Hurley
A*02:157	A*9257	—	—	30883	FJ652064	W Dong
A*02:158	A*9258	—	—	HN-91757-5, HN-53878-6, HN-40256-2, HN-19193-4, HN-61351-2, HN-35480-4, HN-28120-2, HN-85226-8, HN-00731-9, HN-81176-3, HN-22166-0, HN-24072-8, HN30155-4, HN-99545-7, HN-95792-5, HN-22292-8, HN-86446-8, HN-63730-8, HN-77431-0, HN-73984-9	FJ224142, FJ619450, FJ765905, FJ222574, FJ224136, FJ224183, FJ224217, FJ224227, FJ619443, FJ619444, FJ594704, FJ594705, FJ765897, FJ765909, FJ976871, FJ222568, GQ180322, GQ859520, GQ900557, GQ994041	Histogenetics
A*02:159	A*9259	—	—	HN-10039-5	FJ224144	Histogenetics
A*02:160	A*9260	—	—	HN-14041-7, HN-08703-0, HN-69153-4, HN38048-7	FJ224146, FJ224152, FJ222556, GQ859519	Histogenetics
A*02:161	A*9261	—	—	HN-97586-2, HN-91405-5, HN-91271-1, HN-40502-3	FJ224155, FJ594713, FJ224203, FJ875546	Histogenetics
A*02:162	A*9262	—	—	HN-23604-4	FJ392131	Histogenetics
A*02:163	A*9263	—	—	HN-56426-0, HN-71637-4	FJ392133, FJ765896	Histogenetics
A*02:164	A*9264	—	—	HN-181937-4, HN-76878-9, HN-18979-6, HN-28220-2, HN-90276-3	FJ392148, FJ224173, FJ765906, FJ765910, FJ875533	Histogenetics
A*02:165	A*9265	—	—	HN-20600-5, HN-56502-0	FJ-392153, FJ224125	Histogenetics
A*02:166	A*9266	—	—	HN-22727-4, HN-30314-8, HN-05493-4, HN-78285-9	FJ392154, FJ222557, GQ859525, GQ900556	Histogenetics
A*02:167	A*9267	—	—	HN-67321-2	FJ554624	Histogenetics
A*02:168	A*9268	—	—	HN-87835-4	FJ765914	Histogenetics
A*02:169	A*9269	—	—	BY00453, MSNL3154AN	FJ842964, FN553433	CK Hurley, SGE Marsh^b^
A*02:170	A*9270	—	—	ZhangheA2	FJ810057	J He
A*02:171:01	A*927101	—	—	YaobintaoA2	FJ810060	J He
A*02:171:02	A*927102	—	—	BY00576	GU256013	CK Hurley
A*02:172	A*9272	—	—	HN-43576-7	FJ222558	Histogenetics
A*02:173	A*9273	—	—	HN-12839-4, HN-81935-3, HN-08388-8, HN-37051-2	FJ222570, FJ224205, FJ222569, FJ792527	Histogenetics
A*02:174	A*9274	—	—	HN-41136-0	FJ224126	Histogenetics
A*02:175	A*9275	—	—	HN-72502-8	FJ224153	Histogenetics
A*02:176	A*9276	—	—	HN-00973-7	FJ224164	Histogenetics
A*02:177	A*9277	—	—	NT01027, HN-9035051, HN-674214	FJ976685, FJ489876, FJ594707	CK Hurley, Histogenetics^b^
A*02:178	A*9278	—	—	NT01055	FJ976701	CK Hurley
A*02:179	A*9279	—	—	BY00471	FJ999994	CK Hurley
A*02:180	A*9280	—	—	HN-24564-0, HN-12260-8	FJ224187, GQ449615	Histogenetics
A*02:181	A*9281	—	—	HN-79304-6	FJ224201	Histogenetics
A*02:182	A*9282	—	—	HN-20514-0, AKB-650304	FJ224210, FN430730	Histogenetics, R Blasczyk^b^
A*02:183	A*9283	—	—	HN-56519-2, HN-2453129, HN-64358-3, HN-0871736	FJ224223, FJ032365, FJ502338, FJ765887	Histogenetics
A*02:184	A*9284	—	—	HN-40198-6	FJ224224	Histogenetics
A*02:185	A*9285	—	—	HN-04097-0	FJ224232	Histogenetics
A*02:186	A*9286	—	—	HN-5491441	FJ224245	Histogenetics
A*02:187	A*9287	—	—	HN-36531-1	FJ600648	Histogenetics
A*02:188	A*9288	—	—	HN-71612-5	FJ600649	Histogenetics
A*02:189	A*9289	—	—	HN-60457-1, HN-70469-6	FJ600653, FJ659000	Histogenetics
A*02:190	A*9290	—	—	NT01108, HN-83336-8	GQ373166, FJ785211	CK Hurley, Histogenetics^b^
A*02:191	A*9291	—	—	JMDP01K042	AB512685	K Tadokoro
A*02:192	A*9292	—	—	09213041	GQ422609	D Fuerst
A*02:193	A*9293	—	—	BY00514	GQ426485	CK Hurley
A*02:194	A*9294	—	—	HN-2249071	FJ392143	Histogenetics
A*02:195	A*9295	—	—	HN-87787-2	FJ594647	Histogenetics
A*02:196	A*9296	—	—	HN-23582-9	FJ619440	Histogenetics
A*02:197	A*9297	—	—	HN-12024-6	FJ619447	Histogenetics
A*02:198	A*9298	—	—	HN-38943-8	FJ619448	Histogenetics
A*02:199	A*9299	—	—	HN-61873-4	FJ765898	Histogenetics
A*03:01:01:01	A*03010101	A3	A3.1	JG, JD, PP, AP630, CGM1, PGF, B9563	X00492, U32184, AB023056, AL671277, EU445472	(29)^b^, (30)^b^
A*03:01:01:02N	A*03010102N	Null	A*03RS Null	11234121, 41982	AJ532608, AM041068	J Rowlands^b^
A*03:01:01:03	A*03010103	A3	—	GPT	AJ748743	
A*03:01:02	A*030102	A3	DT18-A*0301v	DT18	AF053128, AF053129	
A*03:01:03	A*030103	A3	A*03NJ	12244015, NM4a227	Y17000, Y17001, AF146365, AF146366	
A*03:01:04	A*030104	A3	—	MM2004/10/01, MM2004/10/02, JMDP36K031	AY754872, AY754873, AB536689	K Tadokoro^b^
A*03:01:05	A*030105	A3	—	Z-00023171	AM261460	(68)
A*03:01:06	A*030106	A3	—	LUMC-A5, 79255	AM746339	(64)
A*03:01:07	A*030107	A3	—	CDC09252008	FJ227104	S Cordovado
A*03:01:08	A*030108	A3	—	B26636	FJ355926	(69)
A*03:01:09	A*030109	A3	BJ059	BJ59	FJ428217	Z Zhang
A*03:01:10	A*030110	A3	—	BY00387	FJ517145	CK Hurley
A*03:01:11	A*030111	A3	—	HN-80503-6, HN-26124-9, HN-80480-6, HN-95921-1, HN-79481-8, HN-59431-3, HN-45502-8, HN-32658-6, HN-02317-9	FJ765913, FJ222564, FJ224132, FJ765943, FJ907386, FJ940746, FJ976870, GQ160999, GQ180305	Histogenetics
A*03:01:12	A*030112	A3	—	HN-61746-5, HN-04652-4	FJ392138, FJ392139	Histogenetics
A*03:01:13	A*030113	A3	—	HN-45046-2	FJ222572	Histogenetics
A*03:01:14	A*030114	A3	—	HN-59324-6, HN-70577-3, HN-40419-7, HN-42295-8, HN-B-175649, HN-19428-2, HN-43533-9, HN-47828-5, 4VL071332, HN-48416-3, HN-74235-3, HN-22453-9, HN-16576-0, HN-42799-9, HN-94231-6, HN-3461618, HN-32569-1, HN-52644-0, HN-44681-1, HN-05098-4, HN-52525-8, HN-33985-5, HN-58033-4	FJ222573, FJ224177, FJ224193, FJ224195, FJ224197, FJ619445, FJ976744, FJ976868, FN555233, GQ160969, GQ160996, GQ161022, GQ180290, GQ180298, GQ180324, GQ180335, GQ240356, GQ240541, GQ401175, GQ449621, GQ449622, GQ859512, GQ994053	Histogenetics, A Jakubauskas^b^
A*03:01:15	A*030115	A3	—	HN-01935-0, HN-96260-4, HN-49798-4, HN-08400-6	FJ224209, FJ224231, FJ976755, GQ900560	Histogenetics
A*03:01:16	A*030116	A3	—	HN-4933367	FJ392137	Histogenetics
A*03:01:17	A*030117	A3	—	HN-29269-1	FJ619442	Histogenetics
A*03:02	A*0302	A3	A3.2	E1B2, R69772, CL183, SZ-62	U56434, U56435, AF217561, FJ969862	HY Zou^b^
A*03:03N	A*0303N	Null	A3blank	MMK	L77702	
A*03:04	A*0304	A3	—	CTM-2983694	AF015930	
A*03:05	A*0305	A3	A*03011V	GN00262, GN00309, 99-2197, CS, 34507	AF135546, AF135547, AF173877, AF173878, AF190718, AF190719, AJ252283, AJ252284, AJ252285, AJ401085, AJ401086, AJ401087	
A*03:06	A*0306	—	A*03011V	GN00341	AF226842, AF226843	
A*03:07	A*0307	—	A*03011New	NM5A488, NT01005	AF268399, AF268400, EU924806	CK Hurley^b^
A*03:08	A*0308	—	A*03011v	GN00375, NT00777	AF288047, AF288048, EU484053	CK Hurley^b^
A*03:09	A*0309	—	—	BY00016, NT01104	AF372049, AF372050, GQ251365	CK Hurley^b^
A*03:10	A*0310	—	—	LB60240	AJ506200, AJ506193	
A*03:11N	A*0311N	Null	A*03DK Null	10913246	AJ532607	
A*03:12	A*0312	—	—	80302	AY310505	
A*03:13	A*0313	—	—	9043860	AY354905, AY354906	
A*03:14	A*0314	—	—	9081043	AY368497, AY368498	
A*03:15	A*0315	—	—	Z-00014154	AJ876374	(70)
A*03:16	A*0316	—	—	NT00517	AY874081, AY874082	(33)
A*03:17	A*0317	—	—	29240S	AY619990	S Adams
A*03:18	A*0318	—	A*01ERN0604	MHHZ-00008720	AJ871405	R Blasczyk
A*03:19	A*0319	—	—	2005062436	DQ139312, DQ139313	(71)
A*03:20	A*0320	A3	—	CHRJM	AM056023	A Dormoy
A*03:21N	A*0321N	Null	—	PHNI332212AN	AM087469	AM Little
A*03:22	A*0322	—	A*03MVE1105	I-126320, NT00717	AM161452, EF422079	R Blasczyk, CK Hurley^b^
A*03:23	A*0323	—	—	BY00096	DQ465612	(57)
A*03:24	A*0324	A3	—	T05-915	DQ403156, DQ403157	R Patel
A*03:25	A*0325	—	—	NT00682	DQ987874	(33)
A*03:26	A*0326	—	—	CTM-0695183	EF158451	(72)
A*03:27	A*0327	—	A*03MVE0906	MHHN-189452	AM407885	R Blasczyk
A*03:28	A*0328	—	—	MHHN-266956	AM493250	R Blasczyk
A*03:29	A*0329	—	—	BY00153	EF484942	(36)
A*03:30	A*0330	—	—	Xian30109	EF602747	M Liu
A*03:31	A*0331	—	—	MHHZ-00023725	AM748070	R Blasczyk
A*03:32	A*0332	—	—	BY00196	EU029780	(36)
A*03:33	A*0333	—	—	MHHN-574610	AM778452	R Blasczyk
A*03:34	A*0334	—	—	MHHN-563903	AM778555	R Blasczyk
A*03:35	A*0335	—	—	107028	AM779474	J Enczmann
A*03:36N	A*0336N	Null	—	FAESDid	AM413001, AM849481	A Dormoy
A*03:37	A*0337	—	—	LUMC-A29	AM774358	(64)
A*03:38	A*0338	—	—	NT00737	EU146153	CK Hurley
A*03:39	A*0339	—	—	CTM-1698446	EU305401	(73)
A*03:40	A*0340	—	—	7216728	EU445574	A Vigh
A*03:41	A*0341	—	—	NT00997	EU872416	CK Hurley
A*03:42	A*0342	—	—	DIOUF	AM712914	(74)
A*03:43	A*0343	—	—	163818	FM210536	(75)
A*03:44	A*0344	—	—	CDC09292008	FJ227108	S Cordovado
A*03:45	A*0345	A3	—	175582	FM223686	(75)
A*03:46	A*0346	—	—	BY00374	FJ464348	CK Hurley
A*03:47	A*0347	—	—	HN-19518-9, NT01019, HN-709721	FJ224145, FJ797383, FJ489878	Histogenetics, CK Hurley^b^
A*03:48	A*0348	—	—	HN-32993-0, HN-43969-2, HN-40494-9, HN-52782-1, HN-21690-2, HN-65267-9	FJ358629, FJ875685, FJ976753, GQ161002, GQ180293, GQ401183	Histogenetics
A*03:49	A*0349	—	—	HN-11137-1, HN-22431-5, HN-17777-8, HN-8933692, HN-69245-4	FJ358637, FJ224212, FJ976760, FJ392157, GQ900559	Histogenetics
A*03:50	A*0350	—	—	NT01013, HN-703138, BY00519, HN-61931-3, HN-98248-5, HN-23778-0	FJ797389, FJ792520, GQ867213, GQ161001, GQ240545, GQ914780	CK Hurley, Histogenetics^b^
A*03:51	A*0351	—	—	NT01015, HN-763009, HN-4096256	FJ797387, FJ619459, FJ976747	CK Hurley, Histogenetics^b^
A*03:52	A*0352	—	—	HN-35736-7	FJ222563	Histogenetics
A*03:53	A*0353	—	—	HN-09644-4, HN-09305-2, HN-08709-0, HN-19631-3	FJ224131, FJ619438, FJ358635, GQ994043	Histogenetics
A*03:54	A*0354	—	—	HN-94347-1	FJ224230	Histogenetics
A*03:55	A*0355	—	—	REC1109C14	GQ250940	MdG Bicalho
A*03:56	A*0356	—	—	HN-14964-2	FJ222567	Histogenetics
A*03:57	A*0357	—	—	HN-70922-0, P-661594	FJ224172, FN430729	Histogenetics, R Blasczyk^b^
A*03:58	A*0358	—	—	HN-29857-0	FJ224234	Histogenetics
A*03:59	A*0359	—	—	HN-187805	FJ224241	Histogenetics
A*03:60	A*0360	—	—	LUMC-A60	FN544086	JDH Anholts
A*03:61	A*0361	—	—	HN-7280180	FJ392145	Histogenetics
A*03:62	A*0362	—	—	233509, HN-93337-4	FN422387, GQ160990	T Lebedeva, Histogenetics^b^
A*03:63	A*0363	—	—	HN-50847-0, HN-52450-1	FJ619456, FJ640590	Histogenetics
A*03:64	A*0364	—	—	HN-0664550	FJ619462	Histogenetics
A*03:65	A*0365	—	—	HN-0300840486922	FJ765936	Histogenetics
A*03:66	A*0366	—	—	HN-47860-9	FJ765940	Histogenetics
A*03:67	A*0367	—	—	HN-64366-4	FJ875540	Histogenetics
A*03:68N	A*0368N	Null	—	HN-76532-7	FJ875548	Histogenetics
A*03:69N	A*0369N	Null		HN-89228-9, HN-89227-1	FJ875549, FJ875550	Histogenetics
A*03:70	A*0370	—	—	BY00566	GU138060	CK Hurley
A*03:71	A*0371	—	—	HT-59	GU169071	M Yu
A*03:72	A*0372	—	—	JMDP01K043	AB535153	K Tadokoro
A*03:73	A*0373	—	—	BY00578	GU256011	CK Hurley
A*03:74	A*0374	—	—	BY00575	GU256014	CK Hurley
A*11:01:01	A*110101	A11	A11E, A11.1, A11	CJO-A, K.LIE, MMU, YMU, THA-DCH412, THA-DCH926, THA-DCH1093, SJK-YS, B9910, 02008981	M16007, M16008, X13111, D16841, AF030899, AF030900, AF030901, AF030902, AF030897, AF030898, AY786587, EU445473, GQ996938	(30)^b^, Y Xu^b^
A*11:01:02	A*110102	A11	A*1101new	UCLA201	AJ238608, AJ238609, AJ238610	
A*11:01:03	A*110103	A11	—	7A502	AY289107, AY289108	
A*11:01:04	A*110104	A11	—	NHL, NWW, SRS, SBW	AY786585	
A*11:01:05	A*110105	A11	—	TBC54882	AB213377	M Satake
A*11:01:06	A*110106	A11	—	B11798	EF092416	(76)
A*11:01:07	A*110107	A11	—	TBC63500	AB292218	M Satake
A*11:01:08	A*110108	A11	—	AKB32391	AM849045	S Schwab
A*11:01:09	A*110109	A11	—	73043	AM931011	J Enczmann
A*11:01:10	A*110110	A11	—	BJ50	EU325939	Z Zhang
A*11:01:11	A*110111	A11	—	HN-9813716, HN-4725344, 337084	EU882865, FJ765883, FN422385	Histogenetics, T Lebedeva^b^
A*11:01:12	A*110112	A11	—	HN-1643803	FJ224242	Histogenetics
A*11:01:13	A*110113	A11	—	HN-92780-8	FJ358631	Histogenetics
A*11:01:14	A*110114	A11	—	HN-63517-7	FJ224221	Histogenetics
A*11:01:15	A*110115	A11	—	HN-770624, HN-2772718, HN-679700	FJ489877, FJ765892, FJ792523	Histogenetics
A*11:01:16	A*110116	A11	—	HN-98268-4	FJ765938	Histogenetics
A*11:02:01	A*110201	A11	A11K, A11.2	K.LIE, KOK, CTA, THA-DCH538, THA-DCH639, SZ-56	X13112, D16842, AF030903, AF030904, AF030905, AF030906, FJ969874	HY Zou^b^
A*11:02:02	A*110202	A11	—	NT00640	DQ354440, DQ354441	(77)
A*11:02:03	A*110203	A11	—	SZ-11	EU492459	(78)
A*11:03	A*1103	A11	—	AMAD	X91399, Y17224	
A*11:04	A*1104	A11	87A	HM, I65, 87A, THA-DCH7672, THA-DCH7673	U50574, U59701, U59702, U88250, AF017309, AF030907, AF030908, AF030909, AF030910	
A*11:05	A*1105	A11	—	KH, GN00302, HOATWAY	Y15223, AF147454, AF147455, AJ306733	
A*11:06	A*1106	—	A*1101V	GN00259	AF135540, AF135541	
A*11:07	A*1107	A11	A*11	CMC1	AF165065	
A*11:08	A*1108	A11	A11nou	VPH-IM0002135	AF284443	
A*11:09	A*1109	A11	—	9315466, VTIS143208	AF260828, AF260829, DQ417106, DQ417107	BD Tait^b^
A*11:10	A*1110	A11	A11v	VTIS38035, BY00366	AF329874, AF329875, FJ427298	CK Hurley^b^
A*11:11	A*1111	—	—	2001-26-469	AF440108, AF440109	
A*11:12	A*1112	A11	—	103201, 103195, 106843, 106844	AF439511	
A*11:13	A*1113	A11	A11v	B5997, JMDP01K006	AB073216, AB073217, AB430311	K Tadokoro^b^
A*11:14	A*1114	A11	—	SZ-2	AY134743	
A*11:15:01	A*111501	A11	—	NEQ_ED03/03, 36777	AJ581838, AJ581839, AJ634004	
A*11:15:02	A*111502	A11	A*1128	BY00103	DQ473291	(33)
A*11:16	A*1116	—	—	9727926	AY368499, AY368500	
A*11:17	A*1117	—	—	NT00503, BY00379	AY428803, AY428804, FJ464343	CK Hurley^b^
A*11:18	A*1118	—	—	28003	AJ697942, AJ697943	
A*11:19	A*1119	A11	A*1101V1	TBC8111, NT00745	AB185092, EU185512	CK Hurley^b^
A*11:20	A*1120	—	—	2004110364, NT01054	AY839183, AY839184, FJ976711	CK Hurley^b^
A*11:21N	A*1121N	Null	—	TBC50620	AB196426	
A*11:22	A*1122	—	—	NT00528	AY867794, AY867795	(33)
A*11:23	A*1123	—	—	NT00516, NT00670	AY903431, AY903432, DQ648005	(33)
A*11:24:01	A*112401	—	—	PSP9113	AM114414	K Lebeer
A*11:24:02	A*112402	—	—	JMDP01K007	AB430468	K Tadokoro
A*11:25	A*1125	A11	—	PABR19129AN	AM183562	AM Little
A*11:26	A*1126	—	—	KN9409963	DQ343835	(79)
A*11:27	A*1127	—	—	BY00099	DQ465608	(57)
A*11:29	A*1129	—	—	051407cb, 06d05591, 07d00393	AM261865, AM746507	(80), JDH Anholts^b^
A*11:30	A*1130	—	A*11MVE1106	MHHAKB-206494, HN-75401-4	AM412219, FJ765881	R Blasczyk, Histogenetics^b^
A*11:31	A*1131	—	—	227719	EF465413	(81)
A*11:32	A*1132	—	—	RA1283	EF626806	M Yu
A*11:33	A*1133	—	—	JMDP36K002	AB434757	K Tadokoro
A*11:34	A*1134	—	—	BY00325	EU716062	CK Hurley
A*11:35	A*1135	—	—	HistoCB40216	EU680956	(82)
A*11:36	A*1136	—	—	B23144	EU741677	(83)
A*11:37	A*1137	—	—	7219762	EU445573	A Vigh
A*11:38	A*1138	—	—	B25577	FJ355925	(83)
A*11:39	A*1139	—	—	BY00359	FJ358712	CK Hurley
A*11:40	A*1140	—	—	BY00385	FJ517147	CK Hurley
A*11:41	A*1141	—	—	ERCB-FO-01/00183-379CB08	FN178525	(84)
A*11:42	A*1142	—	—	NT01021	FJ797381	CK Hurley
A*11:43	A*1143	—	—	K19692	AB485772	E Maruya
A*11:44	A*1144	—	—	BY00467, HN-0852668	FJ976702, FJ765937	CK Hurley, Histogenetics^b^
A*11:45	A*1145	—	—	HN-68740-0	FJ224160	Histogenetics
A*11:46	A*1146	—	—	HN-17281-5, HN36629-3	FJ224220, GQ180288	Histogenetics
A*11:47	A*1147	—	—	HN-5915-1, HN-6711-2	FJ222571, FJ224175	Histogenetics
A*11:48	A*1148	A11	—	RAVE676425AN, HN-001504	FN433878, GQ240534	SGE Marsh, Histogenetics^b^
A*11:49	A*1149	—	—	P4081	GQ478195	L Yan
A*11:50	A*1150	—	—	LIWE52997AN	FN435324	SGE Marsh
A*11:51	A*1151	—	—	HN-1615	FJ358630	Histogenetics
A*11:52	A*1152	—	—	BY00518	GQ867212	CK Hurley
A*11:53	A*1153	—	—	7550800636	GQ996939	Y Xu
A*11:54	A*1154	—	—	HN-53533-5	FJ358620	Histogenetics
A*11:55	A*1155	—	—	SZ-38	GU074394	HY Zou
A*23:01:01	A*230101	A23(9)	—	SHJO, ELON, B6001	M64742, L76288, EU445474	(30)
A*23:01:02	A*230102	A23(9)	—	HN-46329-8, HN-25543-9, HN-28515-4, HN-24793-3, HN-07940-9, HN-23088-9, HN-36828-9, HN-76176-8, HN-57136-6, HN-52147-3, HN-135361, HN-98551-2, HN-03764-4, HN-06561-7, HN-40389-1, HN-57799-4	FJ224143, FJ600646, FJ600647, FJ224186, FJ619403, FJ792526, FJ600633, FJ619408, FJ619415, FJ640589, FJ907383, FJ952580, GQ245714, GQ449619, GQ994050, GQ994054	Histogenetics
A*23:02	A*2302	—	A*2301V	GN00274	AF137079, AF137080	
A*23:03:01	A*230301	—	A*2301 variant	GN00250	AF102571, AF102572	
A*23:03:02	A*230302	—	—	NT00754	EU275160	(85)
A*23:04	A*2304	A23(9)^c^	A*2301V	GN00263, NT01103	AF135548, AF135549, GQ251364	CK Hurley^b^
A*23:05	A*2305	—	A*2301New	GN00284, NM5A405	AF140859, AF140860, AF255718, AF255719	
A*23:06	A*2306	—	A*2301New	GM14672	AJ271340	
A*23:07N	A*2307N	Null	A*23MATSi	MATSi	AJ306634	
A*23:08N	A*2308N	Null	—	SH38	AY028848, AY028849, AY028850	
A*23:09	A*2309	—	—	MAWE0816AN	AJ426561	
A*23:10	A*2310	—	—	7A580	AY288929, AY288930	
A*23:11N	A*2311N	Null	—	2003-4363	AY429572, AY429573	
A*23:12	A*2312	-A23(9)	—	UKNEQAS_H&I_411/2003, 41869	AJ604535, AJ604536, AJ619767	
A*23:13	A*2313	—	A*23MVE0705	MHHZ-00017616	AM072344	(86)
A*23:14	A*2314	—	—	20020885CB	AB234291	H Inoko
A*23:15	A*2315	—	—	BY00152, BY00459	EF484941, FJ842970	(36), CK Hurley^b^
A*23:16	A*2316	—	—	D24054	AM493902	O Avinens
A*23:17	A*2317	—	—	LUMC-A33	AM904553	(64)
A*23:18	A*2318	A23(9)	—	F438	EU253478	(87)
A*23:19Q	A*2319Q	—	—	ALX0010, 95230, HN-7579920	DQ145282, DQ145283, EF656476, FJ875682	E Ball, MS Leffell, Histogenetics^b^
A*23:20	A*2320	—	—	BJ056	FJ518827	Z Zhang
A*23:21	A*2321	—	—	NT01022, HN-756995	FJ797380, FJ619458	CK Hurley, Histogenetics^b^
A*23:22	A*2322	—	—	HN-55909-8	FJ619418	Histogenetics
A*23:23	A*2323	—	—	HN-93402-4	FJ619391	Histogenetics
A*23:24	A*2324	—	—	HN-61977-5, HN02796-4	FJ619400, FJ619417	Histogenetics
A*23:25	A*2325	—	—	HN-27733-7	FJ619412	Histogenetics
A*24:02:01:01	A*24020101	A24(9)	A24, A2402	SHJO, 32/37, KRC032, KRC110, THA-DCH538, B9564, 7550800206	M64740, L47206, Z72423, AF030911, AF030912, EU445475, GQ996940	(30)^b^, Y Xu^b^
A*24:02:01:02L	A*24020102L	Low A24(9)	A2402LOW, APET, A24L-LACC	6319, PAn, PMa, Pmi, LACC, 15706, CTM6689449	L76291, Z72422, Z97370, AJ870438, DQ188809	JL Vicario^b^
A*24:02:02	A*240202	A24(9)	—	NM426, NT00994	AF101160, AF101161, EU872413	CK Hurley^b^
A*24:02:03	A*240203	A24(9)	—	KBM-2	AY121128	
A*24:02:04	A*240204	A24(9)	—	A3676, 40148S, CS00014	AY128674, AY619992, GQ292546	S Adams^b^, K Cao^b^
A*24:02:05	A*240205	A24(9)	—	9490723	AY354909, AY354910	
A*24:02:06	A*240206	A24(9)	A*2402V3	TBC45776	AB187124	
A*24:02:07	A*240207	A24(9)	—	28004	AJ697944, AJ697945, AJ878879	EM van den Berg Loonen
A*24:02:08	A*240208	A24(9)	—	BY00060	DQ105564, DQ105565	(88)
A*24:02:09	A*240209	A24(9)	—	2004150JCBN	AB234290	H Inoko
A*24:02:10	A*240210	A24(9)	—	CTM2097181	DQ188808	JL Vicario
A*24:02:11	A*240211	A24(9)	—	2005101727	DQ333354, DQ333355	(89)
A*24:02:12	A*240212	A24(9)	—	BJ004	DQ905958	(90)
A*24:02:13	A*240213	A24(9)	—	91339	EF061083	MS Leffell
A*24:02:14	A*240214	A24(9)	—	R35770	AM497719	(91)
A*24:02:15	A*240215	A24(9)	—	2007-4627	AM922284	(92)
A*24:02:16	A*240216	A24(9)	—	JMDP01K008, HN-4337452	AB434096, FJ976745	K Tadokoro, Histogenetics^b^
A*24:02:17	A*240217	A24(9)	BJ058	BJ58	FJ428239	N Liu
A*24:02:18	A*240218	A24(9)	—	HN-59221-9	FJ619404	Histogenetics
A*24:02:19	A*240219	A24(9)	—	HN-78059-1, HN-86540-2, HN-91110-0, HN-56961-5, HN-07579-9, HN-78007-2, HN-4246661, HN-34236-8, HN-19680-2, HN-7003269, HN-17031-3, HN-77073-5, HN-22305-5, HN-07962-6, HN-14637-8, HN-97781-6, HN-29993-5	FJ619421, FJ224169, FJ224214, FJ224233, FJ224243, FJ619402, FJ619464, FJ619393, FJ619420, FJ619471, FJ797948, FJ875543, GQ149259, GQ149288, GQ240537, GQ240544, GQ859518	Histogenetics
A*24:02:20	A*240220	A24(9)	—	P3749	FJ940681	L Yan
A*24:02:21	A*240221	A24(9)	—	HN-03364-8	FJ224179	Histogenetics
A*24:02:22	A*240222	A24(9)	—	HN-64867-5	FJ358625	Histogenetics
A*24:02:23	A*240223	A24(9)	—	HN-52496-7, HN52246-6, HN52243-3, HN-52275-5	FJ594712, FJ600651, FJ619469, GQ199690	Histogenetics
A*24:02:24	A*240224	A24(9)	—	HN-14585-2	FJ619396	Histogenetics
A*24:02:25	A*240225	A24(9)	—	HN-71176-2	FJ619407	Histogenetics
A*24:02:26	A*240226	A24(9)	—	HN-38289-6	FJ619410	Histogenetics
A*24:02:27	A*240227	A24(9)	—	HN-8436563, HN-00138-7	FJ619476, FJ907388	Histogenetics
A*24:02:28	A*240228	A24(9)	—	HN-7940633	FJ600640	Histogenetics
A*24:02:29	A*240229	A24(9)	—	HN-5429-0, HN-4519810, HN-29841-4	FJ619411, FJ619463, FJ619474	Histogenetics
A*24:02:30	A*240230	A24(9)	—	HN-4999749, HN-3333619	FJ619465, FJ875689	Histogenetics
A*24:03:01	A*240301	A2403	A9.3	APA, KPE, THA-DCH412, THA-DCH8151, THA-DCH8152, SZ-64	M64741, AF030913, AF030914, AF030915, AF030916, AF030917, AF030918, FJ969867	HY Zou^b^
A*24:03:02	A*240302	A2403	A*2403 Variant	GN00247	AF102565, AF102566	
A*24:04	A*2404	A24(9)	A24AK,	ITOU, KJRAID5	D26550, L43532, L43533	
A*24:05	A*2405	A24(9)	—	DST, FST, NT00995, JMDP01K069	X82161, X82189, EU872414, AB536870	CK Hurley^b^, K Tadokoro^b^
A*24:06	A*2406	A24(9)	A*24YM	YM29	U18987, U19733	
A*24:07	A*2407	A24(9)	A#46	PICH, A#46, K92068, THA-DCH507, THA-DCH522, THA-DCH1109, THA-DCH5342, SZ-66	U25971, U36914, L43530, L43531, AF030921, AF030922, AF030919, AF030920, AF030923, AF030924, AF030925, AF030926, FJ969864	HY Zou^b^
A*24:08	A*2408	A24(9)	A*9HH	K62098, HIRH	L43528, L43529, D83516	
A*24:09N	A*2409N	Null	A24Null	SUS-NF, WAG	L47231, AJ251621	
A*24:10	A*2410	A2403^c^	A*24JV	JV1458, KM315, CH121, THA-DCH611, THA-DCH639, THA-DCH1109, JMDP36K023, SZ-63	U37110, U37111, U59699, U59700, Y10695, AF030927, AF030928, AF030929, AF030930, AF030931, AF030932, AB436621, FJ969865	K Tadokoro^b^, HY Zou^b^
A*24:11N	A*2411N	Null	A*24LM	LUME, 38602	L76289, AJ966741	J Rowlands^b^
A*24:13:01	A*241301	A24(9)	A*24YM2	YM81	U37112, U37113	
A*24:13:02	A*241302	A24(9)	—	BY00155, BY00167, HN-73720-6	EF484944, EF563136, FJ976749	CK Hurley^b^, Histogenetics^b^
A*24:14	A*2414	A24(9)	A*24SA	SBD6380, NT00733	U37114, U37115, EF563142	CK Hurley^b^
A*24:15	A*2415	—	—	NM3469	AF042666, AF042667	
A*24:17	A*2417	A24(9)^c^	A*2402v, A*VB	NDS-NH, VB-ARCBS, 0234	AF067436, AF067437, AF117764, AF117765, AJ239035, AJ239036	
A*24:18	A*2418	A24(9)	A*2403v, A24x3	3362	AF065401, AF065402	
A*24:19	A*2419	A9	—	HP-CV	Y17292, Y17291	
A*24:20	A*2420	A24(9)^c^	—	SW36, 21833843, JCBB26794, TBC54975, SZ-57	Y16948, Y16949, AF190716, AF190717, AB032596, AB218626, FJ969866	M Satake^b^, HY Zou^b^
A*24:21	A*2421	A24(9)	A*24Var	DHL, JSL, 12106291	AF106688, AF106689, AJ717300	
A*24:22	A*2422	A9	A*2403New, A9v	CL153, GN00272, CS00013	AF116214, AF137081, AF137082, GQ292545	K Cao^b^
A*24:23	A*2423	A2403	A*24021New, A24v(9)	EA31, 26586	AF128537, AF128538, AJ278667	
A*24:24	A*2424	—	—	GN00275	AF140723, AF140724	
A*24:25	A*2425	—	—	10296952, NM5A251, JMDP01K038	AF190708, AF190709, AF255716, AF255717, AB436158	K Tadokoro^b^
A*24:26	A*2426	—	—	12318945, BY00562	AF190710, AF190711, AF190712, GU138064	CK Hurley^b^
A*24:27	A*2427	A24(9)^c^	A*24Mall	MALL	AJ271626	
A*24:28	A*2428	A24(9)	—	GN00359, TBC60703	AF266519, AF266520, AB261694	M Satake^b^
A*24:29	A*2429	—	A*2402V	GN00379, 443/10	AF291843, AF291844, AJ537477, AJ537478, AJ537479, AJ537480	
A*24:30	A*2430	—	A*2402V	GN00380, JMDP01K010	AF291841, AF291842, AB434098	K Tadokoro^b^
A*24:31	A*2431	—	—	2000-35-513	AF298583, AF298584	
A*24:32	A*2432	—	—	GN00390, 07-S-0025#0100	AF359393, AF359394, AY038075	
A*24:33	A*2433	A2403	—	17933/00, 17670/00	AF363678, AF363679, AF363680	
A*24:34	A*2434	—	—	2000-182-2100	AF443283, AF443284	
A*24:35	A*2435	—	—	PR45	AY045731, AY045732	
A*24:36N	A*2436N	Null	—	022659718, 45188	AF486832, AJ9666740	J Rowlands^b^
A*24:37	A*2437	A24(9)	—	TER1132	AJ506181, AJ507149, AJ507150	
A*24:38	A*2438	—	—	R210304	AJ507645, AJ507646	
A*24:39	A*2439	—	—	FHNG, FHPK	AY028635, AY028636, AY028637	
A*24:40N	A*2440N	Null	—	5096845	AY392503, AY390524, AY390525	
A*24:41	A*2441	—	—	NT00501	AY396025, AY396026	
A*24:42	A*2442	—	—	142654	AJ626847	
A*24:43	A*2443	A24(9)	A*2402V2	TBC01P39382, BY00205	AB180087, AB180088, AB180089, EU029774	CK Hurley^b^
A*24:44	A*2444	—	—	1198/01	AJ537481, AJ537482, AJ537483, AJ537484	
A*24:45N	A*2445N	Null	A*2402N1	TBC372494	AB183459	
A*24:46	A*2446	—	A*2402V4	TBC46049	AB185093	
A*24:47	A*2447	—	—	28005	AJ697946, AJ697947, AJ697948	
A*24:48N	A*2448N	Null	—	43623	AJ867235	
A*24:49	A*2449	—	—	TBC49875	AB196429	
A*24:50	A*2450	—	—	NT00527, BY00179	AY870643, AY870644, EF591031	(33), CK Hurley^b^
A*24:51	A*2451	—	—	14721	AY904343	(93)
A*24:52	A*2452	—	—	TBC53522, BY00115	AB211247, DQ535036	M Satake, CK Hurley^b^
A*24:53	A*2453	—	—	38200S, CS00012	AY619991, GQ281056	S Adams, K Cao^b^
A*24;54	A*2454	—	—	28672	AJ878876	EM van den Berg Loonen
A*24:55	A*2455	—	—	NT00620	DQ205711, DQ205712	(33)
A*24:56	A*2456	—	—	NT00613, NT00657	DQ205705, DQ205706, DQ455022	(33)
A*24:57	A*2457	—	—	NT00614, NT00642	DQ205707, DQ205708, DQ354434, DQ354435	(33)
A*24:58	A*2458	A24(9)	—	301009, BY00097	AM157679, DQ465609	(94), CK Hurley^b^
A*24:59	A*2459	—	—	B8299, BY00117	DQ313255, DQ313256, DQ313257, DQ782332	(95), CK Hurley^b^
A*24:60N	A*2460N	Null	—	TBC56401	AB247152	M Satake
A*24:61	A*2461	—	—	DUB330	AJ616915	C Dunne
A*24:62	A*2462	—	—	K05528, JMDP01K011	AB249885, AB434492	E Maruya, K Tadokoro^b^
A*24:63	A*2463	—	—	TBC5990	AB253623	M Satake
A*24:64	A*2464	—	—	BM05-322, BM05-316	DQ523225	(96)
A*24:65	A*2465	—	—	BY00155, BY00167	EF484944, EF563136	CK Hurley
A*24:66	A*2466	—	—	BY00120	DQ782329	CK Hurley
A*24:67	A*2467	—	—	166914	AM408111	(97)
A*24:68	A*2468	—	—	P1893	EF101926	(98)
A*24:69	A*2469	—	A*24MVE1106	MHHN-206180	AM412220	R Blasczyk
A*24:70	A*2470	—	A*24MHA1106	MHHAKB-206446	AM411623	R Blasczyk
A*24:71	A*2471	—	—	HE-2215662	EF200124	(99)
A*24:72	A*2472	—	—	MHHN-266927, BY00156	AM493252, EF484945	R Blasczyk, (36)
A*24:73	A*2473	—	—	MHHAKB-332722	AM493253	R Blasczyk
A*24:74	A*2474	—	—	2006092224	EF447432	(100)
A*24:75	A*2475	—	—	2006092274	EF447433	(101)
A*24:76	A*2476	A24(9)	—	CY1095078	AM491587	A Dormoy
A*24:77	A*2477	—	—	BY00177	EF591029	(36)
A*24:78	A*2478	A24(9)	—	2004307seq	AB303946	H Inoko
A*24:79	A*2479	—	—	87044	EF656474	MS Leffell
A*24:80	A*2480	—	—	LUMC-A23	AM746483	(64)
A*24:81	A*2481	—	—	BY00189, BY00198, HN-3720876	EU029786, EU882864	(36), Histogenetics^b^
A*24:82	A*2482	—	—	BY00197	EU029779	(36)
A*24:83N	A*2483N	Null	—	DAVEm	AM849480	A Dormoy
A*24:84N	A*2484N	Null	—	6500234	AM849046	S Schwab
A*24:85	A*2485	A24(9)	—	LZ-3, HN-3493876	EU352655, FJ875681	(102), Histogenetics^b^
A*24:86N	A*2486N	Null	—	HZB10788	EF062307	(103)
A*24:87	A*2487	—	—	JMDP01K009, HN-80015-1	AB434097, FJ875536	K Tadokoro, Histogenetics^b^
A*24:88	A*2488	—	—	JMDP36K003	AB434758	K Tadokoro
A*24:89	A*2489	—	—	071SX132	EU643640	(104)
A*24:90N	A*2490N	Null	—	BY00331, HN-1869218, BY00525	EU716068, FJ887803, GQ867219	CK Hurley, Histogenetics^b^
A*24:91	A*2491	—	—	CB5943	EU741678	(69)
A*24:92	A*2492	—	—	BJ55	EU862819	Z Zhang
A*24:93	A*2493	—	—	SENE, NT01016, HN-684098	AM493679, FJ797386, FJ600636	(74), CK Hurley^b^, Histogenetics^b^
A*24:94	A*2494	—	—	BY00350	FJ174580	CK Hurley
A*24:95	A*2495	—	—	BY00363, BY00364, 100482, HN-5899310, HN6086149, HN-2748, HN-7393785, HN1442543	FJ358708, FJ358709, FJ619520, FJ358621, FJ538270, FJ619466, FJ619475, FJ797947	CK Hurley, MS Leffell^b^, Histogenetics^b^
A*24:96	A*2496	—	BJj243	BJ243	FJ428230	X Shan
A*24:97	A*2497	—	—	BY00373, HN-3142614	FJ464349, FJ619473	CK Hurley, Histogenetics^b^
A*24:98	A*2498	—	—	p3531	FJ560745	(69)
A*24:99	A*2499	—	—	C4563	AB441825	C Horie
A*25:01:01	A*250101	A25(10)	—	BM92	M32321	
A*25:01:02	A*250102	A25(10)	—	7A502	AY297162, AY297163	
A*25:02	A*2502	A25(10)^c^	A66var	M54672, TW	X97802, AJ238524	
A*25:03	A*2503	—	A*2501V	GN00273, GN00301	AF137075, AF137076, AF148897, AF148898	
A*25:04	A*2504	—	—	BY0019	AY042682, AY042683	
A*25:05	A*2505	—	—	DN435.5	DQ645957	M Yu
A*25:06	A*2506	—	—	92313	DQ902552	MS Leffell
A*25:07	A*2507	—	—	CDC09272008	FJ227106	S Cordovado
A*25:08	A*2508	—	—	BY00377, HN-51803-6	FJ464345, FJ358628	CK Hurley, Histogenetics^b^
A*25:09	A*2509	—	—	HN-45030-2	FJ224124	Histogenetics
A*25:10	A*2510	—	—	HN-58646-0	FJ600643	Histogenetics
A*25:11	A*2511	—	—	BY00577	GU256012	CK Hurley
A*26:01:01	A*260101	A26(10)	A26.1, A26.3	GM637, O2BN5, MGAR, N.M., MIY-2, MIY-3, QBL, B8596	M24095, U03697, D16843, D32130, D32131, AL845454, EU445476	(105)^b^, (30)^b^
A*26:01:02	A*260102	A26(10)	—	2005031636, BY00068	DQ003050, DQ003051, DQ123855, DQ123856	(106), CK Hurley^b^
A*26:01:03	A*260103	A26(10)	—	TBC53385	AB211246	M Satake
A*26:01:04	A*260104	A26(10)	—	NT00587	DQ086782, DQ086783	(107)
A*26:01:05	A*260105	A26(10)	—	TBC63337	AB292217	M Satake
A*26:01:06	A*260106	A26(10)	—	CambsA26	AM922174	R Goodman
A*26:01:07	A*260107	A26(10)	—	LUMC-A56	FM945329	JDH Anholts
A*26:01:08	A*260108	A26(10)	—	QianhefenA26	FJ810058	J He
A*26:01:09	A*260109	A26(10)	—	HN-77427-2	FJ224137	Histogenetics
A*26:01:10	A*260110	A26(10)	—	HN-32502-5	FJ224216	Histogenetics
A*26:01:11	A*260111	A26(10)	—	HN-49017-7	FJ224222	Histogenetics
A*26:01:12	A*260112	A26(10)	—	346271	FN422390	T Lebedeva
A*26:01:13	A*260113	A26(10)	—	HN-7541	FJ224228	Histogenetics
A*26:02	A*2602	A26(10)	A26.2, A26.1	KT14, Y.I., E.K.	M98453, D14350	
A*26:03	A*2603	A26(10)	A26.4	T.M., S.M., MIY-1	D14351, D32129	
A*26:04	A*2604	A26(10)	A10SA	Y.S.	D14354	
A*26:05	A*2605	A26(10)	A26KY	SAJ022, K91089, K93022	D50068, L43536, L43537	
A*26:06	A*2606	A26(10)	—	KHB102, TBC47774	L43534, L43535, AB282890	M Satake^b^
A*26:07:01	A*260701	A26(10)	A26mic	MIC-ND	L48341	
A*26:07:02	A*260702	A26(10)	—	AMA, LAC	AJ290394, AJ580771	(108)
A*26:08	A*2608	A26(10)	A26RMH, A*26new-66A	MI108, W652D, M.McL, 66A	U45480, U52429, X99733, U43334, AF017310	
A*26:09	A*2609	A26(10)^c^	—	GN00158	U90242, U90243	
A*26:10	A*2610	A10	—	034-SEA-HK	AF001553, AF001554	
A*26:11N	A*2611N	Null	A26Null	JBO13900	AB005048	
A*26:12	A*2612	—	A*2601V	NM1183, CS3, GN00249	AF042186, AF042187, AF065486, AF065487	
A*26:13	A*2613	—	A*2601V	GN00271	AF139766, AF139767	
A*26:14	A*2614	—	A*MJUL	MJUL	AF194529, AF194530	
A*26:15	A*2615	A10^c^	A*26FONT	FONT, FRED, FRED(-1), 22663, 6A29	AJ271225, AJ291695, AJ291696, AY045729, AY045730	
A*26:16	A*2616	—	—	2000-7-951	AF303952, AF303953	
A*26:17	A*2617	—	—	GN0384, JMDP01K012	AF310142, AF310143, AB434691	K Tadokoro^b^
A*26:18	A*2618	—	—	GN00399, BY00360	AY050205, AY050206, FJ358713	CK Hurley^b^
A*26:19	A*2619	—	—	2003-2860	AY334559	
A*26:20	A*2620	—	—	A0009863, 0520-4727-1	AY561761, AJ629868, AJ629869	
A*26:21	A*2621	—	—	TBC41870	AB169978, AB169979, AB169980	
A*26:22	A*2622	A26(10)	A*2601V1	TBC191371	AB180658, AB180659, AB180660	
A*26:23	A*2623	A26(10)	A*2601V2	TBC024931	AB182245, AB182246, AB182247	
A*26:24	A*2624	—	—	TBC51967	AB196453	M Satake
A*26:25N	A*2625N	Null	—	40261	AY963804, AY963805, AY963806	D Hiraki
A*26:26	A*2626	A10	—	Cr01-4298	AJ890835	(109)
A*26:27	A*2627	—	—	NT00615, BY00173	DQ205703, DQ205704, EF591027	(33), CK Hurley^b^
A*26:28	A*2628	—	—	VTIS129380	DQ400520, DQ400521	BD Tait
A*26:29	A*2629	A26(10)	—	UKE-A2629	AM183783	T Binder
A*26:30	A*2630	—	—	m11449	DQ494176	(58)
A*26:31	A*2631	—	—	4497370, HSE-10861(FLS)	DQ530236, EF025769	(110), (111)^b^
A*26:32	A*2632	—	—	2006051203	DQ780570	(112)
A*26:33	A*2633	—	—	UKE-SA	AM295253	T Binder
A*26:34	A*2634	—	—	2006071824	EF103189	(113)
A*26:35	A*2635	—	—	BY00166	EF563135	(36)
A*26:36	A*2636	—	—	CHA06-0792, 16149, HENAN11	EU557970, EU627183, EU785343	HY Seong, J Li, B Zhang^b^
A*26:37	A*2637	—	—	200800714	AM981205	(114)
A*26:38	A*2638	—	—	NT01020, HN-718979	FJ797382, FJ792522	CK Hurley, Histogenetics^b^
A*26:39	A*2639	—	—	HN-68453-0, HN-79781-3, HN-60788-4, HN-83682-2, HN-92817-8, HN-25550-0, 93066816	FJ224158, FJ224199, FJ875684, FJ952579, FJ997936, GQ900563, GU191547	Histogenetics, D Fuerst^b^
A*26:40	A*2640	—	—	BY00466	FJ976695	CK Hurley
A*26:41	A*2641	—	—	334452	FN422388	T Lebedeva
A*26:42	A*2642	—	—	HN-8180403	FJ765894	Histogenetics
A*29:01:01:01	A*29010101	A29(19)	A2901W652R	JOE, W652R, AKB96676, B9545	M23739, U83415, AJ303359, EU445477	(30)
A*29:01:01:02N	A*29010102N	Null	A*GBnu29	GBnu29	AJ293507	
A*29:01:02	A*290102	A29(19)	—	B25468	FJ355924	(115)
A*29:02:01	A*290201	A29(19)	A29.2	LAM, HSR131658, MANN	X60108, AM944568, CR382333	(44)^b^, S Beck^b^
A*29:02:02	A*290202	A29(19)	—	VTIS84092, NT00767	AY216266, AY216267, EU330467	CK Hurley^b^
A*29:02:03	A*290203	A29(19)	—	218584	AJ629870, AJ629871	
A*29:02:04	A*290204	A29(19)	—	HN-97418-1, HN24104-4, HN-1915-3, HN-38969-3	FJ224176, FJ224189, FJ976751, GQ149251	Histogenetics
A*29:02:05	A*290205	A29(19)	—	333143	FN422391	T Lebedeva
A*29:03	A*2903	—	—	CMD004AN	Y09218, AJ000661	
A*29:04	A*2904	—	—	NM3234	AF042188, AF042189	
A*29:05	A*2905	—	—	BY0020	AY042684, AY042685	
A*29:06	A*2906	—	—	GN00412	AY062005, AY062006	
A*29:07	A*2907	—	—	LB56816	AJ506194, AJ506195	
A*29:08N	A*2908N	Null	—	Bes11737	AJ519932, AJ519933, AJ519934	
A*29:09	A*2909	—	—	GN00429, NT00765	AY229983, AY229984, EU330464	CK Hurley^b^
A*29:10	A*2910	A29(19)	—	03119486/BC, VTIS115031	AJ575096, AJ575097, DQ417098, DQ417099	BD Tait^b^
A*29:11	A*2911	—	—	DNA#0283-MHH-V, ESKOM105-884-45	AJ580596, AJ580597, AY623048	
A*29:12	A*2912	—	—	VTIS121184, BY00187	AY826754, AY826755, EF656472	BD Tait, CK Hurley^b^
A*29:13	A*2913	—	—	VTIS123222	AY826756, AY826757	BD Tait
A*29:14	A*2914	—	—	NT00588	DQ086784, DQ086785	(33)
A*29:15	A*2915	—	—	m11842	DQ494177	(58)
A*29:16	A*2916	A29(19)	29GHATT03	FP3924	AM410904	(116)
A*29:17	A*2917	—	—	BY00204	EU029772	(36)
A*29:18	A*2918	—	—	GACH	AM493680	V Dubois
A*29:19	A*2919	—	—	BY00367	FJ464355	CK Hurley
A*29:20	A*2920	—	—	HN-18834-1	FJ222559	Histogenetics
A*29:21	A*2921	—	—	HN-68275-4	FJ875544	Histogenetics
A*29:22	A*2922	—	—	DEDK698352	FN582331	SGE Marsh
A*30:01:01	A*300101	A30(19)	A30.3, A30RSH	LBF, RSH, B10455, SZ-55	M30576, M28414, U07234, EU445478, FJ969875	(30)^b^, HY Zou^b^
A*30:01:02	A*300102	A30(19)	—	ESKOM105-884-45	AY623049	
A*30:02:01	A*300201	A30(19)	A30.2	CR-B, T.B.B.	X61702, AF148862	
A*30:02:02	A*300202	A30(19)	—	ESKOM103-124-02	AY621109	
A*30:02:03	A*300203	A30(19)	—	38645S, 38528S	AY619993, AY619994	S Adams
A*30:02:04	A*300204	A30(19)	—	NT00739	EU146151	CK Hurley
A*30:02:05	A*300205	A30(19)	—	BY00580	GU256009	CK Hurley
A*30:03	A*3003	A30(19)	A30JS	JS, HT	M93657	
A*30:04	A*3004	A30(19)	A*30AD, A30W7, A30JW	AD7563, W7(CC), ASE, TBC62791	U18988, U19734, Z34921, X83770, X83771, AB282891	M Satake^b^
A*30:06	A*3006	—	—	CS48	AF028713, AF028714	
A*30:07	A*3007	—	—	318-409, NT00764	AF065642, AF065643, EU330463	CK Hurley^b^
A*30:08	A*3008	—	—	I3753, NT01009	AJ249308, AJ249309, AJ249310, AJ249311, AJ249312, AJ249313, AJ249314, AJ249315, EU924801	CK Hurley^b^
A*30:09	A*3009	—	A*3002V	99-2196, GN00351	AF198350, AF198351, AF266529, AF266530	
A*30:10	A*3010	—	—	E249, VTIS129599	AF323494, AF323495, AF323496, DQ400532, DQ400533	BD Tait^b^
A*30:11:01	A*301101	A30(19)	A*30New	19302, 23031	AJ308423, AJ308424	
A*30:11:02	A*301102	A30(19)	—	BY00146	EF422076	CK Hurley
A*30:12	A*3012	—	—	0995970	AF480841	
A*30:13	A*3013	—	—	NT00513	AY867796, AY867797	(33)
A*30:14L	A*3014L	Low A30(19)	—	986125	AY323229	K Hirv
A*30:15	A*3015	A30(19)	—	VTIS126747	AY829225, AY829226	BD Tait
A*30:16	A*3016	—	—	NT00643, NT01053	DQ354436, DQ354437, FJ976710	(33), CK Hurley^b^
A*30:17	A*3017	A30(19)	—	118641, 118642	DQ409216	A Amoroso
A*30:18	A*3018	A30(19)	—	SZ-4	DQ872509	(117)
A*30:19	A*3019	—	—	265969	EF050037	K Hirv
A*30:20	A*3020	—	—	34456, B15464	EF207233, EF471208	(118), LYan^b^
A*30:22	A*3022	—	—	BY00182	EF656467	(36)
A*30:23	A*3023	—	—	LUMC-A16	AM746480	(64)
A*30:24	A*3024	—	—	78175	AM778131	J Enczmann
A*30:25	A*3025	—	—	4123	EU623422	(119)
A*30:26	A*3026	—	—	BY00355	FJ196596	CK Hurley
A*30:27N	A*3027N	Null	—	CHO2008-H1	FJ185174	E Trachtenberg
A*30:28	A*3028	—	—	BY00378	FJ464344	CK Hurley
A*30:29	A*3029	—	—	HN-63814-0, HN-8355385, HN-62731-2, HN-20282-0, HN-5746123, HN-08330-2, HN-68905-1, HN-54110-1, HN-54123-4	FJ222566, FJ875686, FJ976741, FJ976748, FJ976733, GQ180292, GQ180303, GQ240351, GQ240352, GQ345035	Histogenetics
A*30:30	A*3030	—	—	BY00520	GQ867214	CK Hurley
A*30:31	A*3031	—	—	HN-2517600	FJ032366	Histogenetics
A*30:32	A*3032	—	—	HN-0546137	FJ765886	Histogenetics
A*30:33	A*3033	—	—	JMDP01K045	AB535154	K Tadokoro
A*30:34	A*3034	—	—	BY00564	GU138062	CK Hurley
A*31:01:02	A*310102	A31(19)	—	KRC033, TB, KRC110, JHAF, KT12, 0229, MHHZ-00006783, B9736, SZ-54	M30578, M28416, M84375, M86405, L78918, AJ239045, AJ239046, AJ889845, EU445479, FJ969876	R Blascyzk^b^, (30)^b^, HY Zou^b^
A*31:01:03	A*310103	A31(19)	—	CB0120020005	AM503964	(120)
A*31:01:04	A*310104	A31(19)	—	HN-5934489	FJ358622	Histogenetics
A*31:01:05	A*310105	A31(19)	—	HN-7047696	FJ358632	Histogenetics
A*31:01:06	A*310106	A31(19)	—	HN-1713861	FJ792524	Histogenetics
A*31:02	A*3102	A31(19)^c^	—	NM2492	AF041369, AF041370	
A*31:03	A*3103	—	A*3101v	NDS-MA	AF067438, AF067439	
A*31:04	A*3104	A31(19)	A31V	NMDP#013528641, NMDP#012891701, NMDP#012797924, T.B.B.	AF105027, AF105028, AF148863	
A*31:05	A*3105	A31(19)	A3101V1, A31v(19)	JCBT1569, JMDP36K032	AB032597, AB477101	K Tadokoro^b^
A*31:06	A*3106	—	—	2000-133-482, LB63242, LB63243, LB63245	AF440106, AF440107, AJ506789, AJ506790	
A*31:07	A*3107	—	—	BY00041	AY094132, AY094133	
A*31:08	A*3108	—	A*19New, A31CT, A*2416	DD3, CRT	AF053481, AF053482, AF012767, AJ011699, AJ011700	
A*31:09	A*3109	—	—	A3564, NT01043, NT01052	AY128676, FJ797355, FJ976709	CK Hurley^b^
A*31:10	A*3110	A31(19)	A*3101V2	TBCT4778	AB182327, AB182587, AB182588	
A*31:11	A*3111	—	A*3101V3	04082410, 04100618, TBC51252	AY786408, AY786409, AB196452	M Satake^b^
A*31:12	A*3112	A31(19)	—	VTIS123281, NT00655, VTIS144743	AY826758, AY826759, DQ436820, DQ436821, EF088204	BD Tait, CK Hurley^b^
A*31:13	A*3113	—	—	HZ7146	DQ206619, DQ206620, DQ206621	(121)
A*31:14N	A*3114N	Null	—	A31-Null	DQ899174	(122)
A*31:15	A*3115	—	—	RA1139, BY00203	EF123262, EU029773	M Yu, CK Hurley^b^
A*31:16	A*3116	—	—	BY00161, 00153823	EF490364, FM995167	(36), (123)^b^
A*31:17	A*3117	—	A*32new	CB4884, Xian28632, BY00369	EF471207, EF602748, FJ464353	(124), M Liu^b^, CK Hurley^b^
A*31:18	A*3118	—	—	BY00172, BY00381,	EF536141, FJ464341	(36), CK Hurley^b^
A*31:19	A*3119	—	—	MHHN-574599	AM749668	R Blasczyk
A*31:20	A*3120	—	—	YC072707, YC081007, YC081607	EU111805	(125)
A*31:21	A*3121	—	—	NT00738	EU146152	CK Hurley
A*31:22	A*3122	—	—	12948	EU580147	J Li
A*31:23	A*3123	A31(19)	—	ROOS4408AN	FM177143	AM Little
A*31:24	A*3124	—	—	BY00372	FJ464350	CK Hurley
A*31:25	A*3125	—	—	08215667	FJ868755	A Vigh
A*31:26	A*3126	—	—	HN-28488-4, HN-02272-3, HN-52155-8, HN-87418-8, HN-69195-0, HN-88007-2, HN-70217-3, HN-62998-9, HN-15148-9, HN-45545-3, HN-78362-1, HN-79551-8, HN-98435-1, HN-53488-9, HN-53647-0, HN-38472-2, HN-72564-3	FJ224134, FJ224156, FJ224180, FJ224188, FJ224204, FJ224206, FJ765915, FJ976734, FJ976743, FJ976754, GQ160988, GQ160989, GQ160992, GQ240527, GQ240528, GQ449620, GQ859507	Histogenetics
A*31:27	A*3127	—	—	HN-27846-7, HN-73973-6, HN-82794-0, HN92083-2, HN-51328-8, HN-86572-0	FJ224182, FJ765932, GQ161013, GQ254316, GQ449625, GQ914776	Histogenetics
A*31:28	A*3128	—	—	CMHRN1660	FJ785520	M Yu
A*32:01:01	A*320101	A32(19)	—	AM, SSTO, B9188	P10314, AJ555413, BX005091, EU445480	S Beck^b^, (30)^b^
A*32:01:02	A*320102	A32(19)	—	92508	EF471360	MS Leffell
A*32:01:03	A*320103	A32(19)	—	BY00190	EU029785	(36)
A*32:01:04	A*320104	A32(19)	—	HN-93481-8, HN-26034-0, HN-30229-2, HN-00356-4, HN-25975-5, HN-68512-9	FJ619392, FJ976750, FJ224236, GQ160971, GQ161005, GQ240542	Histogenetics
A*32:02	A*3202	A32(19)	—	MP	X97120	
A*32:03	A*3203	—	—	023-8001, VTIS70350	AF072761, AF072762, AF517561, AF517562	
A*32:04	A*3204	—	A*0301V	GN00277, GN00278, NT00998	AF139891, AF139892, AF137077, AF137078, EU872418	CK Hurley^b^
A*32:05	A*3205	—	A*32New	CL183	AF217560	
A*32:06	A*3206	—	A*3201V	GN00338	AF226836, AF226837	
A*32:07	A*3207	—	—	GN00388, NT00761	AF359389, AF359390, EU330460	CK Hurley^b^
A*32:08	A*3208	—	—	231826, 232319, 232318, 232558, NT00520	AJ629317, AJ629318, AY867800, AY867801	CK Hurley^b^
A*32:09	A*3209	—	—	VTIS125394	AY829215, AY829216	BD Tait
A*32:10	A*3210	—	—	MHHZ-00011364	AM039954	R Blasczyk
A*32:11Q	A*3211Q	—	—	BY00069	DQ123858, DQ123859	(88)
A*32:12	A*3212	—	—	MHHN-154465	AM282701	R Blasczyk
A*32:13	A*3213	—	—	BY00128	DQ898275	(33)
A*32:14	A*3214	A32(19)	—	VTIS138700	EF088205	BD Tait
A*32:15	A*3215	—	—	R30700	AM422702	(91)
A*32:16	A*3216	—	—	268059	EU410616	J Mytilineos
A*32:17	A*3217	—	—	Scu-5154, Scu06157, BY00369	FJ153228, FJ464353	(126), CK Hurley^b^
A*32:18	A*3218	—	—	BY00370, BY00371, HN-32257-6, HN-44089-8	FJ464351, FJ464352, FJ224215, FJ976867	CK Hurley, Histogenetics^b^
A*32:19N	A*3219N	Null	—	CTM-1003575	FJ951633	(127)
A*32:20	A*3220	—	—	HN-72101-8, HN-33132-1	FJ224133, FJ224219	Histogenetics
A*32:21	A*3221	—	—	BY00581	GU256008	CK Hurley
A*33:01:01	A*330101	A33(19)	Aw33.1, A3301W776R	JOE, LWAGS, LCL80, W776R, SZ-60	M30580, M28415, U18989, U19735, X83004-5, U83416, FJ969869	HY Zou^b^
A*33:01:02	A*330102	A33(19)	—	HN-12761-7	FJ224208	Histogenetics
A*33:01:03	A*330103	A33(19)	—	HN-36756-8, HN-24391-3	FJ224192, GQ914775	Histogenetics
A*33:03:01	A*330301	A33(19)	A33NC, A33MK	CTM4955926, GAO801, LCL82, HOR, IT, B9445, SZ-53, 7550800649	U09740, U18990, U19736, X83002-3, L06440, EU445481, FJ969877, GQ996943	(30)^b^, HY Zou^b^, Y Xu^b^
A*33:03:02	A*330302	A33(19)	—	6A670	AY289105, AY289106	
A*33:03:03	A*330303	A33(19)	—	p3540	FJ560746	(69)
A*33:03:04	A*330304	A33(19)	—	244224	FN422386	T Lebedeva
A*33:04	A*3304	—	—	NM2442	AF041367, AF041368	
A*33:05	A*3305	A33(19)	A*33DU	DU, NM5A679, Leiden-QC1504	AF108447, AF108448, AF268401, AF268402, AJ251541	
A*33:06	A*3306	—	A33 variant	ASM	AF234539, AF234540, AF234541	
A*33:07	A*3307	—	—	Baba	AJ537422, AJ537423, AJ564994	
A*33:08	A*3308	A33(19)	—	HZCB2832, BY00116	DQ089631, DQ089632, DQ782333	(128), CK Hurley^b^
A*33:09	A*3309	A19	—	D21977, BY00194	AM410969, EU029782	(129), CK Hurley^b^
A*33:10	A*3310	—	—	NT00715	EF375695	(85)
A*33:11	A*3311	—	—	BY00163, BY00181	EF536019, EF656466	(36), CK Hurley^b^
A*33:12	A*3312	—	—	BY00180	EF591032	(36)
A*33:13	A*3313	—	—	BY00185	EF656470	(36)
A*33:14	A*3314	—	—	LWS-061204	EU399237	(130)
A*33:15	A*3315	—	—	7112601	EU478436	(131)
A*33:16	A*3316	—	—	OLMEMIGU01	EU480687	P Stastny
A*33:17	A*3317	—	—	ZJCB5940	EU554565	(69)
A*33:18	A*3318	—	—	SZ-14	EU665684	(132)
A*33:19	A*3319	—	—	BY00326	EU827505	CK Hurley
A*33:20	A*3320	—	—	YC040208	EU98095	Y Chang
A*33:21	A*3321	—	—	BY00354	FJ187801	CK Hurley
A*33:22	A*3322	—	—	CTJ-23230	FJ200654	L Yan
A*33:23	A*3323	—	—	BY00376	FJ464346	CK Hurley
A*33:24	A*3324	—	—	NT01062	FJ976688	CK Hurley
A*33:25	A*3325	A33(19)	—	PT08-278, PT08-283	GQ199711, GQ214006	MH Park
A*33:26	A*3326	—	—	HN-69923-0, HN-70027-7, HN-68836-6, HN-47189-8, HN-61409-1	FJ224138, FJ224139, FJ224161, FJ224200, GQ401182	Histogenetics
A*33:27	A*3327	—	—	HN-86603-7, HN-01766-3, HN-55084-3	FJ594710, GQ160973, GQ240543	Histogenetics
A*33:28	A*3328	—	—	BY00513	GQ426486	CK Hurley
A*33:29	A*3329	—	—	HN-7429324	FJ792528	Histogenetics
A*34:01:01	A*340101	A34(10)	—	ENA, QC358, B10328	X61704, AM748385, EU445482	P Dunn^b^, (30)^b^
A*34:01:02	A*340102	A34(10)		QC358	AM748386	(133)
A*34:02	A*3402	A34(10)	—	WWAI, NT00702	X61705, EF370117	CK Hurley^b^
A*34:03	A*3403	—	A*3402V	1998-302-1407, GN00377	AF251352, AF251353, AF315685, AF315686	
A*34:04	A*3404	A34(10)	A34new	ATG	AJ297499, AJ297500	
A*34:05	A*3405	—	—	R211793, GN00431	AJ507647, AJ507648, AY267909, AY267910	
A*34:06	A*3406	—	—	33041S	AY509617	
A*34:07	A*3407	—	—	NT00617	DQ205713, DQ205714	(33)
A*34:08	A*3408	—	—	NT00653, NT00684	DQ436814, DQ436815, EF078984	(33)
A*36:01	A*3601	A36	—	MASCH, NT00694	X61700, EF156374	CK Hurley^b^
A*36:02	A*3602	—	A*3601V	GN00347	AF244504, AF244505	
A*36:03	A*3603	A36	—	HC030101, F.G., NT00685	AF384666, EF173479	CK Hurley^b^
A*36:04	A*3604	A36^c^	—	NH29	AF323849, AF323850	
A*36:05	A*3605	—	—	170705	FN557302	E Keller
A*43:01	A*4301	A43	—	CC, GN00174	X61703, AF008305, AF008306	
A*66:01	A*6601	A66(10)	—	25/1506, TEM, GU5175	X61711, U17571	
A*66:02	A*6602	A66(10)	—	CR-B, MALS, HUT102	X61712, X51745	
A*66:03	A*6603	A10	A66KA	AKI, 29441	X96638, AM423239	T Gervais^b^
A*66:04	A*6604	—	—	BY00015	AF321832, AF321833	
A*66:05	A*6605	—	—	20020721CB	AB213609	H Inoko
A*66:06	A*6606	A66(10)	—	VTIS132513	DQ400518, DQ400519	BD Tait
A*66:07	A*6607	—	—	BJ061913	EU140343	X Shan
A*66:08	A*6608	A10	—	HUAF247AN	FM177142	AM Little
A*66:09	A*6609	—	—	192741, HN-50709-6	FM955271, FJ997934	K Witter, Histogenetics^b^
A*66:10	A*6610	—	—	Sunxianjun	FJ810056	J He
A*66:11	A*6611	—	—	CHN92081, HN-92081	GQ856688, FJ907384	H Alves, Histogenetics^b^
A*66:12	A*6612	—	—	HN-19916-4, HN-07421-8	FJ765944, FJ785209	Histogenetics
A*68:01:01	A*680101	A68(28)	Aw68.1	LB, 10063349	X03070, X03071, AF106692, AJ315642	
A*68:01:02	A*680102	A68(28)	Aw68.1	GRC187, NT00653, B10496	L06425, EF204524, EU445483	CK Hurley^b^, (30)^b^
A*68:01:03	A*680103	A68(28)	—	CB13659	AY263395, AY263396	
A*68:01:04	A*680104	A68(28)	—	2005-1737	AM062639	(134)
A*68:01:05	A*680105	A68(28)	—	m1216	DQ494175	(58)
A*68:01:06	A*680106	A68(28)	—	R241, R242	EF488367	(135)
A*68:01:07	A*680107	A68(28)	—	94333	EF656475	MS Leffell
A*68:02:01:01	A*68020101	A68(28)	Aw68.2	PA, TO, 86988	U03861, AJ844895, AM235885	F Poli^b^
A*68:02:01:02	A*68020102	A68(28)	—	125400	AM235886	(136)
A*68:02:01:03	A*68020103	A68(28)	—	CTM-3695374	EF502101	(137)
A*68:02:02	A*680202	A68(28)	—	BRABRI, NT00753, HN-5310359, HN-5575987, HN-1018683	EU275161, EU189866, FJ765880, FJ765887, FJ765879	(47), CK Hurley^b^, Histogenetics^b^
A*68:02:03	A*680203	A68(28)	—	HN-69877-5	FJ785206	Histogenetics
A*68:02:04	A*680204	A68(28)	—	HN-710638	FJ792521	Histogenetics
A*68:03:01	A*680301	A68(28)^c^	A*68new-69A, A68N	AA859, PIME, 69A, FC	U41057, U56436, U56437, U43336, AF017311, U89946	
A*68:03:02	A*680302	A68(28)^c^	A68N2	GP	U89947	
A*68:04	A*6804	A68(28)^c^	A*68new-65A	65A	U41844, AF017312	
A*68:05	A*6805	A68(28)^c^	A*68new-67A	67A, NT01006	U43335, AF017313, EU924805	CK Hurley^b^
A*68:06	A*6806	—	A*6801Var	GN00156, BY00563	U91627, U91628, GU138063	CK Hurley^b^
A*68:07	A*6807	—	—	NM2514, BY00567	AF041371, AF041372, GU144508	CK Hurley^b^
A*68:08:01	A*680801	A68(28)	A68V	TER#934	AJ223972	
A*68:08:02	A*680802	A68(28)	—	999624, BY00579	FJ196839, GU256010	MS Leffell, CK Hurley^b^
A*68:09	A*6809	—	—	262-492	AF072769, AF072770	
A*68:10	A*6810	A28^c^	A*68011Variant	346-00642, NT00749	AF108430, AF108431, EU256490	CK Hurley^b^
A*68:11N	A*6811N	Null	A68Null	HP2, OV	AF101046	
A*68:12	A*6812	A28	A*68New	KE-GF, NT01008	AJ238362, AJ238363, AJ238364, EU924803	CK Hurley^b^
A*68:13	A*6813	—	A*68KM	FAH, 34687	AJ238523, AJ238151, AJ238152, AJ238153, AM072964	T Gervais^b^
A*68:14	A*6814	—	A*68xx	NMDP0247-8661-2	AF145954, AF145955	
A*68:15	A*6815	—	A*6802V	GN00261, GN00299	AF135544, AF135545, AF181103, AF181104	
A*68:16	A*6816	A68(28)	A68PA	PA87, NT00731	AF144013, EF563144	CK Hurley^b^
A*68:17	A*6817	A28^c^	A*68Dan	K45, NM5A815	AJ245567, AF268397, AF268398	
A*68:18N	A*6818N	Null	A*68BLA	BLA-Fab	AJ278501	
A*68:19	A*6819	—	A*68012V	GN00376, GN00410	AF288049, AF288050, AF408168, AF408169	
A*68:20	A*6820	—	—	GN00389	AF359391, AF359392	
A*68:21	A*6821	—	—	2001-7399	AF479818, AF479819	
A*68:22	A*6822	—	—	K83467	AJ420528	
A*68:23	A*6823	—	—	A3783	AY128678	
A*68:24	A*6824	—	—	Carla, RHD	AJ538194, AJ538195, AJ550620, AJ550621	
A*68:25	A*6825	—	—	1443266, NT00512	AY368501, AY368502, AY608896, AY608897	
A*68:26	A*6826	A28	—	144176	AJ633570	
A*68:27	A*6827	—	—	ESKOM105-370-35, Virologie-493	AY621108, AJ634258, AJ634259	
A*68:28	A*6828	—	—	NT00515	AY867798, AY867799	(33)
A*68:29	A*6829	—	—	NT00619	DQ205717, DQ205718	(33)
A*68:30	A*6830	—	—	NT00616	DQ205709, DQ205710	(33)
A*68:31	A*6831	—	—	NT00644, NT00778, BY00175	DQ354432, DQ354433, EU484052, EU847252	(33), CK Hurley^b^
A*68:32	A*6832	—	—	VTIS12881	DQ400522, DQ400523	BD Tait
A*68:33	A*6833	A68(28)	—	20725, 20726	DQ438173, DQ438174, DQ438175	M Aubrey
A*68:34	A*6834	—	—	NT00669	DQ648004	(33)
A*68:35	A*6835	—	—	A016699, MHHZ-00016776	DQ788799, AM286801	W Dong, R Blasczyk^b^
A*68:36	A*6836	A68(28)	—	CTM-5098515, BY00191, scu00946	EF062994, EU029784, EU189033	(138), CK Hurley^b^, (139)^b^
A*68:37	A*6837	—	A*68MVE0906	MHHN-189456	AM407882	R Blasczyk
A*68:38	A*6838	—	—	MHHN-266949	AM493251	R Blasczyk
A*68:39	A*6839	—	—	BY00165	EF563134	(36)
A*68:40	A*6840	—	—	BY00188	EF656473	(36)
A*68:41	A*6841	—	—	G0424060013350	EU048251	C Cormack
A*68:42	A*6842	—	—	NT00752	EU275152	(85)
A*68:43	A*6843	—	—	DOPIRos	AM950323	A Dormoy
A*68:44	A*6844	—	—	BY00361	FJ358711	CK Hurley
A*68:45	A*6845	—	—	BY00368	FJ464354	CK Hurley
A*68:46	A*6846	—	—	BY00390	FJ517142	CK Hurley
A*68:47	A*6847	—	—	BY00445	FJ797354	CK Hurley
A*68:48	A*6848	—	—	NT01063	FJ976687	CK Hurley
A*69:01	A*6901	A69(28)	—	IDF, ZM, BJ, NT00693, B11264, SZ-71	X03158, X03159, EF156373, EU445484, FJ969870	CK Hurley^b^, (30)^b^, HY Zou^b^
A*74:01	A*7401	A74(19)	—	CC, PDAV, ATUR, GU2037, GU2040	X61701, U17569, U17570	
A*74:02	A*7402	A74(19)	A*74dc	DCH-HLA05, BT2358, SZ-65	X95409, AJ223060, FJ969871	HY Zou^b^
A*74:03	A*7403	A74(19)	A*74pb	PEB, JB-R.B., F468	X95561, AJ002678, DQ870546	(140)^b^
A*74:04	A*7404	—	A*74New	U3765	AJ249370	
A*74:05	A*7405	—	—	NM5A142, NT01065	AF255720, AF255721, FJ976712	CK Hurley^b^
A*74:06	A*7406	A74(19)	—	VTIS23531, NT00996, BY00365	AF329872, AF329873, EU872415, FJ376479	CK Hurley^b^
A*74:07	A*7407	A74(19)	—	BY0021, CHATChr	AY050187, AY050188, AM422778	A Dormoy^b^
A*74:08	A*7408	—	—	2001-40-660, BY00383	AF440110, AF440111, FJ464339	CK Hurley^b^
A*74:09	A*7409	—	—	A3682, NT00692	AY128675, EF156376	CK Hurley^b^
A*74:10	A*7410	—	—	JW1182646	AJ581659	
A*74:11	A*7411	—	—	NT00585, NT00585, BY00195	DQ086788, DQ086789, DQ888175, EU029781	(107), CK Hurley^b^
A*74:12N	A*7412N	Null	—	LCLAg876	AJ865349	K Witter
A*74:13	A*7413	—	—	BY00352, BY00459	FJ187800, FJ842971	CK Hurley
A*74:14N	A*7414N	Null	—	08218395	FJ868756	A Vigh
A*80:01	A*8001	A80	AX “BG”, A-new	VH, 35020, 35841, 32511, CODI, MIKA, LADA, CTM3953540, CTM1953541	M94880, L18898, L19403, U03754	

a Allele names given in bold type have been assigned since the 2004 Nomenclature report.

b This reference is to a confirmatory sequence.

c HLA specificity provided from the HLA dictionary (22–26).

In February 2005 the allele A*30:14L was named. The allele has a mutation in codon 164 encoding a cysteine residue contributing to a structurally critical disulphide bond in the α2 domain of the HLA molecule. Expression studies performed on cells with this allele showed its protein to have a much-reduced expression compared to normal, and the allele name was thus given the suffix ‘L’ to indicate this low expression. Since then several other alleles have been reported that have also lost one of the two cysteine residues (position 101 and 164) that form the α2 domain disulphide bond. It has not, however, been possible to ascertain the expression status of these alleles, due to a lack of viable material. The Nomenclature Committee considered the naming of these alleles during the 14^th^ HLA and Immunogenetics Workshop. As a result of these discussions, it was decided to introduce an additional suffix, Q, to indicate a ‘Questionable’ expression level. The first seven alleles to receive this suffix have been named and are included in this report, A*23:19Q, A*32:11Q, B*13:08Q, B*35:65Q, B*39:38Q, C*02:25Q and C*03:22Q. It is anticipated that when further examples of these alleles are described, their expression status will be determined and the suffix changed accordingly.

As the database of HLA allele sequences has expanded, it has become increasingly difficult to maintain consistent linkage between allele names assigned on the basis of nucleotide sequences and the serological profiles of the encoded proteins. These difficulties are in part technological and in part due to the inherent biological properties of the HLA system. In the first category there is the increasing emphasis on DNA technology and consequent lack of a serological description for many newly discovered HLA alleles. In the second category is the finding that a newly defined antigen does not comfortably fit within any known serological grouping. This is especially true of the HLA-DRB1*03, *11, *13, *14 and *08 family of alleles, for which the description of new alleles has revealed a continuum of allelic diversity rather than five discrete sub-families. It should be stressed that, although a goal is to indicate the serological grouping into which an allele will fall, this is not always possible. Most importantly the allele name should be seen as no more than a unique designation.

## 2. Serological specificities associated with alleles

Where this information is known, lists of the serological specificities or antigens associated with the alleles, is given in Tables 2–7. In most cases these data are based on the serological typing obtained for the cells that were sequenced for the individual alleles and from information submitted to the Committee. In many cases no serological information is available and the entry in the table has been left blank. This is also true for cases when the serological pattern associated with an expressed allele does not correspond to a single defined specificity. A comprehensive dictionary of antigen and allele equivalents is published periodically by the World Marrow Donor Association (WMDA) Quality Assurance Working Group on HLA Serology to DNA Equivalents (22–26). Where additional or superior serological data are available from the dictionary, this has been included in Tables 2–7 and the source of this information indicated. A full list of all officially named serological specificities is given in Table 15. The specificity B82 was assigned following the 14^th^ International HLA and Immunogenetics Workshop in 2005 having been clearly identified as a novel antigen in a number of UCLA cell exchanges.

**Table 15:** List of all recognised serological and cellular HLA specificities

HLA-A	HLA-B	HLA-C	HLA-D	HLA-DR	HLA-DQ	HLA-DP
A1	B5	Cw1	Dw1	DR1	DQ1	DPw1
A2	B7	Cw2	Dw2	DR103	DQ2	DPw2
A203	B703	Cw3	Dw3	DR2	DQ3	DPw3
A210	B8	Cw4	Dw4	DR3	DQ4	DPw4
A3	B12	Cw5	Dw5	DR4	DQ5(1)	DPw5
A9	B13	Cw6	Dw6	DR5	DQ6(1)	DPw6
A10	B14	Cw7	Dw7	DR6	DQ7(3)	
A11	B15	Cw8	Dw8	DR7	DQ8(3)	
A19	B16	Cw9(w3)	Dw9	DR8	DQ9(3)	
A23(9)	B17	Cw10(w3)	Dw10	DR9		
A24(9)	B18		Dw11(w7)	DR10		
A2403	B21		Dw12	DR11(5)		
A25(10)	B22		Dw13	DR12(5)		
A26(10)	B27		Dw14	DR13(6)		
A28	B2708		Dw15	DR14(6)		
A29(19)	B35		Dw16	DR1403		
A30(19)	B37		Dw17(w7)	DR1404		
A31(19)	B38(16)		Dw18(w6)	DR15(2)		
A32(19)	B39(16)		Dw19(w6)	DR16(2)		
A33(19)	B3901		Dw20	DR17(3)		
A34(10)	B3902		Dw21	DR18(3)		
A36	B40		Dw22			
A43	B4005		Dw23	DR51		
A66(10)	B41		Dw24	DR52		
A68(28)	B42		Dw25	DR53		
A69(28)	B44(12)		Dw26			
A74(19)	B45(12)					
A80	B46					
	B47					
	B48					
	B49(21)					
	B50(21)					
	B51(5)					
	B5102					
	B5103					
	B52(5)					
	B53					
	B54(22)					
	B55(22)					
	B56(22)					
	B57(17)					
	B58(17)					
	B59					
	B60(40)					
	B61(40)					
	B62(15)					
	B63(15)					
	B64(14)					
	B65(14)					
	B67					
	B70					
	B71(70)					
	B72(70)					
	B73					
	B75(15)					
	B76(15)					
	B77(15)					
	B78					
	B81					
	B82					
						
	Bw4					
	Bw6					

**Table 7:** Designations of HLA-DQA1 and -DQB1 alleles

HLA allele^a^	Pre 2010 designation	HLA-DQ serological specificities	HLA-D associated (T-cell defined) Specificities	Previous equivalents	Individual or cell line from which the sequences was derived	Accession number	References or submitting author(s)
DQA1*01:01:01	DQA1*010101	—	Dw1	DQA 1.1, 1.9	LG2, BML, KAS116	L34082	
DQA1*01:01:02	DQA1*010102	—		DQA1*0101new	MZ070782, LWAGS, PMG075	AF322867, AF322868, AF322869	
DQA1*01:02:01	DQA1*010201	—	Dw2, w21, w19	DQA 1.2, 1.19, 1.AZH	PGF, LB, CMCC, AZH, WT46, DRA, ROF-NL, EMJ	M20431, L34083	
DQA1*01:02:02	DQA1*010202	—	Dw21	—	KAS011	L34084	
DQA1*01:02:03	DQA1*010203	—	—	—	BM3171	DQ178400, DQ178401, DQ178402, DQ178403	(415)
DQA1*01:02:04	DQA1*010204	—	—	—	24368	AM042559	(416)
DQA1*01:03	DQA1*0103	—	Dw18, w12, w8, Dw’FS'	DQA 1.3, 1.18, DRw8-DQw1	APD, TAB, FPF, WVB, 2012, E4181324	M59802, L34085	
DQA1*01:04:01	DQA1*010401	—	Dw9	—	1183, 2013, 2012, 2708, 31227ABO, EK, KOSE, DEK, REN	M95170, L34086	
DQA1*01:04:02	DQA1*010402	—	—	DQA1*new	KGU	AJ296091, AJ296092	
DQA1*01:05	DQA1*0105	—	—	—	AK93007, 1183, 2708	L42625, L46877	
DQA1*01:06	DQA1*0106	—	—	183DQA1	183		
DQA1*01:07	DQA1*0107	—	—	—	CDC033104	AY585236	
DQA1*02:01	DQA1*0201	—	Dw7, w11	DQA 2, 3.7	LG-10, BEI, DM24, DM28, DM29, MOU	L34087	
DQA1*03:01:01	DQA1*030101	—	Dw4, w10, w13, w14, w15	DQA 3, 3.1, 3.2	MMCC, JY, NIN, BML, DM24, DM29, BOLETH	M29613, M29616, L34088	
DQA1*03:02	DQA1*0302	—	Dw23	DQA 3, 3.1, 3.2, DR9-DQw3	ISK, DKB, YT	M11124, L34089	
DQA1*03:03	DQA1*0303	—	—	—	YT	L34089, L46878	
DQA1*04:01:01	DQA1*040101	—	Dw8, Dw’RSH’	DQA 4.2, 3.8	ARC, 2041, MADURA, SPL (SPACH)^e^	M33906, L34090	
DQA1*04:01:02	DQA1*040102	—	Dw8	—	CDC120202	AY180974	
DQA1*04:02	DQA1*0402	—	—	—	CDC121802	AY197775	
DQA1*04:03N	DQA1*0403N	Null	—	—	1242	AF448425	
DQA1*04:04	DQA1*0404	—	—	—	CDC021704	AY547314	
DQA1*05:01:01	DQA1*050101	—	Dw3, w5, w22	DQA 4.1, 2, dJ93N13	RAJI, CMCC, VAVY, HSF7, SWEIG, QBL	X00370, K01160, L34091, Z84489, AL935026	(105)^b^
DQA1*05:01:02	DQA1*050102	—	Dw5	DQA 4.1, 2	MG3	—	
DQA1*05:02	DQA1*0502	—	—	—	EMA	U03675	
DQA1*05:03	DQA1*0503	—	Dw16	—	AMALA	L34093	
DQA1*05:04	DQA1*0504	—	—	DQA1*05YD, DQA05MC	YD-069, AD-YM23	U85035, U97555	
DQA1*05:05	DQA1*0505	—	Dw5, Dw22	DQA 4.1, 2	BM21, REM (RML), BM16	AB006908, M20506, L34092	
DQA1*05:06	DQA1*0506	—	—	—	BM3114	DQ178404, DQ178405, DQ178406, DQ178407	(415)
DQA1*05:07	DQA1*0507	—	—	—	BM3189	DQ178408, DQ178409, DQ178410, DQ178411	(415)
DQA1*05:08	DQA1*0508	—	—	—	BM3146	DQ178412, DQ178413, DQ178414, DQ178415	(415)
DQA1*05:09	DQA1*0509	—	—	—	20700, 218, 28137	AM042560	(416)
DQA1*05:10	DQA1*0510	—	—	—	CTM-8005076	GU0142876	(417)
DQA1*06:01:01	DQA1*060101	—	Dw8	DQA 4.3	LUY	L34094	
DQA1*06:01:02	DQA1*060102	—	—	—	RV	Y09968	
DQA1*06:02	DQA1*0602	—	—	—	CDC111803	AY206406	
							
DQB1*02:01:01	DQB1*020101	DQ2	Dw3	DQB 2	WT49, CMCC, QBL, MZ, LD, VW, MOR, JNP, DM24, DM28, DM29, BEI, VAVY	K02405, M65043, M81140, L40179	
DQB1*02:01:02	DQB1*020102	DQ2	Dw3	—	NW165	AY619719	
DQB1*02:02	DQB1*0202	DQ2	Dw7	DQB 2	BURKHARDT, BH, MOU	M81141, U07848, L34095	
DQB1*02:03	DQB1*0203	DQ2	—	DQB1*02DL, DQB1*GHA30	RAQ, CAUCA254, CAUCA288, DL-13, GHA30	Z35099, U33329, U39089, U39090, AB002468	
DQB1*02:04	DQB1*0204	—	—	—	CDC111703	AY333121, AY333122	S Cordovado
DQB1*02:05	DQB1*0205	—	—	—	NT00723, NT00758	EF484936, EU275156	(355)
DQB1*03:01:01	DQB1*030101	DQ7(3)	Dw4, w5, w8, w13	DQB 3.1, DQ0301W515R	SWEIG, DQB37, NIN, JHA, JR, JME, DC, JGL, LUY, BML, DM23, MG3, AMALA, W515R, CjAr, CaAr, 06-006, SW	M65040, L34096, U83582, M25325, AY656682	
DQB1*03:01:02	DQB1*030102	DQ7(3)	—	DQB1*03GPT	HM00214	AJ001256, Y10428	
DQB1*03:01:03	DQB1*030103	DQ7(3)	—	—	CHAST03, FFM52459	AM259941, FM995168	(418), C Seidl^b^
DQB1*03:01:04	DQB1*030104	DQ7(3)	—	—	NT00757, NT00786, NT00787, NT00792	EU275157	(355)
DQB1*03:02:01	DQB1*030201	DQ8(3)	Dw4, w10, w13, w14	DQB 3.2	BOLETH, FS, BIN40, WT51, DM24, DM29, JS, MMCC, VW, JNP, JOP, Priess, BrEh, 145b, DaHa	M65038, K01499, L34097, M25326	
DQB1*03:02:02	DQB1*030202	DQ8(3)	—	DQB1*0302New	0096302	AY312054	
DQB1*03:02:03	DQB1*030203	DQ8(3)	—	—	OLI	AM286421	X Lafarge
DQB1*03:02:04	DQB1*030204	DQ8(3)	—	—	106329	AM409256	H Dunckley
DQB1*03:03:02	DQB1*030302	DQ9(3)	Dw23, w11	DQB 3.3	DBB, KOZ, 5112.103, DKB, 06-006	M65039, M60028, L34098, M25328	
DQB1*03:03:03	DQB1*030303	DQ9(3)	—	DQB1*03New	G.C.	AF093815	
DQB1*03:04	DQB1*0304	DQ7(3)	—	DQB1*03HP, *03new	HP, RG, M.M.	M74842, M83770, X76553	
DQB1*03:05:01	DQB1*030501	DQ8(3)^c^	—	DQB1*03KC	G.P., M.A.	X69169, X76554	
DQB1*03:05:02	DQB1*030502	DQ8(3)^c^	—	—	00L53	AJ290396	
DQB1*03:05:03	DQB1*030503	DQ8(3)^c^	—	—	402354872	AJ557776	
DQB1*03:05:04	DQB1*030504	DQ8(3)^c^	—	—	IA	AM231062	(419)
DQB1*03:06	DQB1*0306	DQ3	—	DQB1*MAT	MAT	D78569	
DQB1*03:07	DQB1*0307	—	—	DQB1*D4	D4	Z49215	
DQB1*03:08	DQB1*0308	—	—	—	97-459#1	AJ003005	
DQB1*03:09	DQB1*0309	—	—	DQ3 Var	W469D, W469R	U66400	
DQB1*03:10	DQB1*0310	DQ8(3)	—	DQB1*03new	CTM-8991127	AF195245	
DQB1*03:11	DQB1*0311	—	—	—	VBALA	AF439338	
DQB1*03:12	DQB1*0312	—	—	—	216305	AF469118	
DQB1*03:13	DQB1*0313	—	—	—	10993426	AF479569	
DQB1*03:14	DQB1*0314	—	—	—	GRB2022466	AY762968	
DQB1*03:15	DQB1*0315	—	—	—	171739	AJ854065	
DQB1*03:16	DQB1*0316	—	—	DQB1*03COR	05-0132	DQ026226	N Reinsmoen
DQB1*03:17	DQB1*0317	—	—	—	K32633, K32633F	DQ114427	(420)
DQB1*03:18	DQB1*0318	—	—	—	CDC092905	DQ227421	S Cordovado
DQB1*03:19	DQB1*0319	—	—	—	143576	AM400970	(421)
DQB1*03:20	DQB1*0320	—	—	—	NT00722	EF484939	(355)
DQB1*03:21	DQB1*0321	—	—	—	NT00756	EU275158	(355)
DQB1*03:22	DQB1*0322	—	—	—	175842	AM944346	(422)
DQB1*03:23	DQB1*0323	—	—	—	MHHI-633843	FM200854	(423)
DQB1*03:24	DQB1*0324	—	—	—	JUR-DQ3	FM955320	V Dubois
DQB1*03:25	DQB1*0325	—	—	—	ZMK01	EU770203	ZM Kashi
DQB1*03:26	DQB1*0326	—	—	—	2009-CAP-DL03	FN550110	EKL Yang
DQB1*04:01:01	DQB1*040101	DQ4	Dw15	DQB 4.1, Wa	KT3, YT, HM-K, SW	M13279, L34099, AY656683	
DQB1*04:01:02	DQB1*040102	DQ4	—	—	Bjztt	FJ938169	Z Zhang
DQB1*04:02:01	DQB1*040201	DQ4	Dw8, Dw’RSH’	DQB 4.2, Wa, E1448	ARC, OLN, MZ, 2041, SPL (SPACH), MADURA, RPET01	M33907, M65042, L34100, Z80898	
DQB1*04:03:01	DQB1*040301	—	—	—	6222422	EU410617	J Mytilineos
DQB1*04:03:02	DQB1*040302	—	—	—	AN181945	FN433879	F Poli
DQB1*04:04	DQB1*0404	—	—	—	195212	FN555147	K Witter
DQB1*05:01:01	DQB1*050101	DQ5(1)	Dw1	DQB 1.1, DRw10-DQw1.1	LG2, 45.1, BML, MVL, JR, MDR, WG, DC, KAS116	X03068, M65044, L34101	
DQB1*05:01:02	DQB1*050102	DQ5(1)	—	DQB1*05COT	COT.DA	Y17290	
DQB1*05:02:01	DQB1*050201	DQ5(1)	Dw21	DQB 1.2, 1.21	AZH, FJO, KAS011, HM-K, YH-K	L34102, AY656681	
DQB1*05:02:02	DQB1*050202	DQ5(1)	—	—	J16	AF463516	
DQB1*05:03:01	DQB1*050301	DQ5(1)	Dw9	DQB 1.3, 1.9, 1.3.1	WT52, HU129, HU128, EK	M65047, L34103, L40180	
DQB1*05:03:02	DQB1*050302	DQ5(1)	Dw9	DQB 1.3, 1.9, 1.3.2	AP106, AP109, AP110, AP115	—	
DQB1*05:04	DQB1*0504	DQ5(1)^c^	—	DQB 1.9	DG, R.F.	M65046, M94773	
DQB1*05:05	DQB1*0505	—	—	—	HONO-05	AM259942	(418)
DQB1*06:01:01	DQB1*060101	DQ6(1)	Dw12, w8	DQB 1.4, 1.12	AKIBA, BGE, TAB, E4181324, B.H., B.S.,	L34104, X89194, L40181	
DQB1*06:01:02	DQB1*060102	DQ6(1)	Dw12, w8	DQB1*0601var.	Sk, Rb	M86740	
DQB1*06:01:03	DQB1*060103	DQ6(1)	—	DQ06W649R	W649R	AF000447	
DQB1*06:01:04	DQB1*060104	DQ6(1)	—	—	BJ52	EU594579	Z Zhang
DQB1*06:01:05	DQB1*060105	DQ6(1)	—	—	C143843	GQ355337	(321)
DQB1*06:02:01	DQB1*060201	DQ6(1)	Dw2	DQB 1.5, 1.2	PGF, VYT, 2041, ROF-NL, AMAI, CjAr, CaAr	M20432, M65048, L34105 M25327	
DQB1*06:02:02	DQB1*060202	DQ6(1)	—	—	72330002	AM900764	S Schwab
DQB1*06:03:01	DQB1*060301	DQ6(1)	Dw18, Dw’FS'	DQB 1.6, 1.18	WVB, APD, FPF, 2012, OMW	M65050, M34322, L34106	
DQB1*06:03:02	DQB1*060302	DQ6(1)	—	—	R31718, KN166757	AM293569, AM400237	(91), (424)^b^
DQB1*06:04:01	DQB1*060401	DQ6(1)	Dw19	DQB 1.7, 1.19	CMCC, DAUDI, DM23, LD, WG, EMJ	M65051, L34107	
DQB1*06:04:02	DQB1*060402	DQ6(1)	—	DQB1*0604Variant	GN015	AF113250, U63321	
DQB1*06:04:03	DQB1*060403	DQ6(1)	—	—	WF215-200	DQ503425	(425)
DQB1*06:05:01	DQB1*060501	DQ6(1)	Dw19	DQB 1.8, DQBSLE, 1.19b, 2013-24	CI, KT, MR, 2013	M36472, M59800, M65052	
DQB1*06:05:02	DQB1*060502	DQ6(1)	Dw19	DQB1*MDvR-1	BEN53	L26325	
DQB1*06:06	DQB1*0606	—	—	DQB1*WA1	LINE66	M86226	
DQB1*06:07	DQB1*0607	—	—	DQB1*06BRI1	08-2779-0, BN151	M87041, AF112463	
DQB1*06:08:01	DQB1*060801	DQ6(1)^c^	—	DQB1*06BRI2	R.W., BM675	M87042, AF112464	
DQB1*06:08:02	DQB1*060802	DQ6(1)^c^	—	—	STFO3416AN	AM279416	AM Little
DQB1*06:09	DQB1*0609	DQ6(1)	—	DQB1*06AA	HO301, TRACHT, N076, AK93022	L19951, L27345, D29918, L42626	
DQB1*06:10	DQB1*0610	—	—	DQB1MC	M.M., M.G., N205, L13, L90	X86327, Z75044	
DQB1*06:11:01	DQB1*061101	DQ1	—	UNM-95-228	#MUD0130-14998	U39086	
DQB1*06:11:02	DQB1*061102	DQ1	—	DQB1*06new1	6658K	AJ012155	
DQB1*06:12	DQB1*0612	DQ1	—	DQB1*06GB	GB002	X96420	
DQB1*06:13	DQB1*0613	—	—	DQB1*0602V	BB-(2)	U77344	
DQB1*06:14:01	DQB1*061401	DQ6(1)	—	DQB1*06EMT	OG00018	AJ001257	
DQB1*06:14:02	DQB1*061402	DQ6(1)	—	—	8201310	EU445579	A Vigh
DQB1*06:15	DQB1*0615	—	—	DQB1*06new2	T890	AJ012156	
DQB1*06:16	DQB1*0616	—	—	052DQB1	052	AF087939	
DQB1*06:17	DQB1*0617	—	—	99-3039	15427-00/01/02	AF181983	
DQB1*06:18	DQB1*0618	—	—	DQB1*06nou	IM0000053	AY026349	
DQB1*06:19	DQB1*0619	—	—	DQB1*0602Variant	ACAR	AF091305	
DQB1*06:20	DQB1*0620	—	—	—	CB846	AF384556, AY124588	
DQB1*06:21	DQB1*0621	—	—	—	EFI#7/#0710	AJ535315	
DQB1*06:22	DQB1*0622	—	—	—	YC0402	AY672649	
DQB1*06:23	DQB1*0623	—	—	—	247621	AY733062	
DQB1*06:24	DQB1*0624	—	—	—	S.C.	AJ964903	P Schranz
DQB1*06:25	DQB1*0625	DQ6(1)	—	—	NS7581, KY577	AB211231	K Watabe
DQB1*06:26N	DQB1*0626N	Null	—	—	BMFamily22583	AY258420	I Humphreys
DQB1*06:27	DQB1*0627	—	—	—	5103245	AJ965439	(426)
DQB1*06:28	DQB1*0628	—	—	—	158308	AM181332	(424)
DQB1*06:29	DQB1*0629	—	—	—	MHHZ-00023226	AM403489	R Blasczyk
DQB1*06:30	DQB1*0630	—	—	—	GN00420	AY094140	CK Hurley
DQB1*06:31	DQB1*0631	—	—	—	6430111	AM490069	A Wölpl
DQB1*06:32	DQB1*0632	—	—	—	B-200702759	AM691798	(427)
DQB1*06:33	DQB1*0633	—	—	—	D963.9	EF622510	M Yu
DQB1*06:34	DQB1*0634	—	—	—	167687, 167688	AM421131	(428)
DQB1*06:35	DQB1*0635	—	—	—	B00062	FJ912900	K Du
DQB1*06:36	DQB1*0636	—	—	—	FFM58536, FFM58536son	FN256435	C Seidl
DQB1*06:37	DQB1*0637	—	—	—	09212635	GQ422610	D Fuerst
DQB1*06:38	DQB1*0638	—	—	—	LUMC-DQB63	FN552709	JDH Anholts
DQB1*06:39	DQB1*0639	—	—	—	LUMC-DQB64	FN552710	JDH Anholts

a Allele names given in bold type have been assigned since the 2004 Nomenclature report.

b This reference is to a confirmatory sequence.

c HLA specificity provided from the HLA dictionary (22–26).

## 3. Introduction of colon delimited HLA allele names

The convention of using a four-digit code to distinguish HLA alleles that differ in the proteins they encode was introduced in the 1987 Nomenclature Report (8). Since that time additional digits have been added, and currently an allele name may be composed of four, six or eight digits dependent on its sequence.

The first two digits describe the allele family, which often corresponds to the serological antigen carried by the allotype. The third and fourth digits are assigned in the order in which the sequences have been determined. Alleles whose numbers differ in the first four digits must differ by one or more nucleotide substitutions that change the amino-acid sequence of the encoded protein. Alleles that differ only by synonymous nucleotide substitutions within the coding sequence are distinguished by the use of the fifth and sixth digits. Alleles that only differ by sequence polymorphisms in introns or in the 5′ and 3′ untranslated regions that flank the exons and introns are distinguished by the use of the seventh and eight digits.

In 2002 we faced the issue of the A*02 and B*15 allele families having more than 100 alleles (17). At that time the decision taken was to name further alleles in these families in the rollover allele families A*92 and B*95 respectively. For *HLA-DPB1* alleles, it was decided to assign new alleles within the existing system, hence once DPB1*9901 had been assigned, the next allele would be assigned DPB1*0102, followed by DPB1*0203, DPB1*0302 etc.

When these conventions were adopted it was anticipated that the nomenclature system would accommodate all the HLA alleles likely to be sequenced. Unfortunately this is not the case, as the number of alleles for certain genes is fast approaching the maximum possible with the current naming convention.

With the ever increasing number of HLA alleles described it has been decided to introduce colons (:) into the allele names to act as delimiters of the separate fields. To facilitate the transition from the old to the new nomenclature, a single leading zero must be added to all fields containing the values 1 to 9 but beyond that no leading zeros are allowed. This will help to lessen any confusion in the conversion to the new style of nomenclature.

Hence

A*01010101 becomes A*01:01:01:01

A*02010102L becomes A*02:01:01:02L

A*260101 becomes A*26:01:01

A*3301 becomes A*33:01

B*0808N becomes B*08:08N

DRB1*01010101 becomes DRB1*01:01:01:01

For allele families that have more than 100 alleles such as the A*02 and B*15 groups it will be possible to encode these in a single series. Thus the A*92 and B*95 alleles have now been renamed in to the A*02 and B*15 allele series. For example:

A*9201 becomes A*02:101

A*9202 becomes A*02:102

A*9203 becomes A*02:103 etc

B*9501 becomes B*15:101

B*9502 becomes B*15:102

B*9503 becomes B*15:103 etc

The names A*02:100 and B*15:100 will not be assigned. In cases of other allele families where the number of alleles reaches 100 these will be numbered sequentially, for example A*24:99 will be followed by A*24:100.

The *DPB1* allele names that have been previously assigned names within the existing system have also be renamed, for example:

DPB1*0102 becomes DPB1*100:01

DPB1*0203 becomes DPB1*101:01

DPB1*0302 becomes DPB1*102:01

DPB1*0403 becomes DPB1*103:01

DPB1*0502 becomes DPB1*104:01 etc

The ‘w’ will be removed from the *HLA-C* allele names, but will be retained in the HLA-C antigen names, to avoid confusion with the factors of the complement system and epitopes on the HLA-C molecule often termed C1 and C2 that act as ligands for the Killer-cell Immunoglobulin-like Receptors.

Cw*0103 becomes C*01:03

Cw*020201 becomes C*02:02:01

Cw*07020101 becomes C*07:02:01:01 etc

Details of the new format allele names are given in column 1 of Tables 2–12, with the previous name listed in column 2. These changes to the HLA Nomenclature will be officially introduced in April 2010. A full listing of old and new HLA allele names will be made available through the IMGT/HLA Database (www.ebi.ac.uk/imgt/hla) (27, 28) and be implemented with the April 2010 release of the database.

## 4. Reporting of ambiguous HLA allele typing

The level of resolution achieved by many of the HLA typing technologies employed today does not always allow for a single HLA allele to be unambiguously assigned. Often it is only possible to resolve the presence of a number of closely related alleles. This is referred to as an ambiguous ‘string’ of alleles. In addition, typing strategies are frequently aimed at resolving alleles that encode differences within the peptide binding domains, but fail to exclude those that differ elsewhere. For some purposes it is helpful to provide codes that aid the reporting of certain ambiguous alleles ‘strings’. The decision was taken to introduce codes to allow for the easy reporting of:

a. HLA alleles that encode for identical peptide binding domains

HLA alleles having nucleotide sequences that encode the same protein sequence for the peptide binding domains (exon 2 and 3 for HLA class I and exon 2 only for HLA class II alleles) will be designated by an upper case ‘P’ which follows the allele designation of the lowest numbered allele in the group.

For example the string of allele names below share the same α1 and α2 domain protein sequence encoded by exons 2 and 3.

A*02:01:01:01/A*02:01:01:02L/A*02:01:01:03/A*02:01:02/A*02:01:03/A*02:01:04/A*02:01:05/A*02:01:06/A*02:01:07/A*02:01:08/A*02:01:09/A*02:01:10/A*02:01:11/ A*02:01:12/A*02:01:13/A*02:01:14/A*02:01:15/A*02:01:17/A*02:01:18/A*02:01:19/A*02:01:21/A*02:01:22/A*02:01:23/A*02:01:24/A*02:01:25/A*02:01:26/A*02:01:27/ A*02:01:28/A*02:01:29/A*02:01:30/A*02:01:31/A*02:01:32/A*02:01:33/A*02:01:34/A*02:01:35/A*02:01:36/A*02:01:37/A*02:01:38/A*02:01:39/A*02:01:40/A*02:01:41/ A*02:01:42/A*02:09/A*02:66/A*02:75/A*02:89/A*02:97:01/ A*02:97:02/A*02:132/A*02:134/A*02:140

This string can be reduced to A*02:01P

b. HLA alleles that share identical nucleotide sequences for the exons encoding the peptide binding domains

HLA alleles that have identical nucleotide sequences for the exons encoding the peptide binding domains (exon 2 and 3 for HLA class I and exon 2 only for HLA class II alleles) will be designated by an upper case ‘G’ which follows the allele designation of the lowest numbered allele in the group.

For example the string shown below consists of alleles that have identical nucleotide sequences in exons 2 and 3.

A*02:01:01:01/A*02:01:01:02L/A*02:01:01:03/A*02:01:08/A*02:01:11/A*02:01:14/A*02:01:15/A*02:01:21/A*02:09/A*02:43N/A*02:66/A*02:75/A*02:83N/A*02:89/A*02:97:01/A*02:97:02/A*02:132/A*02:134/A*02:140

This string can be reduced to A*02:01:01G

These reporting codes will be implemented in April 2010 and will be made available through the IMGT/HLA Database (www.ebi.ac.uk/imgt/hla) (27, 28) and will be implemented with the April 2010 release of the database.

## 5. Gene and protein nomenclature

Discussions took place on the use of nomenclature for defining HLA allele sequences at the gene and protein level. The committee recommended the use of standard genetic nomenclature where gene symbols are in uppercase and italicised and protein symbols are the same as the gene symbols but are not italicised. Using this approach it is possible to discriminate between an allele of the *HLA-A* gene, for example A*03:01 and the expressed protein product of the same gene A*03:01.

Additionally it was recommended that when reporting an ambiguous string of HLA alleles, a forward slash (/) should be used as the separator to indicate ‘or’. When reporting genotypes it was recommended to use a comma (,) to indicate ‘and’. Hence an HLA type may be reported as:

A*02:01/02:09, 03:01; B*07:02, 15:02/15:73; C*03:03, 07:02

## 6. The IMGT/HLA Sequence Database

The IMGT/HLA Sequence Database continues to act as the official repository for HLA sequences named by the WHO Nomenclature Committee for Factors of the HLA System (27, 28). The database contains sequences for all HLA alleles officially recognised by the WHO Nomenclature Committee for Factors of the HLA System and provides users with online tools and facilities for their retrieval and analysis. These include allele reports, alignment tools, and detailed descriptions of the source cells. The online IMGT/HLA submission tool allows both new and confirmatory sequences to be submitted directly to the WHO Nomenclature Committee. New releases of the database are made every three months, in January, April, July and October, with the latest version (release 2.28.0 January 2010) containing 4447 HLA alleles. The database may be accessed via the worldwide web at www.ebi.ac.uk/imgt/hla.

The IMGT/HLA Database is currently supported by the following organisations: Histogenetics, Abbott, Biotest, Invitrogen, One Lambda, Olerup SSP, Gen-Probe, The Anthony Nolan Trust (ANT), The American Society for Histocompatibility and Immunogenetics (ASHI), The European Federation for Immunogenetics (EFI), Innogenetics, BAG Healthcare, Be the Match Foundation and the National Marrow Donor Program.

## List of committee members involved in preparing this report:

*SGE Marsh*, Anthony Nolan Research Institute, London, UK (Chairman)

*ED Albert*, Munich, Germany

*WF Bodmer*, Weatherall Institute of Molecular Medicine, Oxford University, Oxford, UK

*B Dupont*, Sloan-Kettering Institute for Cancer Research, New York, USA

*HA Erlich*, Roche Molecular Systems, Alameda, USA

*M Fernández-Viña,* MD Anderson, Houston, USA

*DE Geraghty*, Fred Hutchinson Cancer Center, Seattle, USA

*R Holdsworth,* Victorian Transplantation and Immunogenetics Service, Melbourne, Australia

*CK Hurley*, Georgetown University, Washington, USA

*M Lau,* UCLA School of Medicine, Los Angeles, USA

*KW Lee,* Hallym University, Anyang City, South Korea

*B Mach*, University of Geneva, Geneva, Switzerland

*M Maiers,* National Marrow Donor Program, Minneapolis, USA

*WR Mayr*, Medical University of Vienna, Vienna, Austria

*CR Müller,* Zentrales Knochenmarkspender-Register, Ulm, Germany

*P Parham*, Stanford University School of Medicine, Stanford, USA

*EW Petersdorf*, Fred Hutchinson Cancer Center, Seattle, USA

*T Sasazuki*, International Medical Centre of Japan, Tokyo, Japan

*JL Strominger*, Harvard University, Cambridge, USA

*A Svejgaard*, State University Hospital, Copenhagen, Denmark

*PI Terasaki*, Los Angeles, USA

*JM Tiercy*, Hôpital Cantonal Universitaire, Geneva, Switzerland

*J Trowsdale*, Cambridge University, Cambridge, UK

Co-opted members:

*RE Bontrop*, Biomedical Primate Research Centre, Rijswijk, The Netherlands

Footnote:

New sequences should be communicated to Prof Steven Marsh via the sequence submission tool of the IMGT/HLA Database to receive official names, www.ebi.ac.uk/imgt/hla.
